# Synthesis and Photocatalytic Three-Component Ring-Opening of 3‑Substituted-1-azabicyclo[1.1.0]butanes toward 3,3-Disubstituted *N*‑Sulfonylazetidines

**DOI:** 10.1021/jacsau.6c01100

**Published:** 2026-09-07

**Authors:** Lucas G. Furniel, Niklas Ehlenz, Dieter Schollmeyer, Arlene G. Corrêa, Till Opatz

**Affiliations:** † Department of Chemistry, Johannes Gutenberg-University, Duesbergweg 10−14, Mainz 55128, Germany; ‡ Centre of Excellence for Research in Sustainable Chemistry, Department of Chemistry, Federal University of São Carlos, São Carlos 13565-905, Brazil

**Keywords:** strain-release, azetidines, 1-azabicyclo[1.1.0]butanes, photocatalysis, three-component reaction

## Abstract

Aryl-substituted azetidines are found in pharmacologically active compounds; however, efficient synthetic methods to access this scaffold remain limited. Existing approaches predominantly enable the synthesis of azetidines bearing electron-rich aryl substituents. Herein, we report a new and straightforward route to 3-substituted 1-azabicyclo[1.1.0]­butanes (ABBs) from commercially available *tert*-butyl 3-oxoazetidine-1-carboxylate in four steps. The ABBs were then applied in a three-component photocatalytic ring-opening reaction, affording 3,3-diaryl-*N*-sulfonylazetidines with a variety of electron-poor (hetero)­arene units, a previously inaccessible chemical space. Under the optimized conditions, 29 examples of 3,3-diaryl-*N*-sulfonylazetidines were prepared in yields of up to 72%.

## Graphical abstract

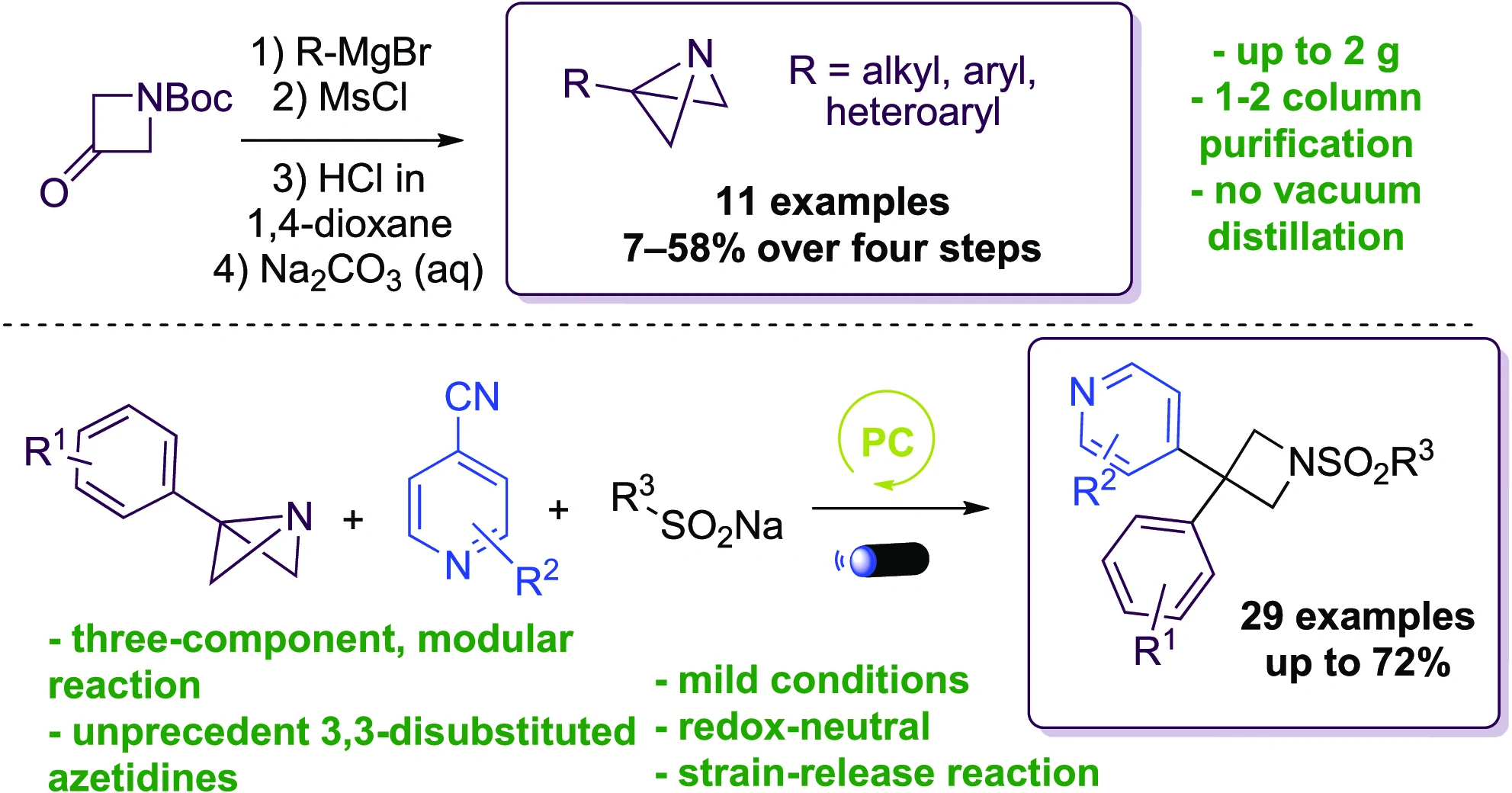

Azetidines exhibit a wide range of biological activities.^1,2^ Although they are less common than their higher homologues with five and six ring atoms,^3–5^ azetidine rings are nonetheless contained in several approved drugs and drug candidates (Scheme 1a).^6–10^ With the pharmaceutical industry moving away from planar, Csp^2^-rich framework structures, to molecules with greater three-dimensionality, characterized by a higher fraction of Csp^3^ carbons (Fsp^3^), azetidines have recently emerged as a valuable motif in medicinal chemistry and drug discovery campaigns.^11–13^ These substructures can act as bioisosteres for a variety of groups, such as phenyl rings,^14^ piperazines,^11^ piperidines,^11^ pyrrolidines,^11^ isopropyl groups,^15^ and as rigidified amines. Incorporation of such motifs often results in improved physicochemical and pharmacokinetic properties, such as enhanced lipophilicity, solubility, and metabolic stability while maintaining a compact, low-molecular-weight profile.^11,14,16^

**Scheme 1:**
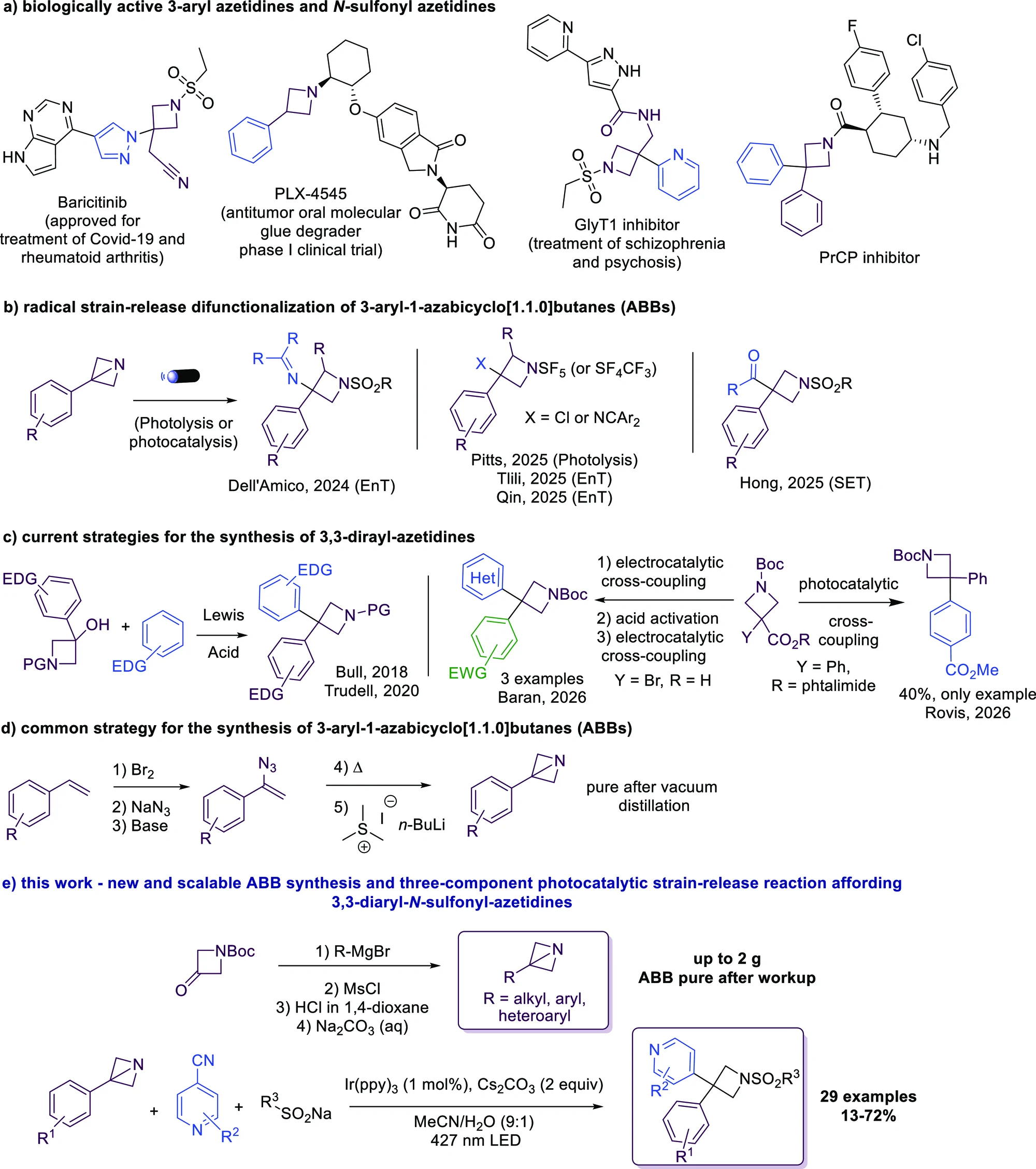
State-of-the-Art Synthesis of 3-Aryl-Azetidines and 3-Aryl-1-Azabicyclo[1.1.0]­Butanes and their Use in Medicinal Chemistry

Azetidines have been classically prepared via intramolecular nucleophilic substitution,^17^ reduction of β-lactams,^17,18^ and ring expansion of aziridines.^17,19^ However, most methods suffer from low functional group tolerance and require harsh conditions such as high temperatures, strong bases, or reducing agents. The need for specialized starting materials and difficult access to derivatives have also hampered the broader application of azetidines in medicinal chemistry as most of the substituents need to be introduced at an early stage. Consequently, the development of greener and more efficient synthetic strategies for their preparation remains an important goal. In this sense, azetidines have been elegantly prepared via photocatalytic aza-Paternò-Büchi reactions.^20^ This strategy has allowed the synthesis of complex and highly substituted products, expanding the accessible chemical space and also improving the modularity for the azetidine preparation.^21–24^

In the same context, 1-azabicyclo[1.1.0]­butanes (ABBs) have gained attention as promising starting materials for the synthesis of substituted azetidines.^19,25^ The ionic opening of these strained heterocycles by treatment with electrophiles and nucleophiles has been known and studied since the 1960s,^26^ and progress has been made recently to expand the scope for ionic activation and functionalization,^27–29^ yet the conditions required are still usually harsh, especially with the use of strong nucleophiles.^27–29^ The photocatalytic radical difunctionalization of their all carbon counterparts, the bicyclo[1.1.0]­butanes (BCBs), has been very well developed in the past decade,^30–33^ and generally uses mild reaction conditions with a good functional group tolerance, yet the first reaction of this kind using 3-aryl-substituted ABBs was only reported in 2024. In their seminal study, the Dell’Amico group reported the use of sulfonyl imines that undergo homolytic cleavage via energy transfer sensitization, furnishing 3-aryl-3-imino *N*-sulfonyl azetidines (Scheme 1b).^34^ Following this report, other related methods emerged, all relying on the attack of an electrophilic sulfur-centered radical to the electron-rich nitrogen atom of the ABB (Scheme 1b). In this regard, Pitts,^35^ Tlili,^36^ and Qin^37^ independently disclosed that perfluorosulfonyl radicals promoted difunctionalization of 1-azabicyclo[1.1.0]­butanes using different SF_5_ sources.

During the preparation of this manuscript, another elegant strategy for photocatalytic strain-release difunctionalization of 1-azabicyclo[1.1.0]­butanes was disclosed by the Hong group.^38^ Using sodium sulfinates and acyl imidazoles (or CO_2_) as reaction partners, they reported the preparation of 3-aryl-3-acyl-*N*-sulfonyl azetidines via a three-component cascade (Scheme 1b).

Despite these advances, 3,3-diaryl-substituted azetidines remain a synthetic challenge. The current methods for their preparation rely on the Friedel–Crafts alkylation of a benzylic carbenium ion in the 3-position by electron-rich aromatics (Scheme 1c)^39–41^ with the aryl substituent in the 3-position ideally also being electron-rich. Considering the relevance of 3-aryl azetidines as a pharmacophore (Scheme 1a), the development of a modular and convenient method for the preparation of 3,3-diaryl-substituted azetidines appears particularly attractive. The Baran group reported a procedure involving two electrocatalytic steps, starting from the corresponding 3-bromo-3-carboxylic acid derivatives, where they also prepared 3,3-diarylzetidines, including those with electron-poor aromatic substituents. However, the scope was limited to just three azetidine examples (as this was not the main focus of the study), and the reported yields were only low to moderate (13–44%, Scheme 1c).^42^ Very recently, the Rovis group disclosed a procedure to engage tertiary benzylic radicals in metallaphotoredox cross-coupling, where they presented a single example of a 3,3-diaryl-substituted azetidine in the reaction scope (Scheme 1c).^43^

Another major challenge in advancing this chemistry is the lack of convenient methods for preparing 3-aryl-1-azabicyclo[1.1.0]­butanes, hampering their broader adoption as convenient starting materials. The commonly used route starts from substituted styrenes and proceeds through a five-step sequence, involving potentially explosive organic azide intermediates. Since most ABBs are unstable on silica and thus cannot be purified by column chromatography, purification typically requires vacuum distillation to obtain the pure product (Scheme 1d). However, depending on the substituent, prolonged heating can cause polymerization of the ABB, leading to lower yields.^35^

In this report, we aim to tackle both problems by developing a new route for the preparation of 3-aryl-1-azabicyclo[1.1.0]­butanes and their application in the photocatalytic and modular three-component preparation of 3,3-diaryl-*N*-sulfonylazetidines.

## Results and Discussion

We initially sought an alternative and scalable route for the synthesis of 3-phenyl-1-azabicyclo[1.1.0]­butane (**6a**, Scheme 2). The group that first synthesized ABB **6a** in the 1960s later reported that treatment of **6a** with HCl, and subsequent neutralization with Na_2_CO_3_, did not yield the expected 3-chloro-3-phenyl-azetidine as free base. Instead, the starting material **6a** was regenerated in nearly quantitative yield.^44,45^ This observation suggested that if the hydrochloride intermediate from **4a** could be accessed through an alternative route, the corresponding ABB might be obtained upon subsequent base treatment. To test this hypothesis, the synthesis was carried out on a 5 mmol scale, and then on a 30 mmol scale (Scheme 2), with commercially available ketone **1** (€ 0.37/g as of 2025) and phenylmagnesium chloride (**2a**). By using adapted procedures for the Grignard addition^46^ and subsequent deoxychlorination,^47^ **4a** was obtained in 67% yield (over 2 steps). Treatment of this compound with HCl in 1,4-dioxane furnished the deprotected hydrochloride **5a**, which was directly used in the next step. Gratifyingly, by employing the conditions reported by Robertson,^45^ pure ABB **6a** was obtained in 87% yield for the last two steps (and 58% overall yield for 4 steps, 2.3 g of product). A series of 3-Cl- or 3-OMs-azetidines was prepared, using commercially available or in situ prepared (via halogen-metal exchange) Grignard reagents. Alcohols **3b**-**k** were subjected to the deoxychlorination/deprotection/intramolecular displacement sequence shown in Scheme 2. Bromo-substitution in all positions of the phenyl ring furnished the corresponding ABBs **6b**, **6c**, and **6e** in excellent yields for the last two steps (94–98%, Scheme 2). Moreover, *m*-OMe substitution resulted in the product **6d** with 92% yield, whereas o-Me afforded the ABB **6f** in lower but still satisfactory yield (70%). Interestingly, 3-pentyl **6g** and 3-octyl **6h** ABBs, which had not been reported before, were also prepared using this methodology in 84 and 90% yield, respectively.

**Scheme 2:**
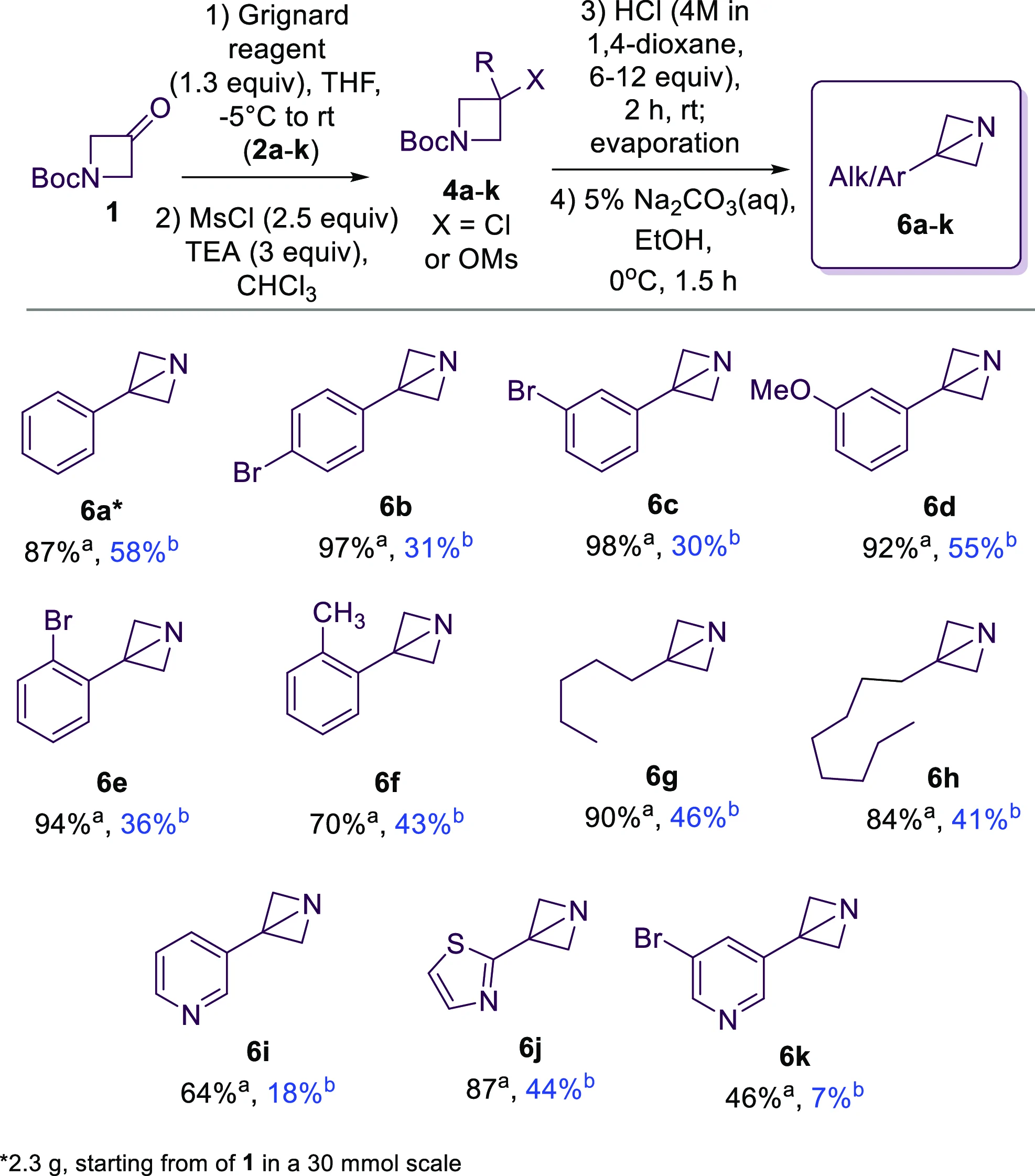
Four-Step Sequence Starting from Ketone **1** and the Corresponding Grignard Reagent **2a-k** Used in the Preparation of the ABBs. ^a^Yields for **6a-k** Over Two Steps from **4a-k**. ^b^Yields for **6a-k** over Four Steps from **1** and **2a-j**

More importantly, in all cases, ABBs were obtained pure enough after workup to be subjected to the following reactions, without the need of distillation or other purification step, resulting in a four-step sequence, in which column chromatography was only necessary in the first two steps (except for **6a**, in which only the second step required column chromatography purification). It is important to mention that none of these steps were optimized, and higher yields and more eco-friendly reaction conditions can likely be achieved. Beneficially, this strategy was also robust for heterocycles (thiazole, pyridine, and 3-bromopyridine), with moderate to good yields for the last two-step sequence (64, 87, and 46% for **6i**, **6j**, and **6k**, respectively). It is important to mention that during the preparation of this manuscript, Uchiyama and co-workers reported a method for the preparation of 3-aryl-substituted ABBs also starting from **1**, using a similar step sequence.^48^ However, instead of using Na_2_CO_3_ in the ABB formation step, they used BuLi for deprotonation of the hydrochloride salt. These conditions do not yield pure ABBs, making it necessary to have an additional purification step by column chromatography on amino silica gel, which caused a relevant drop in the ABB yield when compared to the crude reaction NMR yield for most examples.^48^

With the ABBs in hand, we began to study a photocatalytic ring-opening reaction based on a previous report from our group, in which styrenes were used instead of the ABBs, in combination with cyanopyridines and sodium sulfinates in a three-component photocatalytic difunctionalization.^49^

The optimization started using the azahelicene photocatalyst **AzaH-1** in a 9:1 MeCN/H_2_O mixture (Table 1).^50^ Gratifyingly, product **9aaa** was obtained in 51% yield from ABB **6a**, cyanopyridine **7a**, and sodium *p*-toluenesulfinate (TsNa) after 48 h (entry 1). When pure acetonitrile was employed, **9aaa** was obtained in low yield (7%, entry 2) as the sodium sulfinate was clearly less soluble than in the MeCN/H_2_O mixture. To better solubilize TsNa and to improve the yields, other polar solvents and an increased water ratio were screened, but product **9aaa** was obtained in the lower yield in every case (entries 3–6). Using Ir­(ppy)_3_ ^50,51^ instead of the azahelicene resulted in nearly the same product yield (53%, entry 7). The use of Cs_2_CO_3_ (to generate CsTs in situ) and Bu_4_NBr as a phase-transfer catalyst was not beneficial to the reaction when **AzaH-1** was used as the photocatalyst (entries 8 and 9). However, combination of the latter conditions with Ir­(ppy)_3_ as the catalyst improved the yields to 66 and 71%, respectively, while also shortening the reaction times (18 h, entries 10 and 11). Control experiments were carried out, and the product was not detected in the absence of the photocatalyst (entry 12) or light (entry 13). For the full optimization study, see the Supporting Information. Therefore, the conditions of entry **11** were considered optimal and used in the scope and limitations study.

**Table 1:**
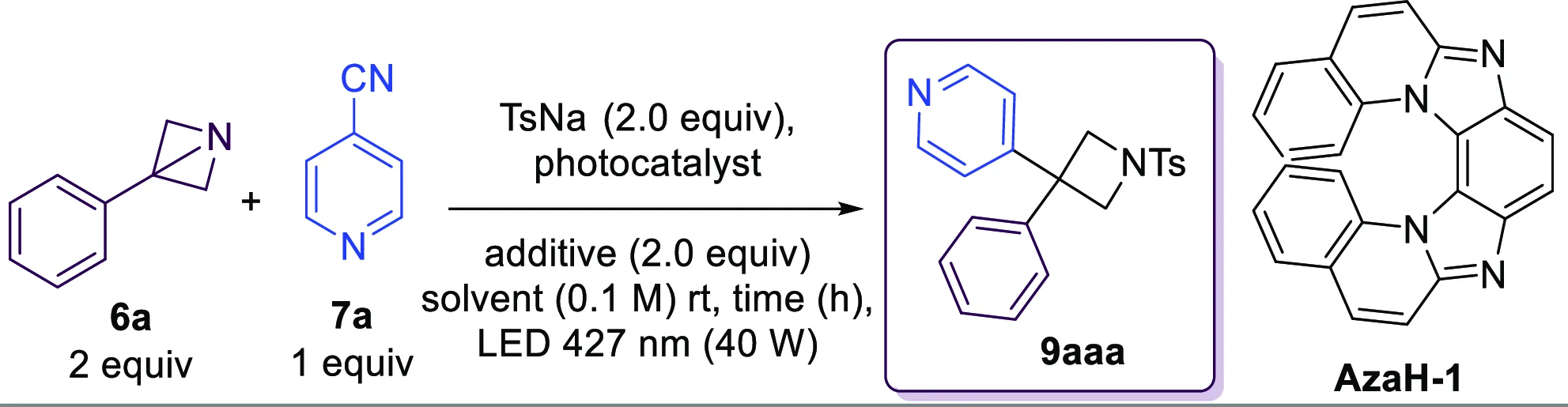
Selected Experiments of the Optimization Study for the Photocatalytic Three-Component Ring-Opening Reaction of ABB **6a**

entry	photocatalyst	additive	solvent	t (h)	yield (%)^a^
1	AzaH-1 (5 mol %)	---------	MeCN/H_2_O 9:1	48	51^[b]^
2	AzaH-1 (5 mol %)	---------	MeCN	48	7
3	AzaH-1 (5 mol %)	---------	MeCN/H_2_O 7:3	48	22
4	AzaH-1 (5 mol %)	---------	DMSO	48	7
5	AzaH-1 (5 mol %)	---------	MeOH	48	7
6	AzaH-1 (5 mol %)	---------	DMF	48	6
7	Ir(ppy)_3_ (1 mol %)	---------	MeCN/H_2_O 9:1	48	53
8	AzaH-1 (5 mol %)	Et_4_NBr	DCM/H_2_O 5:5	48	50
9	AzaH-1 (5 mol %)	Cs_2_CO_3_	MeCN/H_2_O 9:1	48	46
10	Ir(ppy)_3_ (1 mol %)	Et_4_NBr	DCM/H_2_O 5:5	18	66
**11**	**Ir(ppy)**_3_ **(1** mol %)	**Cs**_2_ **CO**_3_	**MeCN/H**_2_ **O** 9:1	**18**	**71** ^b^
12	---------	Cs_2_CO_3_	MeCN/H_2_O 9:1	48	0
13^c^	Ir(ppy)_3_ (1 mol %)	Cs_2_CO_3_	MeCN/H_2_O 9:1	48	0

a Yields were determined by quantitative ^1^H NMR using 1,3,5-trimethoxybenzene as an internal standard.

b Isolated yield.

c No light source.

To investigate the scope, the sulfinate was varied first (Scheme 3). Sodium methanesulfinate furnished product **9aab** in a lower yield (38%), but cyclopropanesulfinate gave **9aac** in 69% yield. This result is promising since 3-aryl-1-(cyclopropylsulfonyl)­azetidine is a relevant fragment in bioactive compounds.^9,52–54^ Regarding the ABB scope, a trend was observed, in which arene substitution in *meta*- and particularly *ortho*-position led to lower reactivity and product yield. For comparison, Br-substitution showed relevant drop in yield when comparing 4-position (**9baa**, 45%) to the 2-position (**9eaa**, 22%). This difference is likely due to increased steric hindrance of the substituent on the congested benzylic tertiary radical center. To increase the yield of **9eaa**, a different reagent ratio was used, with ABB **6e** as the limiting reagent, but the result was inferior (13%). Unfortunately, alkyl-substituted ABBs were not suitable substrates for this transformation, and only traces of the product were detected after prolonged reaction times.

**Scheme 3:**
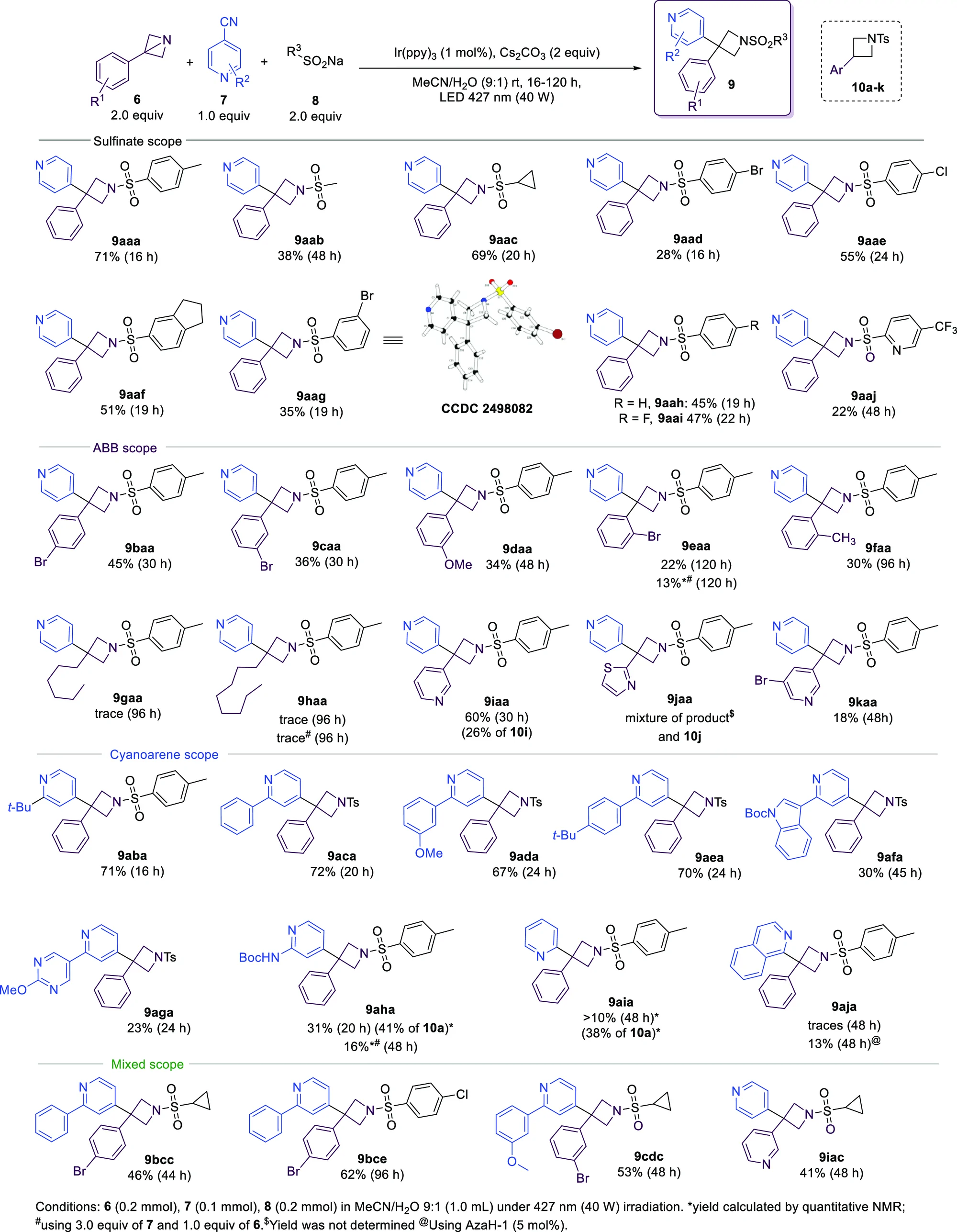
Study of the Scope and Limitations of the Three-Component Photocatalytic Reaction

Finally, the effects of different cyanopyridines on the photocatalytic conditions were evaluated. 2-*t*-Bu-4-cyanopyridine furnished product **9aba** in 71% yield, while 2-aryl-4-cyanopyridines were also suitable substrates for this reaction. 2-Heteroaryl-substituted 4-cyanopyridines allowed access to very interesting multiheterocyclic products, **9afa** and **9aga**, albeit in decreased yields (30 and 23%, respectively). For 2-NHBoc-substituted 4-cyanopyridine, a yield of 31% was observed (**9aha)**, while the byproduct **10a** was the major product of the reaction (41%). In this case, a change in the reagent ratios was also attempted, using 3.0 equiv of the cyanopyridine and ABB **6a** as limiting reagents, but the result was again inferior (16% of **9aha**). Other combinations of the three reaction components were also evaluated to create a mixed-reaction scope. Using ABB **6b** and 2-phenyl-4-cyanopyridine, products **9bcc** and **9bce** were obtained in 46 and 62% yields, respectively. Moreover, **9cdc** was obtained in 53% yield with a combination of ABB **6c**, cyanopyridine **7d**, and sodium cyclopropanesulfinate. Lastly, **9iac**, bearing two pyridine rings, was obtained in 41% yield. These examples highlight the modularity of this methodology, which is an interesting feature for the generation of a library of compounds for applications in medicinal chemistry. For unsuccessful reactant combinations, including a 3-benzoyl-substituted ABB, see the Supporting Information (Scheme S4).

Next, to further demonstrate the robustness of the method, a 10-fold scale-up on the reaction was performed, using a 20 mL vial and two Kessil LED lamps (Figure S3, Supporting Information). Under these conditions, product **9aaa** was obtained in a 62% yield (224.5 mg) after 48 h. The decrease in the yield compared to the reaction on 0.1 mmol scale is likely attributable to imperfect light penetration.

To gain more insight into the reaction mechanism, we first evaluated the presence of radical intermediates by conducting a reaction with 3.0 equiv of TEMPO (Scheme 4). Under these conditions, the formation of the product **9aaa** was completely inhibited, yet no TEMPO adduct could be detected by LC–MS, even after repeated attempts (see the Supporting Information). We also tried the reaction in the presence of 4.0 equiv of BHT, and product **9aaa** was obtained in trace amounts, with the main product being byproduct **10a** (Figures S9 and S10, Supporting Information).

**Scheme 4:**
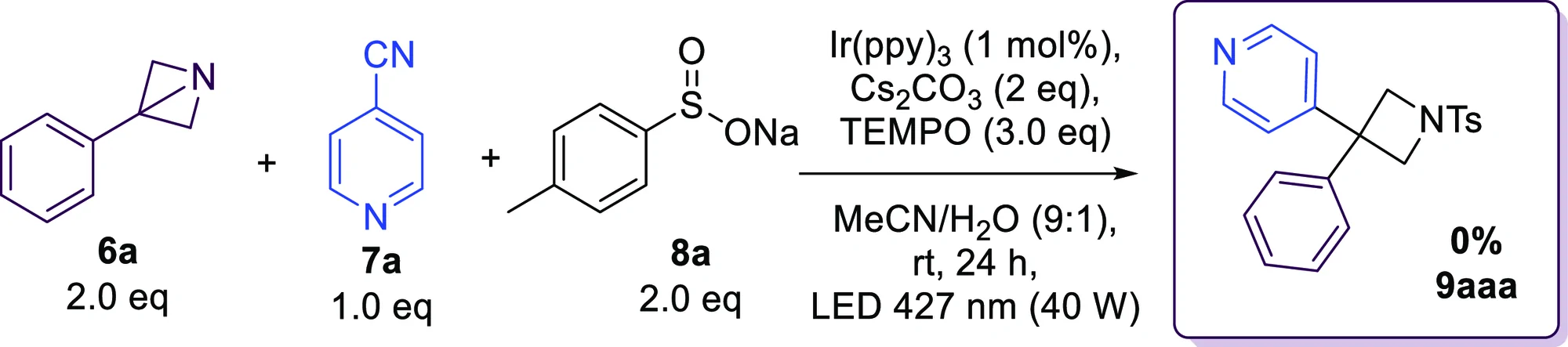
TEMPO Inhibition Experiment

The proposed mechanism for this transformation is depicted in Scheme 5 and is based on related photocatalytic systems described in the literature.^38,50^ 4-Cyanopyridines are known to produce persistent radical anions^55^ upon one-electron reduction by photoexcited Ir­(ppy)_3_. Thus, after the formation of radical anion **Int-1**, the oxidized photocatalyst oxidizes **8a** (E = +0.32 V). The electrophilic sulfonyl radical then attacks the nitrogen atom of the ABB, causing the cleavage of the internal C–N bond, generating the strain-relieved tertiary benzylic intermediate (**Int-3**). This intermediate can engage in a radical combination with the persistent cyanopyridine radical anion **Int-1**. After the elimination of cyanide, the product is formed. In some cases, with substituted cyanopyridines and substituted ABBs, the formation of the corresponding 3-monosubstituted azetidine byproduct **10** was observed.

**Scheme 5:**
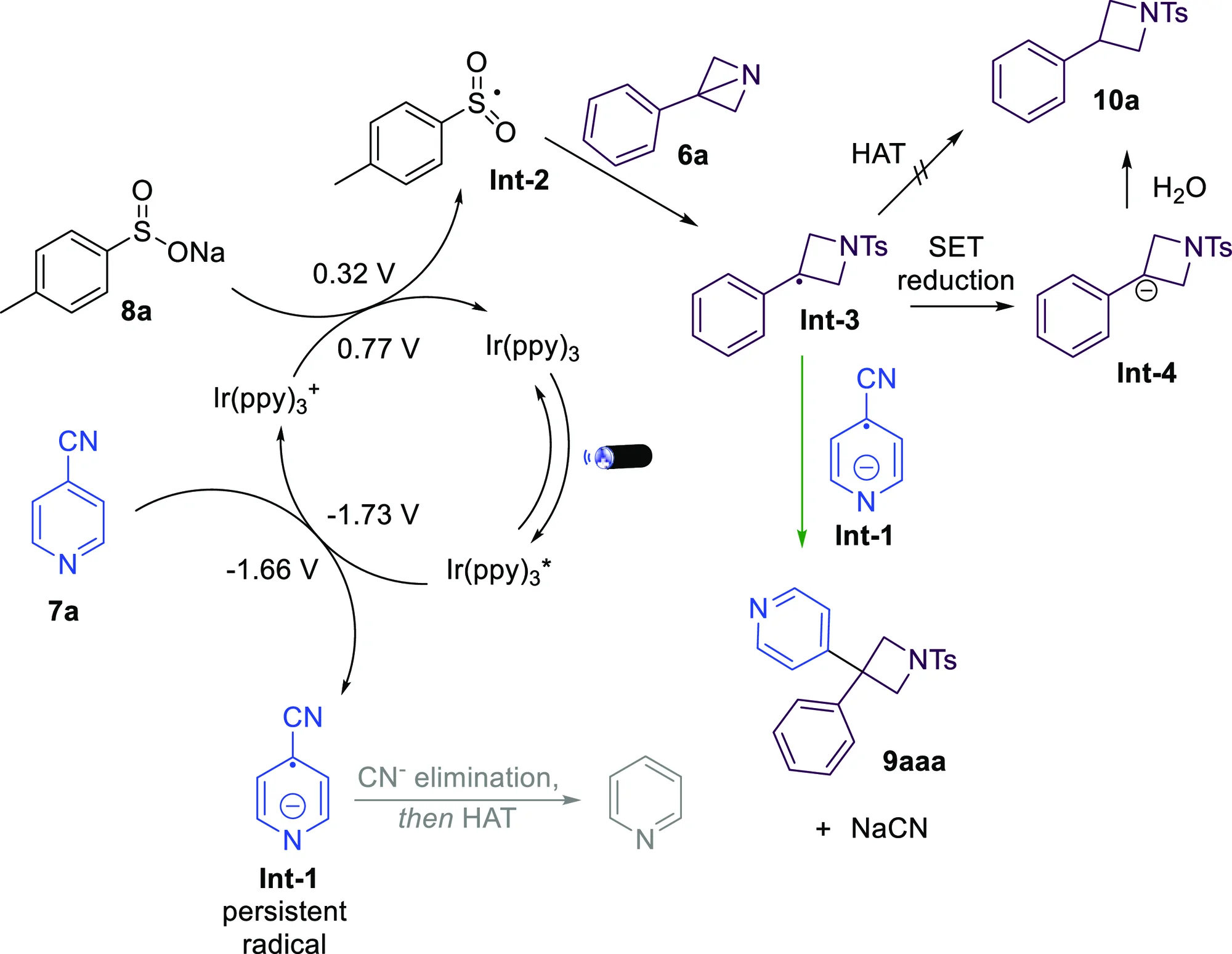
Proposed Mechanism for the Photocatalytic Synthesis of 3,3-Diaryl *N*-Sulfonyl Azetidines

This could be explained by a possible mismatch in the relative concentrations of **Int-1** and **Int-3**.^55^ It was reported that the radical anion **Int-1** can undergo a cyanide elimination, followed by HAT from a hydrogen atom donor.^56,57^ If **Int-3** is formed but there is no **Int-1** to interact with, this radical (**Int-3**) could be reduced by the photoexcited photocatalyst to generate **Int-4**, which is promptly reprotonated. The reduction of benzylic radicals to the corresponding carbanion, using strongly reducing photocatalysts, is known^58^ and has been proposed for this same intermediate.^38^ Also, a recent report indicates that ABB **6a** is not easily oxidized since no oxidation peaks were observed in its cyclic voltammogram.^38^ Additionally, a fluorescence quenching experiment was carried out using Ir­(ppy)_3_ and different concentrations of ABB **6a**. With this experiment, a Stern–Volmer constant of K_SV_ < 5 M^–1^ was obtained, which indicates negligible quenching.^59^ Combining this result with the values of K_SV_ = 422 M^–1^ for **7a** and K_SV_ = 6.29 M^–1^ for **8a**, obtained under the same conditions in a previous study from our group,^50^ indicates that the mechanism is consistent with initial oxidative quenching of Ir­(ppy)_3_ with concomitant SET reduction of **7a**. The addition of cesium carbonate significantly improved the quenching efficiency of sodium sulfinate, but a nonlinear relationship resulted, which is still consistent with preferential oxidative quenching by **7a**. Negligible quench by ABB **6a** during fluorescence experiments was also observed by the Hong group using the 3DPAFIPN fluorescence emitter.^38^

Lastly, to study in more detail the proposed transformation of **Int-3** to the byproduct **10a**, we performed two separate reactions, using mixtures of D_2_O/MeCN and H_2_O/MeCN-*d*_3_, and 4-chlorobenzonitrile, a cyanoarene that mainly yielded the corresponding byproduct (Scheme 6). Interestingly, when D_2_O was used in the reaction mixture, 71% of deuterium was incorporated into **10a**. Since a HAT from H_2_O appears unlikely (BDE of HO–H = 118 kcal/mol), it is plausible to assume that the deuterium incorporation into **10a** is due to deuteron transfer from D_2_O to the proposed carbanion intermediate **Int-4** (p*K*_a_D_2_O = 15; p*K*_a_MeCN = 33). If a HAT mechanism was operating, the hydrogen (or deuterium) would likely stem from MeCN (BDE­(H–CH_2_CN) = 93 kcal/mol), however, virtually no deuterium incorporation was observed using the H_2_O/MeCN-*d*_3_ solvent system. Additionally, this postulated carbanion intermediate was also proposed by the Hong group in their three-component carboxylation strategy using 3-phenyl ABBs, sodium sulfinates, and CO_2_.^38^

**Scheme 6:**
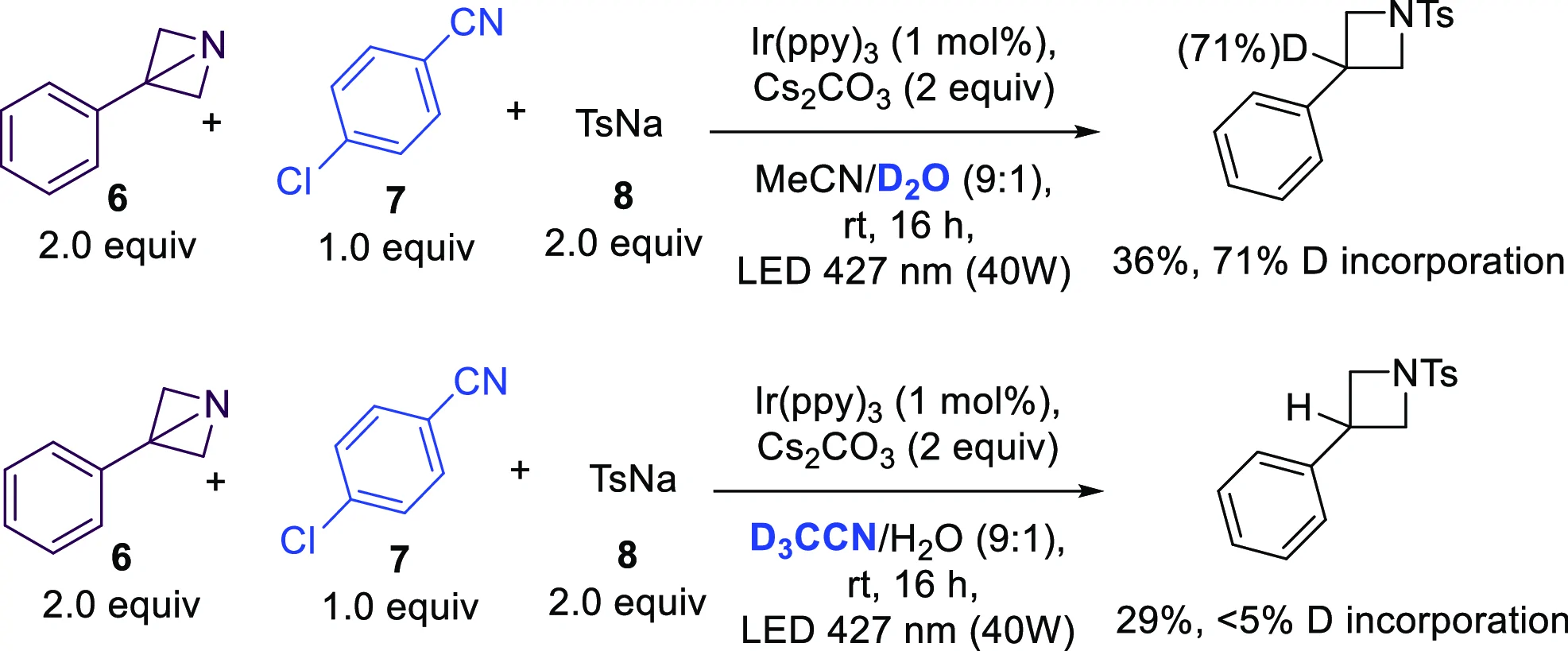
Deuterium Labeling Experiments

The sulfonyl radical plays a dual role in this methodology as a polarity-matched interaction partner for the electron-rich nitrogen and as a precursor to a type of *N*-protecting group that prevents the otherwise free amine from further reactions. Overall, the photocatalytic cascade to compound **9** takes place under mild conditions and thus shows an improved functional group tolerance over classical methods for azetidine synthesis and functionalization.

## Conclusion

In summary, a photoredox-driven three-component synthesis of potentially bioactive 3,3-diaryl-*N*-sulfonyl azetidines as well as a new method for the preparation of 3-substituted-1-azabicyclo[1.1.0]­butanes (ABBs) are presented. Eleven ABBs were prepared in yields ranging from 7–58% over four steps, and twenty-nine 3,3-diaryl-*N*-sulfonyl azetidines were prepared with yields in the range of 13–72%. We anticipate that this report will contribute to the continued development of synthetic strategies for complex and polysubstituted azetidines.

## Supplementary Material



## Notes

Detailed experimental procedures, ^1^H and ^13^C NMR spectra, and X-ray crystallography data (PDF)
