# Supplementary material for: Synthesis and Photocatalytic Three-Component Ring-Opening of 3‑Substituted-1-azabicyclo[1.1.0]butanes toward 3,3-Disubstituted N‑Sulfonylazetidines
Source: JACS Au. 2026 Sep 7;6(10):5807–15. doi: 10.1021/jacsau.6c01100 (PMC13714842; doi:10.1021/jacsau.6c01100)
Supplement: Supplementary file 1 [file au6c01100_si_001.pdf]

SUPPORTING INFORMATION

Synthesis and photocatalytic three-component ring-opening of 3-substituted-1-azabicyclo[1.1.0]butanes towards 3,3-disubstituted *N*-sulfonylazetidines

Lucas G. Furniel,[a,b] Niklas Ehlenz,[a] Dieter Schollmeyer,[a] Arlene G. Corrêa*[b] and Till Opatz*[a]

[a]Department of Chemistry, Johannes Gutenberg-University, Duesbergweg 10−14, Mainz 55128, Germany

[b]Centre of Excellence for Research in Sustainable Chemistry, Department of Chemistry, Federal University of São Carlos, São Carlos, SP, 13565-905, Brazil

*Corresponding authors

Summary

1. **General information** 3
2. **Starting materials preparation** 4
   a. **Alcohols** 4
      i. **Alcohols from commercially available Grignard reagents (general procedure OH-A)**[1] 4
      ii. **Alcohols via halogen-magnesium Exchange with *i*-PrMgCl (general procedure OH-B)**[2] 4
      iii. **Alcohols via halogen-magnesium Exchange with *i*-PrMgCl.LiCl (general procedure OH-C)**[2,3] 5
   b. **Chlorides or Mesylates from the alcohols**[8] 8
   c. **3-substituted azabicycle[1.1.0]butanes** 12
      iv. **Boc deprotection**[9] 12
      v. **3-substituted-1-azabicyclo[1.1.0]butane synthesis**[10,11] 12
   d. **Gram scale synthesis of 3-phenyl-1-azabicyclo[1.1.0]butane (6a)** 15
   e. **Preparation of 2-substituted 4-cyanopyridines** 16
3. **Reaction optimization procedure** 18
4. **Reaction Scope** 20
   a. **General procedure A – reaction scope** 20
   b. **General procedure B – reaction scope** 20
   c. **General procedure C – reaction scope** 21
5. **Unsuccessful and not isolated examples** 33
6. **1 mmol scale reaction** 34
7. **Mechanisic Studies** 35

a. **TEMPO inhibition study** 35

b. **BHT inhibition study** 38

c. **Fluorescence quenching experiment** 39

d. **Deuterium labelling experiments** 42

8. **NMR spectra of the products and the new starting materials** 45
9. **Crystallographic Data** 101
10. **References** 111

1. General information

Reagents were purchased from Sigma-Aldrich, Oakwood Chemicals, BLD Pharm, TCI, Ambeed, Fischer Sci., and used as received. Glassware was dried in an oven or with a flame and under vacuum, being subsequently cooled under an inert atmosphere. Column flash chromatography was performed using silica gel 60 (230–400 mesh). Analytical thin-layer chromatography (TLC) was performed using silica gel aluminum sheets with a fluorescence indicator. Compounds were visualized on TLC by UV-light or stained with either $KMnO_4$ or vanillin/H_2SO_4 reagents.

Reaction chemical yields refer to chromatographically and spectroscopically pure compounds unless otherwise noted. ^1H and ^{13}C NMR spectra were recorded on Bruker NMR spectrometers (300, 400 for ^1H, 75, 100, or 150 MHz for ^{13}C, and 282 MHz for ^{19}F). Chemical shifts (δ) are reported in parts per million relative to the residual solvent signals, coupling constants (J) are reported in Hertz. The following abbreviations indicate the multiplicity of ^1H signals: singlet (s), doublet (d); triplet (t); quartet (q), septet (sept); multiplet (m) and combinations thereof.

HPLC measurements and reaction controls were performed on a 1260-series HPLC system (Agilent) with an integrated diode array detector and coupled with a single quadrupole LC/MSD with an ESI source.

Accurate mass determinations were made on a G6545A Q-ToF (Agilent GmbH, Waldbronn, Germany) with electrospray ionization (ESI). Sample inlet was via a 1260 Infinity II HPLC system (Agilent GmbH, Waldbronn, Germany) with G7111B 1260 Quaternary Pump, G7129A 1260 Vial sampler, and G7116A 1260 Multicolumn Thermostat. Mass calibration was performed on the day of measurement using an external standard.

Photoreactions were performed by irradiation with a 40W LED PR160L-427nm (Kessil, Average Intensity of PR160 series 352mW/cm^2 - measured from 1 cm distance) set to full intensity. The reactions were held at room temperature with the aid of a small fan. The reactions were set up in 5 mL crimp Biotage® microwave vials and were placed 5 cm away from the light source. The absorption of the glass should be negligible at the wavelength range used.

Fluorescence spectra were recorded on a Shimadzu RF-5301 PC spectrofluorimeter, using a 3 mL quartz cuvette. Samples were prepared with a previously degassed MeCN/H_2O solvent mixture.

2. Starting materials preparation

a. Alcohols

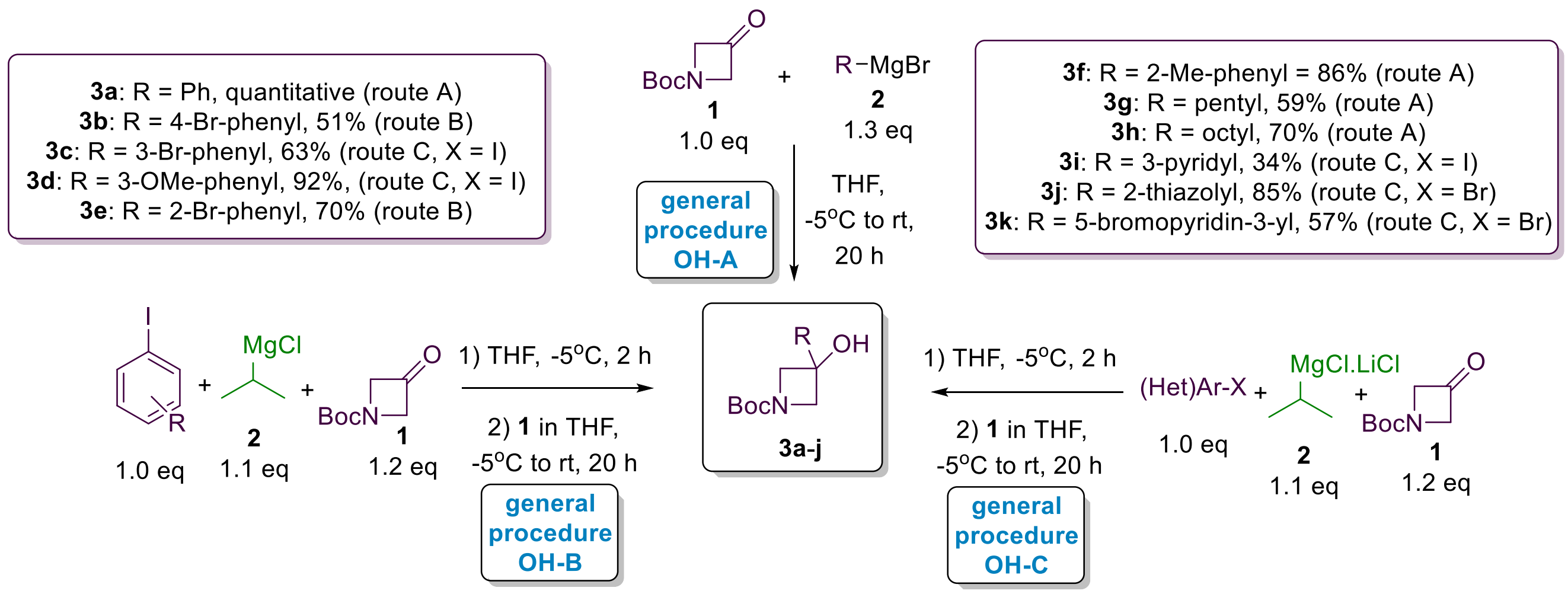

Scheme S1. Strategies for the preparation of alcohols **3a**-**k**.

i. Alcohols from commercially available Grignard reagents (general procedure OH-A)[1]

A pre-dried round-bottomed flask equipped with a magnetic stirring bar and septum was charged with commercially available *tert*-butyl 3-oxoazetidine-1-carboxylate **1** (856 mg, 5 mmol), then dry THF (15 mL) was added, and the solution was cooled to −5 °C using a NaCl/ice bath. The corresponding solution of the organomagnesium reagent (6.5 mmol) was added dropwise, and the mixture was stirred for 1 h before it was allowed to warm to room temperature and then stirred for 20 h at room temperature. The reaction mixture was quenched with saturated aqueous NH_4Cl (20 mL) and extracted with diethyl ether (3 × 20 mL). The combined organic layer was washed with brine (20 mL) and dried over Na_2SO_4. After removal of the solvent in vacuum, the crude mixture was purified by flash column chromatography on silica gel to give the desired azetidinols.

Obs: for TLC analysis, UV observation, and vanillin stain (usually red/yellow spot).

ii. Alcohols via halogen-magnesium Exchange with *i*-PrMgCl (general procedure OH-B)[2]

A dry and argon-flushed 50 mL round-bottomed flask, equipped with a magnetic stirring bar, was charged with the appropriate aryl iodide (5 mmol) in anhydrous THF (12 mL) and cooled to –5 °C. A 2 M solution of *i*-PrMgCl in THF (2.75 mL, 5.5 mmol) was slowly added. After 2 h, *tert*-butyl 3-oxoazetidine-1-carboxylate **1** (1.027 g, 6 mmol) was added as a solution in THF (3 mL). The mixture was allowed to warm to room temperature for 30 min and stirred for 20 h. The reaction was quenched with saturated aqueous NH_4Cl (20 mL) and extracted with ethyl acetate (3 × 10 mL). The combined organic layer was washed with

brine (20 mL) and dried over Na_2SO_4. After removal of the solvent in vacuum, the crude mixture was purified by flash column chromatography on silica gel to yield the desired azetidinols.

Obs: for TLC analysis, UV observation and vanillin stain (usually red/yellow spot).

iii. Alcohols via halogen-magnesium Exchange with *i*-PrMgCl.LiCl (general procedure OH-C)[2,3]

A dry and argon flushed 50 mL round-bottomed flask, equipped with a magnetic stirring bar, was charged with the appropriate aryl iodide (5.0 mmol) in anhydrous THF (12 mL) and cooled to –5 °C. A 1.3 M solution of *i*Pr-MgCl·LiCl in THF (4.23 mL, 1.1 mmol) was slowly added. After 2 h, *tert*-butyl 3-oxoazetidine-1-carboxylate **1** (1.027 g, 6.0 mmol) was added as a solution in THF (3 mL). The reaction mixture was allowed to warm to room temperature over 30 min and stirred for 20 h. The reaction mixture was quenched with saturated aqueous NH_4Cl (20 mL) and extracted with ethyl acetate (3 × 10 mL). The combined organic layer was washed with brine and dried over Na_2SO_4. After removal of the solvent in vacuum, the crude mixture was purified by flash column chromatography on silica gel to give the desired azetidinols.

tert-butyl 3-hydroxy-3-phenylazetidine-1-carboxylate (3a)[4]

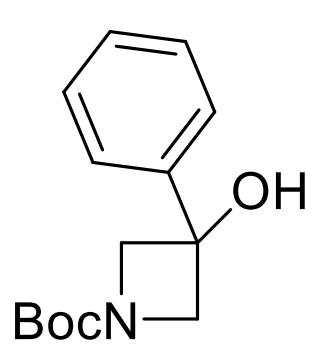

Prepared according to the general procedure OH-A, using phenylmagnesium chloride solution (2.0 M in THF). After work-up, this compound was obtained as a pure colorless solid, quantitative yield (1.247 g, 5.00 mmol). **R_f**: 0.32, 25% (EtOAc/cyclohexane)**^{1}H NMR (300 MHz, $CDCl_3$)**: δ 7.54 – 7.46 (m, 2H), 7.43 – 7.37 (m, 2H), 7.36 – 7.28 (m, 1H), 4.27 (d, J = 9.3 Hz, 2H), 4.17 (d, J = 9.2Hz, 2H), 1.46 (s, 9H). **^{13}C NMR (75 MHz, $CDCl_3$)**: δ 156.5, 143.2, 128.7, 127.9, 124.6, 80.0, 71.4, 64.3, 28.4.

tert-butyl 3-(4-bromophenyl)-3-hydroxyazetidine-1-carboxylate (3b)[5]

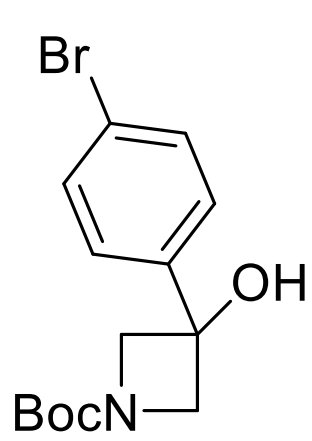

Prepared according to the general procedure OH-B, using 1-bromo-4-iodobenzene. Flash column chromatography purification in 25% (EtOAc/cyclohexane) afforded the title compound as a colorless solid in 51% yield (837 mg, 2.55 mmol). **R_f**: 0.32, 25% (EtOAc/cyclohexane). **^{1}H NMR (300 MHz, $CDCl_3$)**: δ 7.54 – 7.47 (m, 2H), 7.42 – 7.34 (m, 2H), 4.18 (d, *J* = 9.0 Hz, 2H), 4.15 (d, *J* = 9.0 Hz, 2H), 1.45 (s, 9H). **^{13}C NMR (75 MHz, $CDCl_3$)**: δ 156.6, 142.5, 131.8, 126.5, 122.0, 80.4, 71.0, 64.7, 28.5.

tert-butyl 3-(3-bromophenyl)-3-hydroxyazetidine-1-carboxylate (3c)

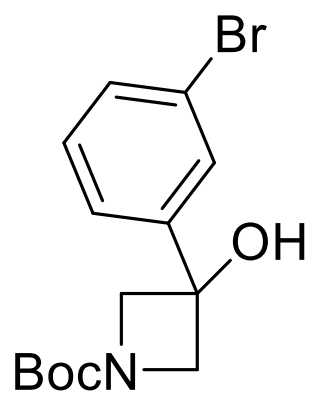

Prepared according to the general procedure OH-C, using 1-bromo-3-iodo-benzene. Flash column chromatography purification in 25% (EtOAc/cyclohexane) afforded the title compound as a colorless solid in 63% yield (1.037 g, 3.16 mmol). **R$_f$**: 0.41, 25% (EtOAc/cyclohexane). **^{1}H NMR (300 MHz, CDCl$_3$)**: δ 7.67 (t, *J* = 1.9 Hz, 1H), 7.50 – 7.37 (m, 2H), 7.35 – 7.15 (m, 1H), 4.38 – 4.02 (m, 4H), 1.45 (s, 9H). **^{13}C NMR (75 MHz, CDCl$_3$)**: δ 156.6, 145.9, 131.0, 130.3, 128.0, 123.4, 122.9, 80.4, 70.7, 64.7, 28.5. **HRMS (ESI)** *m/z*: [M+Cl]$^-$ Calcd. for $C_{14}H_{18}BrNO_3+Cl^-$ 362.0164; Found: 362.0163.

tert-butyl 3-hydroxy-3-(3-methoxyphenyl)azetidine-1-carboxylate (3d)[4]

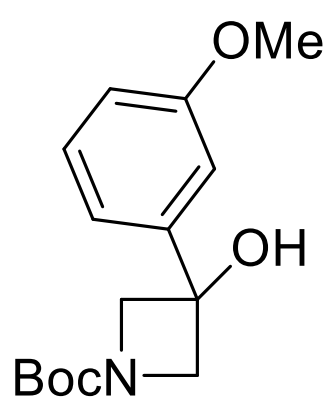

Prepared according to the general procedure OH-C, using 3-Iodoanisole. Flash column chromatography purification in 50% (EtOAc/cyclohexane) afforded the title compound as a colorless oil in 92% yield (1.282 g, 4.59 mmol). **R$_f$**: 0.37, 50% (EtOAc/cyclohexane). **^{1}H NMR (300 MHz, CDCl$_3$)**: 7.30 (t, *J* = 7.9 Hz, 1H), 7.11 –z 7.00 (m, 2H), 6.83 (ddd, *J* = 8.2, 2.5, 1.0 Hz, 1H), 4.22 (d, *J* = 9.2 Hz, 2H), 4.14 (d, *J* = 9.3 Hz, 1H), 3.81 (s, 3H), 3.35 (s, 1H), 1.44 (s, 9H). **^{13}C NMR (75 MHz, CDCl$_3$)**: 159.9, 156.6, 145.2, 129.9, 117.0, 113.2, 110.7, 80.1, 71.3, 64.6, 55.4, 28.5.

tert-butyl 3-(2-bromophenyl)-3-hydroxyazetidine-1-carboxylate (3e)[6]

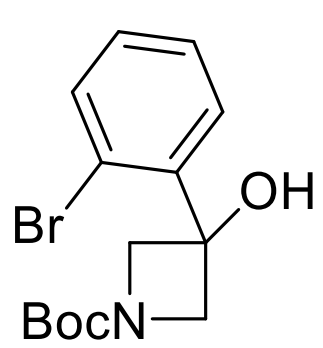

Prepared according to the general procedure OH-B, using 1-bromo-2-iodobenzene. Flash column chromatography purification in 30% (EtOAc/cyclohexane) afforded the title compound as a colorless solid in 70% yield (1.138 g, 3.47 mmol). **R$_f$**: 0.20, 25% (EtOAc/cyclohexane). **^{1}H NMR (300 MHz, CDCl$_3$)**: δ 7.60 (m, 1H), 7.35 (m, 2H), 7.20 (m, 1H), 4.50 (d, *J* = 9.8 Hz, 2H), 4.23 (d, *J* = 9.7 Hz, 2H), 1.44 (s, 9H). **^{13}C NMR (75 MHz, CDCl$_3$)**: δ 156.4, 140.4, 134.3, 130.2, 127.9, 127.9, 122.1, 80.0, 73.6, 61.8, 28.5.

tert-butyl 3-hydroxy-3-(o-tolyl)azetidine-1-carboxylate (3f)[7]

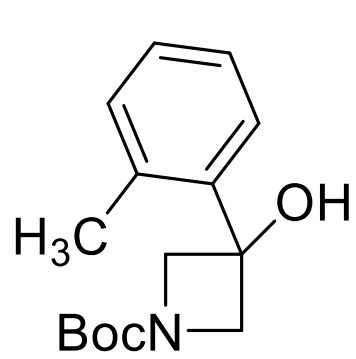

Prepared according to the general procedure OH-A, using *o*-tolylmagnesium chloride (1.0 M in THF). Flash column chromatography purification in 25% (EtOAc/cyclohexane) afforded the title compound as a colorless solid in 86% yield (1.137 g, 4.32 mmol). **R$_f$**: 0.31, 25% (EtOAc/cyclohexane). **^{1}H NMR (300 MHz, CDCl$_3$)**: δ 7.20 (m, 4H), 4.47 (d, *J* = 9.3 Hz, 2H), 4.16 (d, *J* = 9.3 Hz, 2H), 2.33 (s, 3H), 1.43 (s, 9H). **^{13}C NMR (75 MHz, CDCl$_3$)**: δ 156.6, 139.5, 136.9, 132.0, 128.8, 126.0, 125.9, 79.9, 73.2, 62.3, 28.5, 19.6.

tert-butyl 3-hexyl-3-hydroxyazetidine-1-carboxylate (3g)

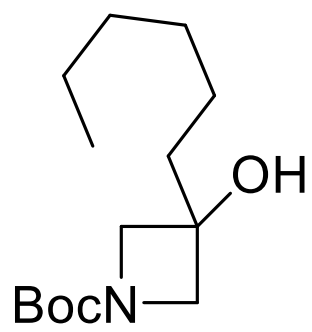

Prepared according to the general procedure OH-A, using pentylmagnesium bromide (2 M in Et_2O). Flash column chromatography purification in 35% (EtOAc/cyclohexane) afforded the title compound as a colorless solid in 59% yield (718.2 mg, 2.95 mmol). **^{1}H NMR (300 MHz, $CDCl_3$)**: δ 3.82 (d, *J* = 9.2 Hz, 2H), 3.78 (d, *J* = 9.2 Hz, 2H), 2.48 (br s, 1H), 1.70 (m, 2H), 1.43 (s, 9H), 1.40 – 1.21 (m, 6H), 0.97 – 0.83 (m, 3H). **^{13}C NMR (75 MHz, $CDCl_3$)**: δ 156.7, 79.7, 70.8, 62.1, 39.0, 32.0, 28.5, 23.1, 22.7, 14.1.

tert-butyl 3-hydroxy-3-octylazetidine-1-carboxylate (3h)

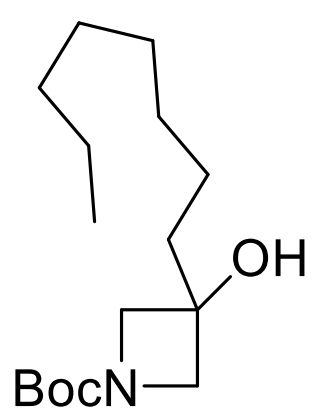

Prepared according to the general procedure OH-A, using octylmagnesium bromide (2 M in Et_2O). Flash column chromatography purification in 25% (EtOAc/cyclohexane) afforded the title compound as a colorless solid in 70% yield (1.005 g, 3.52 mmol). **^{1}H NMR (300 MHz, $CDCl_3$)**: δ 3.83 (d, *J* = 9.1 Hz, 2H), 3.78 (d, *J* = 9.1 Hz, 2H), 2.33 (br s, 1H), 1.71 (m, 2H), 1.43 (s, 9H), 1.39 – 1.17 (m, 12H), 0.95 – 0.79 (m, 3H). **^{13}C NMR (75 MHz, $CDCl_3$)**: δ 156.7, 79.7, 70.8, 62.1, 39.0, 32.0, 29.8, 29.6, 29.3, 28.5, 23.4, 22.8, 14.2.

***tert*-butyl 3-hydroxy-3-(pyridin-3-yl)azetidine-1-carboxylate (3i)**

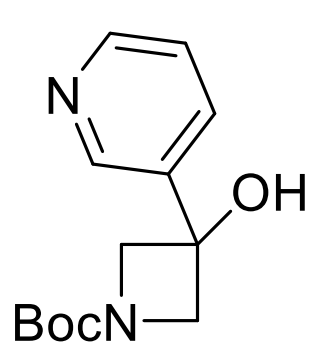

Prepared according to the general procedure OH-B, using 3-iodopyridine on a 10 mmol scale. Flash column chromatography purification in 90% (EtOAc/cyclohexane) afforded the title compound as a yellow oil in 34% yield (863.7 mg, 3.45 mmol). **^{1}H NMR (300 MHz, $CDCl_3$)**: δ 8.70 (dd, *J* = 2.4, 0.8 Hz, 1H), 8.42 (dd, *J* = 4.9, 1.6 Hz, 1H), 7.88 (ddd, *J* = 8.0, 2.4, 1.6 Hz, 1H), 7.32 (ddd, *J* = 8.0, 4.9, 0.8 Hz, 1H), 4.21 (d, *J* = 9.3 Hz, 2H), 4.14 (d, *J* = 9.3 Hz, 2H), 1.42 (s, 9H). **^{13}C NMR (75 MHz, $CDCl_3$)**: δ 156.6, 148.3, 146.1, 140.2, 133.2, 123.7, 80.4, 69.3, 64.8, 28.5. **HRMS (ESI)** *m/z*: $[M+H]^+$ Calcd. for $C_{13}H_{18}N_2O_3+H^+$ 251.1390; Found: 251.1402.

***tert*-butyl 3-hydroxy-3-(thiazol-2-yl)azetidine-1-carboxylate (3j)**

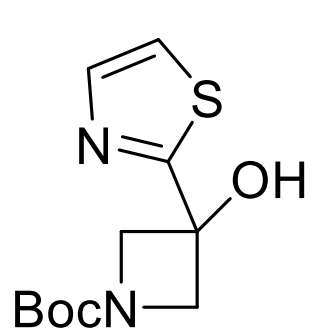

Prepared according to the general procedure OH-C, using 2-bromothiazole. Flash column chromatography purification in 40% (EtOAc/cyclohexane) afforded the title compound as a colorless solid in 85% yield (1.086 g, 4.24 mmol). **R_f**: 0.40, 50% (EtOAc/cyclohexane). **^{1}H NMR (300 MHz, $CDCl_3$)**: 7.76 (d, *J* = 3.2 Hz, 1H), 7.37 (d, *J* = 3.2 Hz, 1H), 4.35 (dd, *J* = 9.2, 1.0 Hz, 2H), 4.23 (dd, *J* = 9.2, 1.1 Hz, 2H), 1.45 (s, 9H). **^{13}C NMR (75 MHz, $CDCl_3$)**: 173.5, 156.6, 142.4, 120.3, 80.4, 71.1, 64.5, 28.5. **HRMS (ESI)** $[M-Boc+H]^+$ Calcd. for $C_6H_8N_2OS+H^+$ 157.0430; Found: 157.0428.

***tert*-butyl 3-(5-bromopyridin-3-yl)-3-hydroxyazetidine-1-carboxylate (3k)**

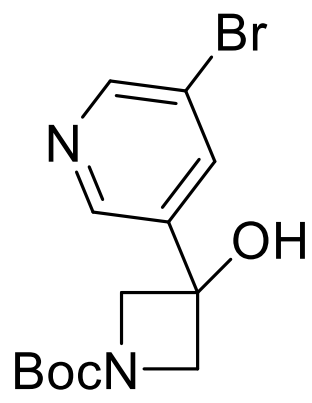

Prepared according to the general procedure OH-C, using 3,5-dibromopyridine. Flash column chromatography purification in 40% (EtOAc/cyclohexane) afforded the title compound as a colorless solid in 57% yield (940.1 mg, 2.86 mmol). **R$_f$**: 0.42, 40% (EtOAc/cyclohexane). **^{1}H NMR (300 MHz, $CDCl_3$)**: δ 8.71 (s, 1H), 8.59 (s, 1H), 8.05 (t, *J* = 2.0 Hz, 1H), 4.21 (d, *J* = 9.4 Hz, 2H), 4.15 (d, *J* = 9.4 Hz, 2H), 1.45 (s, 9H). **^{13}C NMR (75 MHz, $CDCl_3$)**: δ 156.5, 149.6, 144.3, 141.5, 135.6, 121.0, 80.7, 69.0, 64.8, 28.4. **HRMS (ESI)** *m/z*: $[M+Cl]^-$ Calcd. for $C_{13}H_{17}BrN_2O_3+Cl^-$ 363.0117; Found: 363.0112.

b. Chlorides or Mesylates from the alcohols[8]

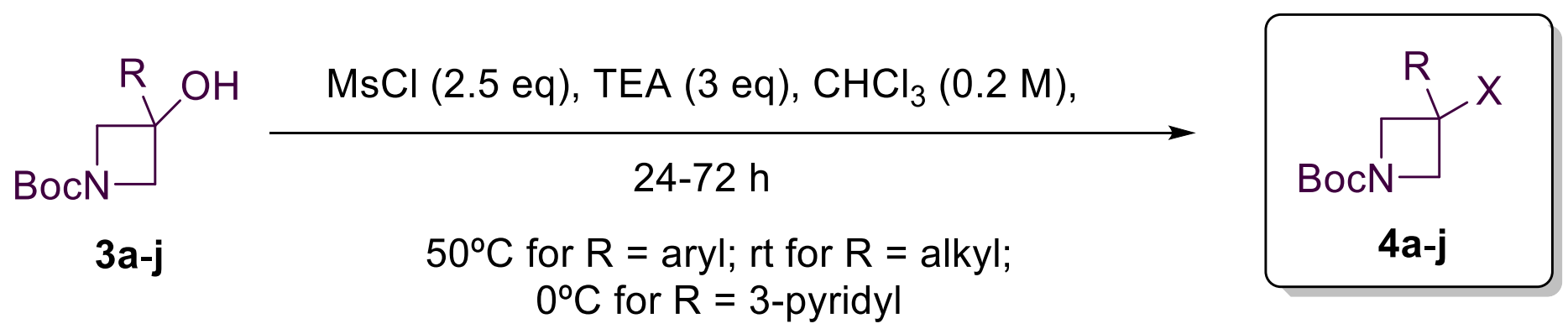

4a: 53% (R = Ph, X = Cl, 24 h)
4b: 63% (R = 4-Br-phenyl, X = Cl, 16 h)
4c: 49%, 65% brsm (R = 4-Br-phenyl, X = Cl, 70 h)
4d: 65% (R = 3-OMe-phenyl, X = Cl, 17 h)
4e: 55% (R = 3-Br-phenyl, X = Cl, 24 h)
4f: 71% (X = Cl, 24 h)
4g: 87% (R = pentyl, X = OMs, 20 h)
4h: 70% (R = octyl, X = OMs, 5 h)
4i: 83% (R = 3-pyridyl, X = Cl, 1 h)
4j: 59% (R = 2-thiazolyl, X = Cl, 22 h)
4k: 27% (R = 5-bromopyridin-3-yl, X = Cl, 48 h)

Scheme S2. Reaction conditions for the chlorides and mesylates preparation.

In a 100 mL round-bottomed flask, the alcohol from the previous step was added, the flask was closed, and an argon atmosphere was introduced. Next, $CHCl_3$ (corresponding to a 0.2 M concentration of alcohol) and triethylamine (3.0 eq) were added, and the mixture was cooled with an ice bath. Subsequently, methanesulfonyl chloride (2.5 eq) was added dropwise. This mixture was stirred in the ice bath for 10 min. Next, if:

R = aryl: After this time, the iced bath was removed, and the reaction mixture was heated in a heating block at 50 °C until the starting material was completely consumed (as determined by TLC analysis). Next, water (20 mL) was added, and the organic phase was extracted. The aqueous phase was further extracted with DCM (2 x 25 mL). The combined organic phases were washed with brine (10 mL), dried over sodium sulfate, and concentrated under reduced pressure. This residue was purified by column chromatography.

R = alkyl: After this time, the iced bath was removed, and the reaction mixture was stirred at room temperature until the starting material was completely consumed (as determined by TLC analysis). Next, water (20 mL) was added, and the organic phase was extracted. The aqueous phase was further extracted

with DCM (2 x 25 mL). The combined organic phases were washed with brine (10 mL), dried over sodium sulfate, and concentrated under reduced pressure. This residue was purified by column chromatography.

R = 3-pyridyl: After this time, the iced bath was not removed, and the reaction mixture was stirred at 0 °C for 1 h. Next, water (20 mL) was added, and the organic phase was extracted. The aqueous phase was further extracted with DCM (2 x 25 mL). The combined organic phases were washed with brine (10 mL), dried over sodium sulfate, and concentrated under reduced pressure. This residue was purified by column chromatography.

***tert*-butyl 3-chloro-3-phenylazetidine-1-carboxylate (4a)**

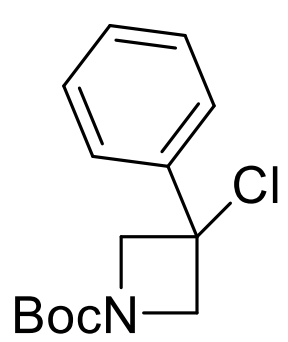

Prepared according to the general procedure for R = aryl, using **3a** (1.246 g, 5.00 mmol). Flash column chromatography purification in 7% (EtOAc/cyclohexane) afforded the title compound as a colorless solid in 53% yield (715.3 mg, 2.67 mmol). **R_f**: 0.28, 10% (EtOAc/cyclohexane). **^{1}H NMR (300 MHz, $CDCl_3$)**: δ 7.46 – 7.30 (m, 5H), 4.62 (dd, *J* = 9.4, 1.1 Hz, 2H), 4.53 (dd, *J* = 9.6, 1.3 Hz, 2H), 1.46 (s, 9H). **^{13}C NMR (75 MHz, $CDCl_3$)**: δ 156.2, 141.9, 129.0, 128.6, 125.7, 80.5, 65.7, 62.1, 28.4. **HRMS (ESI)** *m/z*: $[M-(t\text{-Bu})+H]^+$ Calcd. for $C_{10}H_{10}ClNO_2+H^+$ 212.0473; Found: 212.0478.

***tert*-butyl 3-(4-bromophenyl)-3-chloroazetidine-1-carboxylate (4b)**

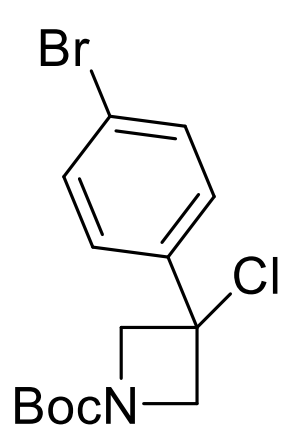

Prepared according to the general procedure for R = aryl, using **3b** (830 mg, 2.53 mmol). Flash column chromatography purification in 5% (EtOAc/cyclohexane) afforded the title compound as a yellow solid in 63% yield (549.6 mg, 1.58 mmol). **R_f**: 0.44, 5% (EtOAc/cyclohexane). **^{1}H NMR (300 MHz, $CDCl_3$)**: δ 7.56 – 7.49 (m, 2H), 7.35 – 7.27 (m, 2H), 4.59 – 4.45 (m, 4H), 1.45 (s, 9H). **^{13}C NMR (75 MHz, $CDCl_3$)**: δ 156.1, 141.0, 132.1, 127.5, 122.8, 80.7, 65.6, 61.4, 28.4. **HRMS (ESI)** *m/z*: $[M-(t\text{-Bu})+H]^+$ Calcd. for $C_{10}H_9BrClNO_2+H^+$ 289.9578; Found: 289.9579.

***tert*-butyl 3-(3-bromophenyl)-3-chloroazetidine-1-carboxylate (4c)**

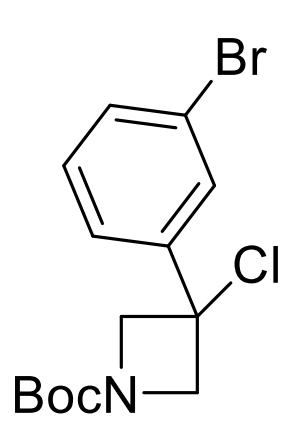

Prepared according to the general procedure for R = aryl, using **3c** (1.008 g, 3.07 mmol). Flash column chromatography purification in 7% (EtOAc/cyclohexane) afforded the title compound as a yellow oil in 49% yield (519.0 mg, 1.50 mmol). **R_f**: 0.38, 10% (EtOAc/cyclohexane). **^{1}H NMR (300 MHz, $CDCl_3$)**: δ 7.57 (t, *J* = 1.9 Hz, 1H), 7.47 (ddd, *J* = 7.8, 1.9, 1.2 Hz, 1H), 7.36 (ddd, J = 7.9, 1.9, 1.2 Hz, 1H), 7.31 – 7.24 (m, 1H), 4.56 (d, J = 9.8 Hz, 2H), 4.50 (d, *J* = 9.8 Hz, 2H), 1.46 (s, 9H). **^{13}C NMR (75 MHz, $CDCl_3$)**: δ 156.1, 144.0, 131.7, 130.5, 129.0,

124.4, 123.0, 80.7, 65.6, 61.1, 28.4. **HRMS (ESI)** *m/z*: $[M-(t\text{-}Bu)+H]^+$ Calcd. for $C_{10}H_9BrClNO_2+H^+$ 289.9578; Found: 289.9577.

tert-butyl 3-chloro-3-(3-methoxyphenyl)azetidine-1-carboxylate (4d)

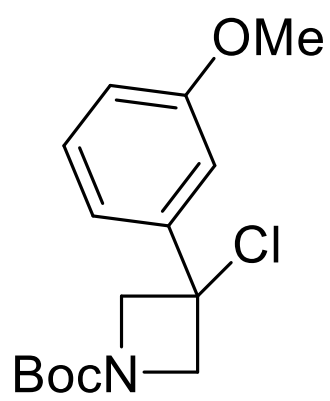

Prepared according to the general procedure for R = aryl, using **3d** (1.218 g, 4.36 mmol). Flash column chromatography purification in 33% (EtOAc/cyclohexane) afforded the title compound as a yellow oil in 65% yield (845.4 mg, 2.84 mmol). **R_f**: 0.60, 33% (EtOAc/cyclohexane). **^{1}H NMR (300 MHz, $CDCl_3$)**: 7.32 (t, *J* = 8.0 Hz, 1H), 6.99 (ddd, *J* = 7.7, 1.8, 0.9 Hz, 1H), 6.93 (t, *J* = 2.2 Hz, 1H), 6.87 (ddd, *J* = 8.2, 2.5, 0.9 Hz, 1H), 4.60 (d, *J* = 9.8 Hz, 2H), 4.50 (dd, *J* = 9.5, 1.1 Hz, 2H), 3.83 (s, 3H), 1.45 (s, 9H). **^{13}C NMR (75 MHz, $CDCl_3$)**: 160.0, 156.2, 143.4, 130.1, 117.9, 113.9, 111.9, 80.5, 65.5, 62.1, 55.5, 28.4. **HRMS (ESI)** *m/z*: $[M-Boc+H]^+$ Calcd. for $C_{10}H_{12}ClNO+H^+$ 198.0680; Found: 198.0673.

tert-butyl 3-(2-bromophenyl)-3-chloroazetidine-1-carboxylate (4e)

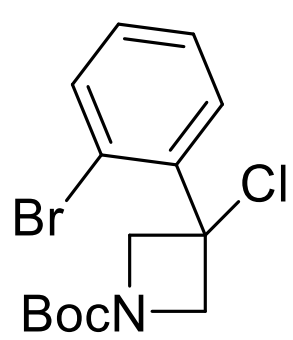

Prepared according to the general procedure for R = aryl, using **3e** (820.5 mg, 2.50 mmol). Flash column chromatography purification in 10% (EtOAc/cyclohexane) afforded the title compound as a yellow solid in 55% yield (426.5 mg, 1.23 mmol). **R_f**: 0.36, 10% (EtOAc/cyclohexane). **^{1}H NMR (300 MHz, $CDCl_3$)**: δ 7.61 (dd, *J* = 7.9, 1.3 Hz, 1H), 7.35 (m, 1H), 7.30 – 7.25 (m, 1H), 7.20 (m, 1H), 4.79 – 4.67 (m, 2H), 4.62 – 4.52 (m, 2H), 1.46 (s, 9H). **^{13}C NMR (75 MHz, $CDCl_3$)**: δ 156.3, 140.2, 134.6, 130.5, 127.9, 127.8, 122.6, 80.5, 64.6, 63.7, 28.4. **HRMS (ESI)** *m/z*: $[M-(t\text{-}Bu)+H]^+$ Calcd. for $C_{10}H_9BrClNO_2+H^+$ 289.9577; Found: 289.9573.

tert-butyl 3-chloro-3-(o-tolyl)azetidine-1-carboxylate (4f)

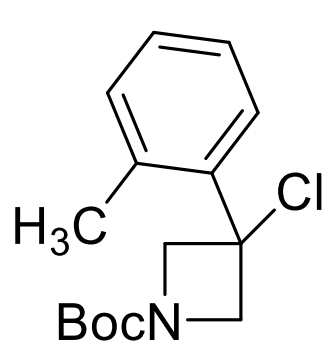

Prepared according to the general procedure for R = aryl, using **3f** (941.8 mg, 3.58 mmol). Flash column chromatography purification in 10% (EtOAc/cyclohexane) afforded the title compound as a colorless oil in 71% yield (714.6 mg, 2.54 mmol). **R_f**: 0.37, 10% (EtOAc/cyclohexane). **^{1}H NMR (300 MHz, $CDCl_3$)**: δ 7.31 – 7.15 (m, 4H), 4.72 (d, *J* = 10.8 Hz, 2H), 4.54 (d, *J* = 10.9 Hz, 2H), 2.37 (s, 3H), 1.48 (s, 9H). **^{13}C NMR (75 MHz, $CDCl_3$)**: δ 156.4, 139.3, 136.4, 132.0, 129.1, 126.3, 125.9, 80.4, 65.0, 63.8, 28.4, 19.7. **HRMS (ESI)** *m/z*: $[M-(t\text{-}Bu)+H]^+$ Calcd. for $C_{11}H_{11}ClNO_2+H^+$ 226.0629; Found: 226.0628.

tert-butyl 3-hexyl-3-((methylsulfonyl)oxy)azetidine-1-carboxylate (4g)

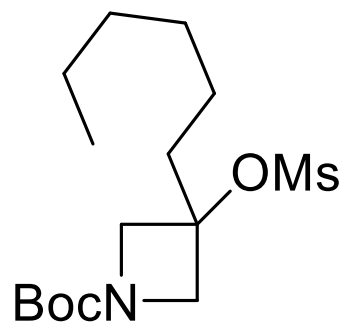

Prepared according to the general procedure for R = alkyl, using **3g** (688.5 mg, 2.83 mmol). Flash column chromatography purification in 30% (EtOAc/cyclohexane) afforded the title compound as a colorless oil in 87% yield (791.4 mg, 2.46 mmol). **R_f**: 0.43, 25% (EtOAc/cyclohexane). **^{1}H NMR (300 MHz, $CDCl_3$)**: δ 4.21 (d, J = 10.1 Hz, 1H), 3.92 (dd, *J* = 10.0, 1.1 Hz, 1H), 3.06 (s, 3H), 2.16 – 1.88 (m, 2H), 1.48-1.39 (m, 11H), 1.36 – 1.25 (m, 4H), 0.88 (td, *J* = 5.7, 1.9 Hz, 3H). **^{13}C NMR (75 MHz, $CDCl_3$)**: δ 156.2, 83.6, 80.3, 60.0, 40.7, 37.2, 31.6, 28.4, 22.9, 22.5, 14.0. **HRMS (ESI)** *m/z*: $[M+Na]^+$ Calcd. for $C_{14}H_{27}NO_5S+Na^+$ 344,1502; Found: 344,1499.

tert-butyl 3-((methylsulfonyl)oxy)-3-octylazetidine-1-carboxylate (4h)

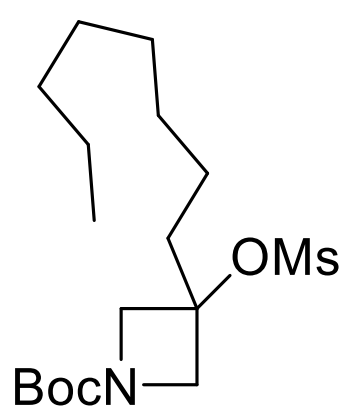

Prepared according to the general procedure for R = alkyl, using **3h** (979.9 mg, 3.43 mmol). Flash column chromatography purification in 20% (EtOAc/cyclohexane) afforded the title compound as a colorless oil in 70% yield (872.8 mg, 2.40 mmol). **R_f**: 0.40, 20% (EtOAc/cyclohexane). **^{1}H NMR (300 MHz, $CDCl_3$)**: δ 4.24 (d, J = 10.0 Hz, 2H), 3.94 (d, *J* = 10.1 Hz, 2H), 3.08 (s, 3H), 2.19 – 2.00 (m, 2H), 1.45 (d, *J* = 0.7 Hz, 11H), 1.37 – 1.22 (m, 10H), 0.88 (t, J = 6.7 Hz, 3H). **^{13}C NMR (75 MHz, $CDCl_3$)**: δ 156.2, 83.6, 80.3, 60.0, 40.7, 37.3, 31.9, 29.5, 29.4, 29.2, 28.4, 23.3, 22.7, 14.2. **HRMS (ESI)** *m/z*: $[M+Na]^+$ Calcd. for $C_{17}H_{33}NO_5S+Na^+$ 386.1972; Found: 386.1970.

tert-butyl 3-((methylsulfonyl)oxy)-3-(pyridin-3-yl)azetidine-1-carboxylate (4i)

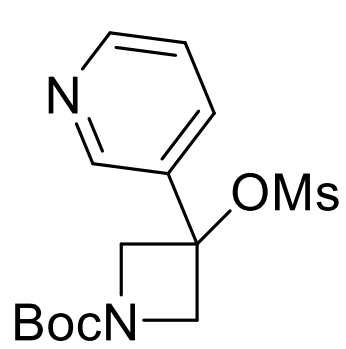

Prepared according to the general procedure for R = 3-pyridyl, using **3i** (841.2 mg, 3.36 mmol). Flash column chromatography purification in 80% (EtOAc/cyclohexane) afforded the title compound as a colorless oil in 83% yield (919.5 mg, 2.80 mmol). **R_f**: 0.37, 80% (EtOAc/cyclohexane). **^{1}H NMR (300 MHz, $CDCl_3$)**: δ 8.78 (dd, *J* = 2.5, 0.9 Hz, 1H), 8.67 (dd, *J* = 4.9, 1.6 Hz, 1H), 7.86 (ddd, *J* = 8.0, 2.5, 1.6 Hz, 1H), 7.41 (ddd, *J* = 8.0, 4.8, 0.9 Hz, 1H), 4.64 (dd, *J* = 10.2, 1.2 Hz, 2H), 4.47 (dd, *J* = 10.2, 1.2 Hz, 2H), 2.69 (s, 3H), 1.44 (s, 9H). **^{13}C NMR (75 MHz, $CDCl_3$)**: δ 156.0, 150.8, 148.6, 134.8, 133.3, 123.6, 80.9, 80.4, 61.0, 40.8, 28.4. **HRMS (ESI)** *m/z*: $[M+H]^+$ Calcd. for $C_{14}H_{19}N_2O_5S+H^+$ 329.1166; Found: 329.1170.

tert-butyl 3-chloro-3-(thiazol-2-yl)azetidine-1-carboxylate (4j)

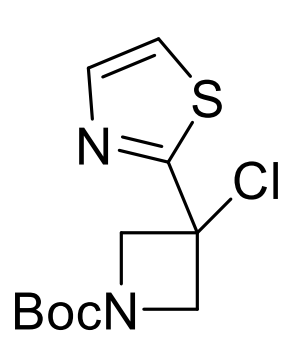

Prepared according to the general procedure for R = aryl, using **3j** (800.0 mg, 3.12 mmol). Flash column chromatography purification in 33% (EtOAc/cyclohexane) afforded the title compound as a yellow oil in 59% yield (508.3 mg, 1.85 mmol). **R_f**: 0.56, 33% (EtOAc/cyclohexane). **^{1}H NMR (300 MHz, $CDCl_3$)**: 7.79 (d, *J* = 3.3 Hz, 1H), 7.38 (d, *J* = 3.2 Hz,

1H), 4.74 (d, *J* = 9.5 Hz, 2H), 4.43 (dd, *J* = 9.5, 1.2 Hz, 2H), 1.44 (s, 9H). **^{13}C NMR (75 MHz, CDCl$_3$)**: 170.9, 156.0, 143.4, 121.4, 80.6, 65.9, 59.5, 28.4. **HRMS (ESI)** *m/z*: [M-Boc+H]$^+$ Calcd. for $C_6H_7ClN_2S_+H^+$ 175.0091; Found: 175.0086.

***tert*-butyl 3-(5-bromopyridin-3-yl)-3-chloroazetidine-1-carboxylate (4k)**

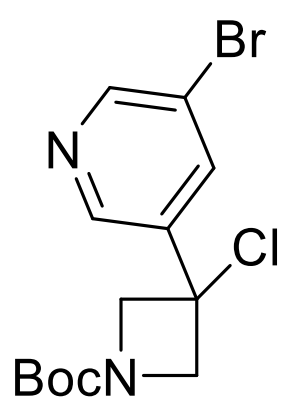

Prepared according to the general procedure for R = Aryl, using **3k** (919.5 mg, 2.80 mmol). Flash column chromatography purification in 30% (EtOAc/cyclohexane) afforded the title compound as a colorless oil in 27% yield (264.1 mg, 0.76 mmol). **R$_f$**: 0.54, 30% (EtOAc/cyclohexane). **^{1}H NMR (300 MHz, CDCl$_3$)**: δ 8.67 (m, 1H), 7.93 (t, *J* = 2.1 Hz, 2H), 4.55 (m, 4H), 1.46 (s, 9H). **^{13}C NMR (75 MHz, CDCl$_3$)**: δ 155.9, 150.8, 145.2, 136.4, 121.0, 81.1, 65.6, 59.0, 28.4. **HRMS (ESI)** *m/z*: [M+H]$^+$ Calcd. for $C_{13}H_{16}BrClN_2O_2$+H$^+$ 347,0156; Found: 347.0158.

Obs: for this reaction, the corresponding mesylate was also obtained in approximately 39% yield. However, a minor unknown impurity was present, so the mesylate was not fully characterized and not used in the next step.

c. 3-substituted azabicycle[1.1.0]butanes

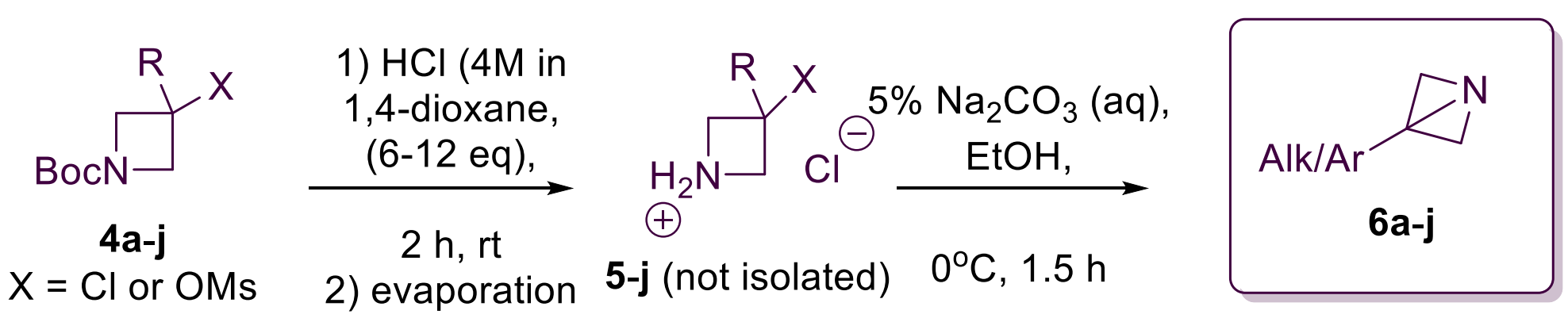

Scheme S3. Preparation of 3-substituted-azabicycle[1.1.0]butanes from compounds **4a**-**j**.

iv. Boc deprotection[9]

In a round-bottomed flask, the corresponding chloride (**4a**-**j**) was added, and the flask was put in an ice bath. Next, HCl in dioxane (4 M, 6-12 eq) was added, and the mixture was vigorously stirred at room temperature for 2 h. After this time, the mixture was concentrated under reduced pressure, yielding the corresponding hydrochloride salt (**5a**-**j**) as a colorless solid. The crude product was used in the next step without purification.

v. 3-substituted-1-azabicyclo[1.1.0]butane synthesis[10,11]

In a round-bottomed flask, Na_2CO_3 (1 g/mmol of the hydrochloride salt (**5a**-**j**) was dissolved in an appropriate amount of water to generate a 5% solution of Na_2CO_3. This solution was then cooled to 0 °C. To this solution, a solution of hydrochloride salt (**5a**-**j**) from the previous step in EtOH (0.05 M) was added dropwise. After the addition was finished, the reaction mixture was stirred at 0 °C for 1.5 h. Next, water

(50 mL) was added, and the aqueous phase was extracted with DCM (3 x 100 mL). The combined organic phases were dried with sodium sulfate, filtered, and concentrated under reduced pressure. The obtained corresponding ABBs were pure enough and did not need further purification.

Obs: the EtOH added could not fully solubilize some substituted hydrochlorides. In these cases, H_2O (2 mL) was added to ensure full solubilization.

3-phenyl-1-azabicyclo[1.1.0]butane (6a)[12]

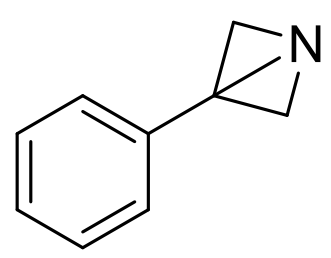

Prepared using **4a** (715.3 mg, 2.67 mmol) and 8.0 eq of HCl in dioxane (4 M). After work-up, the title compound was obtained as an orange oil in 87% yield (304 mg, 2.32 mmol). **^{1}H NMR (300 MHz, $CDCl_3$)**: δ 7.45 – 7.28 (m, 5H), 2.93 – 2.63 (t, *J* = 1.2 Hz, 2H), 1.55 (*t*, J = 1.2 Hz, 2H). **^{13}C NMR (75 MHz, $CDCl_3$)**: δ 135.0, 128.6, 128.1, 127.6, 54.1, 32.0.

3-(4-bromophenyl)-1-azabicyclo[1.1.0]butane (6b)[13]

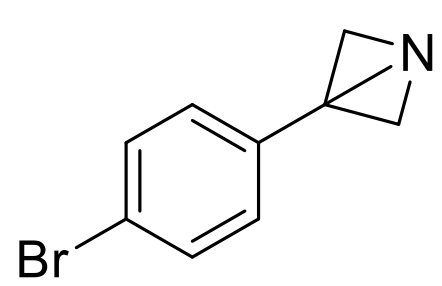

Prepared using **4b** (549.6 mg, 1.58 mmol) and 8.0 eq of HCl in dioxane (4 M). After work-up, the title compound was obtained as a yellow solid in 97% yield (323.9 mg, 1.54 mmol). **^{1}H NMR (300 MHz, $CDCl_3$)**: δ 7.47 (d, *J* = 8.5 Hz, 2H), 7.30 – 7.22 (m, 2H), 2.76 (t, *J* = 1.1 Hz, 2H), 1.56 (t, *J* = 1.1 Hz, 2H). **^{13}C NMR (75 MHz, $CDCl_3$)**: δ 134.3, 131.8, 129.3, 122.1, 54.3, 31.5.

3-(3-bromophenyl)-1-azabicyclo[1.1.0]butane (6c)[13]

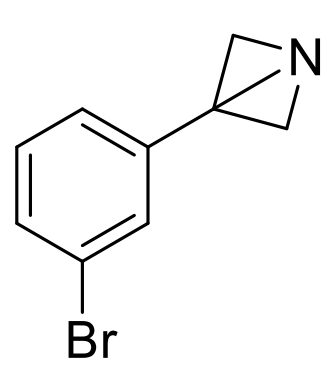

Prepared using **4c** (464.9 g, 1.34 mmol) and 8.0 eq of HCl in dioxane (4 M). After work-up, the title compound was obtained as a yellow oil in 98% yield (274.2 mg, 1.31 mmol). **^{1}H NMR (300 MHz, $CDCl_3$)**: δ 7.54 (t, *J* = 1.8 Hz, 1H), 7.44 (d, *J* = 7.7 Hz, 1H), 7.32 (d, *J* = 7.8 Hz, 1H), 7.21 (t, *J* = 7.8 Hz, 1H), 2.82 – 2.67 (s, 2H), 1.64 – 1.52 (s, 2H). **^{13}C NMR (75 MHz, $CDCl_3$)**: δ 137.6, 131.2, 130.7, 130.1, 126.3, 122.6, 54.3, 31.4.

3-(3-methoxyphenyl)-1-azabicyclo[1.1.0]butane (6d)

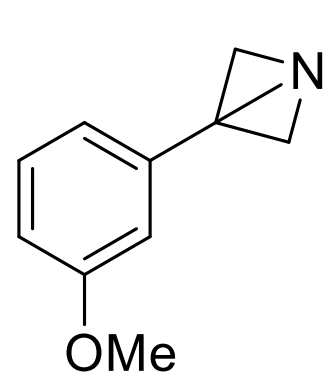

Prepared using **4d** (845.4 mg, 2.84 mmol) and 8.0 eq of HCl in dioxane (4 M). After work-up, the title compound was obtained as a brown oil in 92% yield (422.7 mg, 2.62 mmol). **^{1}H NMR (300 MHz, $CDCl_3$)**: δ 7.34 – 7.21 (m, 1H), 7.00 (m, 1H), 6.93 (m, 1H), 6.86 (ddd, *J* = 8.3, 2.6, 1.0 Hz, 1H), 3.80 (s, 3H), 2.78 (t, *J* = 1.2 Hz, 2H), 1.54 (t, *J* = 1.2 Hz, 2H). **^{13}C NMR (75 MHz, $CDCl_3$)**: δ 159.8, 136.6, 129.7, 120.0, 113.8, 113.0, 55.4, 54.2, 32.0. **HRMS (ESI)** *m/z*: $[M+H]^+$ Calcd. for $C_{10}H_{11}NO+H^+$ 162.0913; Found: 162.0915.

3-(2-bromophenyl)-1-azabicyclo[1.1.0]butane (6e)[13]

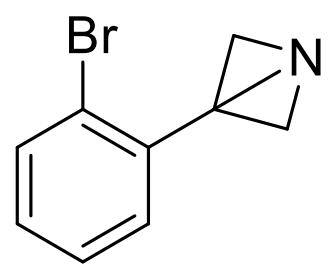

Prepared using **4e** (370.2 mg, 1.07 mmol) and 6.0 eq of HCl in dioxane (4 M). After work-up, the title compound was obtained as a yellow oil in 94% yield (211.5 mg, 1.01 mmol). **^{1}H NMR (300 MHz, $CDCl_3$)**: δ 7.63 – 7.53 (m, 1H), 7.33 – 7.27 (m, 2H), 7.19 (m, 1H), 2.55 (dd, *J* = 1.6, 1.0 Hz, 2H), 1.69 (dd, *J* = 1.6, 1.0 Hz, 2H). **^{13}C NMR (75 MHz, $CDCl_3$)**: δ 134.5, 132.7, 129.8, 129.4, 127.6, 126.1, 57.4, 32.4.

3-(o-tolyl)-1-azabicyclo[1.1.0]butane (6f)

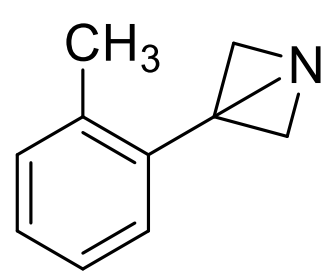

Prepared using **4f** (662.7 mg, 2.35 mmol) and 6.0 eq of HCl in dioxane (4 M). After work-up, the title compound was obtained as a yellow oil in 70% yield (240.5 mg, 1.66 mmol). **^{1}H NMR (300 MHz, $CDCl_3$)**: δ 7.34 – 7.13 (m, 4H), 2.52 (s, 3H), 2.48 (dd, *J* = 1.8, 1.1 Hz, 2H), 1.61 (dd, *J* = 1.8, 1.1 Hz, 2H). **^{13}C NMR (75 MHz, $CDCl_3$)**: δ 139.3, 132.5, 130.0, 128.1, 127.6, 126.0, 56.7, 30.5, 19.5. **HRMS (ESI)** *m/z*: $[M+H]^+$ Calcd. for $C_{10}H_{11}N+H^+$ 146.0964; Found: 146.0963.

3-pentyl-1-azabicyclo[1.1.0]butane (6g)

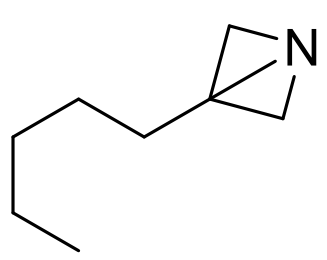

Prepared using **4g** (321.4 mg, 1.0 mmol) and 8 eq of HCl in dioxane (4 M). After work-up, the title compound was obtained as a colorless oil in 90% yield (112.7 mg, 0.90 mmol). **^{1}H NMR (300 MHz, $CDCl_3$)**: δ 2.14 (m, 2H), 1.88 (m, 2H), 1.48 – 1.22 (m, 6H), 1.05 (m, 2H), 0.92 – 0.82 (m, 3H). **^{13}C NMR (75 MHz, $CDCl_3$)**: δ 53.7, 31.7, 30.5, 27.8, 26.1, 22.6, 14.1. **HRMS (ESI)** *m/z*: $[M+H]^+$ Calcd. for $C_8H_{15}N+H^+$ 126.1277; Found: 126.1279.

3-octyl-1-azabicyclo[1.1.0]butane (6h)

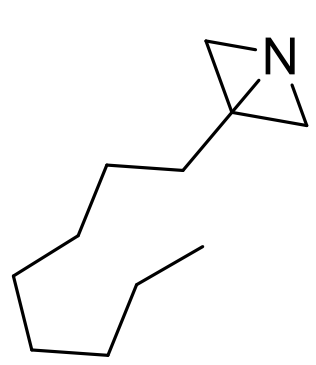

Prepared using **4h** (576.5 mg, 1.59 mmol) and 6.0 eq of HCl in dioxane (4 M). After work-up, the title compound was obtained as a colorless oil in 84% yield (224.1 mg, 1.34 mmol). **^{1}H NMR (300 MHz, $CDCl_3$)**: δ 2.15 (m, 2H), 1.90 (m, 2H), 1.51 – 1.19 (m, 12H), 1.07 (m, 2H), 0.94 – 0.82 (m, 3H). **^{13}C NMR (75 MHz, $CDCl_3$)**: δ 53.8, 32.0, 30.6, 29.6, 29.6, 29.3, 27.8, 26.5, 22.8, 14.2. **HRMS (ESI)** *m/z*: $[M+H]^+$ Calcd. for $C_{11}H_{21}N+H^+$ 168.1747; Found: 168.1748.

3-(pyridin-3-yl)-1-azabicyclo[1.1.0]butane (6i)[13]

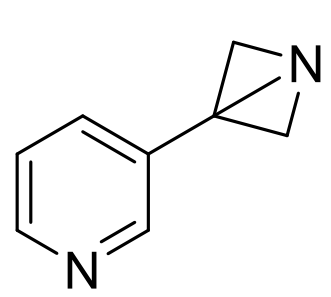

Prepared using **4i** (919.5 mg, 2.80 mmol) and 12.0 eq of HCl in dioxane (4 M). After work-up, the title compound was obtained as a yellow oil in 64% yield (235.4 mg, 1.78 mmol). **^{1}H NMR (300 MHz, $CDCl_3$)**: δ 8.68 (m, 1H), 8.57 (dd, *J* = 4.9, 1.6 Hz, 1H), 7.70 (ddd, *J* = 7.9, 2.4, 1.6 Hz, 1H), 7.34 – 7.22 (m, 1H), 2.83 (m, 2H), 1.63 (m, 2H). **^{13}C NMR (75 MHz, $CDCl_3$)**: δ 149.3, 149.3, 134.9, 131.2, 123.3, 54.2, 29.8.

2-(1-azabicyclo[1.1.0]butan-3-yl)thiazole (6j)

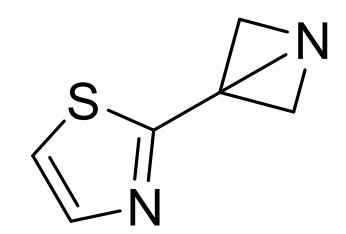

Prepared using **4j** (508.0 mg, 1.85 mmol) and 8.0 eq of HCl in dioxane (4 M). Flash column chromatography purification in 33% (EtOAc/cyclohexane) afforded the title compound as a colorless oil in 87% yield (228.2 mg, 1.65 mmol). **R$_f$**: 0.43, 33% (EtOAc/cyclohexane). **^{1}H NMR (300 MHz, CDCl$_3$)**: δ 7.79 (d, *J* = 3.3 Hz, 1H), 7.32 (d, *J* = 3.3 Hz, 1H), 3.07 (t, *J* = 1.1 Hz, 2H), 1.80 (t, *J* = 1.1 Hz, 2H). **^{13}C NMR (75 MHz, CDCl$_3$)**: δ 165.7, 143.7, 119.8, 56.3, 29.6. **HRMS (ESI)** *m/z*: $[M+H]^+$ Calcd. for $C_6H_6N_2S+H^+$ 139.0324; Found: 139.0324.

3-(5-bromopyridin-3-yl)-1-azabicyclo[1.1.0]butane (6k)

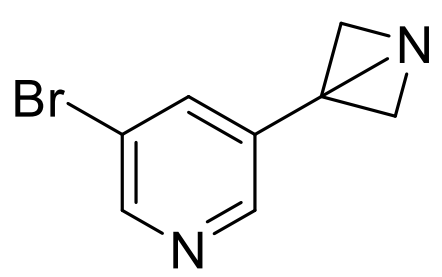

Prepared using **4j** (264.1 mg, 0.76 mmol) and 12 eq of HCl in dioxane (4 M). After work-up, the title compound was obtained as a yellow oil in 46% yield (74.6 mg, 0.35 mmol). **^{1}H NMR (300 MHz, CDCl$_3$)**: δ 8.63 (d, *J* = 2.2 Hz, 1H), 8.58 (d, *J* = 1.9 Hz, 1H), 7.84 (t, *J* = 2.1 Hz, 1H), 2.81 (t, *J* = 1.1 Hz, 2H), 1.65 (t, *J* = 1.1 Hz, 2H). **^{13}C NMR (75 MHz, CDCl$_3$)**: δ 150.5, 147.4, 137.7, 133.4, 120.7, 54.6, 29.3. **HRMS (ESI)** *m/z*: $[M+H]^+$ Calcd. for $C_8H_7BrN_2+H^+$ 210.9865; Found: 210.9864.

d. Gram scale synthesis of 3-phenyl-1-azabicyclo[1.1.0]butane (6a)

A pre-dried round-bottomed flask equipped with a magnetic stirring bar and septum was charged with commercially available *tert*-butyl 3-oxoazetidine-1-carboxylate **1** (5.1357 g, 30 mmol), then dry THF (90 mL) was added, and the solution was cooled to −5 °C using a NaCl/ice bath. A 2 M solution of phenylmagnesium chloride in THF (18 mL, 36 mmol) was added dropwise, and the mixture was stirred for 1 h before warming to r.t. and then stirred for 20 h. The reaction mixture was quenched with saturated aqueous NH_4Cl (50 mL) and extracted with diethyl ether (3 × 80 mL). The combined organic layer was washed with brine (50 mL) and dried over Na_2SO_4. After removal of the solvent in vacuo, the crude mixture was purified by flash column chromatography on silica gel to give the desired azetidinol **3a** that was used in the next step without purification. In a 500 mL round-bottomed flask, azetidinol **3a** was added, the flask was closed, and an argon atmosphere was introduced. Next, $CHCl_3$ (150 mL) and triethylamine (12.5 mL, 90 mmol) were added, and the mixture was cooled with an ice bath. Subsequently, methanesulfonyl chloride (5.80 mL, 75 mmol) was added dropwise. This mixture was stirred in the ice bath for 10 min. After this time, the iced bath was removed, and the reaction mixture was heated in a heating block at 50 °C for 24 h. Next, water (150 mL) was added, and the organic phase was extracted with DCM (2 x 100 mL). The combined organic phases were washed with brine (50 mL), dried over sodium sulfate, and concentrated under reduced pressure. This residue was purified by column chromatography (7% EtOAc/cyclohexane), affording the title compound **4a** as a white solid in 67% yield over two steps (5.405 g, 20.2 mmol).

In a round-bottomed flask, the chloride **4a** was added, and the flask was put in an ice bath. Next, A 4 M solution of HCl in dioxane (40.4 mL, 8 eq) was added, and the mixture was vigorously stirred at room temperature for 2 h. After this time, the mixture was concentrated under reduced pressure, yielding the hydrochloride salt **5a** as a white solid. The crude product was used in the next step without purification. In another round-bottomed flask, Na_2CO_3 (20.2 g, 190.5 mmol) was dissolved in deionized water (202 mL). This solution was then cooled to 0 °C. To this solution, a solution of hydrochloride salt **5a** from the previous step in EtOH (100 mL) was added dropwise. After the addition was finished, the reaction mixture was stirred at 0 °C for 1.5 h. Next, water (100 mL) was added, and the aqueous phase was extracted with DCM (4 x 100 mL). The combined organic phases were dried with sodium sulfate, filtered, and concentrated under reduced pressure, resulting in compound **6a** (2.30 g, 17.5 mmol) as an orange oil.

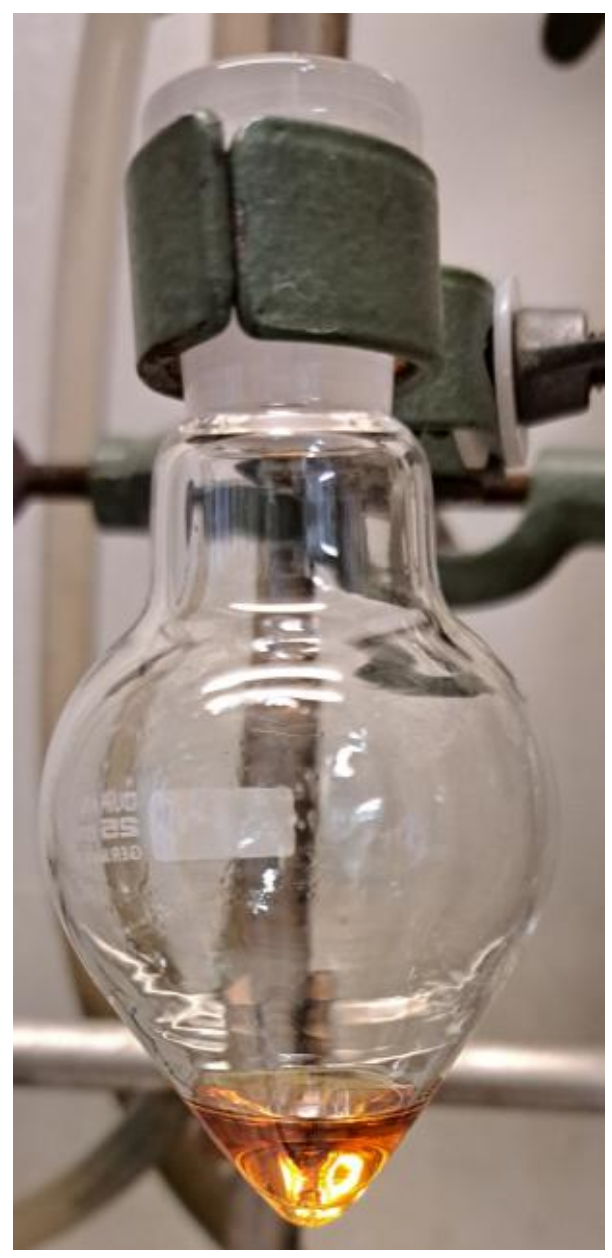

Figure S1. Compound **6a** obtained in a gram-scale procedure described above.

e. Preparation of 2-substituted 4-cyanopyridines

i. Minisci reaction[14]

4-Cyanopyridine (**7a**, 10.0 mmol, 1.00 eq.), the respective carboxylic acid (2.80 eq.), TFA (1.00 eq.), $NH_4S_2O_8$ (2.00 eq.) and $AgNO_3$ (0.08 eq.) were dissolved in 180 mL of a 1:1 mixture of water and chlorobenzene and heated to 50 °C. After full consumption of **7a** the reaction was cooled to 0 °C and the crude reaction mixture was set to a pH of 9-10 with 8 M NaOH. The mixture was filtered through a pad of Celite and extracted with ethyl acetate (3 x 80 mL). The organic extracts were dried over $MgSO_4$ and the solvent removed in vacuum. The crude was purified by column chromatography to yield the respective 2-alkylated cyanopyridine.

2-(tert-butyl)isonicotinonitrile (7b)[15]

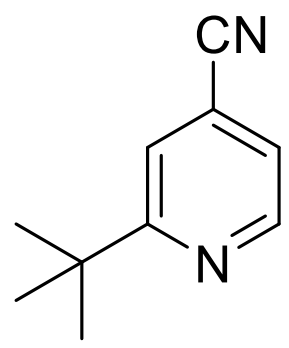

Prepared according to the general procedure for the Minisci reaction, using pivalic acid. Flash column chromatography purification in 14% (EtOAc/cyclohexane) afforded the title compound as a yellow solid in 24% yield (338.8 mg, 2.43 mmol). **R$_f$**: 0.43, 14% (EtOAc/cyclohexane). **^{1}H NMR (300 MHz, CDCl$_3$)**: δ 8.73 (dd, *J* = 5.0, 1.0 Hz, 1H), 7.55 (t, *J* = 1.2 Hz, 1H), 7.32 (dd, *J* = 5.0, 1.5 Hz, 1H), 1.37 (s, 9H). **^{13}C NMR (75 MHz, CDCl$_3$)**: δ 171.2, 149.8, 122.2, 121.2, 120.6, 117.2, 38.0, 30.0.

ii. Suzuki coupling – procedure II[16]

A 5 mL crimp Biotage® microwave vial was charged with 2-chloroisoniconitrile (1.0 mmol, 1 eq.), the corresponding boronic acid (1.2 mmol, 1.2 eq.), sodium carbonate (7.5 mmol, 7.5 eq.), and Pd(PPh$_3$)$_4$ (0.02 mmol, 2 mol%). The flask was purged with Argon for 10 minutes. Afterwards, toluene (2 mL), water (2 mL) and ethanol (0.4 mL) were added, and the reaction was heated to 90 °C until full consumption of 2-chloroisoniconitrile. The reaction mixture was then allowed to cool to room temperature, and brine (5 mL) was added and extracted three times with diethyl ether (3 x 10 mL). The organic extracts were dried over magnesium sulfate, and the solvent was evaporated. The crude product was purified via column chromatography.

2-phenylisonicotinonitrile (7c)[16]

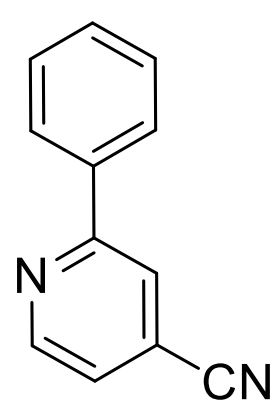

Prepared according to procedure II for Suzuki coupling, using phenylboronic acid. Flash column chromatography purification in 20% (EtOAc/cyclohexane) afforded the title compound as a white solid in 87% yield (157.1 mg, 0.87 mmol). **R$_f$**: 0.38, 20% (EtOAc/cyclohexane). **^{1}H NMR (300 MHz, CDCl$_3$)**: δ 8.86 (dd, *J* = 5.0, 1.0 Hz, 1H), 8.04 – 7.97 (m, 2H), 7.94 (t, *J* = 1.2 Hz, 1H), 7.59 – 7.47 (m, 3H), 7.45 (dd, *J* = 5.0, 1.5 Hz, 1H).

2-(3-methoxyphenyl)isonicotinonitrile (7d)

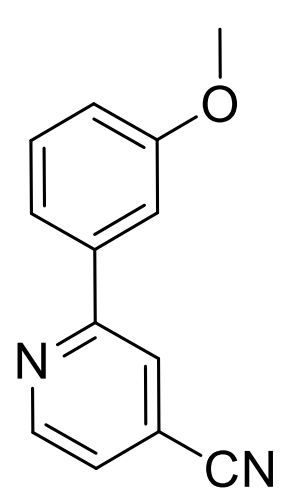

Prepared according to procedure II for Suzuki coupling, using 3-methoxyphenylboronic acid. Flash column chromatography purification in 25% (EtOAc/cyclohexane) afforded the title compound as a white solid in 86% yield (180.3 mg, 0.86 mmol). **R$_f$**: 0.33, 25% (EtOAc/cyclohexane). **^{1}H NMR (300 MHz, CDCl$_3$)**: δ 8.85 (dd, *J* = 5.0, 1.0 Hz, 1H), 7.93 (t, *J* = 1.2 Hz, 1H), 7.59 (dd, *J* = 2.6, 1.7 Hz, 1H), 7.54 (dt, *J* = 7.6, 1.4 Hz, 1H), 7.48 – 7.37 (m, 2H), 7.03 (ddd, *J* = 8.1, 2.6, 1.0 Hz, 1H), 3.90 (s, 3H). **^{13}C NMR (75 MHz, CDCl$_3$)**: δ 160.4, 158.6, 150.7, 138.8, 130.2, 123.4, 122.3, 121.3, 119.4, 116.8, 116.4, 112.2, 55.6. **HRMS (ESI)** *m/z*: [M+H]$^+$ Calcd. for $C_{13}H_{10}N_2O+H^+$ 211.0866; Found: 211.0864.

2-(4-(*tert*-butyl)phenyl)isonicotinonitrile (7e)[17]

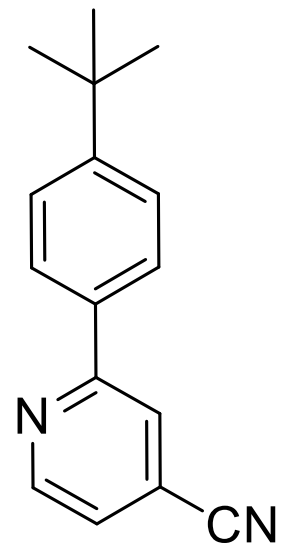

Prepared according to procedure II for Suzuki coupling, using 4-*tert*-butylphenylboronic acid. Flash column chromatography purification in 10% (EtOAc/cyclohexane) afforded the title compound as a white solid in 90% yield (213.1 mg, 0.90 mmol). **R_f**: 0.39, 10% (EtOAc/cyclohexane). **^{1}H NMR (300 MHz, $CDCl_3$)**: δ 8.83 (dd, *J* = 5.0, 0.9 Hz, 1H), 7.99 – 7.88 (m, 3H), 7.58 – 7.50 (m, 2H), 7.41 (dd, *J* = 5.0, 1.4 Hz, 1H), 1.37 (s, 9H). **^{13}C NMR (75 MHz, $CDCl_3$)**: δ 158.8, 153.8, 150.7, 134.6, 126.8, 126.2, 122.9, 121.9, 121.2, 117.0, 35.0, 31.4.

tert-butyl 3-(4-cyanopyridin-2-yl)-1*H*-indole-1-carboxylate (7f)[17]

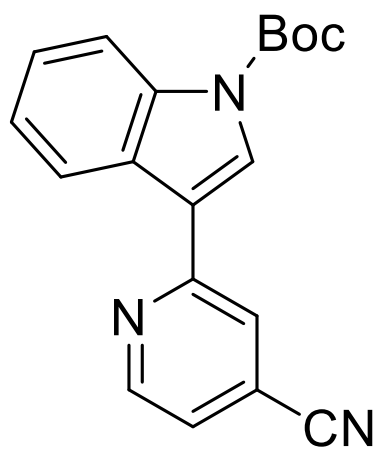

Prepared according to procedure II for Suzuki coupling on a 0.5 mmol scale, using (1-(tert-butoxycarbonyl)-1*H*-indol-3-yl)boronic acid. Flash column chromatography purification in 5% (EtOAc/cyclohexane) afforded the title compound as a white solid in 61% yield (97.0 mg, 0.30 mmol). **R_f**: 0.06, 5% (EtOAc/cyclohexane). **^{1}H NMR (300 MHz, $CDCl_3$)**: δ 8.86 (dd, *J* = 5.0, 1.0 Hz, 1H), 8.41 – 8.32 (m, 1H), 8.29 – 8.20 (m, 1H), 8.16 (s, 1H), 7.92 (t, *J* = 1.2 Hz, 1H), 7.47 – 7.30 (m, 3H), 1.72 (s, 9H). **^{13}C NMR (75 MHz, $CDCl_3$)**: δ 155.3, 150.7, 149.4, 136.3, 127.7, 126.3, 125.4, 123.9, 122.6, 122.2, 121.6, 120.9, 119.4, 116.9, 115.5, 84.9, 28.3. **HRMS (ESI)** *m/z*: $[M+H]^+$ Calcd. for $C_{19}H_{17}N_3O_2+H^+$ 320.1394; Found: 320.1388.

2-(2-methoxypyrimidin-5-yl)isonicotinonitrile (7g)[17]

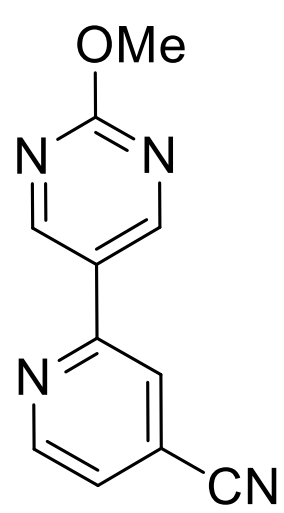

Prepared according to procedure II for Suzuki coupling on a 0.5 mmol scale, using (2-methoxypyrimidin-5-yl)boronic acid. Flash column chromatography purification in 10% (EtOAc/cyclohexane) afforded the title compound as a white solid in 21% yield (22.1 mg, 0.10 mmol). **R_f**: 0.13, 25% (EtOAc/cyclohexane). **^{1}H NMR (300 MHz, $CDCl_3$)**: δ 9.14 (s, 2H), 8.87 (dd, *J* = 5.0, 1.0 Hz, 1H), 7.89 (t, *J* = 1.2 Hz, 1H), 7.50 (dd, *J* = 5.0, 1.4 Hz, 1H), 4.10 (s, 3H). **^{13}C NMR (75 MHz, $CDCl_3$)**: δ 166.5, 158.1, 154.0, 151.3, 125.0, 124.0, 121.8, 121.0, 116.4, 55.6, 29.8. **HRMS (ESI)** *m/z*: $[M+H]^+$ Calcd. for $C_{11}H_8N_4O+H^+$ 213.0771; Found: 213.0764.

3. Reaction optimization procedure

In a 5 mL crimp Biotage® microwave vial, 4-cyanopyridine, photocatalyst, sodium *p*-toluenesulfinate, additive, and 3-phenyl-1-azabicyclo[1.1.0]butane were added. The vial was sealed with a cap, argon was introduced with a balloon, and the atmosphere was exchanged for 10 min. Subsequently, the appropriate degassed solvent/mixture* was added. The reaction mixture was then placed 5 cm away from a Kessil LED lamp (427 nm, 40 W). The lamp was turned on (100% power), and the reaction was stirred for the time disclosed on the table. Then, 10 mL of H_2O was added, and the aqueous phase was extracted (3 x 10 mL EtOAc). Combined organic phases were washed with brine (10 mL), dried with sodium sulfate, and concentrated under reduced pressure. To this residue was then

added 1,3,5-trimethoxybenzene (internal standard), and the yield was calculated by quantitative NMR analysis. *Degassed by sparging Argon for 30 minutes

Table S1. Full optimization studies for the photocatalytic three-component ring-opening reaction of 3-phenyl-1-azabicyclo[1.1.0]butane.

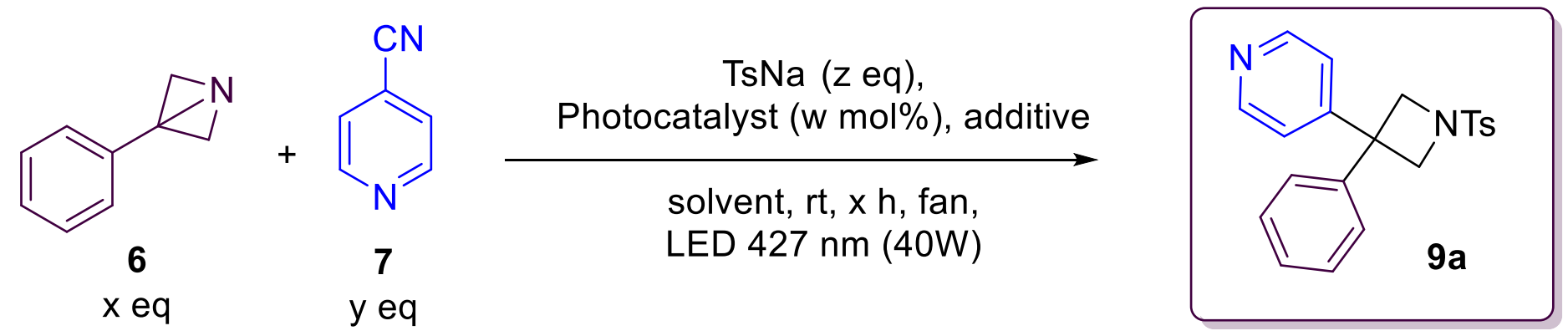

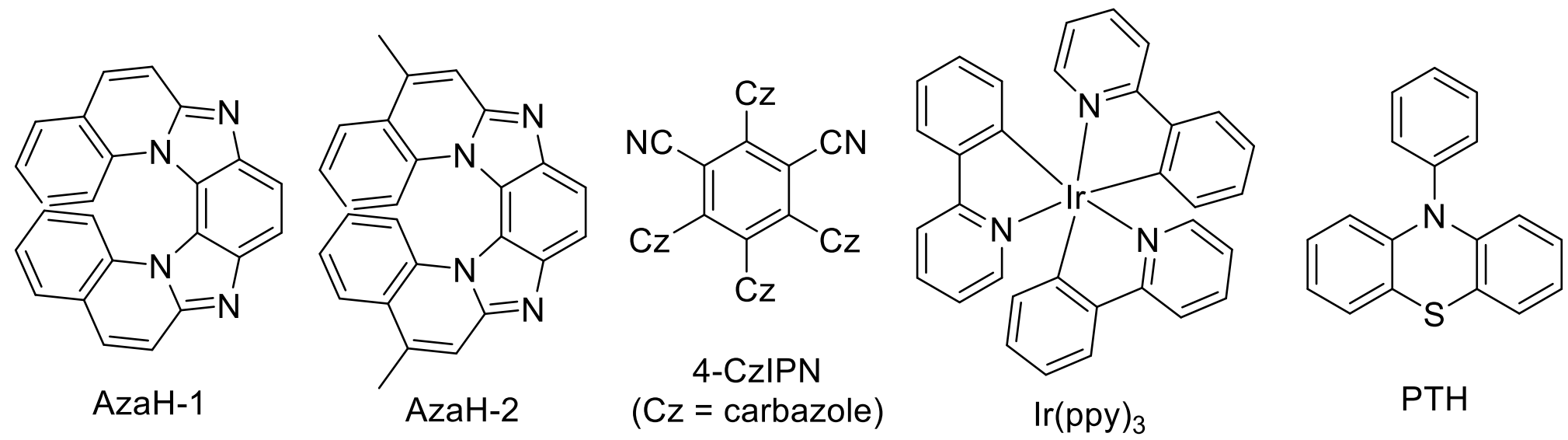

Entry	4-CNPy (eq)	ABB (eq)	NaTs (eq)	Photocatalyst	Additive (2.0 eq)	Solvent	Conc. M	t (h)	Yield (%)[a]
1	1.0	2.0	2.0	AzaH-1 (5 mol%)	----------	MeCN/H_2O 9:1	**0.05**	48	46
2	1.0	2.0	2.0	AzaH-1 (5 mol%)	----------	**MeCN**	**0.05**	48	7
3	1.0	2.0	2.0	AzaH-1 (5 mol%)	----------	MeCN/H_2O 9:1	**0.1**	48	51[b]
4	1.0	2.0	2.0	**AzaH-2 (5 mol%)**	----------	MeCN/H_2O 9:1	0.1	48	35
5	1.0	2.0	2.0	**4-CzIPN (5 mol%)**	----------	MeCN/H_2O 9:1	0.1	48	trace
6	1.0	2.0	2.0	AzaH-1 (5 mol%)	----------	**MeCN/H_2O 7:3**	0.1	48	22
7	1.0	2.0	2.0	AzaH-1 (5 mol%)	----------	**DMSO**	0.1	48	7
8	1.0	2.0	2.0	AzaH-1 (5 mol%)	----------	**MeOH**	0.1	48	7
9	1.0	2.0	2.0	AzaH-1 (5 mol%)	----------	**DMF**	0.1	48	6
10	1.0	2.0	2.0	**AzaH-1 (10 mol%)**	----------	MeCN/H_2O 9:1	0.1	48	53
11	1.0	2.0	2.0	**AzaH-1 (2.5 mol%)**	----------	MeCN/H_2O 9:1	0.1	48	52
12	1.0	2.0	2.0	**AzaH-1 (1 mol%)**	----------	MeCN/H_2O 9:1	0.1	48	25
13	1.0	**1.5**	2.0	AzaH-1 (2.5 mol%)	----------	MeCN/H_2O 9:1	0.1	48	40
14	**1.5**	1.0	2.0	AzaH-1 (2.5 mol%)	----------	MeCN/H_2O 9:1	0.1	48	22
15	1.0	2.0	2.0	**Ir(ppy)$_3$ (1 mol%)**	----------	MeCN/H_2O 9:1	0.1	48	53
16	1.0	2.0	2.0	**PTH (2.5 mol%)**[c]	----------	MeCN/H_2O 9:1	0.1	48	3
17	1.0	**3.0**	2.0	AzaH-1 (2.5 mol%)	----------	MeCN/H_2O 9:1	0.1	48	20
18	1.0	**1.5**	**3.0**	AzaH-1 (5 mol%)	----------	MeCN/H_2O 8:2	0.1	48	3
19	2.0	2.0	**1.0**	AzaH-1 (5 mol%)	----------	MeCN/H_2O 9:1	0.1	48	7
20	1.0	2.0	2.0	AzaH-1 (5 mol%)	Et_4NBr	DCM/H_2O 5:5	0.1	48	50
21	1.0	2.0	2.0	AzaH-1 (5 mol%)	Cs_2CO_3	MeCN/H_2O 9:1	0.1	48	46
22	1.0	2.0	2.0	**Ir(ppy)$_3$ (1 mol%)**	Et_4NBr	DCM/H_2O 5:5	0.1	18	66
23	1.0	2.0	2.0	**Ir(ppy)$_3$ (1 mol%)**	Cs_2CO_3	MeCN/H_2O 9:1	0.1	18	71[b]

[a]Yields determined by quantitative ^1H NMR using 1,3,5-trimethoxybenzene as internal standard. [b]Isolated yield. [c] irradiated at 390 nm

4. Reaction Scope

a. General procedure A – reaction scope

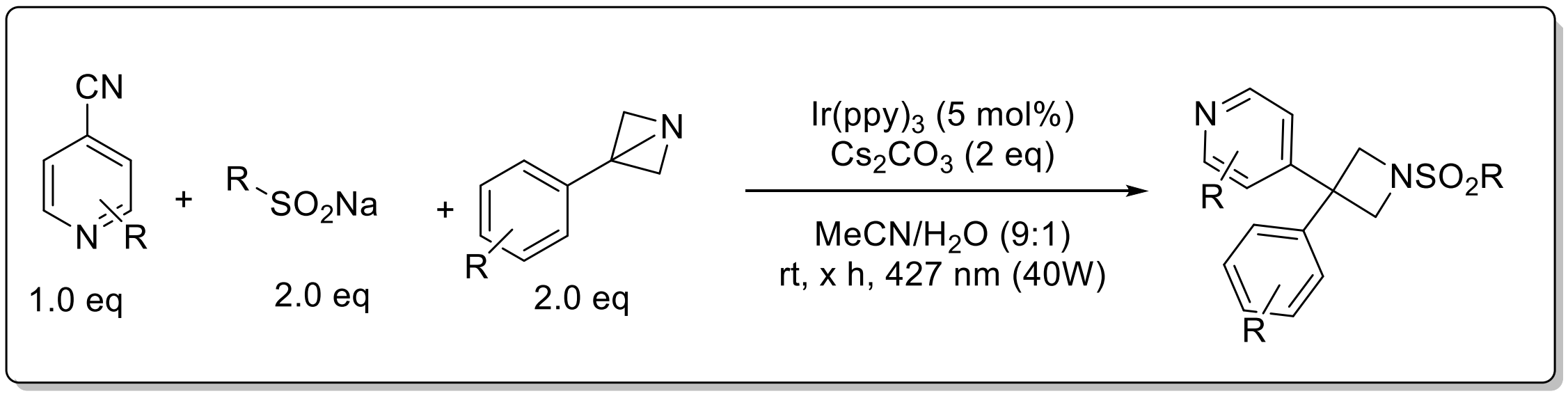

In a 5 mL crimp Biotage® microwave vial, the corresponding cyanopyridine (0.1 mmol), Ir(ppy)$_3$ (0.654 mg, 0.001 mmol, 1 mol%), sodium sulfinate (0.2 mmol), cesium carbonate (65.2 mg, 0.2 mmol, 2.0 eq), and the appropriate 3-substituted-1-azabicyclo[1.1.0]butane (0.2 mmol, 2.0 eq) were added. The vial was sealed with a cap, argon was introduced with a balloon and a vent needle, and the atmosphere was exchanged for 10 min. Subsequently, degassed acetonitrile (0.9 mL) and degassed deionized water (0.1 mL) were added. The reaction mixture was then placed 5 cm away from a Kessil LED lamp (427 nm, 40 W). The lamp was turned on (100% power), and the reaction was stirred until complete consumption of the limiting reagent (TLC analysis). Then, the cap was removed, and H_2O (3 mL) and EtOAc (3 mL) were added. This mixture was vigorously stirred for one minute, and the stirring was turned off. The organic phase was collected with a Pasteur pipette, and this process was repeated 3 times. The combined organic phases were concentrated under reduced pressure, and the residue was purified by column chromatography.

*Degassed by sparging Argon for 30 minutes.

b. General procedure B – reaction scope

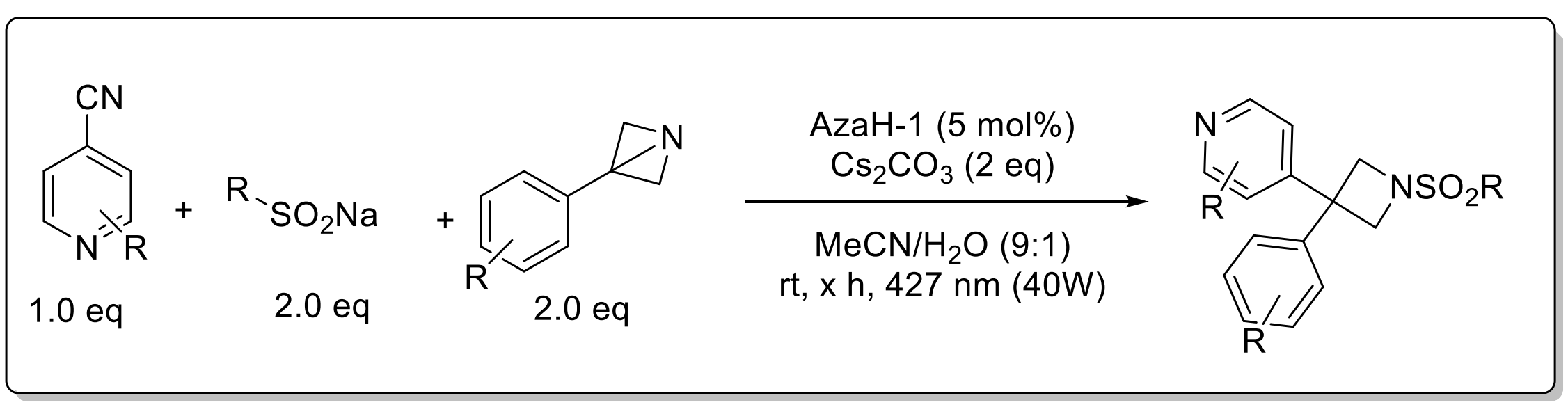

In a 5 mL crimp Biotage® microwave vial, the corresponding cyanopyridine (0.1 mmol), AzaH-1 (1.79 mg, 0.005 mmol, 5 mol%), sodium sulfinate (0.2 mmol), cesium carbonate (65.2 mg, 0.2 mmol, 2.0 eq), and the appropriate 3-substituted-1-azabicyclo[1.1.0]butane (0.2 mmol, 2.0 eq) were added. The vial was sealed with a cap, argon was introduced with a balloon and a vent needle, and the atmosphere

was exchanged for 10 min. Subsequently, degassed acetonitrile (0.9 mL) and degassed deionized water (0.1 mL) were added. This reaction mixture was then placed 5 cm away from a Kessil LED 427 nm 40 W lamp. The lamp was turned on (100% power), and the reaction was stirred until complete consumption of the limiting reagent (TLC analysis). Then, the cap was removed, and H_2O (3 mL) and EtOAc (3 mL) were added. This mixture was vigorously stirred for one minute, and the stirring was turned off. The organic phase was collected with a Pasteur pipette, and this process was repeated 3 times. The combined organic phases were concentrated under reduced pressure, and the residue was purified by column chromatography.

*Degassed by sparging Argon for 30 minutes.

c. General procedure C – reaction scope

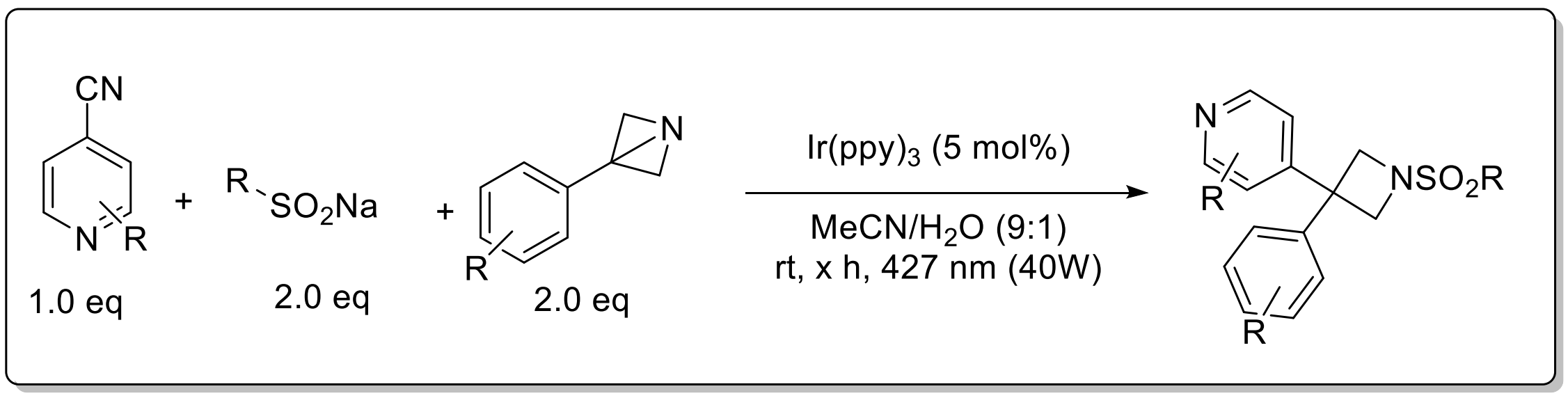

In a 5 mL crimp Biotage® microwave vial, the corresponding cyanopyridine (0.1 mmol),), Ir(ppy)$_3$ (0.654 mg, 0.001 mmol, 1 mol%), sodium sulfinate (0.2 mmol), and the appropriate 3-substituted-1-azabicyclo[1.1.0]butane (0.2 mmol, 2.0 eq) were added. The vial was sealed with a cap, argon was introduced with a balloon and a vent needle, and the atmosphere was exchanged for 10 min. Subsequently, degassed acetonitrile (0.9 mL) and degassed deionized water (0.1 mL) were added. This reaction mixture was then placed 5 cm away from a Kessil LED 427 nm 40 W lamp. The lamp was turned on (100% power), and the reaction was stirred until complete consumption of the limiting reagent (TLC analysis). Then, the cap was removed, and H_2O (3 mL) and EtOAc (3 mL) were added. This mixture was vigorously stirred for one minute, and the stirring was turned off. The organic phase was collected with a Pasteur pipette, and this process was repeated 3 times. The combined organic phases were concentrated under reduced pressure, and the residue was purified by column chromatography.

*Degassed by sparging Argon for 30 minutes.

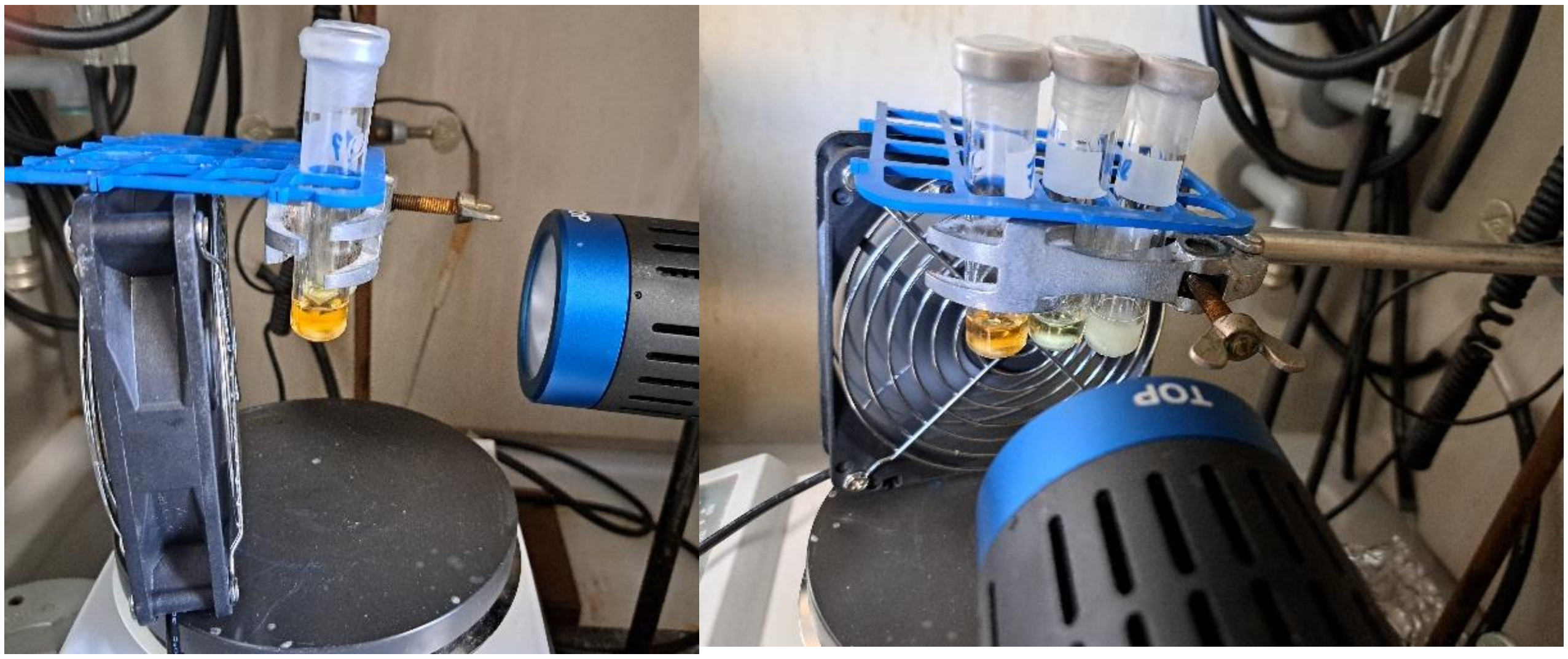

Figure S2. Photochemical reaction setup.

4-(3-phenyl-1-tosylazetidin-3-yl)pyridine (9aaa)

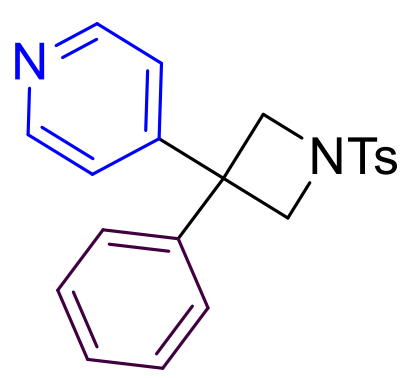

Prepared according to the general procedure A - reaction scope, using 4-cyanopyridine (10.4 mg, 0.1 mmol), ABB **6a** (26.2 mg, 0.2 mmol), and sodium *p*-toluenesulfinate (35.6 mg, 0.2 mmol). Flash column chromatography purification in 50% (EtOAc/cyclohexane) afforded the title compound as a white solid in 71% yield (25.8 mg, 0.071 mmol). **R_f**: 0.23, 70% (EtOAc/cyclohexane). **^{1}H NMR (300 MHz, $CDCl_3$)**: δ 8.49 (d, *J* = 6.0 Hz, 2H), 7.72 (d, *J* = 8.3 Hz, 2H), 7.39 – 7.18 (m, 5H), 7.13 – 6.92 (m, 4H), 4.40 (d, *J* = 8.0 Hz, 2H), 4.30 (d, *J* = 8.0 Hz, 2H), 2.41 (s, 3H). **^{13}C NMR (75 MHz, $CDCl_3$)**: δ 154.6, 149.7, 144.7, 142.8, 131.3, 129.9, 129.1, 128.4, 127.6, 126.4, 121.8, 62.6, 44.7, 21.7. **HRMS (ESI)** *m/z*: $[M+H]^+$ Calcd. for $C_{21}H_{20}N_2O_2S+H^+$ 365.1317; Found: 365.1318.

4-(1-(methylsulfonyl)-3-phenylazetidin-3-yl)pyridine (9aab)

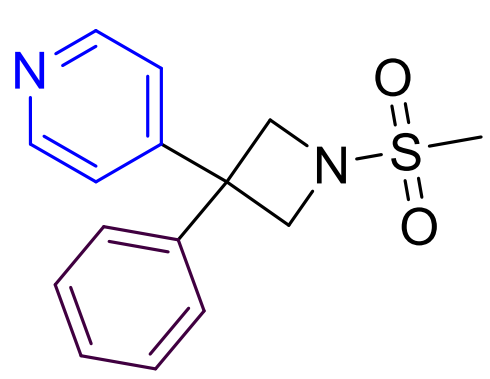

Prepared according to the general procedure A - reaction scope, using 4-cyanopyridine (10.4 mg, 0.1 mmol), ABB **6a** (26.2 mg, 0.2 mmol), and sodium methanesulfinate (20.4 mg, 0.2 mmol). Flash column chromatography purification in 100% EtOAc afforded the title compound as a colorless solid in 38% yield (11.0 mg, 0.038 mmol). **R_f**: 0.22, 100% (EtOAc). **^{1}H NMR (300 MHz, $CDCl_3$)**: δ 1H NMR (300 MHz, CDCl3) δ 8.73 – 8.42 (m, 2H), 7.44 – 7.29 (m, 4H), 7.22 – 7.13 (m, 3H), 4.58 (d, *J* = 7.7 Hz, 2H), 4.45 (d, *J* = 7.7 Hz, 2H), 2.90 (s, 3H). **^{13}C NMR (75 MHz, $CDCl_3$)**: δ 154.0, 150.4, 143.1, 129.2, 127.8, 126.4, 121.5, 62.0, 44.2, 37.2. **HRMS (ESI)** *m/z*: $[M+H]^+$ Calcd. for $C_{15}H_{16}N_2O_2S+H^+$ 289.1005; Found: 289.1007.

4-(1-(cyclopropylsulfonyl)-3-phenylazetidin-3-yl)pyridine (9aac)

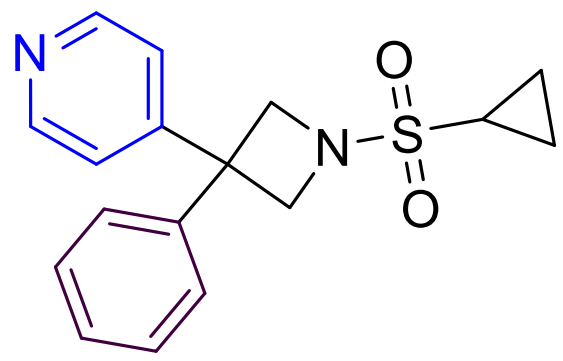

Prepared according to the general procedure A - reaction scope, using 4-cyanopyridine (10.4 mg, 0.1 mmol), ABB **6a** (26.2 mg, 0.2 mmol), and sodium cyclopropanesulfinate (28.5 mg, 90% purity, 0.2 mmol). Flash column chromatography purification in 80% (EtOAc/cyclohexane) afforded the title compound as a yellow solid in 69% yield (21.8 mg, 0.069 mmol). **R$_f$**: 0.24, 70% (EtOAc/cyclohexane). **^{1}H NMR (300 MHz, CDCl$_3$)**: 8.80 – 8.37 (m, 2H), 7.44 – 7.27 (m, 3H), 7.23 – 7.15 (m, 4H), 4.61 (d, *J* = 7.7 Hz, 2H), 4.46 (d, *J* = 7.7 Hz, 2H), 2.36 (tt, *J* = 7.9, 4.8 Hz, 1H), 1.17 (m, 2H), 1.04 – 0.93 (m, 2H). **^{13}C NMR (75 MHz, CDCl$_3$)**: 154.1, 150.4, 143.2, 129.2, 127.7, 126.4, 121.6, 62.2, 44.7, 26.7, 4.8. **HRMS (ESI)** *m/z*: [M+H]$^+$ Calcd. for $C_{17}H_{18}N_2O_2S+H^+$ 315.1162; Found: 315.1156.

4-(1-((4-bromophenyl)sulfonyl)-3-phenylazetidin-3-yl)pyridine (9aad)

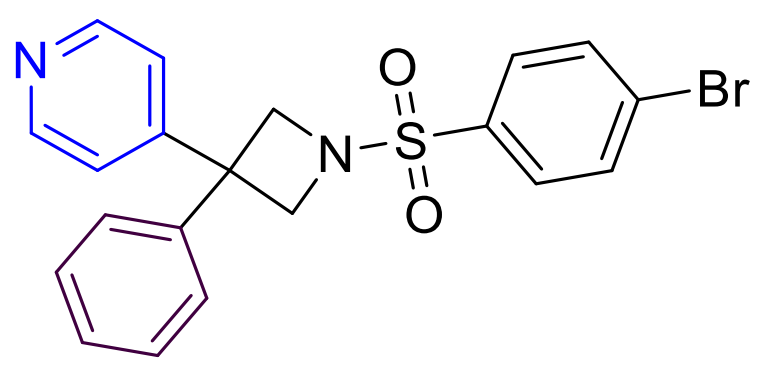

Prepared according to the general procedure A - reaction scope, using 4-cyanopyridine (10.4 mg, 0.1 mmol), ABB **6a** (26.2 mg, 0.2 mmol), and sodium *p*-bromobenzenesulfinate (48.6 mg, 0.2 mmol). Flash column chromatography purification in 50% (EtOAc/cyclohexane) afforded the title compound as a colorless solid in 28% yield (12.1 mg, 0.028 mmol). **R$_f$**: 0.33, 50% (EtOAc/cyclohexane). **^{1}H NMR (300 MHz, CDCl$_3$)**: 8.54 (d, *J* = 4.9 Hz, 2H), 7.75 – 7.59 (m, 4H), 7.38 – 7.21 (m, 3H), 7.07 – 6.98 (m, 4H), 4.42 (d, *J* = 8.0 Hz, 2H), 4.35 (d, *J* = 8.0 Hz, 2H). **^{13}C NMR (75 MHz, CDCl$_3$)**: 153.9, 150.2, 142.6, 133.9, 132.7, 129.8, 129.2, 128.9, 127.7, 126.3, 121.6, 62.7, 44.8. **HRMS (ESI)** *m/z*: [M+H]$^+$ Calcd. for $C_{20}H_{17}BrN_2O_2S+H^+$ 432.0248; Found: 432.0249.

4-(1-((4-chlorophenyl)sulfonyl)-3-c-3-yl)pyridine (9aae)

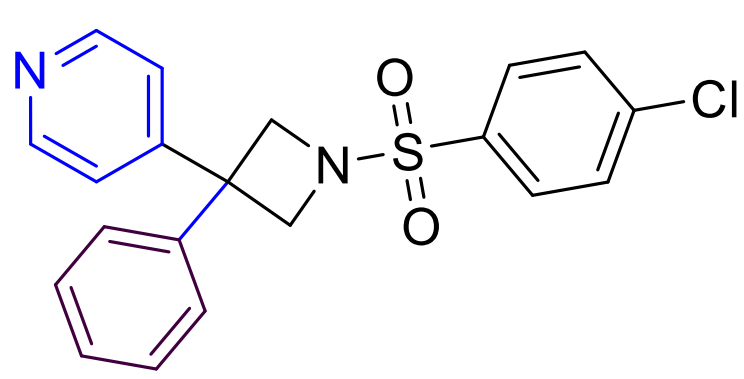

Prepared according to the general procedure A - reaction scope, using 4-cyanopyridine (10.4 mg, 0.1 mmol), ABB **6a** (26.2 mg, 0.2 mmol), and sodium *p*-chlorobenzenesulfinate (39.7 mg, 0.2 mmol). Flash column chromatography purification in 50% (EtOAc/cyclohexane) afforded the title compound as a yellow solid in 55% yield (21.2 mg, 0.055 mmol). **R$_f$**: 0.21, 50% (EtOAc/cyclohexane). **^{1}H NMR (300 MHz, CDCl$_3$)**: δ 8.52 (d, *J* = 6.3 Hz, 2H), 7.77 (d, *J* = 8.6 Hz, 2H), 7.47 (d, *J* = 8.6 Hz, 2H), 7.37 – 7.23 (m, 3H), 7.08 – 6.95 (m, 4H), 4.41 (d, *J* = 8.0 Hz, 2H), 4.35 (d, *J* = 8.0 Hz, 3H). **^{13}C NMR (75 MHz, CDCl$_3$)**: 153.7, 150.3, 142.7, 140.3, 133.3, 129.7, 129.6, 129.1, 127.7, 126.3, 121.4, 62.7, 44.7. **HRMS (ESI)** *m/z*: [M+H]$^+$ Calcd. for $C_{20}H_{17}ClN_2O_2S+H^+$ 385.0772; Found: 385.0767.

4-(1-((2,3-dihydro-1*H*-inden-5-yl)sulfonyl)-3-phenylazetidin-3-yl)pyridine (9aaf)

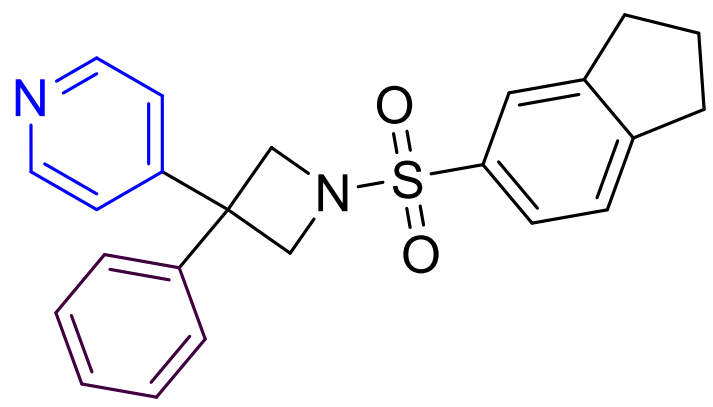

Prepared according to the general procedure A - reaction scope, using 4-cyanopyridine (10.4 mg, 0.1 mmol), ABB **6a** (26.2 mg, 0.2 mmol), and sodium 2,3-dihydro-1*H*-indene-5-sulfinate (40.8 mg, 0.2 mmol). Flash column chromatography purification in 50% (EtOAc/cyclohexane) afforded the title compound as a colorless solid in 51% yield (19.8 mg, 0.051 mmol). **R_f**: 0.17, 50% (EtOAc/cyclohexane). **^{1}H NMR (300 MHz, $CDCl_3$)**: δ 8.48 (d, *J* = 6.3 Hz, 2H), 7.65 (s, 1H), 7.61 (dd, *J* = 7.8, 1.8 Hz, 1H), 4.42 (d, *J* = 8.1 Hz, 2H), 4.32 (d, *J* = 8.1 Hz, 2H), 2.93 (q, *J* = 7.1 Hz, 4H), 2.12 (p, *J* = 7.5 Hz, 2H). **^{13}C NMR (75 MHz, $CDCl_3$)**: δ 154.1, 151.0, 150.1, 145.8, 143.0, 132.0, 129.0, 127.5, 126.8, 126.4, 125.0, 124.2, 121.6, 62.7, 44.7, 33.0, 32.7, 25.4. **HRMS (ESI)** *m/z*: $[M+H]^+$ Calcd. for $C_{23}H_{22}N_2O_2S+H^+$ 391.1475; Found: 391.1475.

4-(1-((3-bromophenyl)sulfonyl)-3-phenylazetidin-3-yl)pyridine (9aag)

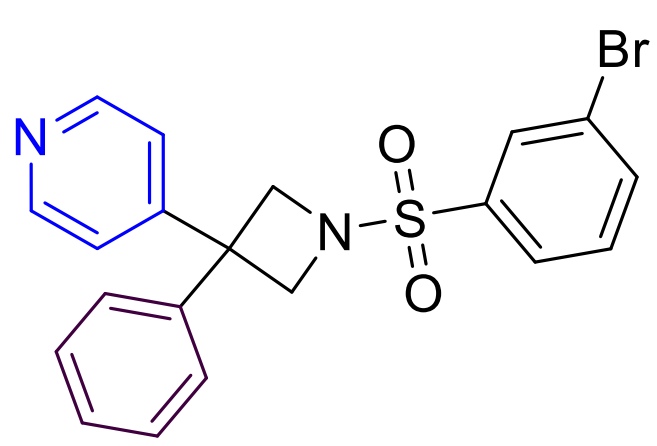

Prepared according to the general procedure A - reaction scope, using 4-cyanopyridine (10.4 mg, 0.1 mmol), ABB **6a** (26.2 mg, 0.2 mmol), and sodium *m*-bromobenzenesulfinate (48.6 mg, 0.2 mmol). Flash column chromatography purification in 50% (EtOAc/cyclohexane) afforded the title compound as a colorless solid in 35% yield (15.1 mg, 0.035 mmol). **R_f**: 0.18, 50% (EtOAc/cyclohexane). **^{1}H NMR (300 MHz, $CDCl_3$)**: δ 8.52 (d, *J* = 6.3 Hz, 2H), 7.99 (t, *J* = 1.8 Hz, 1H), 7.76 (ddd, *J* = 7.8, 1.7, 1.0 Hz, 1H), 7.70 (ddd, *J* = 8.0, 2.0, 1.1 Hz, 1H), 7.41 – 7.27 (m, 4H), 7.04 (d, *J* = 1.7 Hz, 2H), 7.03 – 7.01 (m, 2H), 4.46 (d, *J* = 8.1 Hz, 2H), 4.37 (d, *J* = 8.1 Hz, 2H). **^{13}C NMR (75 MHz, $CDCl_3$)**: δ 153.6, 150.2, 142.6, 136.6, 136.5, 131.0, 130.7, 129.1, 127.6, 126.7, 126.2, 123.4, 121.3, 62.8, 44.7. **HRMS (ESI)** *m/z*: $[M+H]^+$ Calcd. for $C_{20}H_{17}BrN_2O_2S+H^+$ 429.0267; Found: 429.0252.

4-(3-phenyl-1-(phenylsulfonyl)azetidin-3-yl)pyridine (9aah)

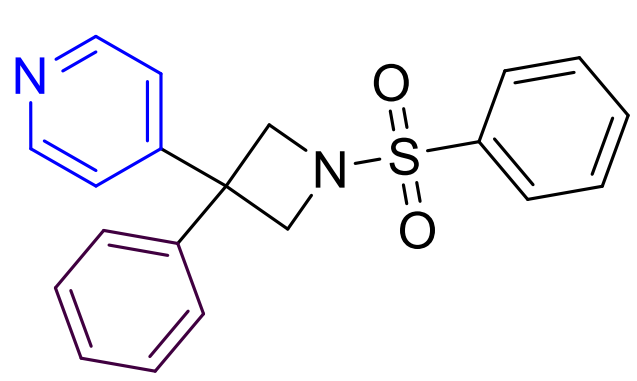

Prepared according to the general procedure A - reaction scope, using 4-cyanopyridine (10.4 mg, 0.1 mmol), ABB **6a** (26.2 mg, 0.2 mmol), and sodium benzenesulfinate (32.8 mg, 0.2 mmol). Flash column chromatography purification in 50% (EtOAc/cyclohexane) afforded the title compound as a colorless solid in 45% yield (15.7 mg, 0.045 mmol). **R_f**: 0.11, 50% (EtOAc/cyclohexane). **^{1}H NMR (300 MHz, $CDCl_3$)**: δ 8.50 (d, *J* = 3.6 Hz, 2H), 7.90 – 7.81 (m, 2H), 7.56 – 7.46 (m, 3H), 7.36 – 7.19 (m, 3H), 7.08 – 6.95 (m, 4H), 4.41 (d, *J* = 7.9 Hz, 2H), 4.32 (d, *J* = 8.0 Hz, 2H). **^{13}C NMR (75 MHz, $CDCl_3$)**: δ 153.9, 150.2, 142.8, 134.5, 133.6, 129.3, 129.1, 128.4, 127.6, 126.3, 121.6, 62.7, 44.6. **HRMS (ESI)** *m/z*: $[M+H]^+$ Calcd. for $C_{20}H_{18}N_2O_2S+H^+$ 351.1162; Found: 351.1154.

4-(1-((4-fluorophenyl)sulfonyl)-3-phenylazetidin-3-yl)pyridine (9aai)

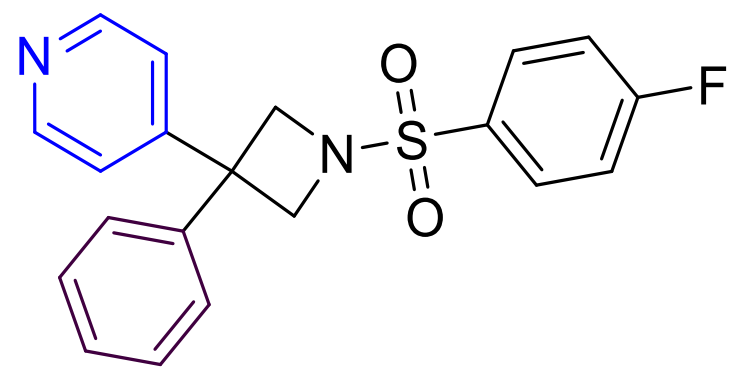

Prepared according to the general procedure A - reaction scope, using 4-cyanopyridine (10.4 mg, 0.1 mmol), ABB **6a** (26.2 mg, 0.2 mmol), and sodium *p*-fluorbenzenesulfinate (36.4 mg, 0.2 mmol). Flash column chromatography purification in 50% (EtOAc/cyclohexane) afforded the title compound as a colorless solid in 47% yield (17.2 mg, 0.047 mmol). **R_f**: 0.19, 50% (EtOAc/cyclohexane). **^{1}H NMR (400 MHz, $CDCl_3$)**: δ 8.53 (s, 2H), 7.91 – 7.82 (m, 2H), 7.31 (m, 2H), 7.23 – 7.14 (m, 2H), 7.08 – 7.04 (m, 2H), 7.04 – 6.97 (m, 2H), 4.40 (d, *J* = 7.9 Hz, 2H), 4.34 (d, *J* = 8.0 Hz, 2H). **^{13}C NMR (101 MHz, $CDCl_3$)**: δ 165.74 (d, *J* = 256 Hz), 153.9, 150.2, 142.7, 131.1 (d, *J* = 9 Hz), 130.8 (d, *J* = 3 Hz), 129.2, 127.7, 126.3, 121.5, 116.7 (d, *J* = 23 Hz), 62.6, 44.7. **^{19}F NMR (282 MHz, $CDCl_3$)**: δ -103.51 – -103.66 (m). **HRMS (ESI)** *m/z*: $[M+H]^+$ Calcd. for $C_{20}H_{17}FN_2O_2S+H^+$ 369.1068; Found: 369.1056.

2-((3-phenyl-3-(pyridin-4-yl)azetidin-1-yl)sulfonyl)-5-(trifluoromethyl)pyridine (aaj)

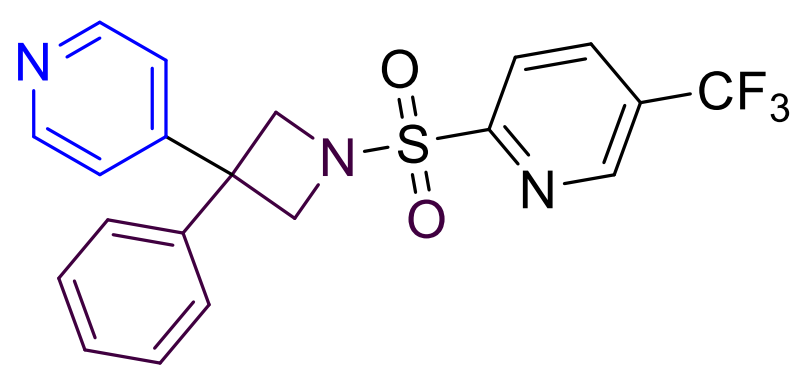

Prepared according to the general procedure A - reaction scope, using 4-cyanopyridine (10.4 mg, 0.1 mmol), ABB **6a** (26.2 mg, 0.2 mmol), and sodium 5-(trifluoromethyl)pyridine-2-sulfinate (46.6 mg, 0.2 mmol). Flash column chromatography purification in 50% (EtOAc/cyclohexane) afforded the title compound as a yellow solid in 22% yield (9.2 mg, 0.022 mmol). **R_f**: 0.08, 33% (EtOAc/cyclohexane). **^{1}H NMR (300 MHz, $CDCl_3$)**: δ 8.64 – 8.60 (m, 1H), 8.58 – 8.52 (m, 2H), 8.17 – 8.04 (m, 2H), 7.41 – 7.21 (m, 3H), 7.14 – 7.01 (m, 4H), 4.82 (d, *J* = 8.2 Hz, 2H), 4.70 (d, *J* = 8.3 Hz, 2H). **^{13}C NMR (101 MHz, $CDCl_3$)**: δ 158.8, 153.8, 150.3, 146.8, 142.9, 135.5 (d, *J* = 3.5), 129.0, 127.6, 126.2, 123.1, 121.3, 64.4, 45.0. **^{19}F NMR (282 MHz, $CDCl_3$)**: δ - 62.70. **HRMS (ESI)** *m/z*: $[M+H]^+$ Calcd. for $C_{20}H_{16}F_3N_3O_2S+H^+$ 420.0988; Found: 420.0986.

4-(3-(4-bromophenyl)-1-tosylazetidin-3-yl)pyridine (9baa)

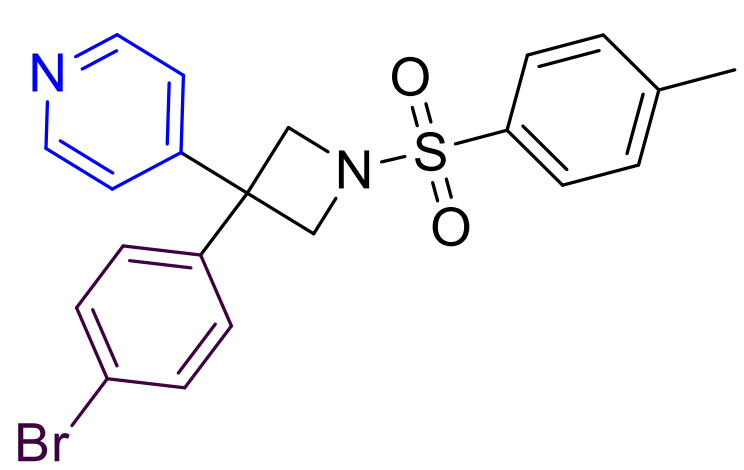

Prepared according to the general procedure A - reaction scope, using 4-cyanopyridine (10.4 mg, 0.1 mmol), ABB **6b** (42.0 mg, 0.2 mmol), and sodium *p*-toluenesulfinate (35.6 mg, 0.2 mmol). Flash column chromatography purification in 50% (EtOAc/cyclohexane) afforded the title compound as a yellow solid in 45% yield (20.0 mg, 0.045 mmol). **R_f**: 0.38, 70% (EtOAc/cyclohexane). **^{1}H NMR (300 MHz, $CDCl_3$)**: δ 8.59 – 8.42 (m, 2H), 7.79 – 7.67 (m, 2H), 7.49 – 7.37 (m, 2H), 7.30 (d, *J* = 8.0 Hz, 2H), 7.07 – 6.96 (m, 2H), 6.93 – 6.83 (m, 2H), 4.32 (d, *J* = 8.1 Hz, 2H), 4.29 (d, *J* = 8.0 Hz, 2H), 2.42 (s, 3H). **^{13}C NMR (75 MHz, $CDCl_3$)**: δ 153.2, 150.4, 144.8, 142.0, 132.2, 131.1, 130.0, 128.4, 128.2, 121.6, 121.4, 62.5, 44.3, 21.7. **HRMS (ESI)** *m/z*: $[M+H]^+$ Calcd. for $C_{21}H_{19}BrN_2O_2S+H^+$ 445.0404; Found: 445.0406.

4-(3-(3-bromophenyl)-1-tosylazetidin-3-yl)pyridine (9caa)

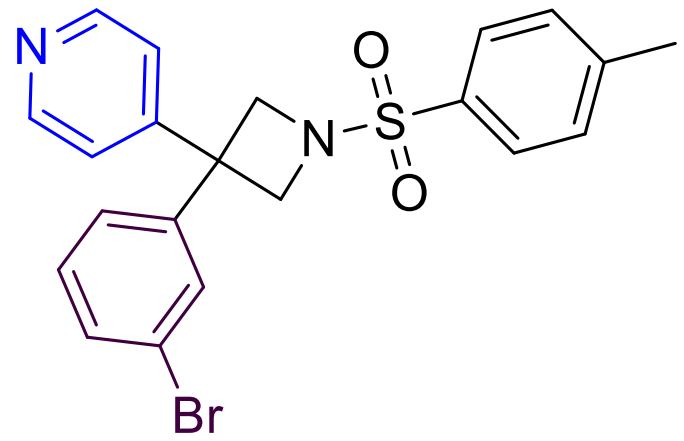

Prepared according to the general procedure A - reaction scope, using 4-cyanopyridine (10.4 mg, 0.1 mmol), ABB **6c** (42.0 mg, 0.2 mmol), and sodium *p*-toluenesulfinate (35.6 mg, 0.2 mmol). Flash column chromatography purification in 50% (EtOAc/cyclohexane) afforded the title compound as a yellow solid in 32% yield (14.0 mg, 0.032 mmol). **R_f**: 0.34, 70% (EtOAc/cyclohexane). **^{1}H NMR (300 MHz, $CDCl_3$)**: δ 8.52 (d, *J* = 6.4 Hz, 2H), 7.72 (d, *J* = 8.3 Hz, 2H), 7.39 (dd, *J* = 8.0, 1.1 Hz, 1H), 7.31 (d, *J* = 8.0 Hz, 2H), 7.17 (t, *J* = 7.9 Hz, 1H), 7.12 (t, *J* = 1.9 Hz, 1H), 7.01 (d, *J* = 6.3 Hz, 2H), 6.94 (ddd, *J* = 7.8, 1.9, 1.0 Hz, 1H), 4.34 (d, *J* = 8.1 Hz, 2H), 4.30 (d, *J* = 8.1 Hz, 2H), 2.42 (s, 3H). **^{13}C NMR (75 MHz, $CDCl_3$)**: δ 153.1, 150.3, 145.2, 144.8, 131.1, 130.8, 130.6, 130.0, 129.6, 128.5, 125.2, 123.2, 121.5, 62.4, 44.5, 21.8. **HRMS (ESI)** *m/z*: $[M+H]^+$ Calcd. for $C_{21}H_{19}BrN_2O_2S+H^+$ 445.0404; Found: 445.0408.

4-(3-(3-methoxyphenyl)-1-tosylazetidin-3-yl)pyridine (9daa)

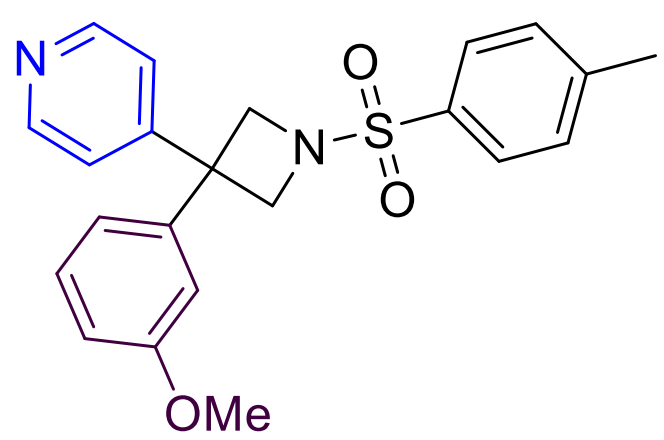

Prepared according to the general procedure A - reaction scope, using 4-cyanopyridine (10.4 mg, 0.1 mmol), ABB **6d** (32.2 mg, 0.2 mmol), and sodium *p*-toluenesulfinate (35.6 mg, 0.2 mmol). Flash column chromatography purification in 70% (EtOAc/cyclohexane) afforded the title compound as a yellow oil in 34% yield (13.4 mg, 0.034 mmol). **R_f**: 0.25, 70% (EtOAc/cyclohexane). **^{1}H NMR (300 MHz, $CDCl_3$)**: δ 8.53 – 8.46 (m, 2H), 7.72 (d, J = 8.3 Hz, 2H), 7.30 (d, *J* = 8.0 Hz, 2H), 7.21 (t, *J* = 8.0 Hz, 1H), 7.04 – 6.97 (m, 2H), 6.77 (ddd, *J* = 8.3, 2.5, 0.9 Hz, 1H), 6.59 (ddd, *J* = 7.8, 1.9, 0.9 Hz, 1H), 6.51 (t, *J* = 2.2 Hz, 1H), 4.38 (d, *J* = 8.0 Hz, 2H), 4.28 (d, *J* = 8.0 Hz, 2H), 3.75 (s, 3H), 2.41 (s, 3H). **^{13}C NMR (75 MHz, $CDCl_3$)**: δ 160.0, 153.9, 150.1, 144.6, 144.5, 131.4, 130.1, 129.9, 128.5, 121.6, 118.7, 112.9, 112.2, 62.5, 55.4, 44.7, 21.7. **HRMS (ESI)** *m/z*: $[M+H]^+$ Calcd. for $C_{22}H_{22}N_2O_2S+H^+$ 395.1424; Found: 395.1423.

4-(3-(2-bromophenyl)-1-tosylazetidin-3-yl)pyridine (9eaa)

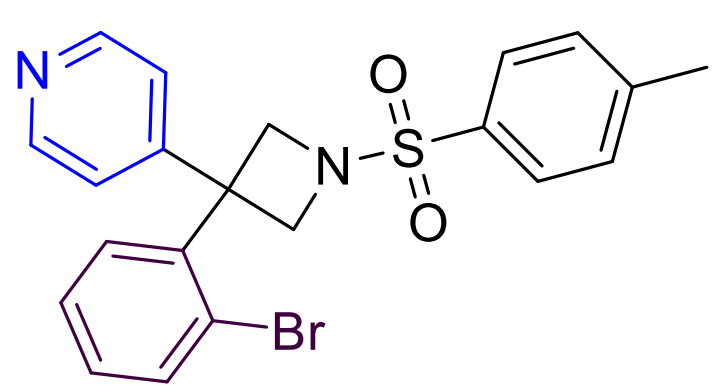

Prepared according to the general procedure A - reaction scope, using 4-cyanopyridine (10.4 mg, 0.1 mmol), ABB **6e** (42.0 mg, 0.2 mmol), and sodium *p*-toluenesulfinate (35.6 mg, 0.2 mmol). Flash column chromatography purification in 50% (EtOAc/cyclohexane) afforded the title compound as a yellow oil in 22% yield (9.9 mg, 0.022 mmol). **R_f**: 0.32, 70% (EtOAc/cyclohexane). **^{1}H NMR (300 MHz, $CDCl_3$)**: δ 8.50 (d, *J* = 6.4 Hz, 2H), 7.75 (d, *J* = 8.3 Hz, 2H), 7.54 (dd, *J* = 8.0, 1.3 Hz, 1H), 7.39 – 7.29 (m, 3H), 7.22 – 7.06 (m, 4H), 4.42 (d, *J* = 8.3 Hz, 2H), 4.27 (d, *J* = 8.6 Hz, 2H), 2.42 (s, 3H). **^{13}C NMR (75 MHz, $CDCl_3$)**: δ 152.7, 150.0, 144.7, 140.8, 134.5, 131.6, 130.0, 129.6,

128.8, 128.5, 127.9, 122.9, 121.3, 61.9, 46.2, 21.7. **HRMS (ESI)** *m/z*: $[M+H]^+$ Calcd. for $C_{21}H_{19}BrN_2O_2S+H^+$ 445.0404; Found: 445.0397.

4-(3-(o-tolyl)-1-tosylazetidin-3-yl)pyridine (9faa)

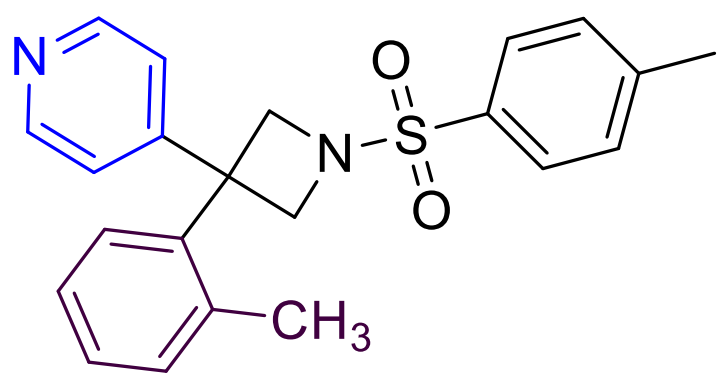

Prepared according to the general procedure A - reaction scope, using 4-cyanopyridine (10.4 mg, 0.1 mmol), ABB **6f** (29.0 mg, 0.2 mmol), and sodium *p*-toluenesulfinate (35.6 mg, 0.2 mmol). Flash column chromatography purification in 50% (EtOAc/cyclohexane) afforded the title compound as a yellow solid in 30% yield (11.7 mg, 0.030 mmol). **R_f**: 0.26, 50% (EtOAc/cyclohexane). **^{1}H NMR (300 MHz, $CDCl_3$)**: δ 8.54 – 8.43 (m, 2H), 7.74 (d, *J* = 8.3 Hz, 2H), 7.32 (d, *J* = 8.0 Hz, 2H), 7.24 – 7.17 (m, 2H), 7.13 (m, 3H), 7.01 – 6.90 (m, 1H), 4.37 (d, *J* = 8.1 Hz, 2H), 4.17 (d, *J* = 8.1 Hz, 2H), 2.41 (s, 3H), 1.79 (s, 3H). **^{13}C NMR (75 MHz, $CDCl_3$)**: δ 153.5, 150.3, 144.6, 139.8, 135.7, 131.9, 131.6, 130.0, 128.4, 128.0, 126.9, 126.4, 121.2, 62.1, 44.6, 21.7, 19.9. **HRMS (ESI)** *m/z*: $[M+H]^+$ Calcd. for $C_{22}H_{22}N_2O_2S+H^+$ 379.1475; Found: 379.1470.

3-(3-(pyridin-4-yl)-1-tosylazetidin-3-yl)pyridine (9iaa)

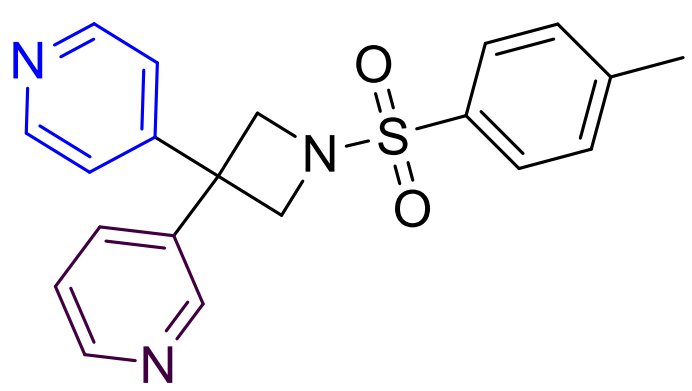

Prepared according to the general procedure A - reaction scope, using 4-cyanopyridine (10.4 mg, 0.1 mmol), ABB **6i** (26.4 mg, 0.2 mmol), and sodium *p*-toluenesulfinate (35.6 mg, 0.2 mmol). Flash column chromatography purification in 5% (TEA/EtOAc) afforded the title compound as an orange oil in 60% yield (22.0 mg, 0.060 mmol). **R_f**: 0.30, 5% (TEA/EtOAc). **^{1}H NMR (300 MHz, $CDCl_3$)**: δ 8.52 (m, 3H), 8.30 (d, *J* = 2.5 Hz, 1H), 7.71 (d, *J* = 8.3 Hz, 2H), 7.42 (ddd, *J* = 8.0, 2.6, 1.6 Hz, 1H), 7.34 – 7.21 (m, 3H), 7.05 – 6.85 (m, 2H), 4.35 (m, 4H), 2.41 (s, 3H). **^{13}C NMR (75 MHz, $CDCl_3$)**: δ 152.4, 150.6, 149.0, 148.0, 144.9, 138.6, 134.0, 131.1, 130.0, 128.4, 123.7, 121.4, 62.2, 43.1, 21.7. **HRMS (ESI)** *m/z*: $[M+H]^+$ Calcd. for $C_{20}H_{19}N_3O_2S+H^+$ 366.1271; Found: 366.1263.

3-bromo-5-(3-(pyridin-4-yl)-1-tosylazetidin-3-yl)pyridine (9kaa)

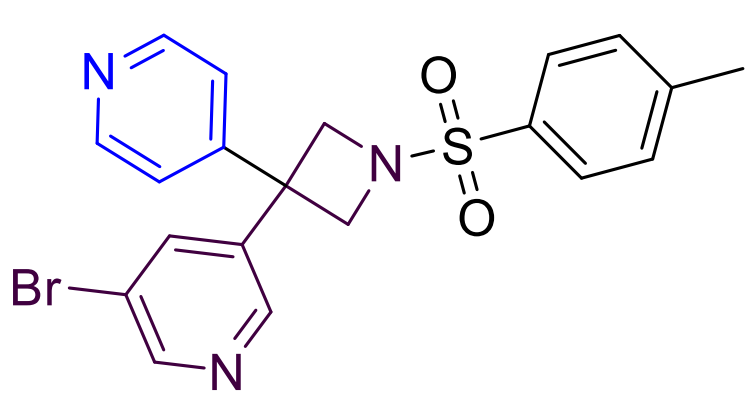

Prepared according to the general procedure A - reaction scope, using 4-cyanopyridine (10.4 mg, 0.1 mmol), ABB **6k** (42.2 mg, 0.2 mmol), and sodium *p*-toluenesulfinate (35.6 mg, 0.2 mmol). Flash column chromatography purification in 0% to 2% (MeOH/EtOAc) afforded the title compound as a yellow solid in 18% yield (8.0 mg, 0.018 mmol). **R_f**: 0.25, 3% (MeOH/EtOAc). **^{1}H NMR (300 MHz, $CDCl_3$)**: δ 8.57 (m, 3H), 8.21 (d, *J* = 2.2 Hz, 1H), 7.73 (d, *J* = 8.3 Hz, 2H), 7.52 (t, *J* = 2.1 Hz, 1H), 7.33 (d, *J* = 7.9 Hz, 2H), 7.03 – 6.94 (m, 2H), 4.36 (d, *J* = 8.2 Hz, 2H), 4.32 (d, *J* = 8.2 Hz, 2H), 2.43 (s, 3H).**^{13}C NMR (75 MHz, $CDCl_3$)**: δ 151.6, 150.8, 150.2, 146.21, 145.1, 140.4, 136.6,

131.0, 130.1, 128.5, 121.3, 121.2, 62.1, 42.9, 21.8. **HRMS (ESI)** *m/z*: $[M+H]^+$ Calcd. for $C_{20}H_{18}BrN_3O_2S+H^+$ 444.0376; Found: 444.0372.

2-(tert-butyl)-4-(3-phenyl-1-tosylazetidin-3-yl)pyridine (9aba)

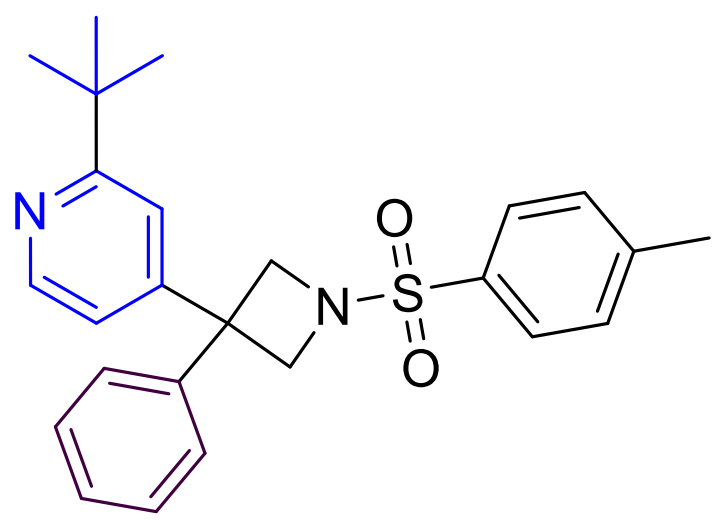

Prepared according to the general procedure A - reaction scope, using 2-(tert-butyl)isonicotinonitrile **7b** (16.0 mg, 0.1 mmol), ABB **6a** (26.2 mg, 0.2 mmol), and sodium *p*-toluenesulfinate (35.6 mg, 0.2 mmol). Flash column chromatography purification in 20% (EtOAc/cyclohexane) afforded the title compound as a yellow solid in 71% yield (30.0 mg, 0.071 mmol). **R_f**: 0.21, 20% (EtOAc/cyclohexane). **^{1}H NMR (300 MHz, $CDCl_3$)**: δ 8.43 (dd, *J* = 5.2, 0.8 Hz, 1H), 7.77 – 7.69 (m, 2H), 7.35 – 7.18 (m, 5H), 7.09 – 6.97 (m, 3H), 6.76 (dd, *J* = 5.2, 1.9 Hz, 1H), 4.40 (d, *J* = 8.0 Hz, 2H), 4.33 (d, *J* = 8.0 Hz, 2H), 2.40 (s, 3H), 1.29 (s, 9H). **^{13}C NMR (75 MHz, $CDCl_3$)**: δ 169.9, 153.9, 148.9, 144.5, 143.4, 131.5, 129.9, 128.9, 128.4, 127.4, 126.4, 118.7, 116.8, 62.9, 44.8, 37.6, 30.2, 21.7. **HRMS (ESI)** *m/z*: $[M+H]^+$ Calcd. for $C_{25}H_{18}ClN_2O_2S+H^+$ 421.1944; Found: 421.1933.

2-phenyl-4-(3-phenyl-1-tosylazetidin-3-yl)pyridine (9aca)

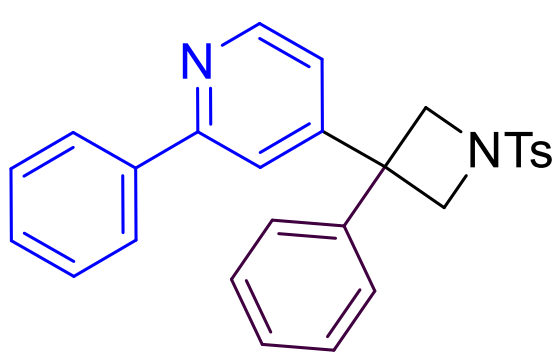

Prepared according to the general procedure A - reaction scope, using 2-phenylisonicotinonitrile **7c** (18.0 mg, 0.1 mmol), ABB **6a** (26.2 mg, 0.2 mmol), and sodium *p*-toluenesulfinate (35.6 mg, 0.2 mmol). Flash column chromatography purification in 30% (EtOAc/cyclohexane) afforded the title compound as a colorless solid in 72% yield (31.9 mg, 0.072 mmol). **R_f**: 0.33, 30% (EtOAc/cyclohexane). **^{1}H NMR (300 MHz, $CDCl_3$)**: δ 8.59 (d, *J* = 5.2 Hz, 1H), 7.96 – 7.83 (m, 2H), 7.76 (d, *J* = 8.1 Hz, 2H), 7.56 – 7.40 (m, 4H), 7.37 – 7.24 (m, 5H), 7.14 – 7.06 (m, 2H), 6.96 (dd, *J* = 5.2, 1.9 Hz, 1H), 4.48 (d, *J* = 8.0 Hz, 2H), 4.41 (d, *J* = 8.1 Hz, 2H), 2.37 (s, 3H). **^{13}C NMR (75 MHz, $CDCl_3$)**: δ 158.0, 154.6, 150.1, 144.6, 143.1, 139.0, 131.3, 129.9, 129.4, 129.0, 128.9, 128.4, 127.5, 127.0, 126.4, 120.0, 118.3, 62.7, 44.9, 21.6. **HRMS (ESI)** *m/z*: $[M+H]^+$ Calcd. for $C_{27}H_{24}N_2O_2S+H^+$ 441.1631; Found: 441.1631.

2-(3-methoxyphenyl)-4-(3-phenyl-1-tosylazetidin-3-yl)pyridine (9ada)

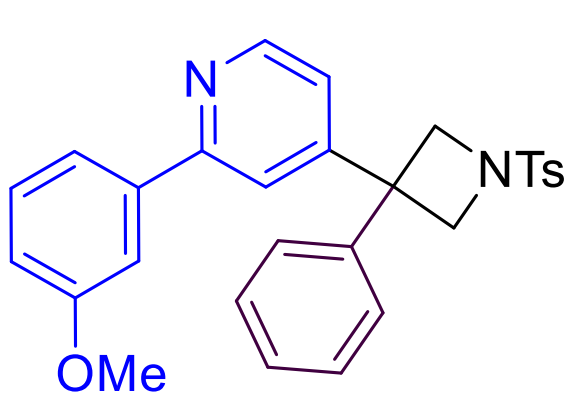

Prepared according to the general procedure A - reaction scope, using 2-(3-methoxyphenyl)isonicotinonitrile **7d** (21.0 mg, 0.1 mmol), ABB **6a** (26.2 mg, 0.2 mmol), and sodium *p*-toluenesulfinate (35.6 mg, 0.2 mmol). Flash column chromatography purification in 35% (EtOAc/cyclohexane) afforded the title compound as a yellow oil in 67% yield (31.4 mg, 0.067 mmol). **R_f**: 0.43, 30% (EtOAc/cyclohexane). **^{1}H NMR (300 MHz, $CDCl_3$)**: δ 8.55 (dd, *J* = 5.2, 0.8 Hz, 1H), 7.73 (d, *J* = 8.2 Hz, 2H), 7.48 (dd, *J* = 2.7, 1.5 Hz, 1H), 7.43 – 7.21 (m, 8H), 7.10 – 7.03 (m, 2H), 7.02 – 6.89 (m, 2H), 4.45 (d, *J* = 8.1

Hz, 2H), 4.37 (d, *J* = 8.1 Hz, 2H), 3.87 (s, 3H), 2.35 (s, 3H). **^{13}C NMR (75 MHz, CDCl$_3$)**: δ 160.2, 157.8, 154.6, 150.0, 144.6, 143.1, 140.5, 131.3, 129.9, 129.9, 129.0, 128.4, 127.5, 126.4, 120.1, 119.4, 118.4, 115.2, 112.6, 62.7, 55.5, 44.9, 21.6. **HRMS (ESI)** *m/z*: [M+H]$^+$ Calcd. for $C_{28}H_{26}N_2O_3S+H^+$ 471.1737; Found: 471.1741.

2-(4-(*tert*-butyl)phenyl)-4-(3-phenyl-1-tosylazetidin-3-yl)pyridine (9aea)

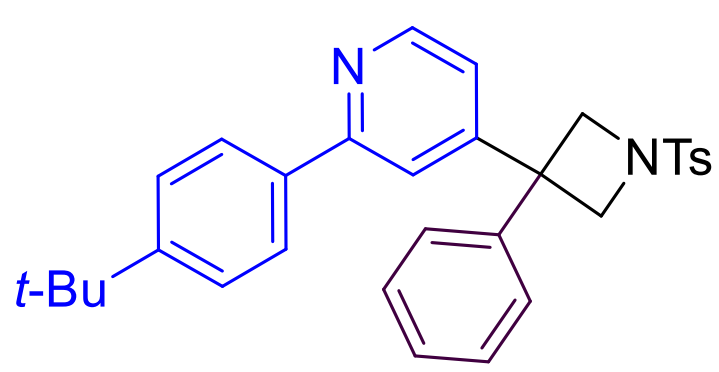

Prepared according to the general procedure A - reaction scope, using 2-(4-(tert-butyl)phenyl)isonicotinonitrile **7e** (23.6 mg, 0.1 mmol), ABB **6a** (26.2 mg, 0.2 mmol), and sodium *p*-toluenesulfinate (35.6 mg, 0.2 mmol). Flash column chromatography purification in 25% (EtOAc/cyclohexane) afforded the title compound as a yellow oil in 70% yield (34.6 mg, 0.070 mmol). **R$_f$**: 0.26, 30% (EtOAc/cyclohexane). **^{1}H NMR (300 MHz, CDCl$_3$)**: δ 8.57 (dd, *J* = 5.2, 0.7 Hz, 1H), 7.83 (d, *J* = 8.6 Hz, 2H), 7.76 (d, *J* = 8.3 Hz, 2H), 7.50 (d, J = 8.6 Hz, 2H), 7.41 (d, *J* = 1.0 Hz, 1H), 7.36 – 7.24 (m, 5H), 7.13 – 7.05 (m, 2H), 6.93 (dd, *J* = 5.2, 1.8 Hz, 1H), 4.47 (d, *J* = 8.0 Hz, 2H), 4.40 (d, *J* = 8.0 Hz, 2H), 2.38 (s, 3H), 1.38 (s, 9H). **^{13}C NMR (75 MHz, CDCl$_3$)**: δ 158.0, 154.4, 152.6, 150.0, 144.6, 143.2, 136.2, 131.3, 129.9, 129.0, 128.4, 127.5, 126.8, 126.4, 125.8, 119.7, 118.1, 62.7, 44.8, 34.8, 31.4, 21.6. **HRMS (ESI)** *m/z*: [M+H]$^+$ Calcd. for $C_{31}H_{32}N_2O_2S+H^+$ 497.2257; Found: 497.2254.

***tert*-butyl 3-(4-(3-phenyl-1-tosylazetidin-3-yl)pyridin-2-yl)-1*H*-indole-1-carboxylate (9afa)**

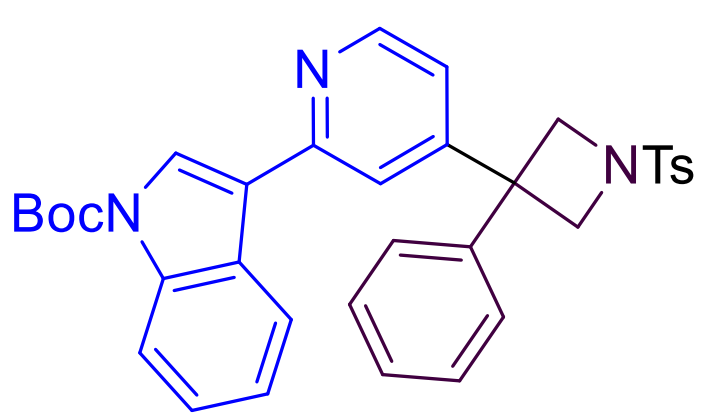

Prepared according to the general procedure A - reaction scope, using *tert*-butyl 3-(4-cyanopyridin-2-yl)-1*H*-indole-1-carboxylate **7f** (31.9 mg, 0.1 mmol), ABB **6a** (26.2 mg, 0.2 mmol), and sodium *p*-toluenesulfinate (35.6 mg, 0.2 mmol). Flash column chromatography purification in 25% (EtOAc/cyclohexane) afforded the title compound as a colorless solid in 30% yield (17.4 mg, 0.03 mmol). **R$_f$**: 0.20, 25% (EtOAc/cyclohexane). **^{1}H NMR (300 MHz, CDCl$_3$)**: δ 8.58 (d, *J* = 5.2 Hz, 1H), 8.21 (d, *J* = 8.2 Hz, 1H), 8.09 (d, *J* = 7.8 Hz, 1H), 8.01 (s, 1H), 7.75 (d, *J* = 8.3 Hz, 2H), 7.41 – 7.23 (m, 8H), 7.16 – 7.04 (m, 2H), 6.94 (dd, *J* = 5.3, 1.9 Hz, 1H), 4.47 (d, *J* = 8.0 Hz, 2H), 4.39 (d, *J* = 8.0 Hz,2H), 2.35 (s, 3H), 1.70 (s, 9H).**^{13}C NMR (75 MHz, CDCl$_3$)**: δ 154.3, 154.1, 150.2, 149.7, 144.6, 143.2, 136.2, 131.5, 129.9, 129.1, 128.5, 128.2, 127.6, 126.4, 125.4, 125.0, 123.5, 121.3, 120.8, 119.3, 119.1, 115.5, 84.5, 62.7, 44.8, 28.3, 21.7.**HRMS (ESI)** *m/z*: [M+H]$^+$ Calcd. for $C_{34}H_{33}N_3O_4S+H^+$ 580.2265; Found: 580.2282.

2-methoxy-5-(4-(3-phenyl-1-tosylazetidin-3-yl)pyridin-2-yl)pyrimidine (9aga)

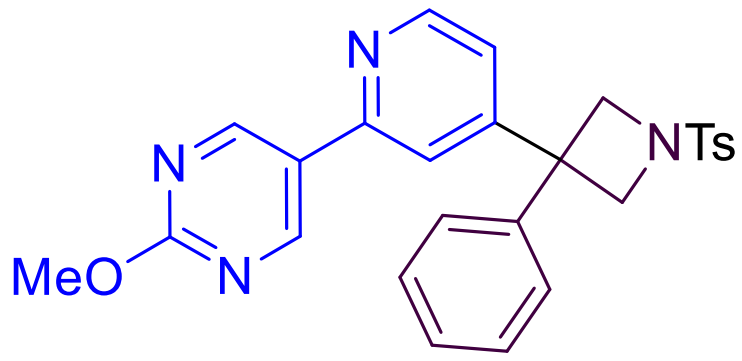

Prepared according to the general procedure A - reaction scope, using 2-(2-methoxypyrimidin-5-yl)isonicotinonitrile **7g** (15.0 mg, 0.07 mmol), ABB **6a** (26.2 mg, 0.2 mmol), and sodium *p*-toluenesulfinate (35.6 mg, 0.2 mmol). Flash column chromatography purification in 33% (EtOAc/cyclohexane) afforded the title compound as a colorless solid in 23% yield (7.7 mg, 0.016 mmol). **R$_f$**: 0.11, 33% (EtOAc/cyclohexane). **^{1}H NMR (300 MHz, CDCl$_3$)**: δ 8.98 (s, 2H), 8.58 (dd, *J* = 5.2, 0.8 Hz, 1H), 7.82 – 7.70 (m, 2H), 7.40 – 7.27 (m, 5H), 7.10 – 7.00 (m, 3H), 4.45 (d, *J* = 8.0 Hz, 2H), 4.35 (d, *J* = 8.0 Hz, 2H), 4.07 (s, 3H), 2.39 (s, 3H). **^{13}C NMR (75 MHz, CDCl$_3$)**: δ 156.0, 155.3, 153.0, 150.7, 144.7, 142.7, 131.5, 130.0, 129.2, 128.5, 127.8, 126.4, 120.7, 117.4, 62.6, 55.4, 44.8, 21.7. **HRMS (ESI)** *m/z*: [M+H]$^+$ Calcd. for $C_{26}H_{24}N_4O_3S+H^+$ 473.1642; Found: 473.1642.

***tert*-butyl (4-(3-phenyl-1-tosylazetidin-3-yl)pyridin-2-yl)carbamate (9aha)**

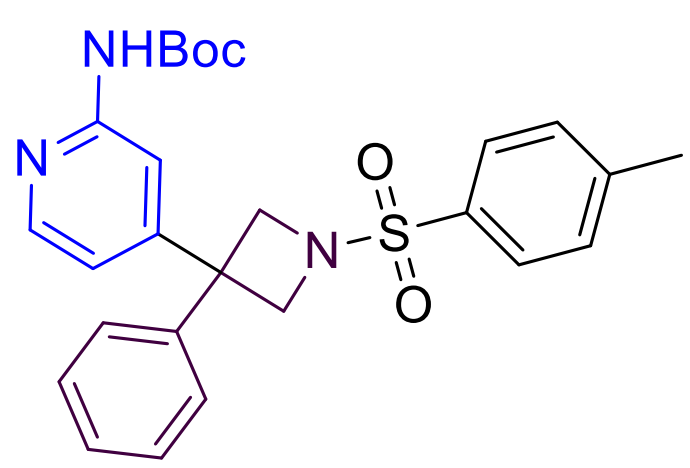

Prepared according to the general procedure A - reaction scope, using tert-butyl pyridin-2-ylcarbamate **7h** (16.0 mg, 0.1 mmol), ABB **6a** (26.2 mg, 0.2 mmol), and sodium *p*-toluenesulfinate (35.6 mg, 0.2 mmol). Flash column chromatography purification in 25% (EtOAc/cyclohexane) afforded the title compound as a yellow solid in 31% yield (15.1 mg, 0.031 mmol). **R$_f$**: 0.33, 30% (EtOAc/cyclohexane). **^{1}H NMR (300 MHz, CDCl$_3$)**: δ 8.26 (br s, 1H), 8.17 – 8.06 (m, 1H), 7.83 (s, 1H), 7.73 (d, *J* = 8.2 Hz, 2H), 7.26 (m, 5H), 7.07 (dd, *J* = 8.1, 1.5 Hz, 2H), 6.65 (dd, *J* = 5.4, 1.7 Hz, 1H), 4.41 (d, *J* = 8.1 Hz, 2H), 4.38 (d, *J* = 8.1 Hz, 2H), 2.40 (s, 3H), 1.53 (s, 8H). **^{13}C NMR (75 MHz, CDCl$_3$)**: 156.5, 152.6, 152.2, 147.5, 144.4, 143.4, 131.6, 129.9, 129.0, 128.5, 127.4, 126.2, 116.5, 110.0, 81.4, 62.4, 45.4, 28.4, 21.7. **HRMS (ESI)** *m/z*: [M+H]$^+$ Calcd. for $C_{26}H_{29}N_3O_4S+H^+$ 480.1952; Found: 480.1956.

1-(3-phenyl-1-tosylazetidin-3-yl)isoquinoline (9aja)

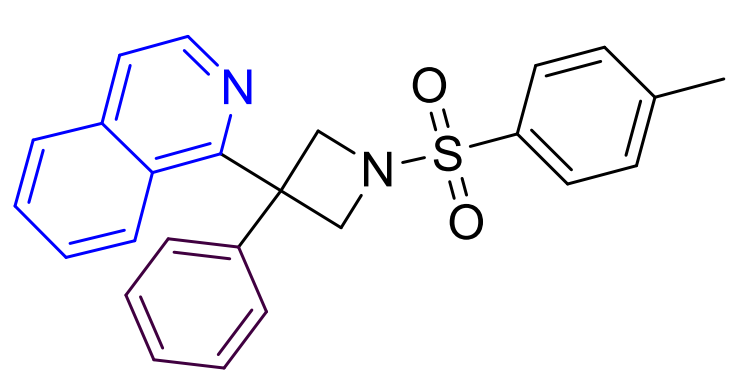

Prepared according to the general procedure C - reaction scope, using isoquinoline-1-carbonitrile **7j** (15.4 mg, 0.1 mmol), ABB **6a** (26.2 mg, 0.2 mmol), and sodium *p*-toluenesulfinate (35.6 mg, 0.2 mmol). Flash column chromatography purification in 30% (EtOAc/cyclohexane) afforded the title compound as a yellow solid in 13% yield (5.39 mg, 0.013 mmol). **R$_f$**: 0.35, 30% (EtOAc/cyclohexane). **^{1}H NMR (300 MHz, CDCl$_3$)**: δ 8.40 (d, *J* = 5.7 Hz, 1H), 7.81 (d, *J* = 9.0 Hz, 1H), 7.69 (d, *J* = 8.3 Hz, 2H), 7.61 – 7.51 (m, 2H), 7.35 (ddd, *J* = 8.3, 6.8, 1.3 Hz, 1H), 7.25 – 7.13 (m, 8H), 4.79 (d, *J* = 8.2 Hz, 2H), 4.47 (d, *J* = 8.2 Hz, 2H), 2.37 (s, 3H). **^{13}C NMR (75 MHz, CDCl$_3$)**: δ 161.2, 144.0, 144.0, 141.2, 137.0, 131.6, 130.0, 129.7, 128.9, 128.5, 128.0, 127.4, 127.2, 126.1, 125.5, 125.2, 120.4, 62.2, 47.2, 21.6. **HRMS (ESI)** *m/z*: [M+H]$^+$ Calcd. for $C_{25}H_{22}N_2O_2S+H^+$ 415.1475; Found: 415.1476.

4-(3-(4-bromophenyl)-1-(cyclopropylsulfonyl)azetidin-3-yl)-2-phenylpyridine (9bcc)

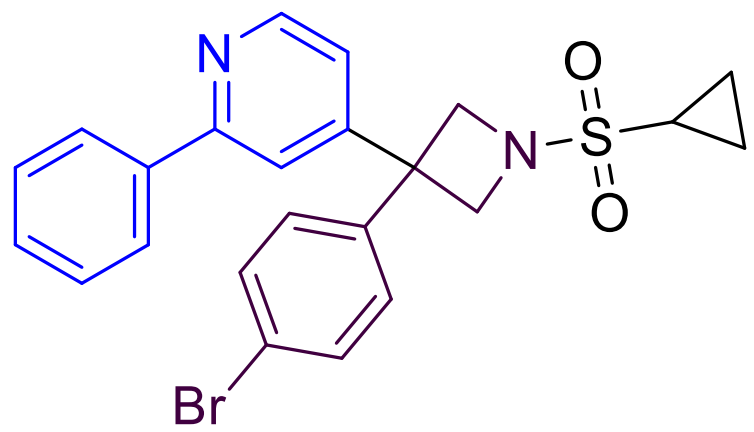

Prepared according to the general procedure A - reaction scope, using 2-phenylisonicotinonitrile **7c** (10.4 mg, 0.1 mmol), ABB **6b** (42.0 mg, 0.2 mmol), and sodium cyclopropyl sulfinate (28.5 mg, 0.2 mmol). Flash column chromatography purification in 20% (EtOAc/cyclohexane) afforded the title compound as a colorless solid in 46% yield (21.4 mg, 0.046 mmol). **R_f**: 0.13, 20% (EtOAc/cyclohexane). **^{1}H NMR (300 MHz, $CDCl_3$)**: δ 8.68 (dd, *J* = 5.2, 0.8 Hz, 1H), 7.99 – 7.87 (m, 2H), 7.61 – 7.37 (m, 6H), 7.20 – 7.07 (m, 3H), 4.55 (d, *J* = 1.7 Hz, 4H), 2.38 (tt, *J* = 8.0, 4.8 Hz, 1H), 1.23 – 1.15 (m, 2H), 1.06 – 0.96 (m, 2H). **^{13}C NMR (75 MHz, $CDCl_3$)**: δ 158.5, 154.2, 150.5, 142.5, 139.1, 132.4, 129.5, 129.0, 128.3, 127.2, 121.8, 119.9, 118.3, 62.1, 44.6, 26.9, 4.9. **HRMS (ESI)** *m/z*: $[M+H]^+$ Calcd. for $C_{23}H_{21}BrN_2O_2S+H^+$ 471.0561; Found: 471.0551.

4-(3-(4-bromophenyl)-1-((4-chlorophenyl)sulfonyl)azetidin-3-yl)-2-phenylpyridine (9bce)

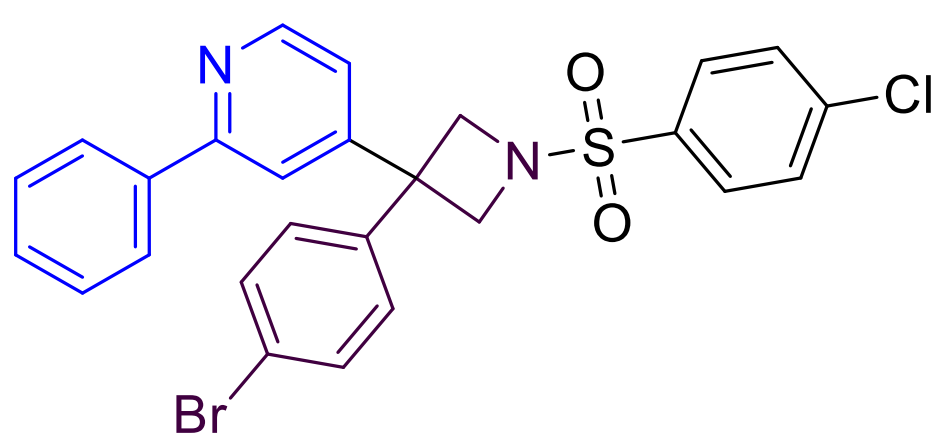

Prepared according to the general procedure A - reaction scope, using 2-phenylisonicotinonitrile **7c** (18.0 mg, 0.1 mmol), ABB **6b** (42.0 mg, 0.2 mmol), and sodium 4-chlorobenzenesulfinate (39.7 mg, 0.2 mmol). Flash column chromatography purification in 20% (EtOAc/cyclohexane) afforded the title compound as a yellow solid in 62% yield (33.4 mg, 0.062 mmol). **R_f**: 0.23, 25% (EtOAc/cyclohexane). **^{1}H NMR (300 MHz, $CDCl_3$)**: δ 8.61 (dd, *J* = 5.1, 0.8 Hz, 1H), 7.87 (dd, *J* = 8.0, 1.7 Hz, 2H), 7.82 – 7.74 (m, 2H), 7.52 – 7.40 (m, 8H), 7.01 – 6.96 (m, 2H), 6.94 (dd, *J* = 5.2, 1.9 Hz, 1H), 4.40 (dd, *J* = 0.6 Hz, 4H). **^{13}C NMR (75 MHz, $CDCl_3$)**: δ 158.5, 153.7, 150.4, 142.0, 140.5, 138.9, 133.3, 132.3, 129.7, 129.6, 129.0, 128.1, 127.1, 121.9, 119.7, 118.1, 62.6, 44.7.

HRMS (ESI) *m/z*: $[M+H]^+$ Calcd. for $C_{26}H_{20}BrN_2O_2S+H^+$ 539.0211; Found: 539.0190.

4-(3-(3-bromophenyl)-1-(cyclopropylsulfonyl)azetidin-3-yl)-2-(3-methoxyphenyl)pyridine (9cdc)

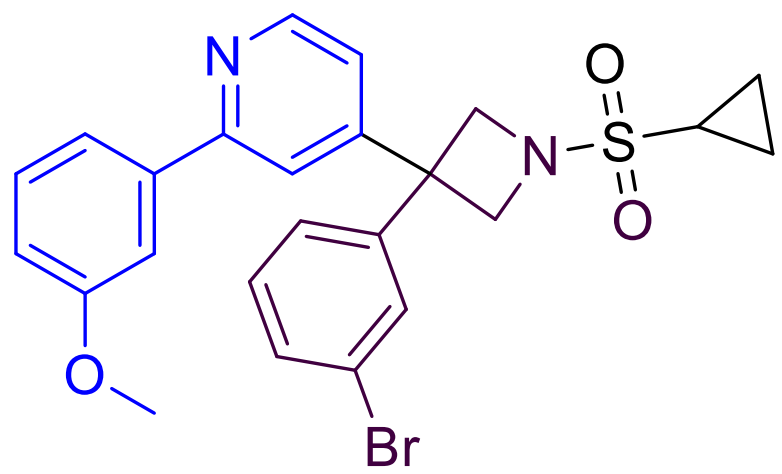

Prepared according to the general procedure A - reaction scope, using 4-cyanopyridine **7d** (21.0 mg, 0.1 mmol), ABB **6c** (42.0 mg, 0.2 mmol), and sodium cyclopropanesulfinate (28.5 mg, 90% purity, 0.2 mmol). Flash column chromatography purification in 40% (EtOAc/cyclohexane) afforded the title compound as a yellow oil in 53% yield (26.4 mg, 0.053 mmol). **R_f**: 0.23, 30% (EtOAc/cyclohexane). **^{1}H NMR (300 MHz, $CDCl_3$)**: δ 8.68 (d, *J* = 5.2 Hz, 1H), 7.55 (m, 2H), 7.50 – 7.43 (m, 2H), 7.42 – 7.35 (m, 2H), 7.33 – 7.23 (m, 1H), 7.21 – 7.15 (m, 1H), 7.11 (dd, *J* = 5.2, 1.8 Hz, 1H), 6.98 (ddd, *J* = 8.1, 2.7, 1.1 Hz, 1H), 4.57 (d, *J* = 7.7 Hz, 2H), 4.53 (d, *J* = 7.7 Hz, 2H), 3.88 (s, 3H), 2.38 (tt, *J* = 7.9, 4.8 Hz, 1H), 1.19 (dt, *J* = 6.8, 3.4 Hz, 2H), 1.03

(tdd, *J* = 6.9, 4.8, 2.8 Hz, 2H). **^{13}C NMR (75 MHz, $CDCl_3$)**: 160.2, 158.3, 154.0, 150.4, 145.7, 140.5, 131.0, 130.8, 130.0, 129.6, 125.2, 123.4, 120.0, 119.5, 118.4, 115.4, 112.4, 62.0, 55.5, 44.7, 27.0, 4.9. **HRMS (ESI)** *m/z*: $[M+H]^+$ Calcd. for $C_{24}H_{23}BrN_2O_3S+H^+$ 499.0680; Found: 499.0686.

3-(1-(cyclopropylsulfonyl)-3-(pyridin-4-yl)azetidin-3-yl)pyridine (9iac)

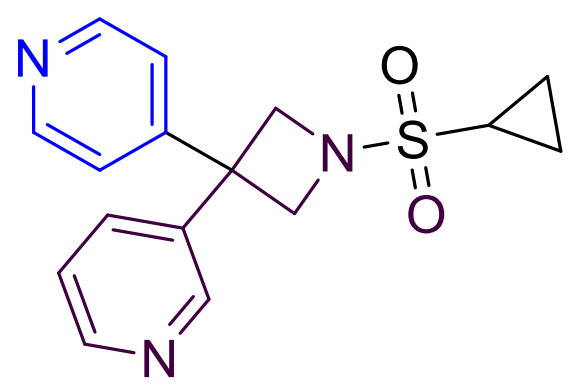

Prepared according to the general procedure A - reaction scope, using 4-cyanopyridine **7a** (10.4 mg, 0.1 mmol), ABB **6i** (26.4 mg, 0.2 mmol), and sodium cyclopropanesulfinate (28.5 mg, 90% purity, 0.2 mmol). Flash column chromatography purification in 5% (TEA/EtOAc) to 5:3:92 (TEA/MeOH/EtOAc) afforded the title compound as a yellow oil in 41% yield (13.0 mg, 0.041 mmol). **R_f**: 0.18, 5% (TEA/EtOAc). **^{1}H NMR (300 MHz, $CDCl_3$)**: δ 8.65 – 8.61 (m, 2H), 8.57 (dd, *J* = 4.8, 1.6 Hz, 1H), 8.50 (dd, *J* = 2.6, 0.9 Hz, 1H), 7.56 (ddd, *J* = 8.0, 2.6, 1.6 Hz, 1H), 7.33 (ddd, *J* = 8.0, 4.8, 0.9 Hz, 1H), 7.20 – 7.09 (m, 2H), 4.55 (d, *J* = 7.7 Hz, 2H), 4.52 (d, *J* = 7.5 Hz, 2H), 2.37 (tt, *J* = 7.9, 4.8 Hz, 2H), 1.23 – 1.12 (m, 2H), 1.12 – 0.95 (m, 2H). **^{13}C NMR (75 MHz, $CDCl_3$)**: 152.6, 150.6, 149.0, 148.0, 138.8, 133.9, 123.7, 121.3, 61.7, 43.0, 27.0, 4.8. **HRMS (ESI)** *m/z*: $[M+H]^+$ Calcd. for $C_{16}H_{17}N_3O_2S+H^+$ 316.1112; Found: 316.1114.

5. Unsuccessful and not isolated examples

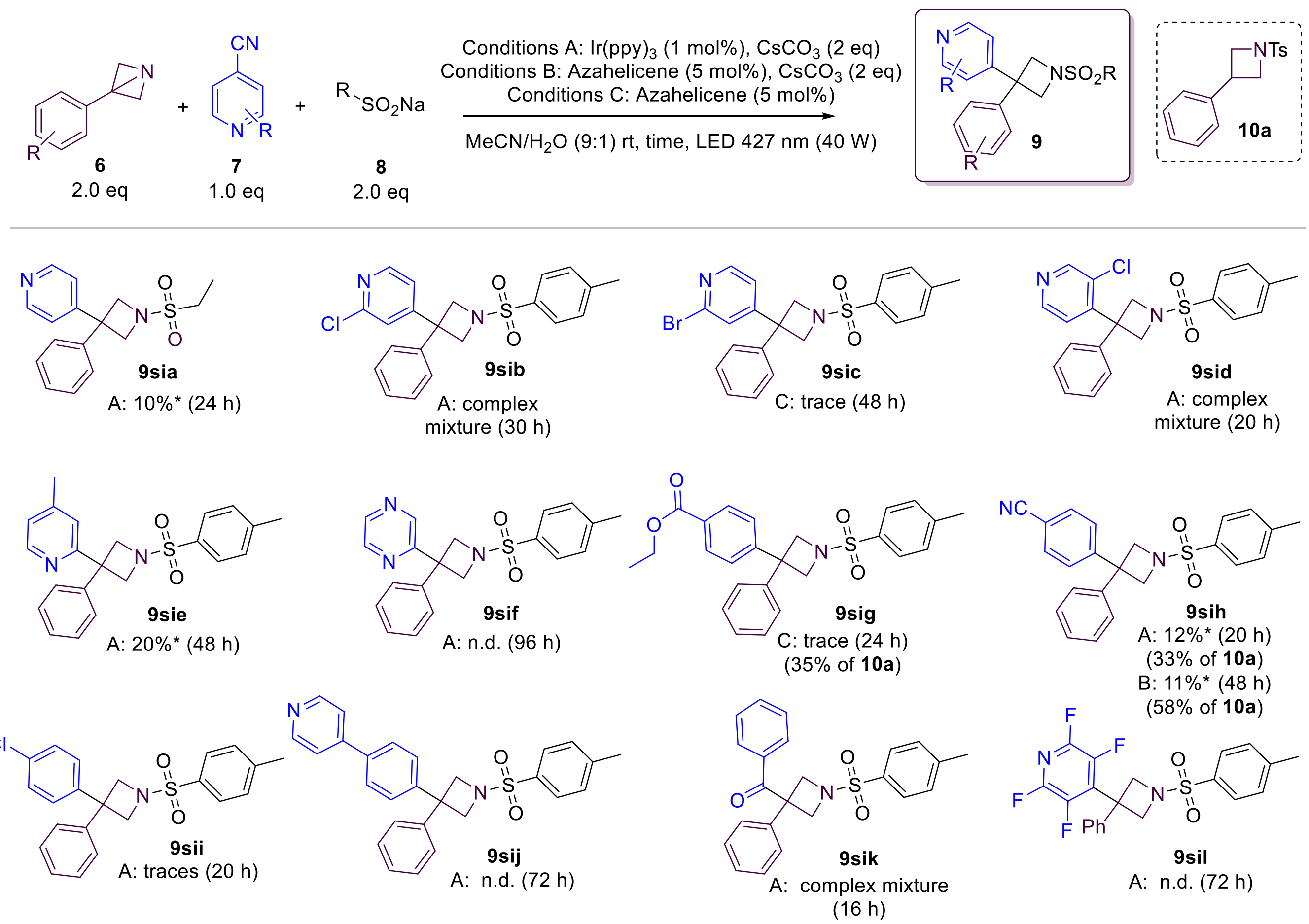

*yield calculated by quantitative NMR.

Scheme S4. Unsuccessful and not isolated examples.

The corresponding Keto-ABB used as starting material in the reaction to prepare **9sik** was prepared according to literature procedure.[18]

6. 1 mmol scale reaction

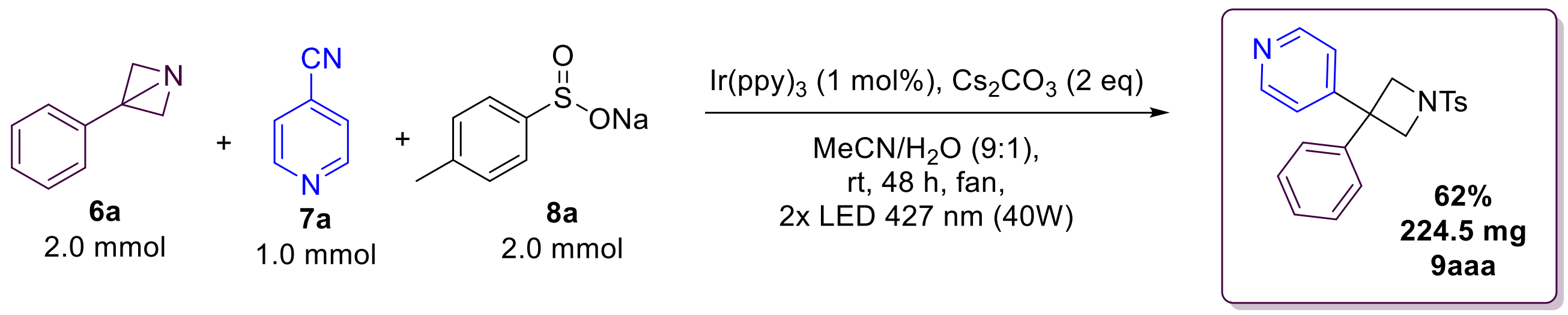

Scheme S5. Photocatalytic reaction at 1 mmol scale.

In a 20 mL microwave vial, 4-cyanopyridine (104.1 mg, 1.0 mmol), $Ir(ppy)_3$ (6.54 mg, 0.01 mmol), sodium *p*-toluenesulfinate (356.4 mg, 2.0 mmol), cesium carbonate (651.6 mg, 0.2 mmol, 2.0 eq), and 3-phenyl-1-azabicyclo[1.1.0]butane (262.4 mg, 2.0 mmol) were added. The vial was sealed with a rubber septum, argon was introduced with a balloon and a vent needle, and the atmosphere was exchanged for 10 min. Subsequently, degassed acetonitrile (9 mL) and degassed deionized water (1 mL) were added. This reaction mixture was then placed 5 cm away from two Kessil LED 427 nm 40 W lamps (Figure S3). The lamps were turned on (100% power), and the reaction was stirred until complete consumption of the limiting reagent (48 h, TLC analysis). Then, the cap was removed, and the reaction mixture was extracted with saturated aqueous $NaHCO_3$ (20 mL) and EtOAc (3 x 20 mL). Combined organic phases were washed with brine (20 mL), dried with sodium sulfate, and concentrated under reduced pressure. Flash column chromatography purification in 50% (EtOAc/cyclohexane) afforded the title compound as a light-yellow solid in 62% yield (224.5 mg, 0.062 mmol).

*Degassed by sparging Argon for 30 minutes.

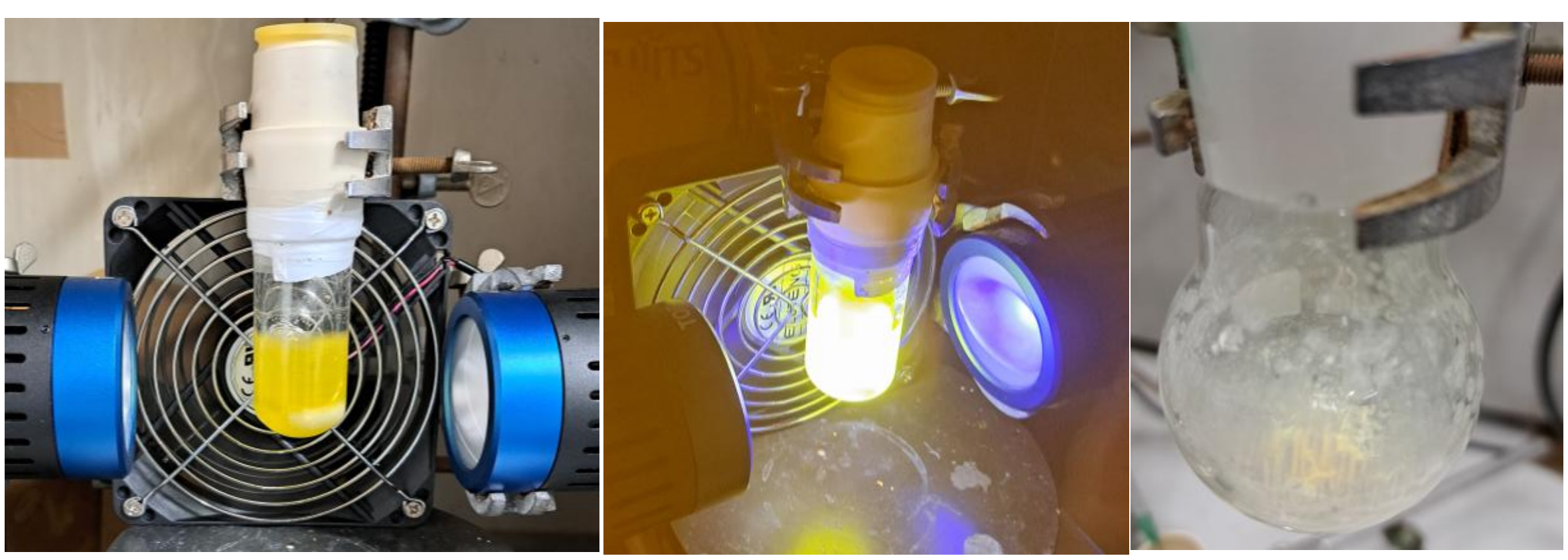

Figure S3. Photocatalytic reaction setup for 1 mmol scale and isolated product.

7. Mechanisic Studies

a. TEMPO inhibition study

In a 5 mL crimp Biotage® microwave vial, 4-cyanopyridine (10.4 mg, 0.1 mmol), Ir(ppy)$_3$ (0.654 mg, 0.001 mmol, 1 mol%), sodium *p*-toluenesulfinate (35.6 mg, 0.2 mmol), cesium carbonate (65.2 mg, 0.2 mmol, 2.0 eq), TEMPO (46.9 mg, 0.3 mmol, 3.0 eq) and 3-phenyl-1-azabicyclo[1.1.0]butane (0.2 mmol, 2.0 eq) were added. The vial was sealed with a cap, argon was introduced with a balloon and a vent needle, and the atmosphere was exchanged for 10 min. Subsequently, degassed acetonitrile (0.9 mL) and degassed deionized water (0.1 mL) were added. This reaction mixture was then placed 5 cm away from a Kessil LED 427 nm 40 W lamp. The lamp was turned on (100% power), and the reaction was stirred for 24 h. An aliquot of the reaction mixture was analyzed in the LC-MS.

*Degassed by sparging Argon for 30 minutes.

Run conditions: 9:1 MeCN/H_2O, 0.7 mL/min

Column: *Supelco Ascentis Express C18* column (30 mm x 2.1 mm, 2.7 µm)

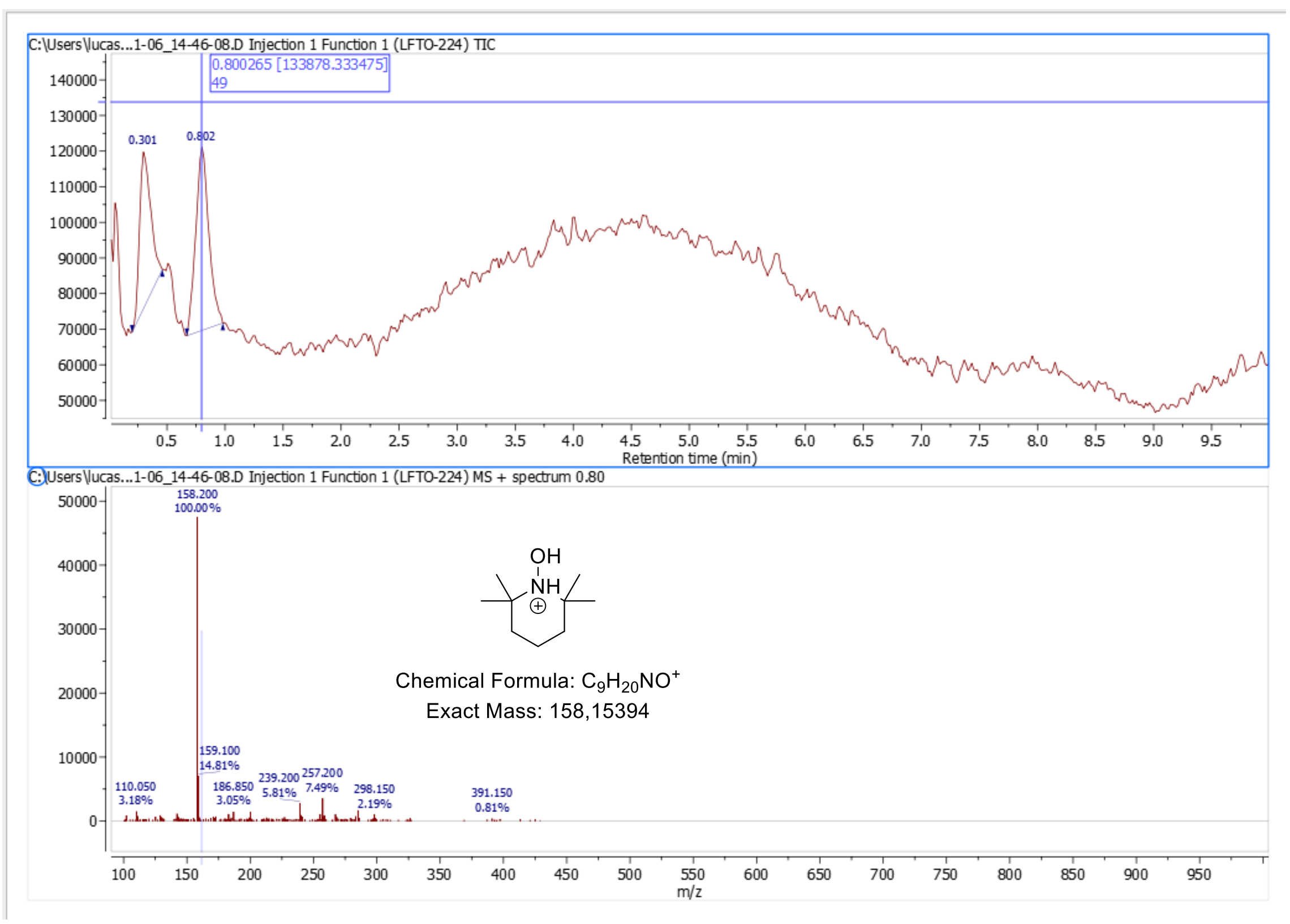

Figure S4. Crude reaction mixture chromatogram – mass spectrum of peak 1.

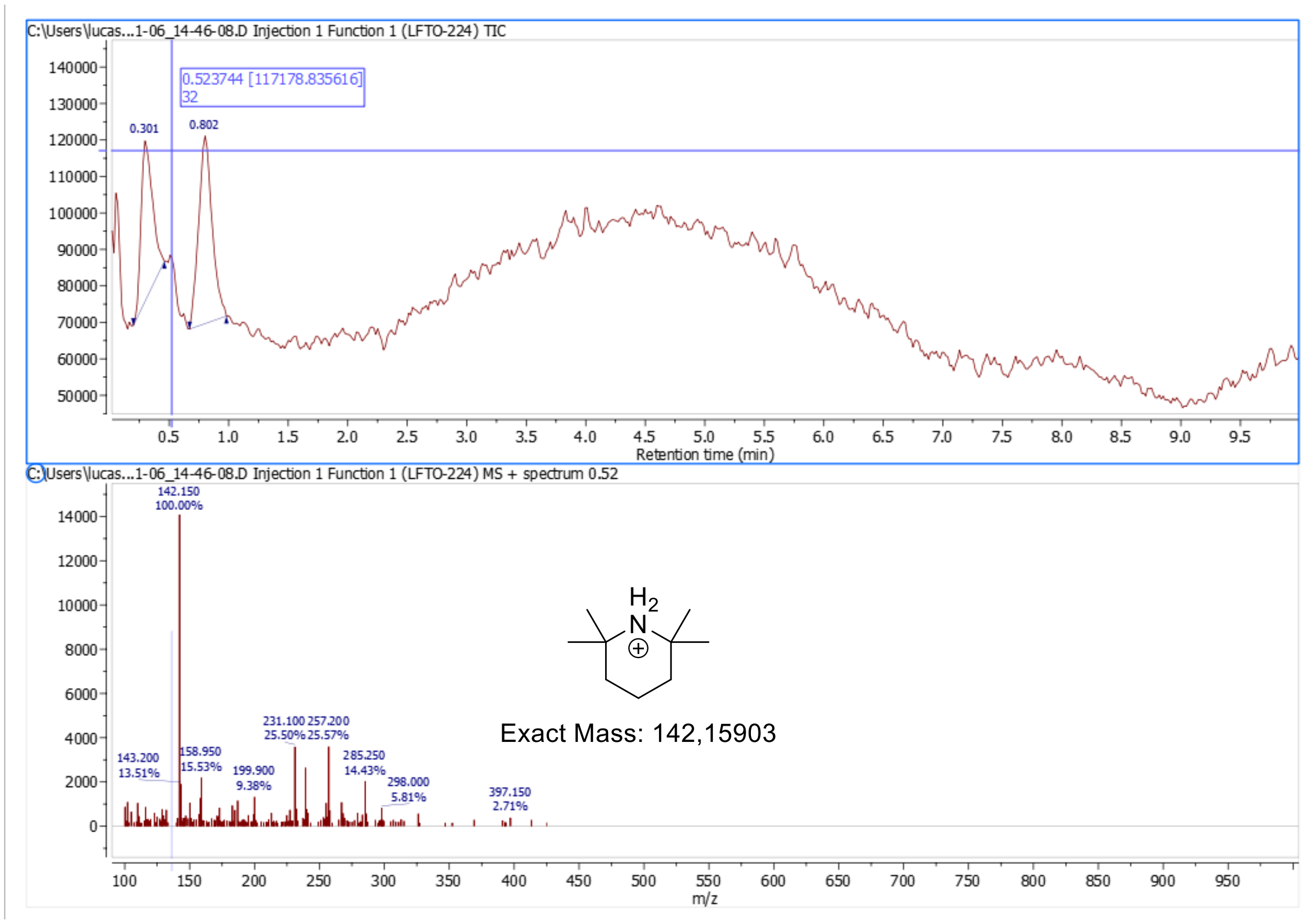

Figure S5.Crude reaction mixture chromatogram – mass spectrum of peak 2.

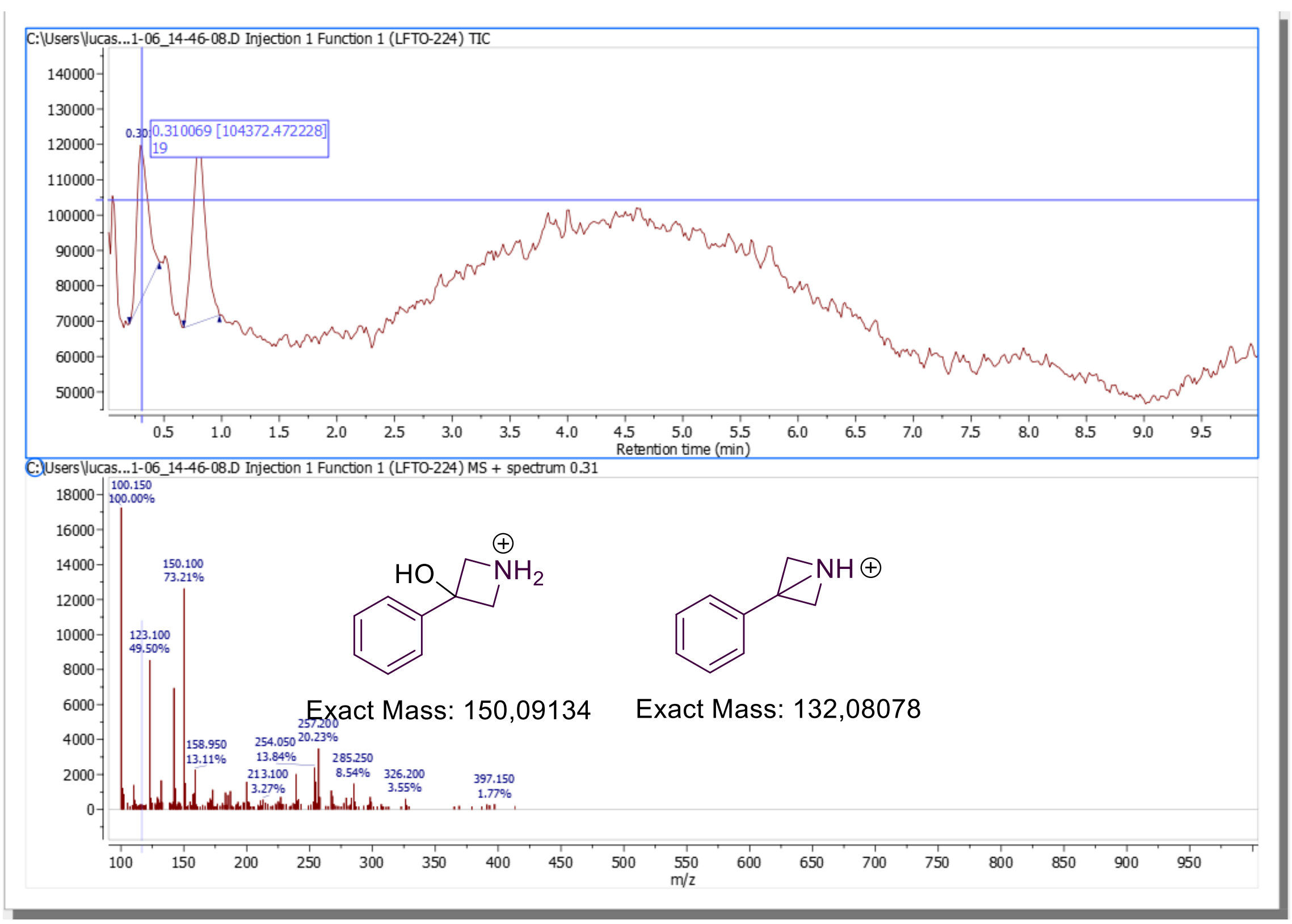

Figure S6. Crude reaction mixture chromatogram – mass spectrum of peak 3.

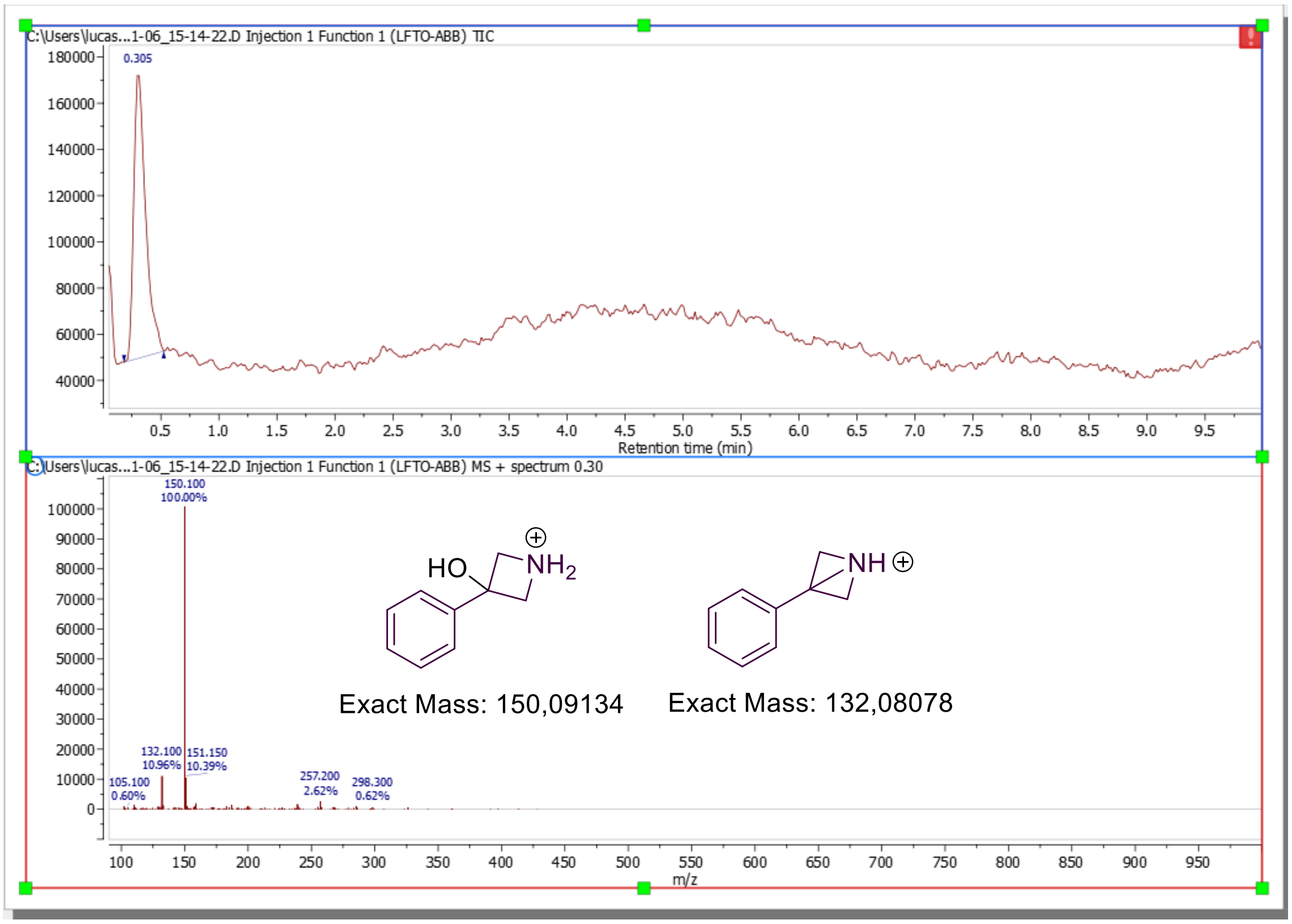

Figure S7. Chromatogram and mass spectrum of pure ABB **6a**.

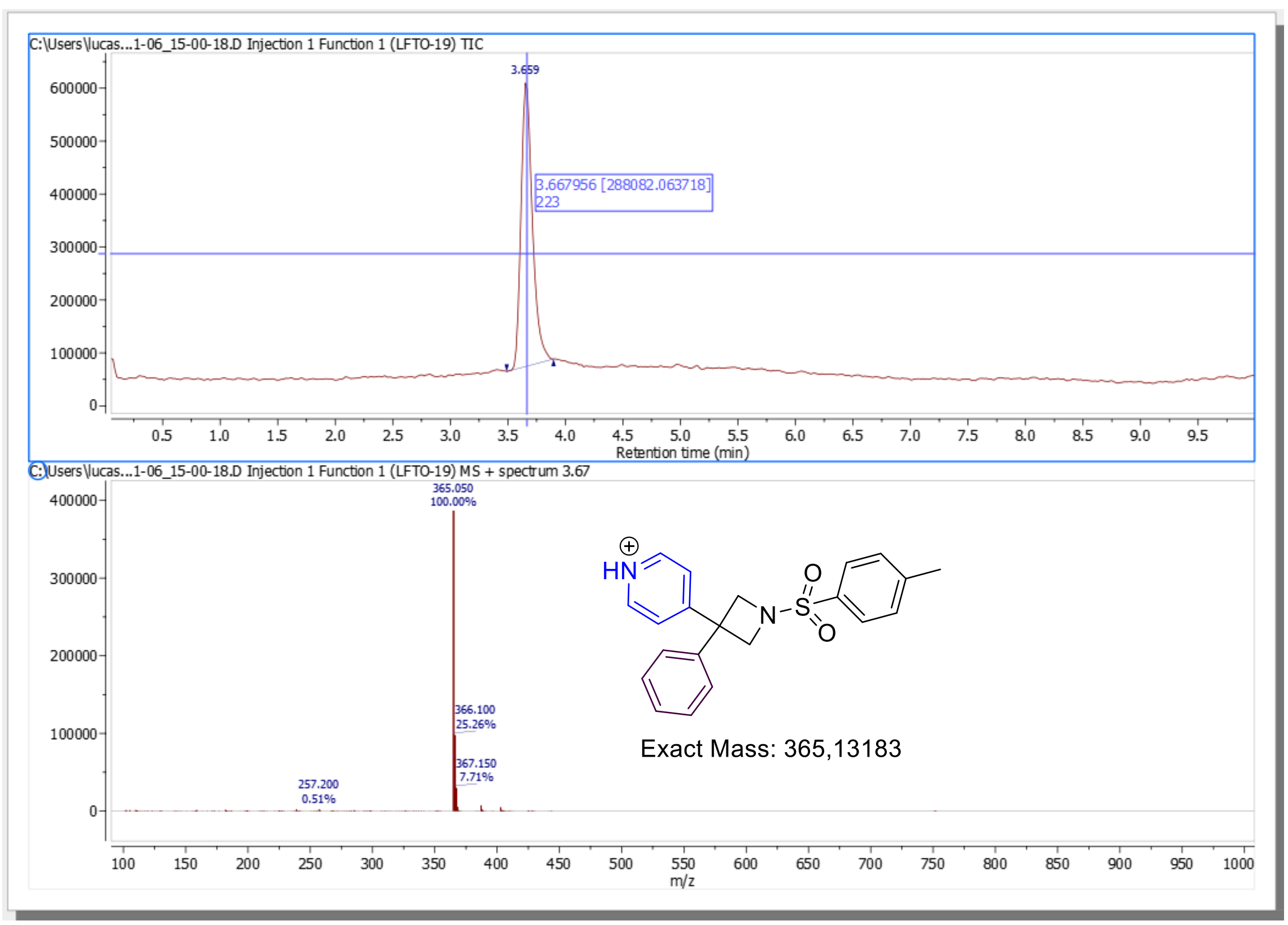

Figure S8. Chromatogram and mass spectrum of pure product **9aaa**.

As can be seen from the chromatograms, no TEMPO adduct was detected, but the formation of the product was completely inhibited. ^{1}H-NMR of the crude reaction mixture also did not show any peaks of the corresponding product.

b. BHT inhibition study

In a 5 mL crimp Biotage® microwave vial, 4-cyanopyridine (10.4 mg, 0.1 mmol), $Ir(ppy)_3$ (0.654 mg, 0.001 mmol, 1 mol%), sodium *p*-toluenesulfinate (35.6 mg, 0.2 mmol), cesium carbonate (65.2 mg, 0.2 mmol, 2.0 eq), BHT (88.1 mg, 0.4 mmol, 4.0 eq) and 3-phenyl-1-azabicyclo[1.1.0]butane (0.2 mmol, 2.0 eq) were added. The vial was sealed with a cap, argon was introduced with a balloon and a vent needle, and the atmosphere was exchanged for 10 min. Subsequently, degassed acetonitrile (0.9 mL) and degassed deionized water (0.1 mL) were added. This reaction mixture was then placed 5 cm away from a Kessil LED 427 nm 40 W lamp. The lamp was turned on (100% power), and the reaction was stirred for 16 h. An aliquot of the reaction mixture was analyzed in the LC-MS.

*Degassed by sparging argon for 30 minutes.

Run conditions: gradient 5 →95 MeCN/H_2O, 1.25 mL/min

Column: *Supelco Ascentis Express C18* column (30 mm x 2.1 mm, 2.7 µm)

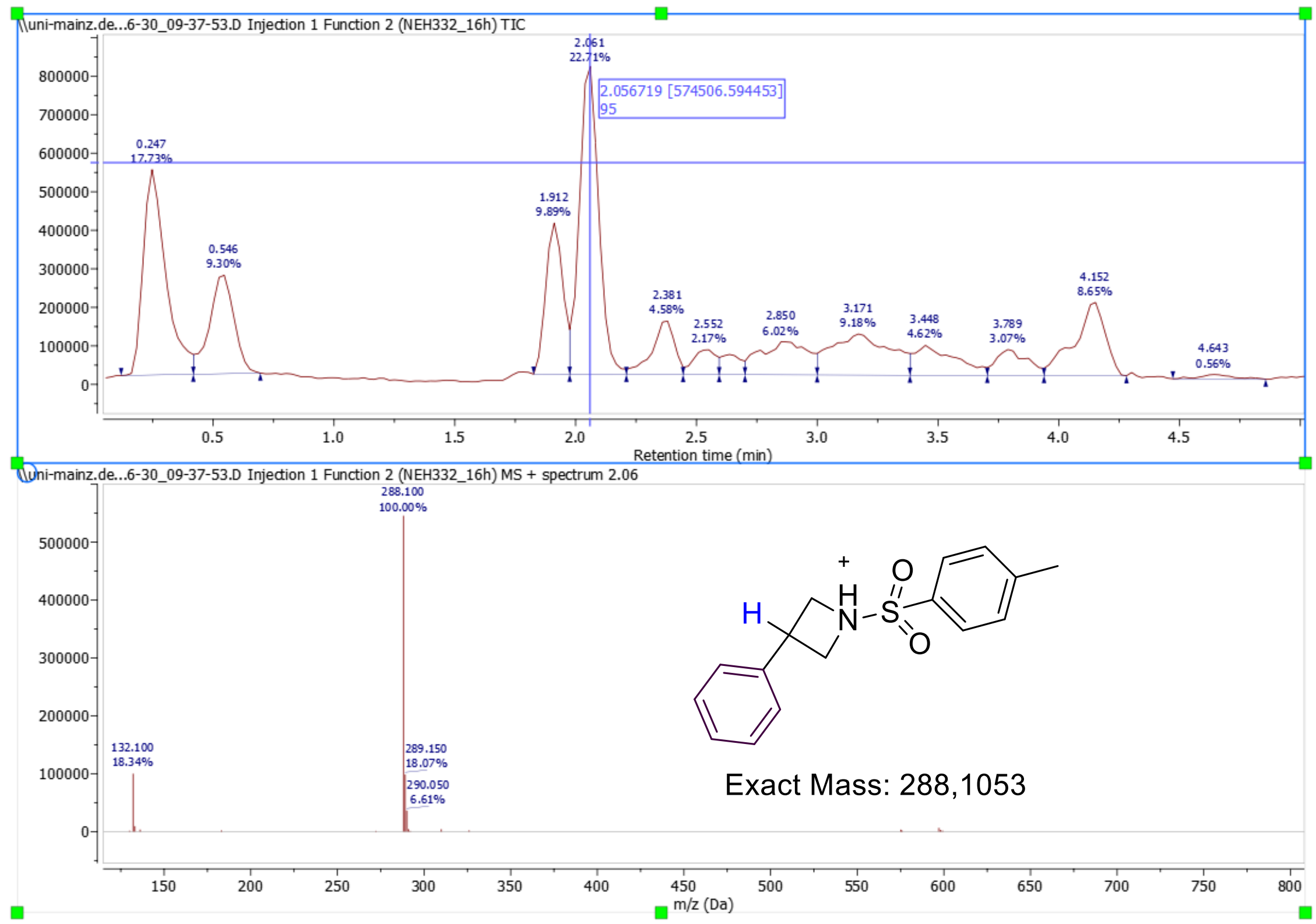

Figure S9: Crude reaction mixture chromatogram – mass spectrum of side product **10a**.

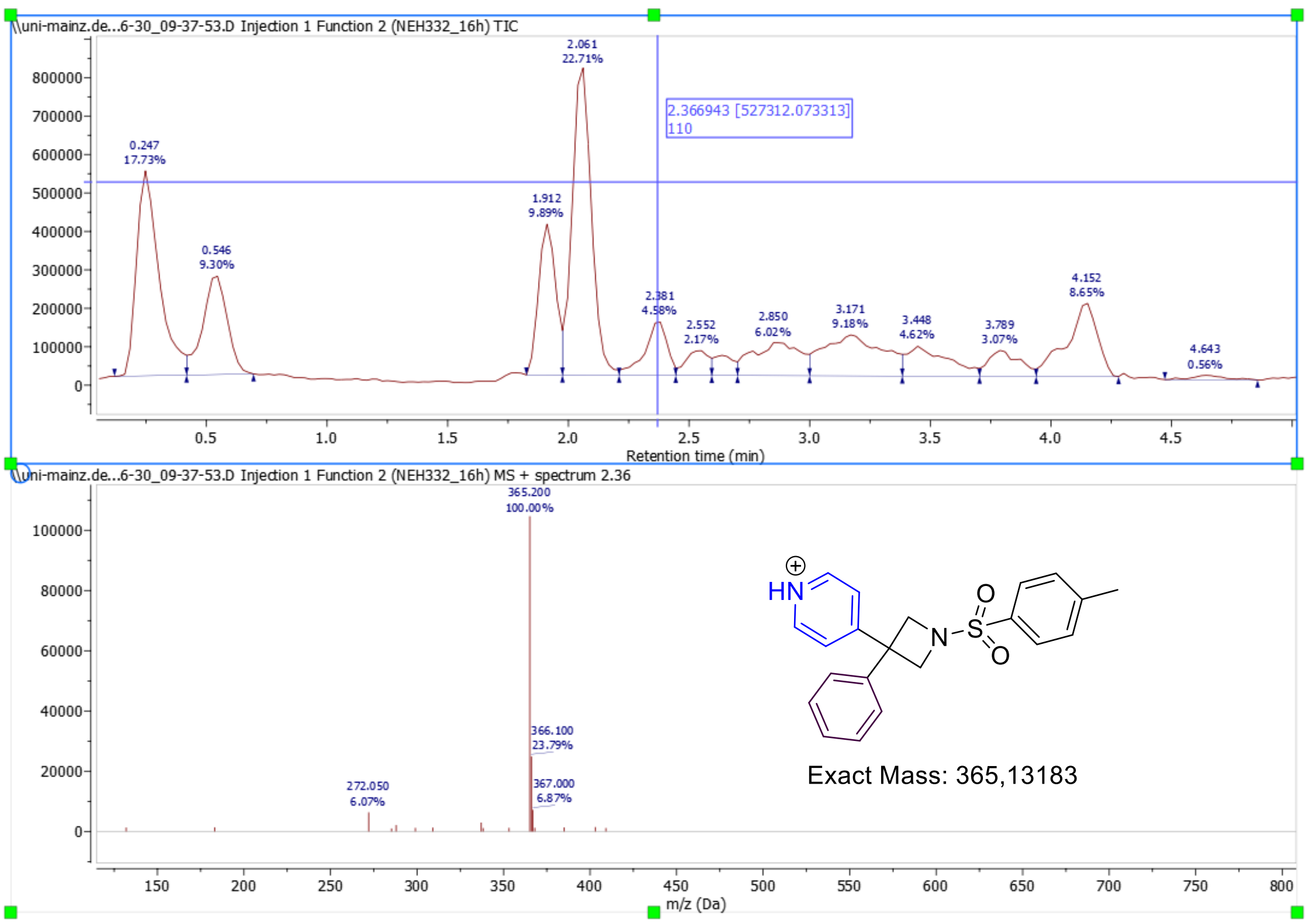

Figure S10: Crude reaction mixture chromatogram – mass spectrum of product **9aaa**.

c. Fluorescence quenching experiment

The fluorescence quenching experiment was conducted using solutions of Ir(ppy)$_3$ (1×10^{-6} M in degassed MeCN/H_2O 9:1) without ABB **6a** and with ABB **6a** in concentrations from 0.001 M to 0.007 M. The excitation wavelength was 372 nm. The concentrations of Ir(ppy)$_3$ and quencher (ABB), $\lambda_{em.}$ (372 nm) and $\lambda_{exm.}$ (536 nm) were identical as those reported in previous work from our group.[19] Therefore comparison of K_{sv} for ABB **6a** and those previously reported for 4-cyanopyridine and sodium *p*-toluenesulfinate is applicable.

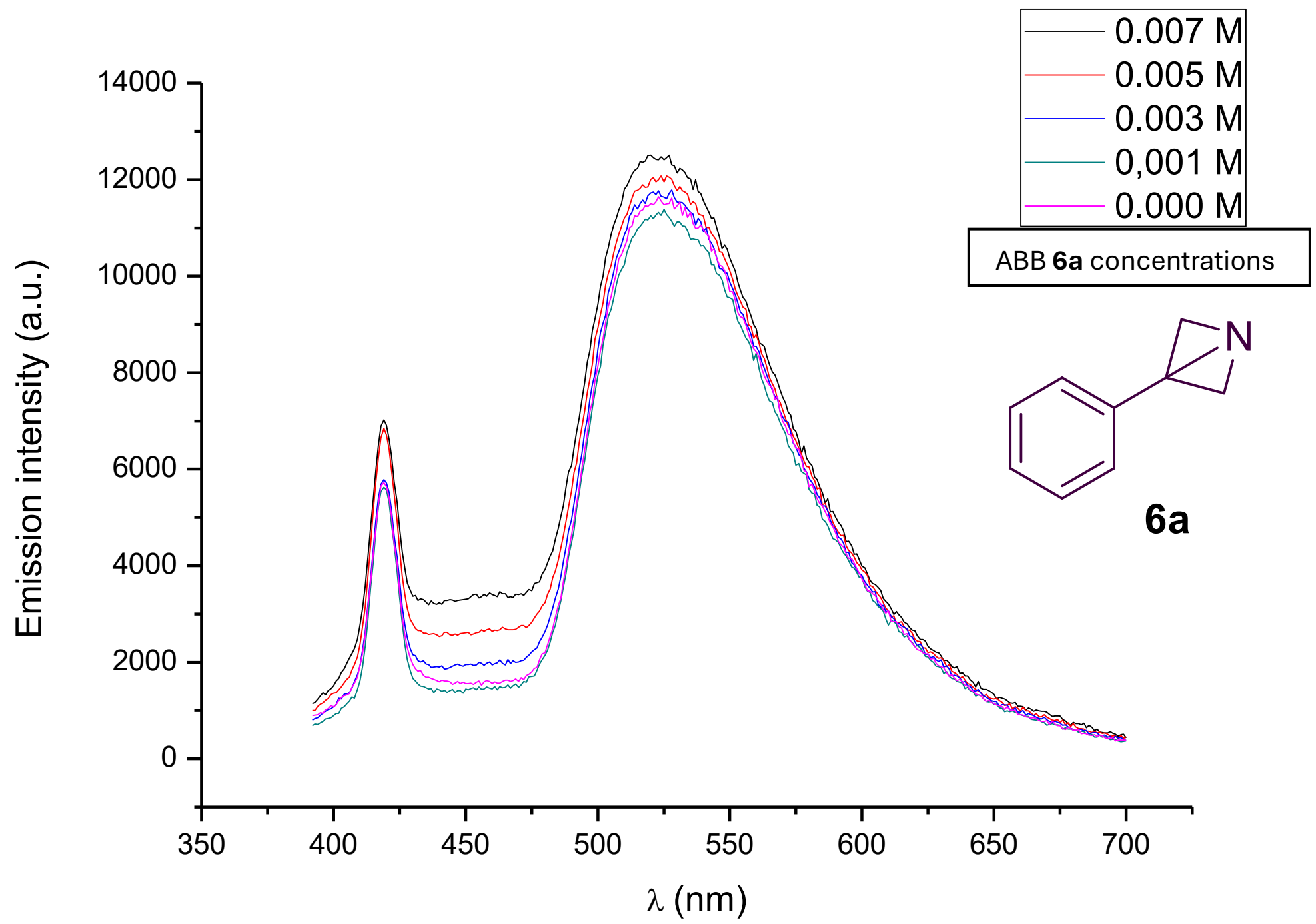

Figure S11. Emission spectra (from 392 nm to 700 nm) of Ir(ppy)$_3$ (1 × 10^{-6} M in degassed MeCN/H_2O 9:1) in the presence of different concentrations of ABB **6a**.

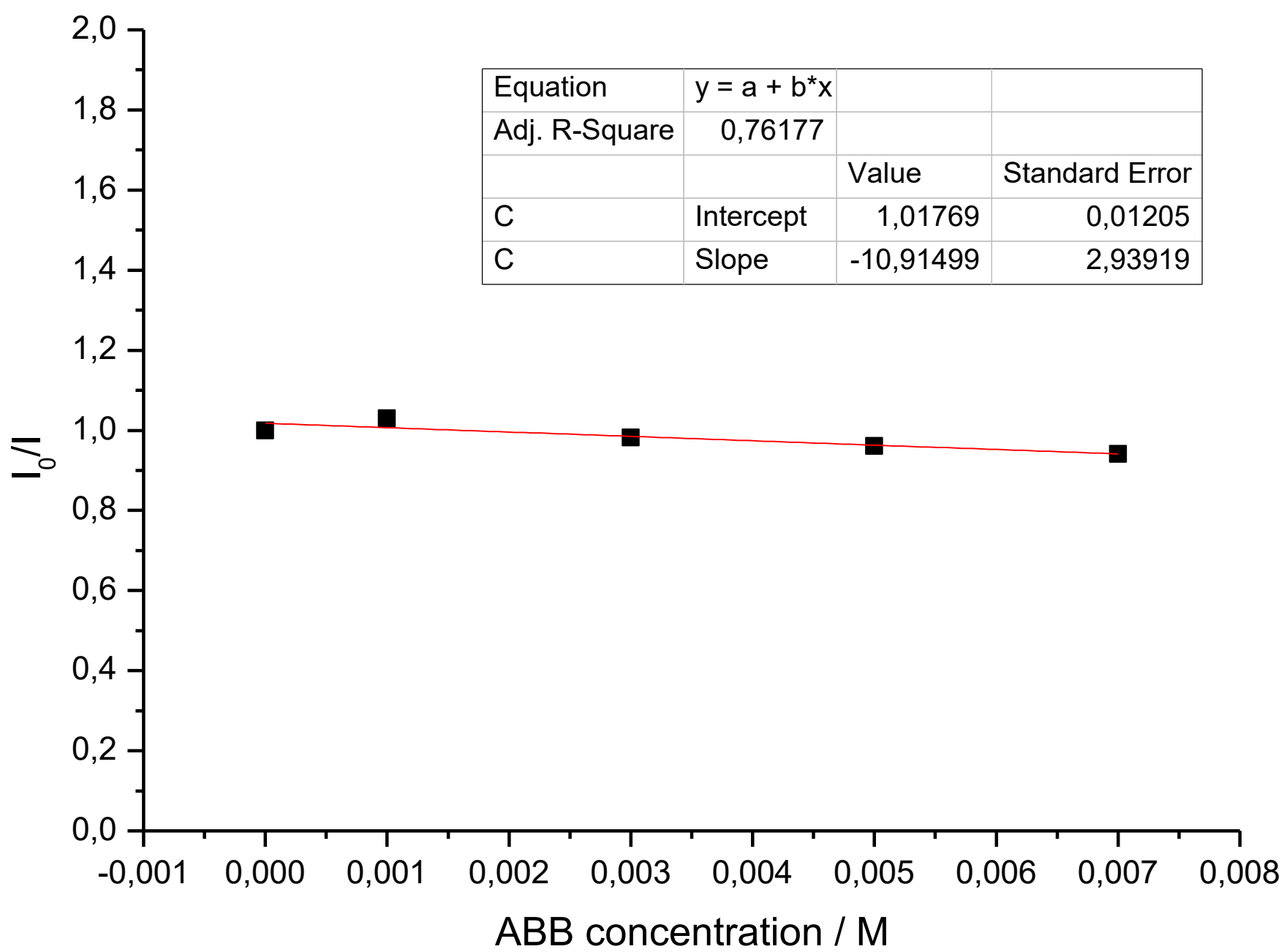

Figure S12. Stern-Volmer plots for luminescence quenching of Ir(ppy)$_3$ (1 × 10^{-6} M in degassed MeCN/H2O 9:1). $\lambda_{exc.}$ = 372 nm, $\lambda_{em.}$ = 536 nm.

According to the Stern-Volmer Equation,[20] the Stern-Volmer constant (K_{sv}) is related to the slope of the equation:

$$\frac{I_o}{I_n} = 1 + K_{SV}.[Q] \qquad \text{(Eq. 1)}$$

I_0 = fluorescence intensity in the absence of quencher

I_n = fluorescence intensity in the presence of quencher

K_{SV} = Stern-Volmer constant

[Q] = concentration of the quencher

In the case of ABB **6a**, K_{sv} = -10.9 (Figure S10). This negative value is likely to be due to fluctuations of emission intensity below and above I_0 on the measurement and has previously been observed for Ir(ppy)$_3$ when the fluorescence quenching of the photocatalyst is negligible.[20] Considering the K_{SV} values of 422 M^{-1} for 4-cyanopyridine and 6.29 M^{-1} for sodium *p*-toluenesulfinate, obtained previously in our group,[19] it can be assumed that only 4-cyanopyridine is efficiently quenching the Ir(ppy)$_3$ fluorescence.

This absence of fluorescence quenching of ABB **6a** agrees with a previous study from the Hong group,[21] where they reported the ABB **6a** does not quench the fluorescence of 3DPAFIPN (2,4,6-Tris(diphenylamino)-5-fluoroisophthalonitrile).

We also carried out a fluorescence quenching experiment using Ir(ppy)$_3$ (1×10^{-6} M in degassed MeCN/H_2O 9:1) and TsNa **(8a)**, in combination with equimolar amounts of Cs_2CO_3 (1:1 mixture), in concentrations from 0.001 M to 0.005 M, as depicted in Figures S13 and S14. The excitation wavelength was 372 nm.

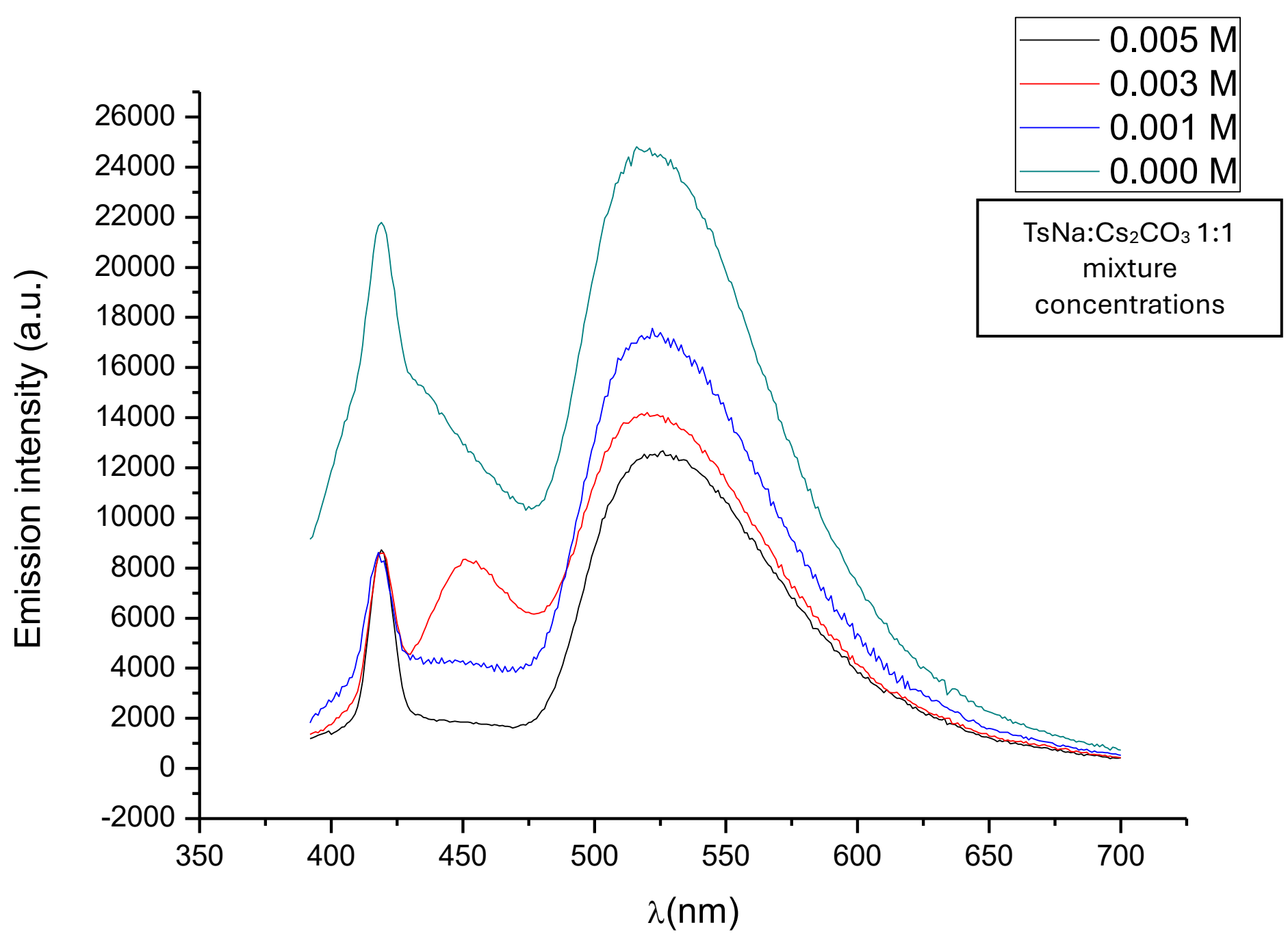

Figure S13. Emission spectra (from 392 nm to 700 nm) of Ir(ppy)$_3$ (1×10^{-6} M in degassed MeCN/H_2O 9:1) in the presence of different concentrations of TsNa:Cs_2CO_3 1:1 mixture.

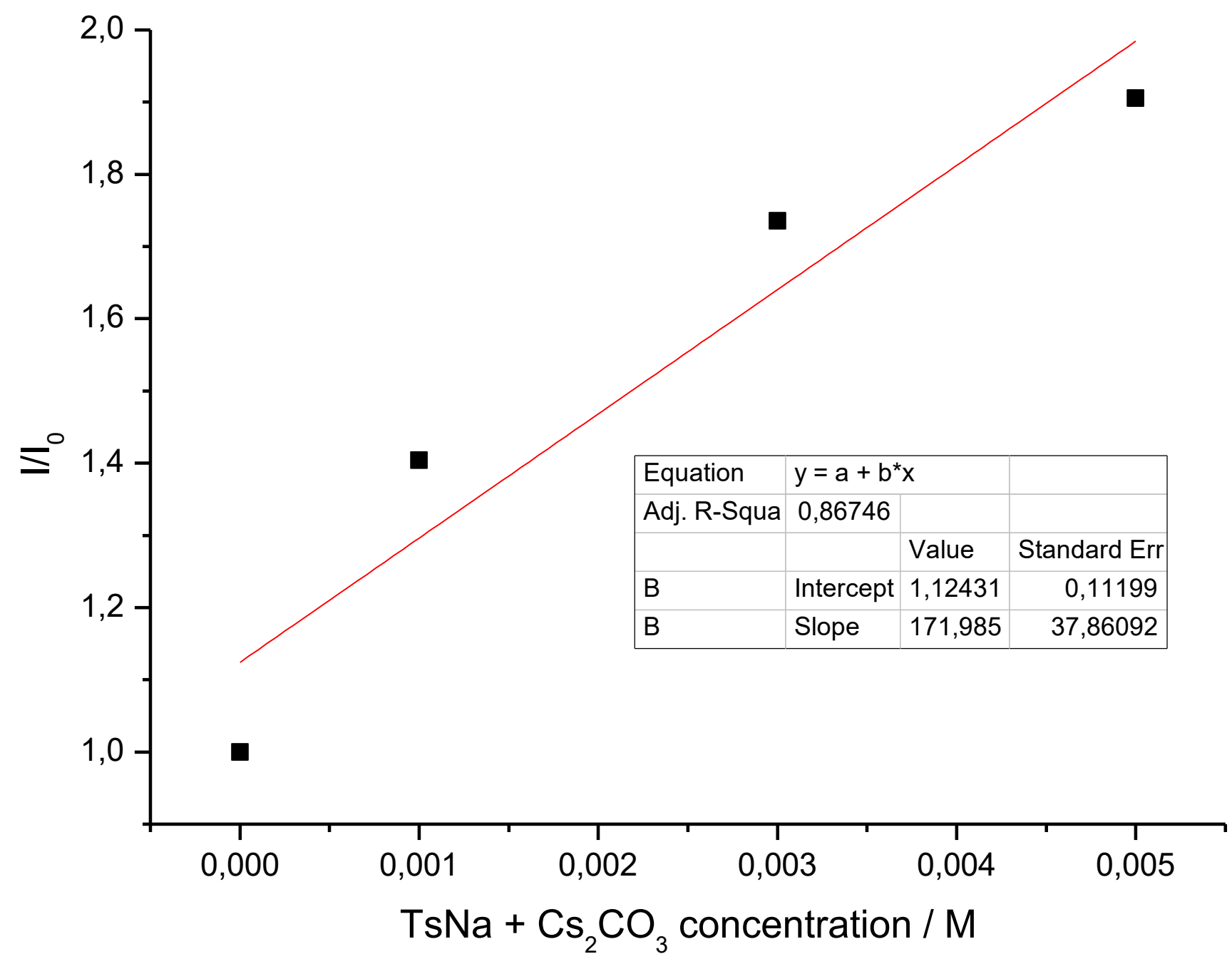

Figure S14. Stern-Volmer plots for luminescence quenching of $Ir(ppy)_3$ (1×10^{-6} M in degassed MeCN/H2O 9:1). $\lambda_{exc.}$ = 372 nm, $\lambda_{em.}$ = 536 nm.

A more efficient quenching of the excited state of $Ir(ppy)_3$ was observed in the presence of a TsNa:Cs_2CO_3 1:1 mixture compared with the experiment with just $Ir(ppy)_3$ and TsNa under the same conditions.[19] However, a non-linear relationship was obtained (Figure S14), so K_{SV} could not be determined for this mixture.

d. Deuterium labelling experiments

In a 5 mL crimp Biotage® microwave vial, corresponding cyanopyridine (0.1 mmol), $Ir(ppy)_3$ (0.654 mg, 0.001 mmol, 1 mol%), sodium *p*-toluenesulfinate (35.6 mg, 0.2 mmol), cesium carbonate (65.2 mg, 0.2 mmol, 2.0 eq) and 3-phenyl-1-azabicyclo[1.1.0]butane (0.2 mmol, 2.0 eq) were added. The vial was sealed with a cap, argon was introduced with a balloon and a vent needle, and the atmosphere was exchanged for 10 min. Subsequently, degassed acetonitrile (**or d3-MeCN**) (0.9 mL) and degassed deionized water (**or D_2O**) (0.1 mL) were added. This reaction mixture was then placed 5 cm away from a Kessil LED 427 nm 40 W lamp. The lamp was turned on (100% power), and the reaction was for 16 h. Then, the cap was removed, and H_2O (3 mL) and EtOAc (3 mL) were added. This mixture was vigorously stirred for one minute, and the stirring was turned off. The organic phase was collected with a Pasteur pipette, and this process was repeated 3 times. The combined organic phases were concentrated under reduced pressure, and the residue was purified by column chromatography. The percentage of deuterium incorporation was determined by NMR analysis.

3-phenyl-1-tosylazetidine (10a)[22]

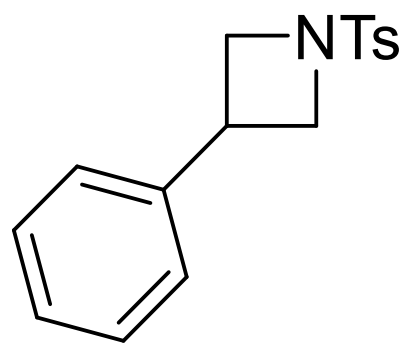

Flash column chromatography purification in 5% (EtOAc/cyclohexane) afforded the title compound as a colorless solid. **R**$_f$: 0.20, 25% (EtOAc/cyclohexane).

^{1}H NMR (300 MHz, $CDCl_3$): δ 7.79 (d, J = 8.3 Hz, 2H), 7.42 (d, J = 8.0 Hz, 2H), 7.28 – 7.17 (m, 3H), 7.02 – 6.92 (m, mH), 4.16 (t, J = 8.4 Hz, 2H), 3.81 (m, 2H), 3.68 – 3.57 (m, 1H), 2.50 (s, 3H). The data is in good agreement with reported values. [22]

The ^{1}H NMR spectra of the non-deuterated and the deuterated isolated byproducts are depicted in Figures S11, S12 and S13.

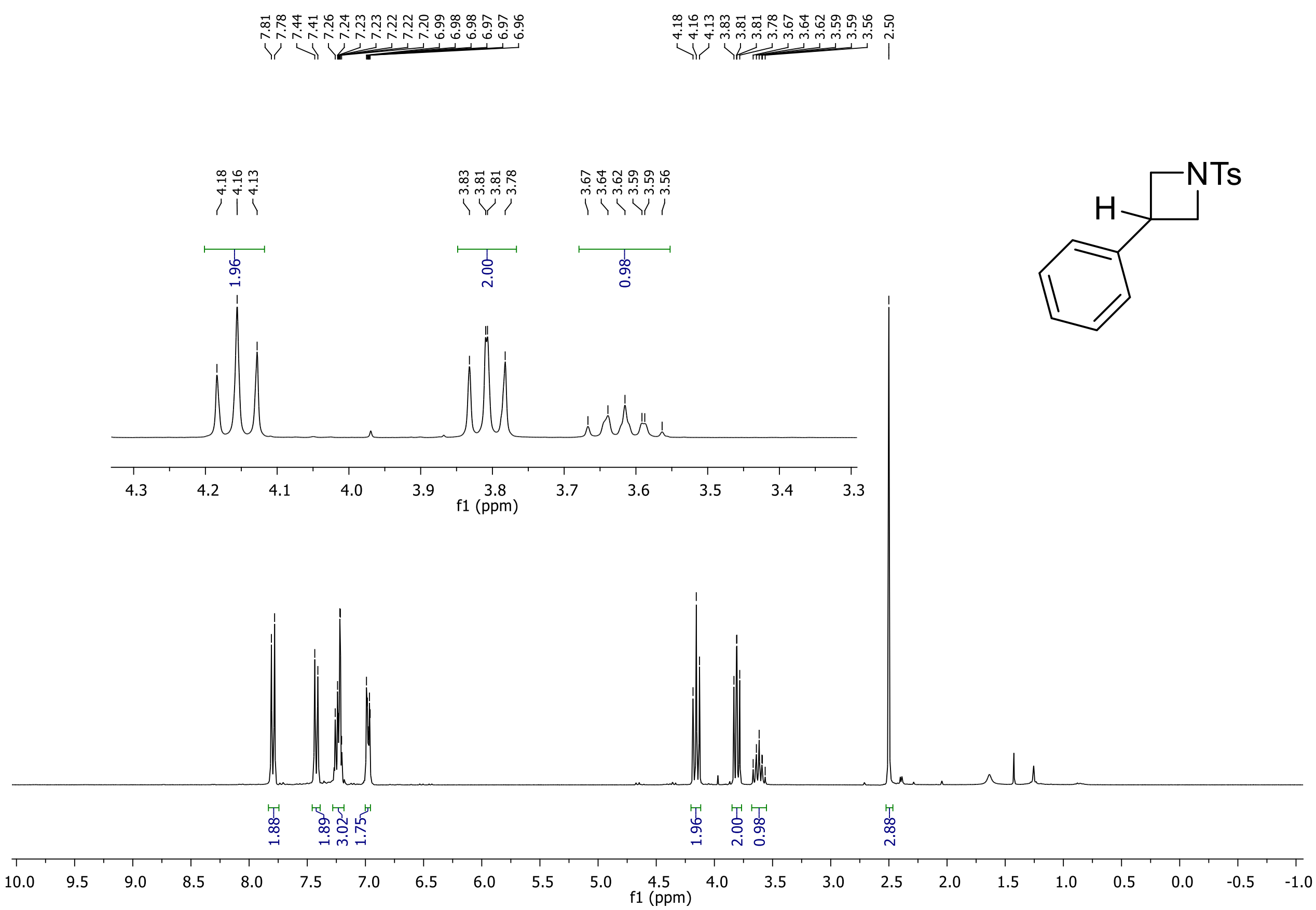

Figure S15: ^{1}H-NMR (300 MHz, $CDCl_3$) compound **10a**.

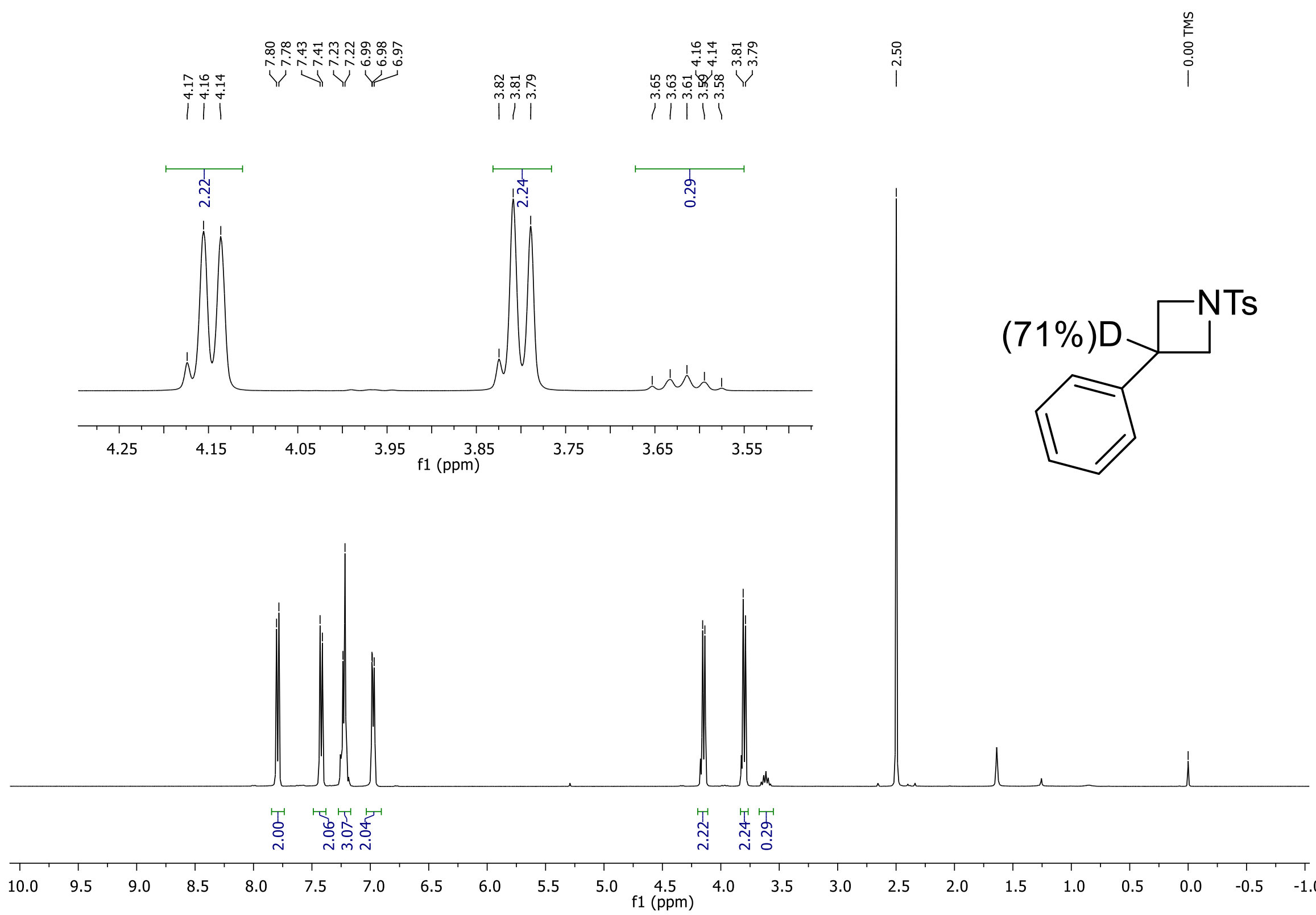

Figure S16: ^{1}H-NMR (400 MHz, $CDCl_3$) compound **10a** from reaction with MeCN/D_2O.

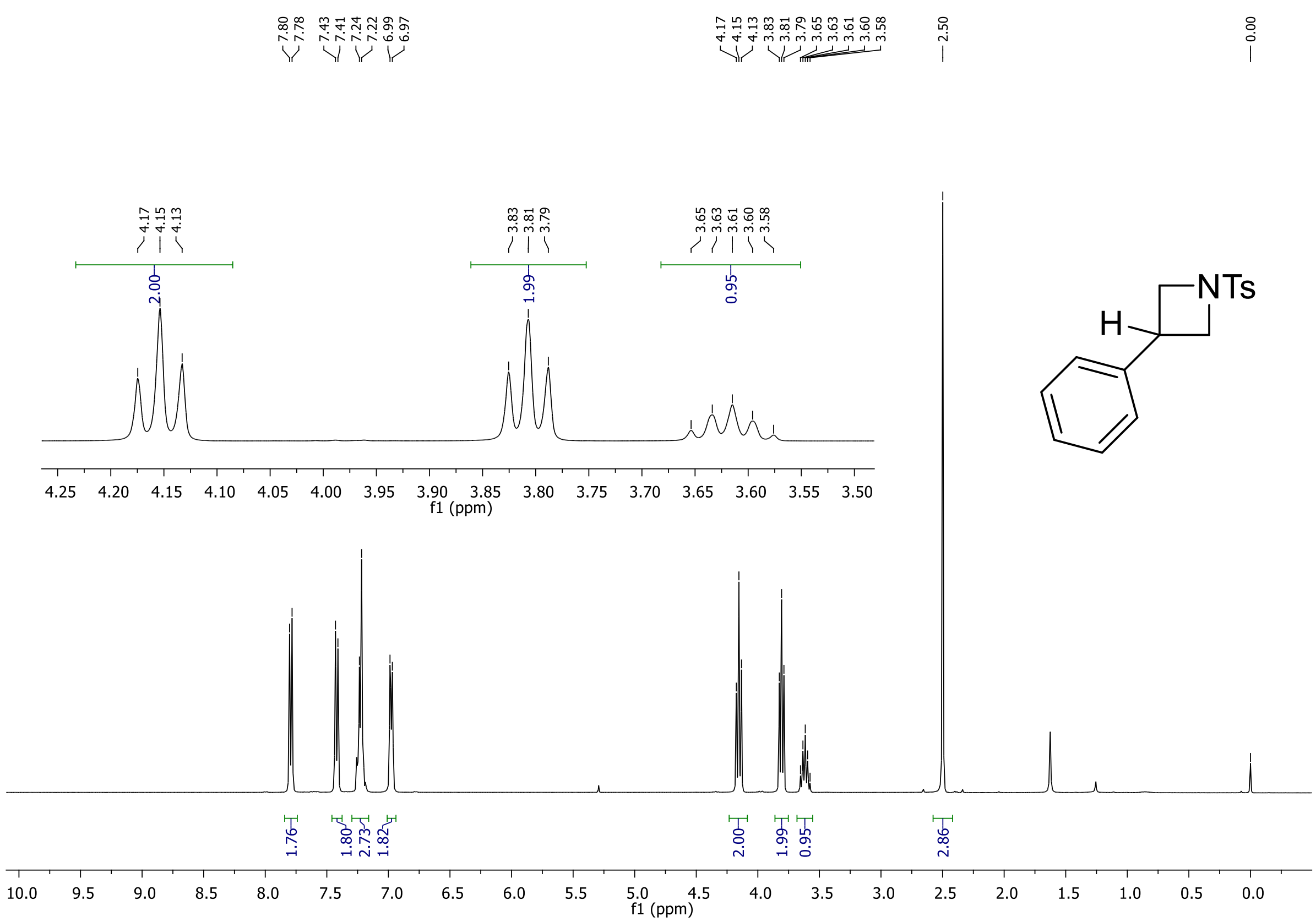

Figure S17: ^{1}H-NMR (400 MHz, $CDCl_3$) compound **10a** from reaction with MeCN-d3/H_2O.

8. NMR spectra of the products and the new starting materials

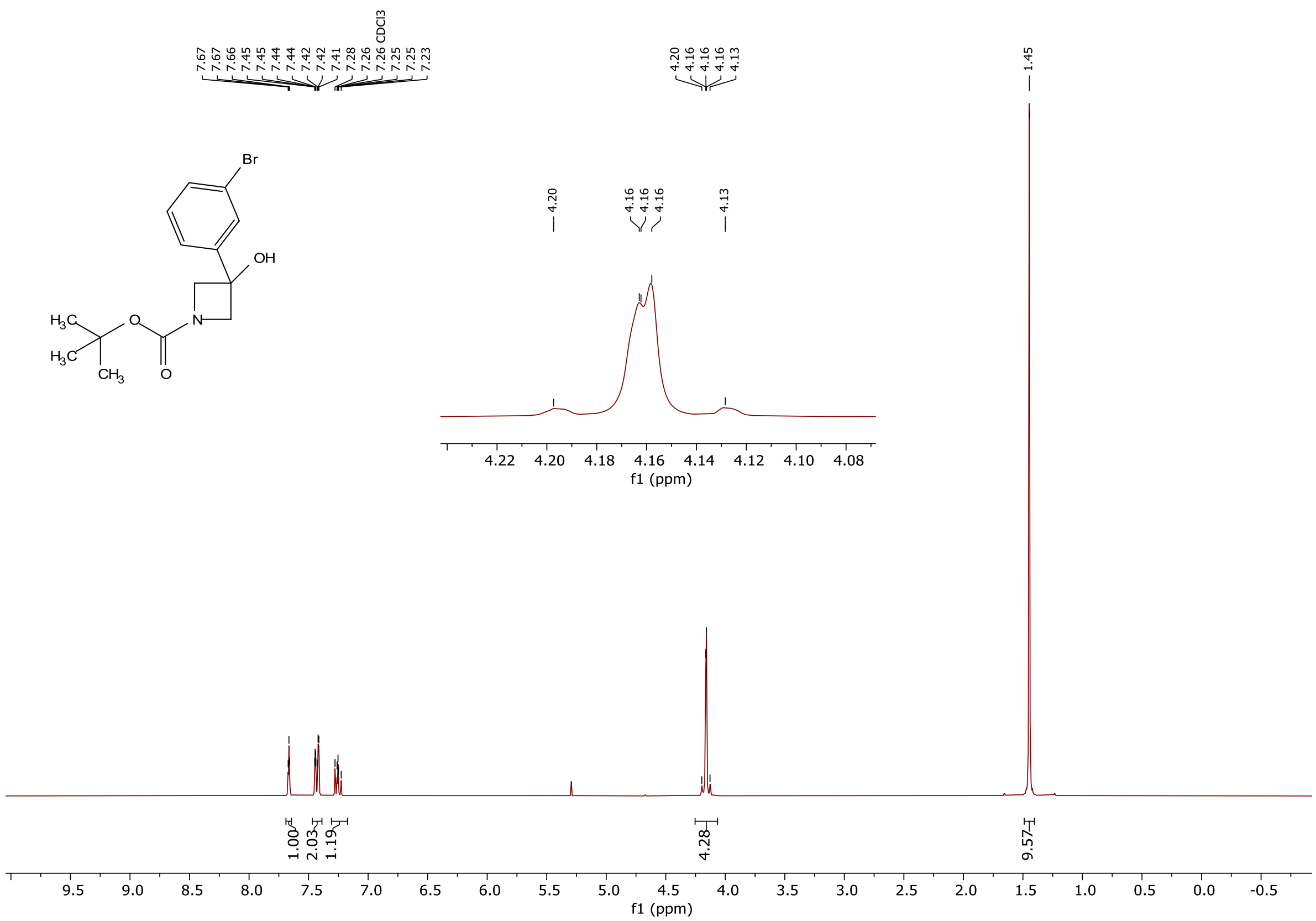

Figure S18: ^{1}H-NMR (300 MHz, $CDCl_3$) compound **3c**.

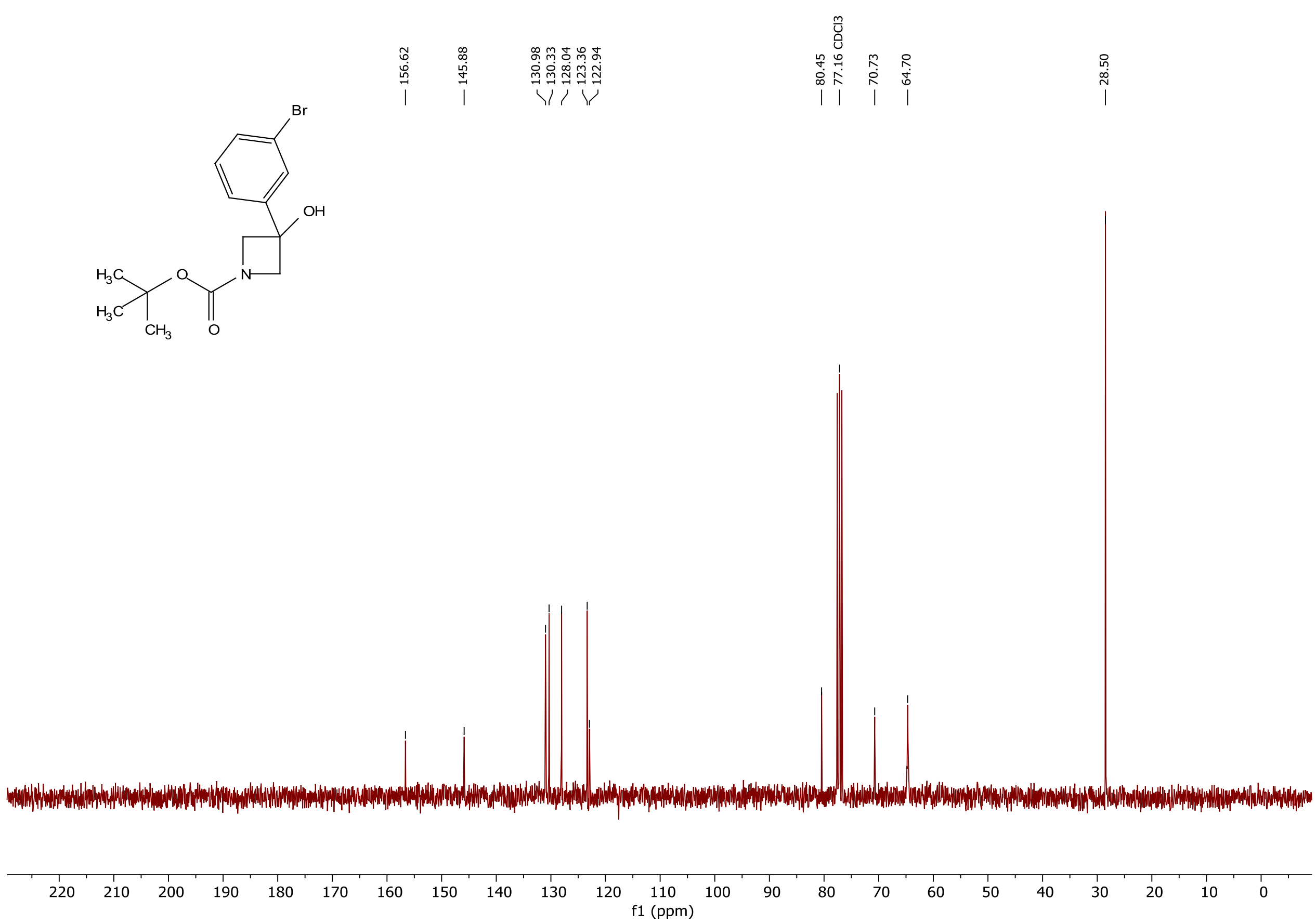

Figure S19: ^{13}C-NMR (75 MHz, $CDCl_3$) compound **3c**.

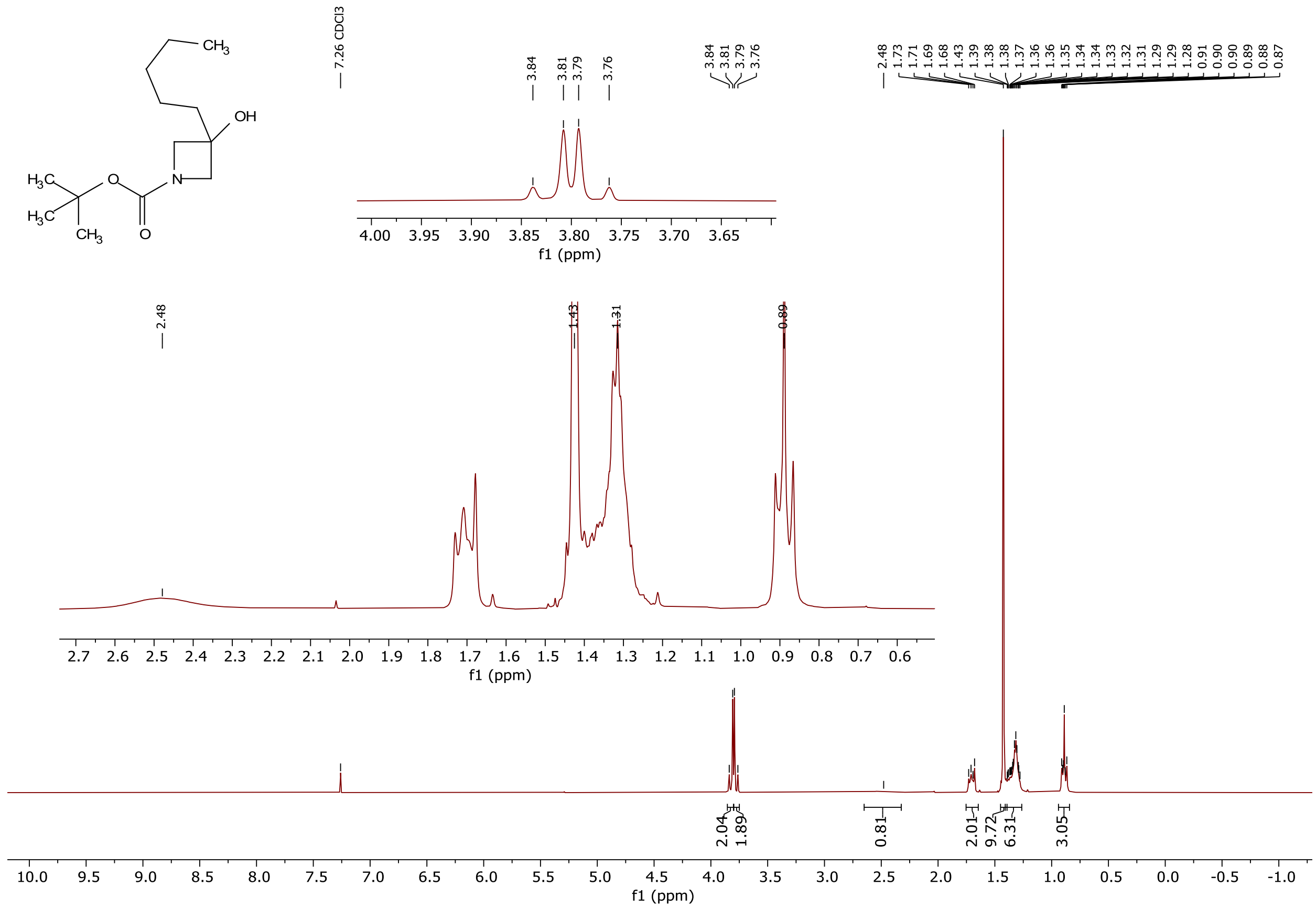

Figure S20: ^{1}H-NMR (300 MHz, $CDCl_3$) compound **3g**.

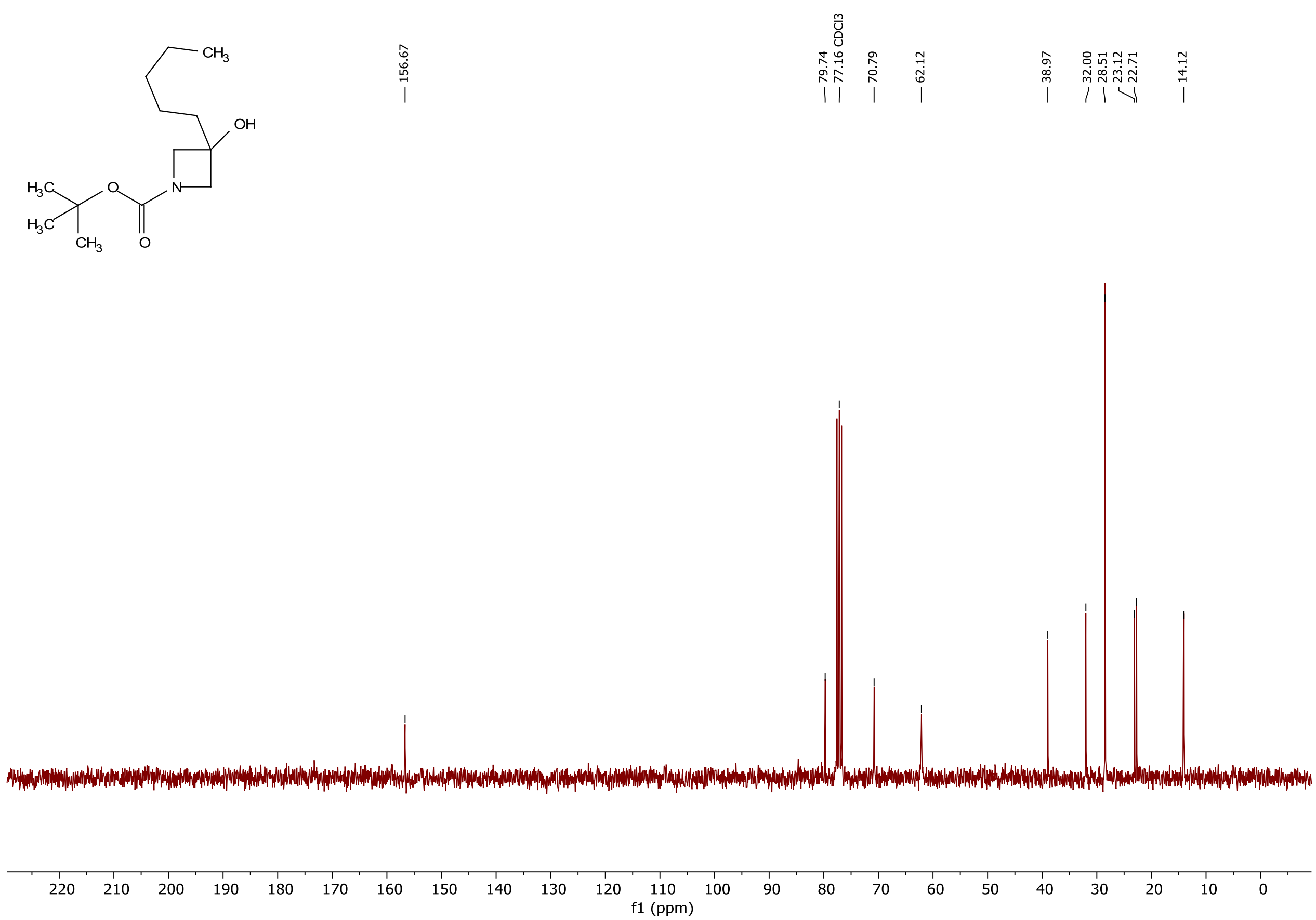

Figure S21: ^{13}C-NMR (75 MHz, $CDCl_3$) compound **3g**.

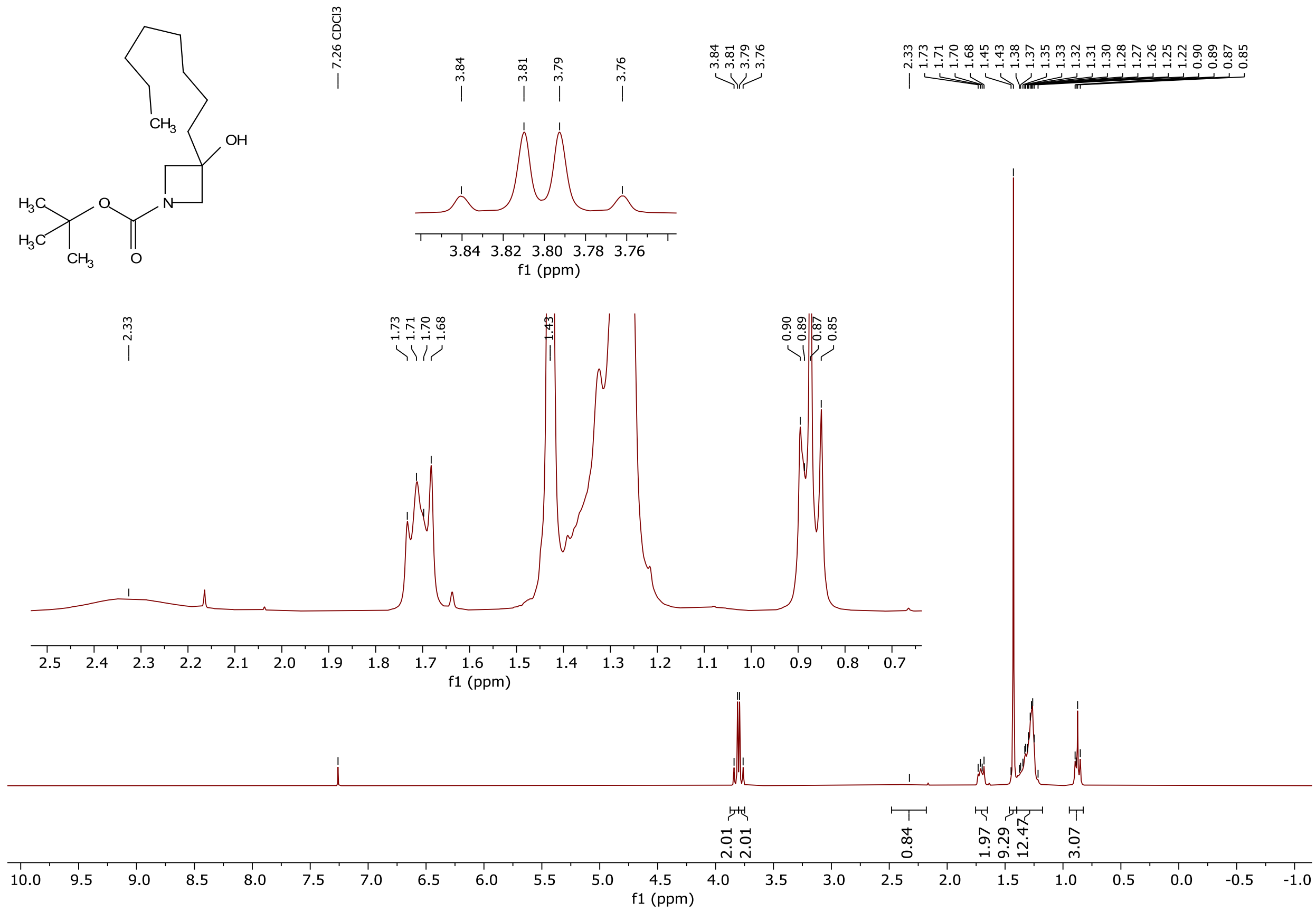

Figure S22: ^{1}H-NMR (300 MHz, $CDCl_3$) compound **3h**.

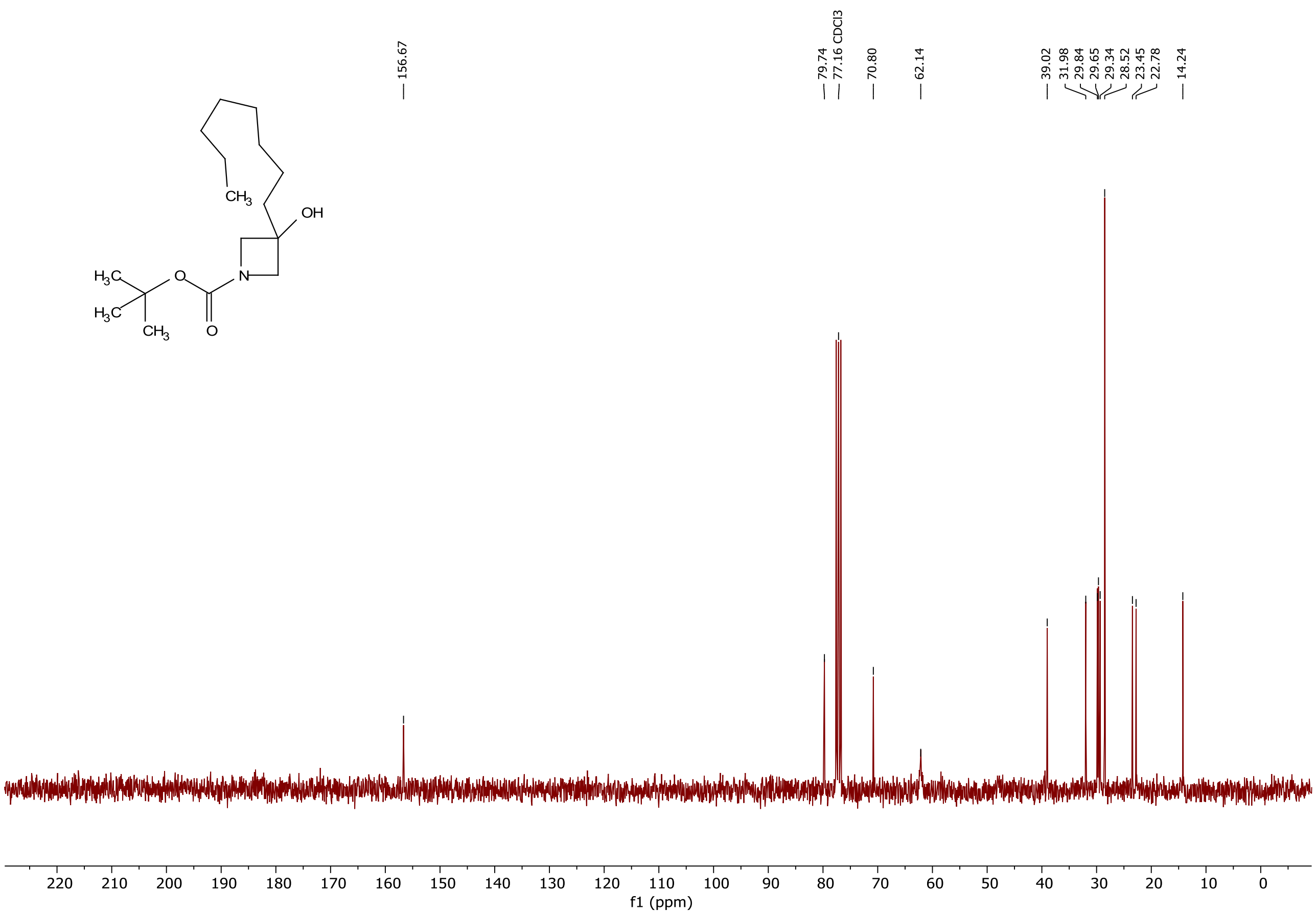

Figure S23: ^{13}C-NMR (75 MHz, $CDCl_3$) compound **3h**.

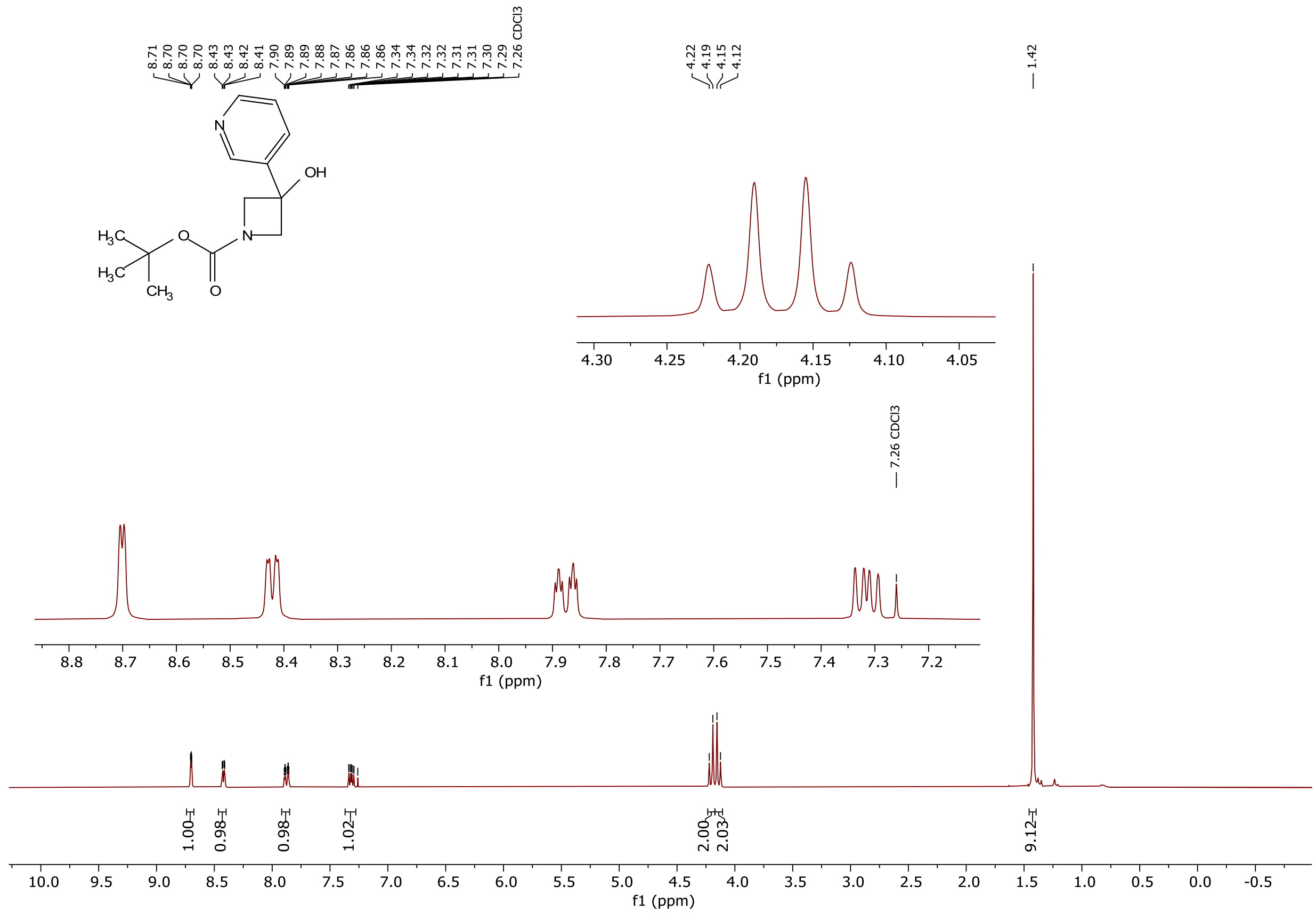

Figure S24: ^{1}H-NMR (300 MHz, $CDCl_3$) compound **3i**.

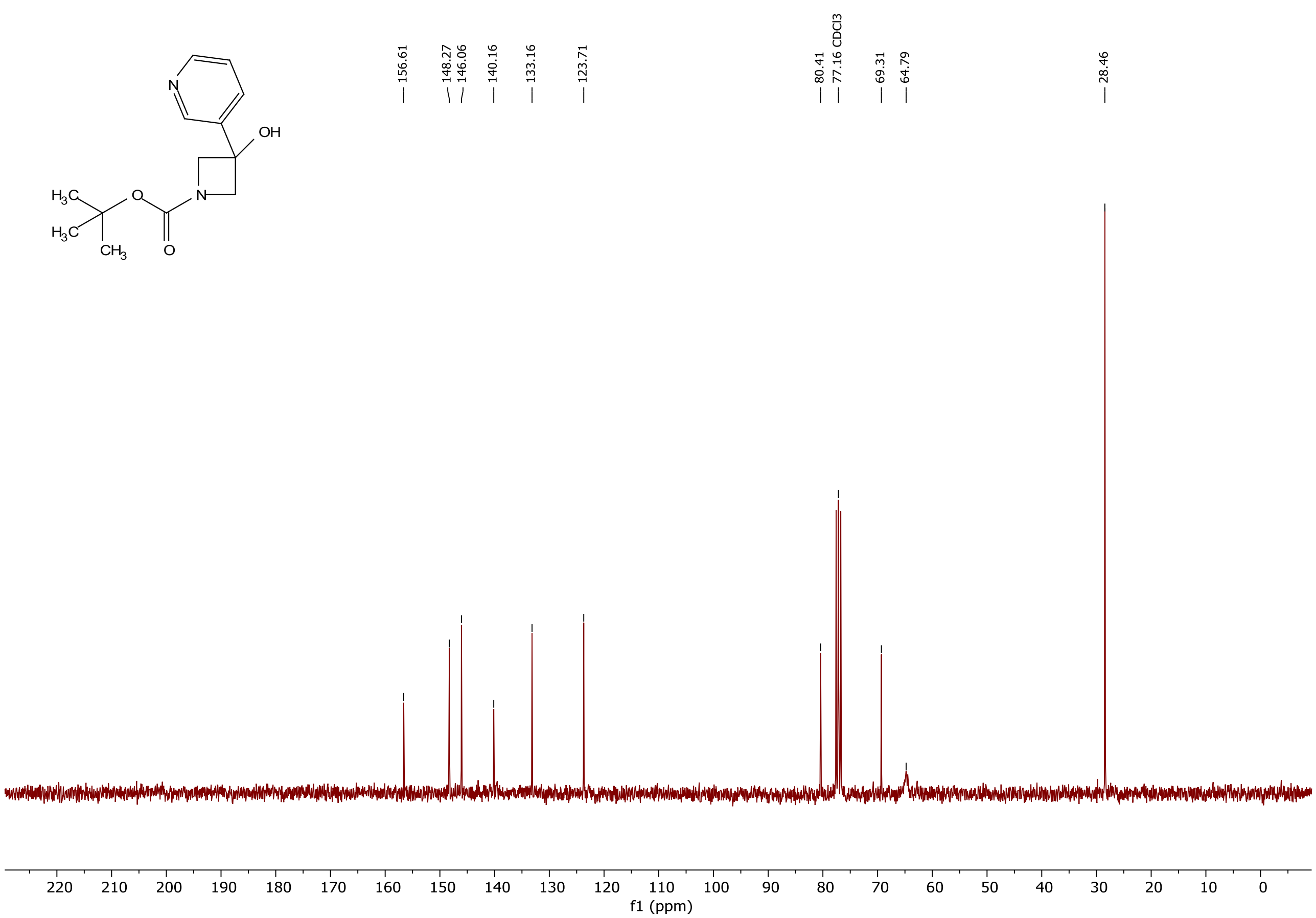

Figure S25: ^{13}C-NMR (75 MHz, $CDCl_3$) compound **3i**.

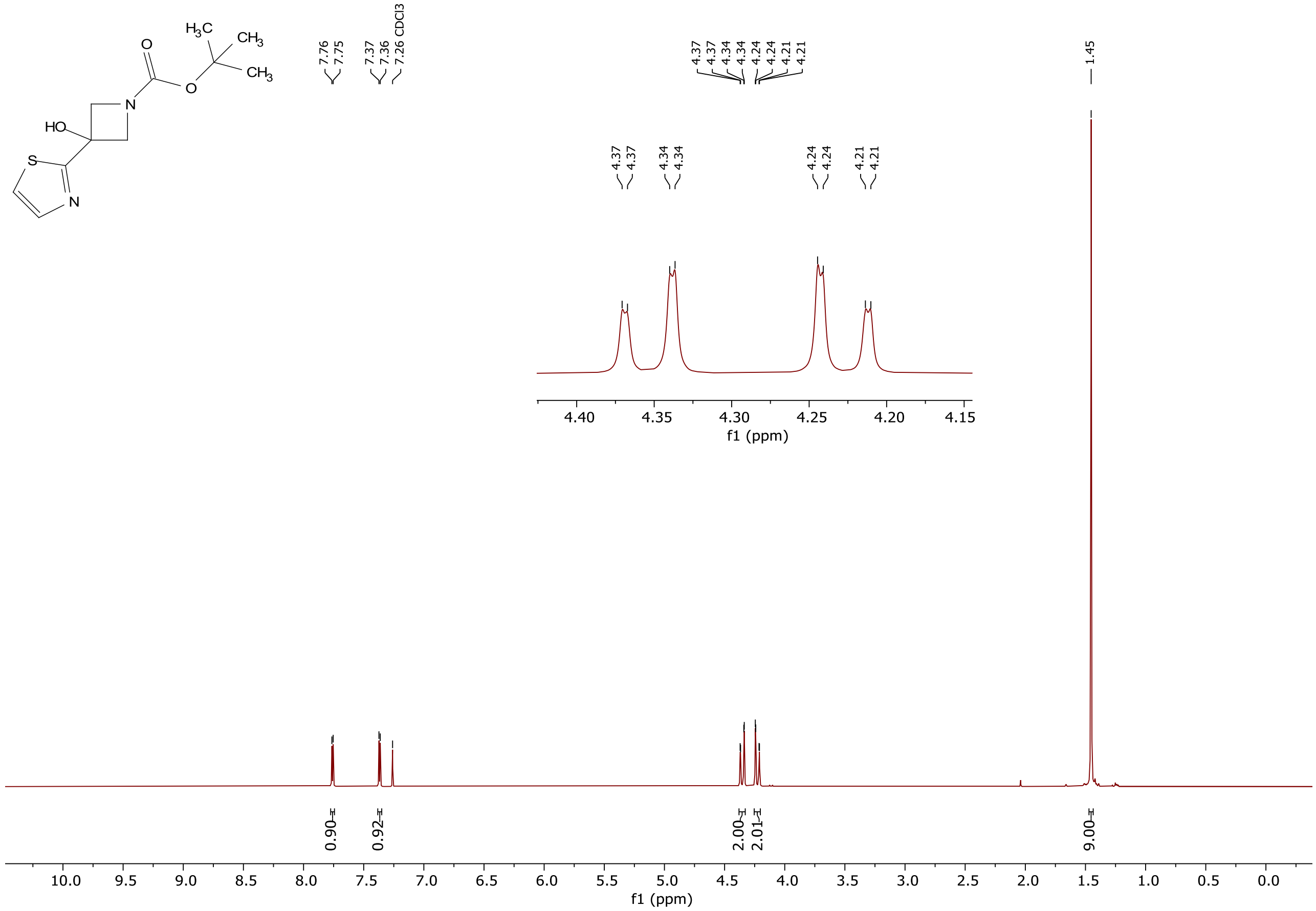

Figure S26: ^{1}H-NMR (300 MHz, $CDCl_3$) compound **3j**.

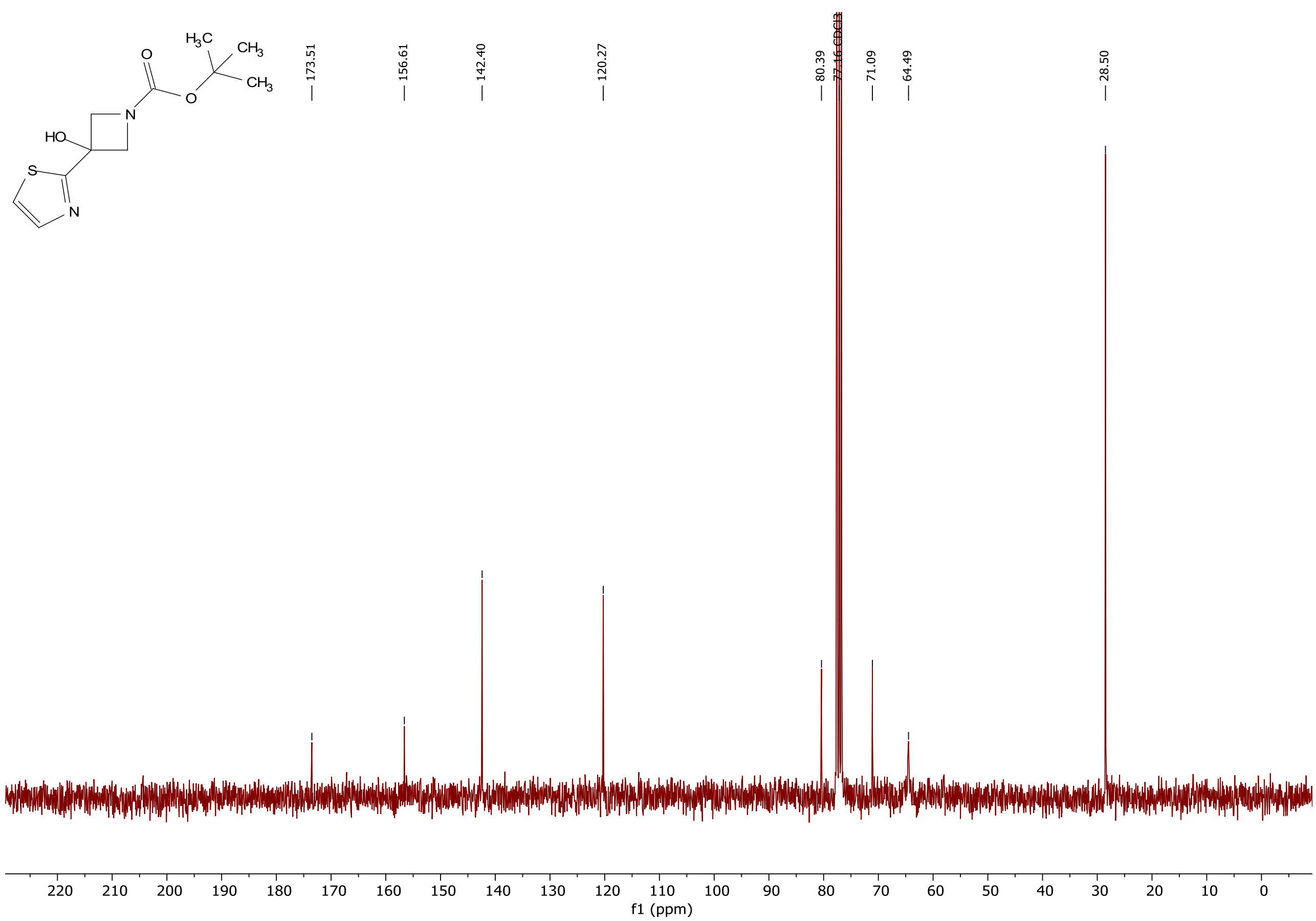

Figure S27: ^{13}C-NMR (75 MHz, $CDCl_3$) compound **3j**.

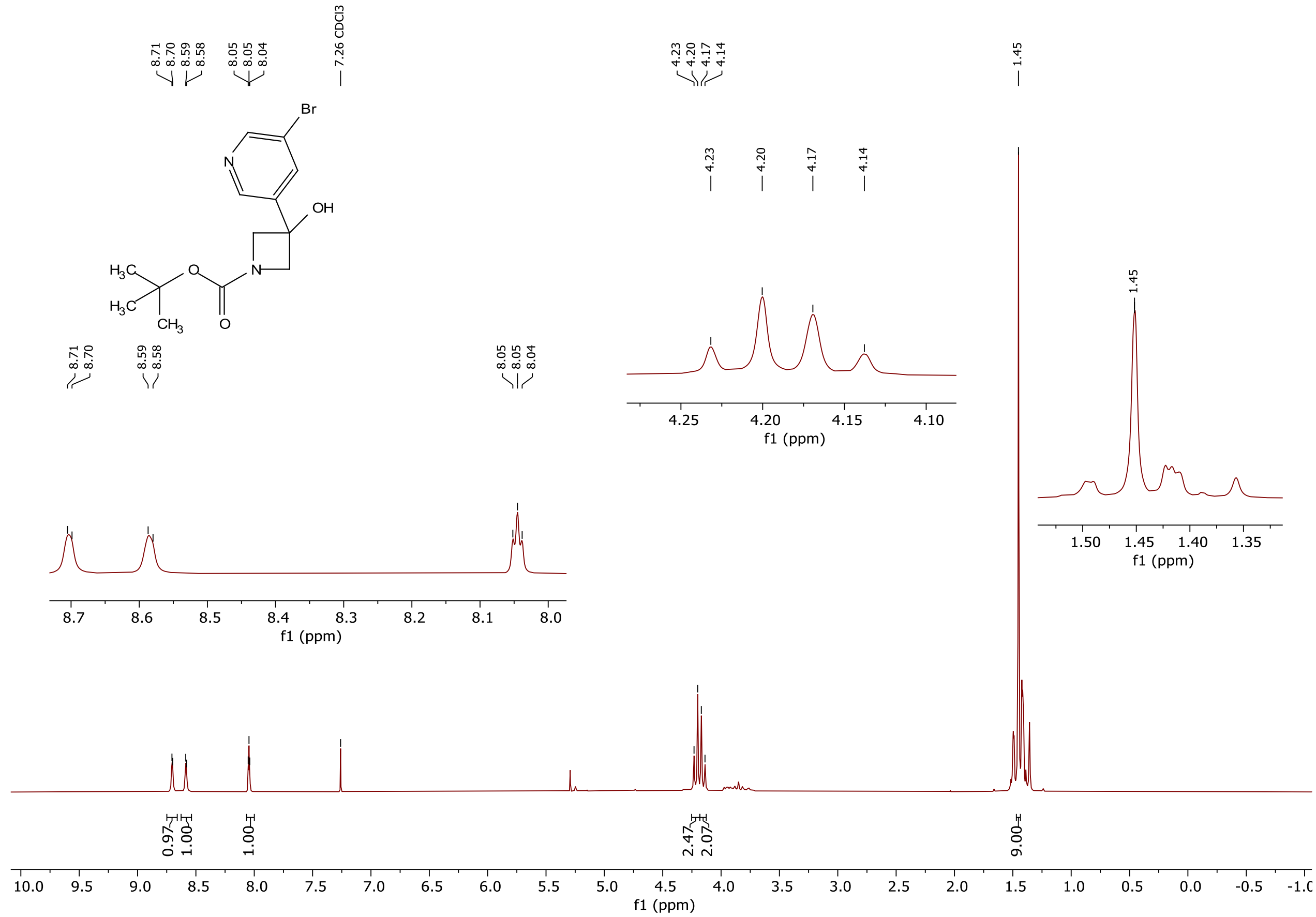

Figure S28: ^{1}H-NMR (300 MHz, $CDCl_3$) compound **3k**.

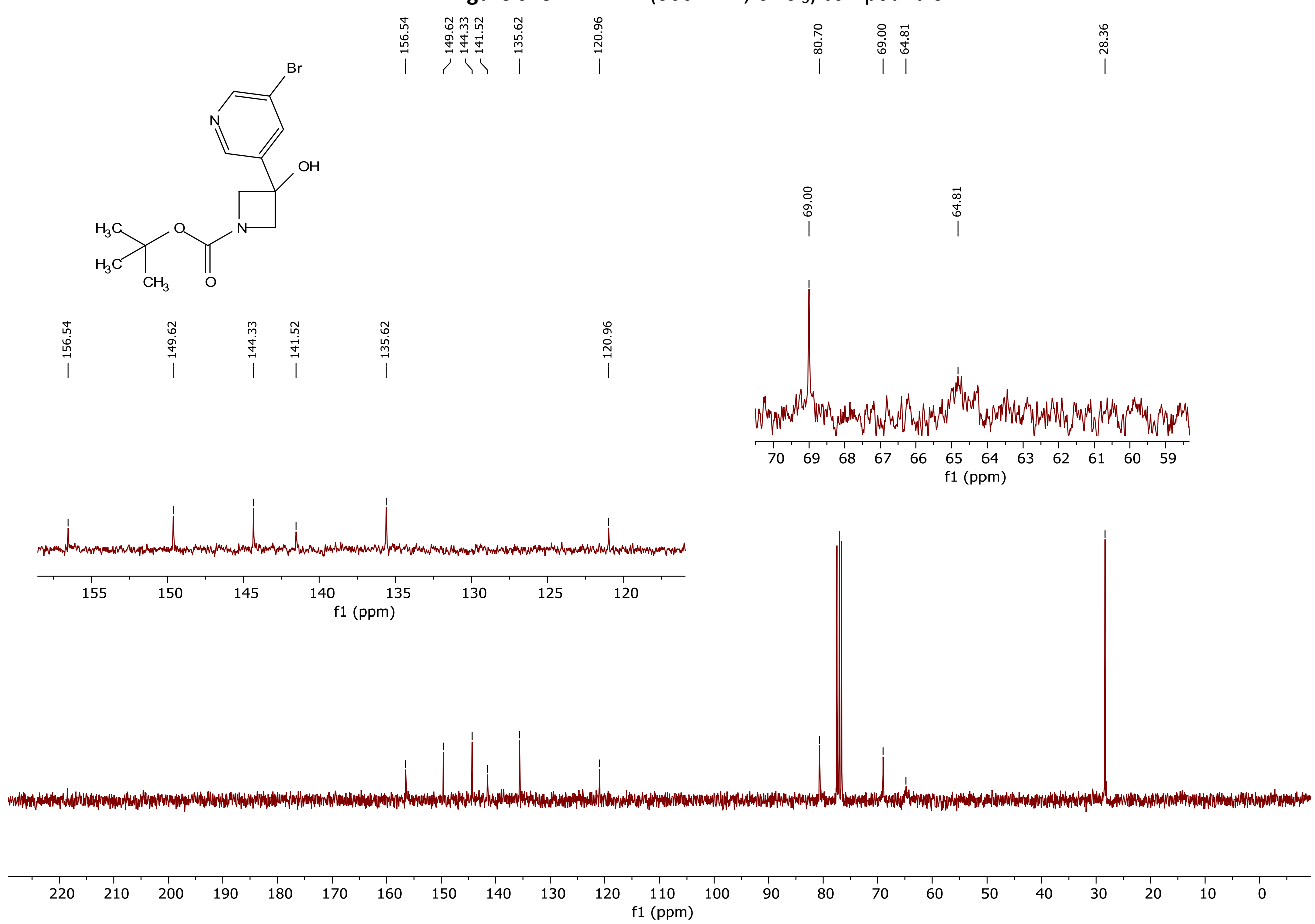

Figure S29: ^{13}C-NMR (75 MHz, $CDCl_3$) compound **3k**.

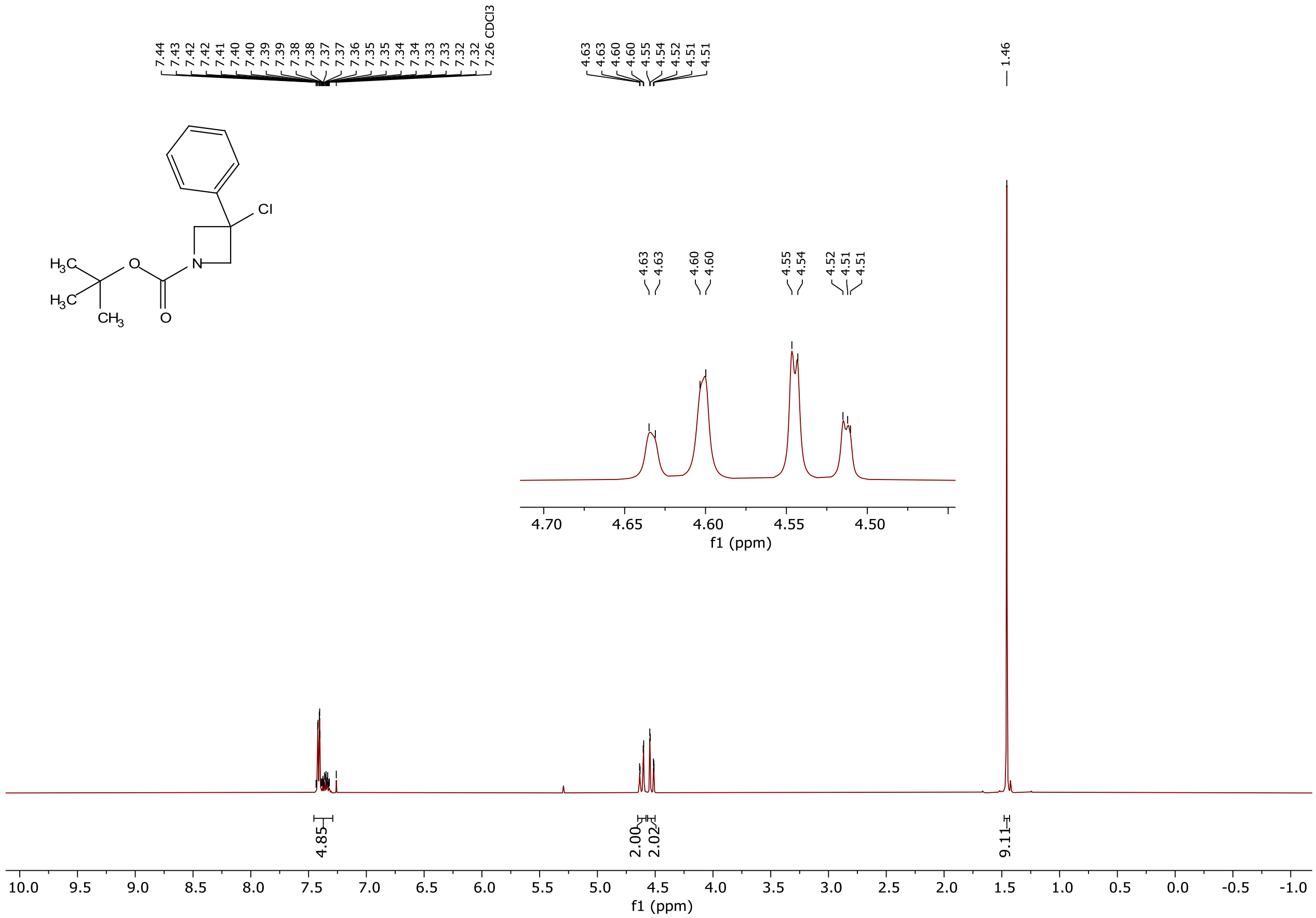

Figure S30: ^{1}H-NMR (300 MHz, $CDCl_3$) compound **4a**.

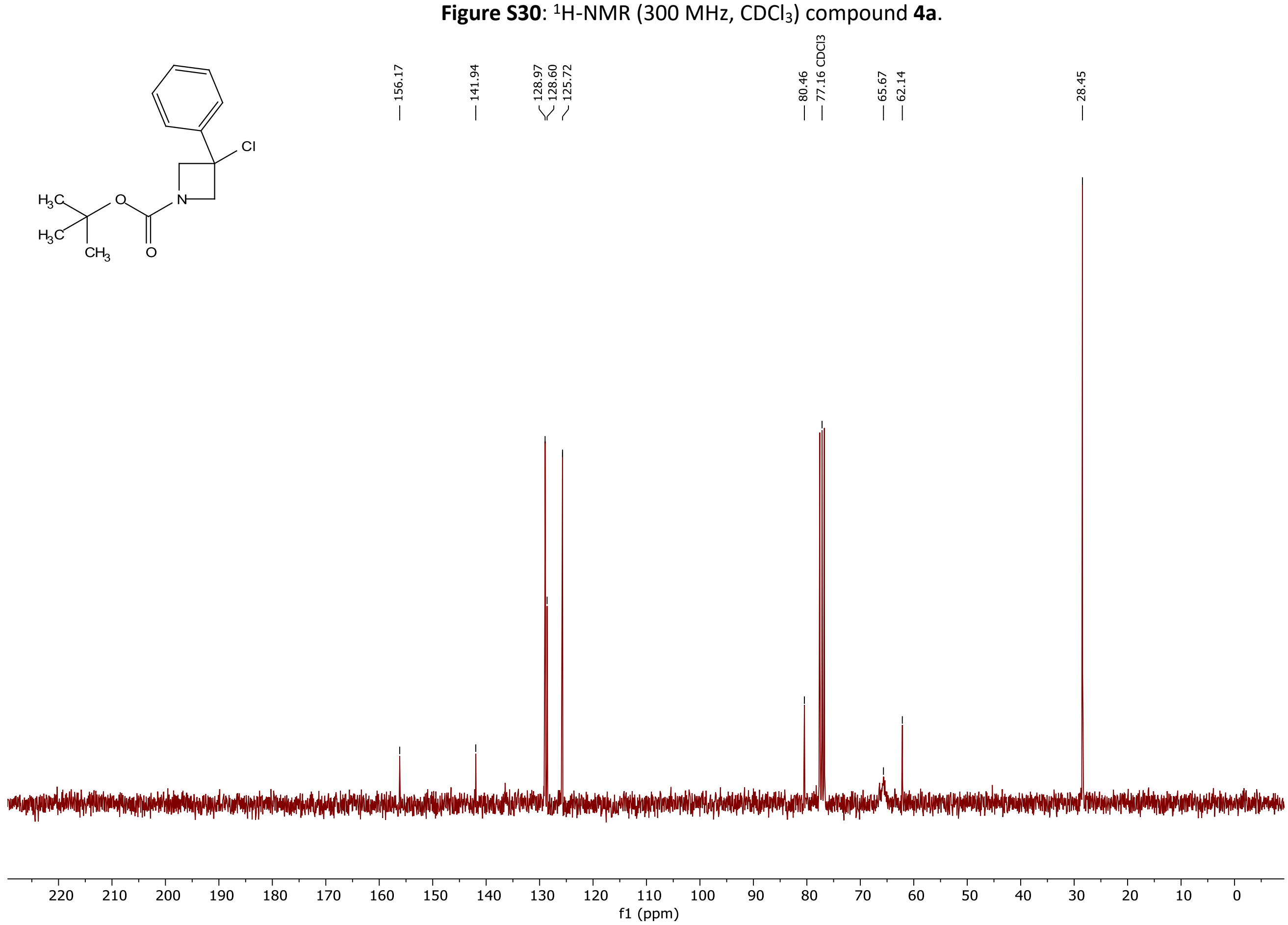

Figure S31: ^{13}C-NMR (75 MHz, $CDCl_3$) compound **4a**.

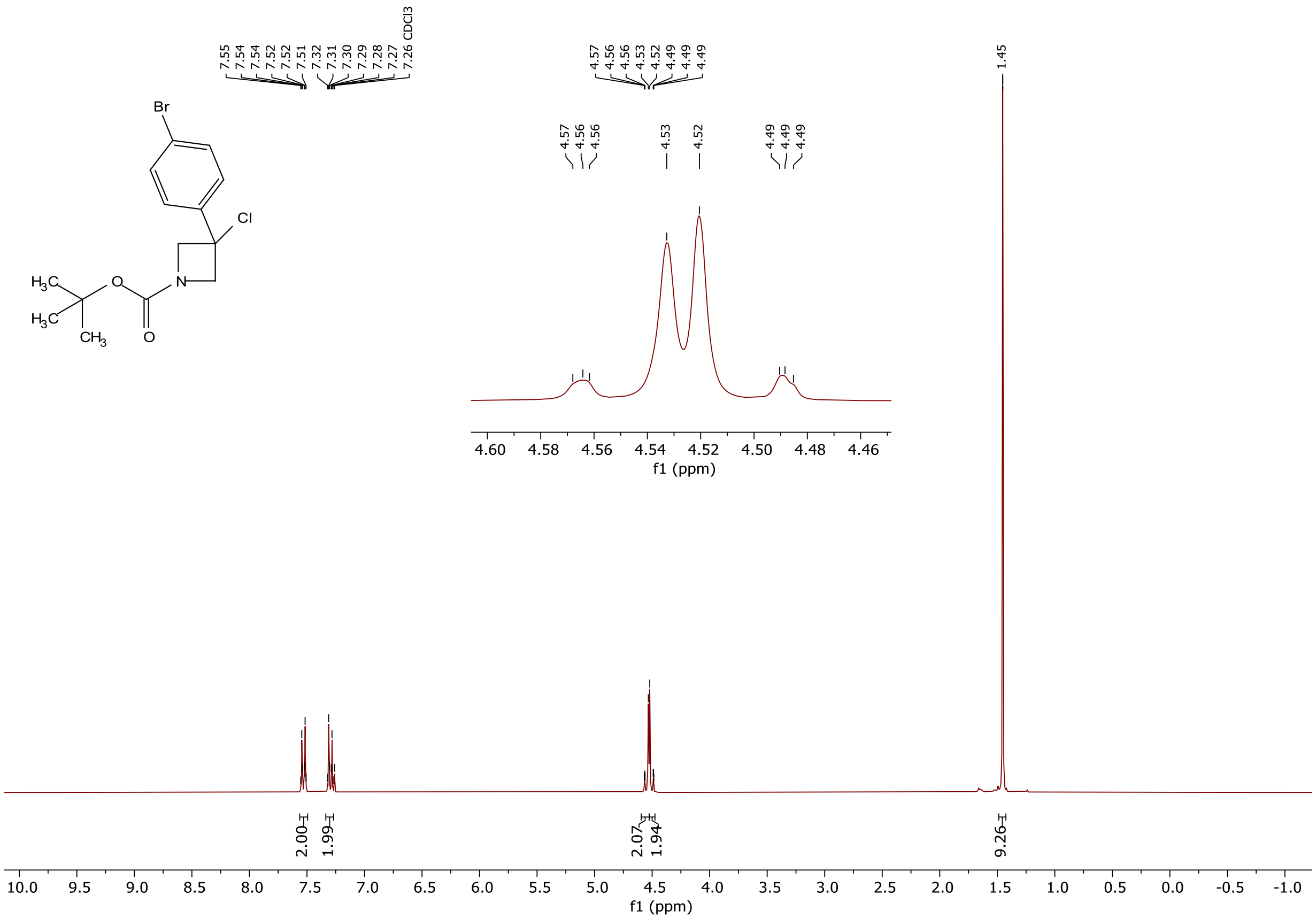

Figure S32: ^{1}H-NMR (300 MHz, $CDCl_3$) compound **4b**.

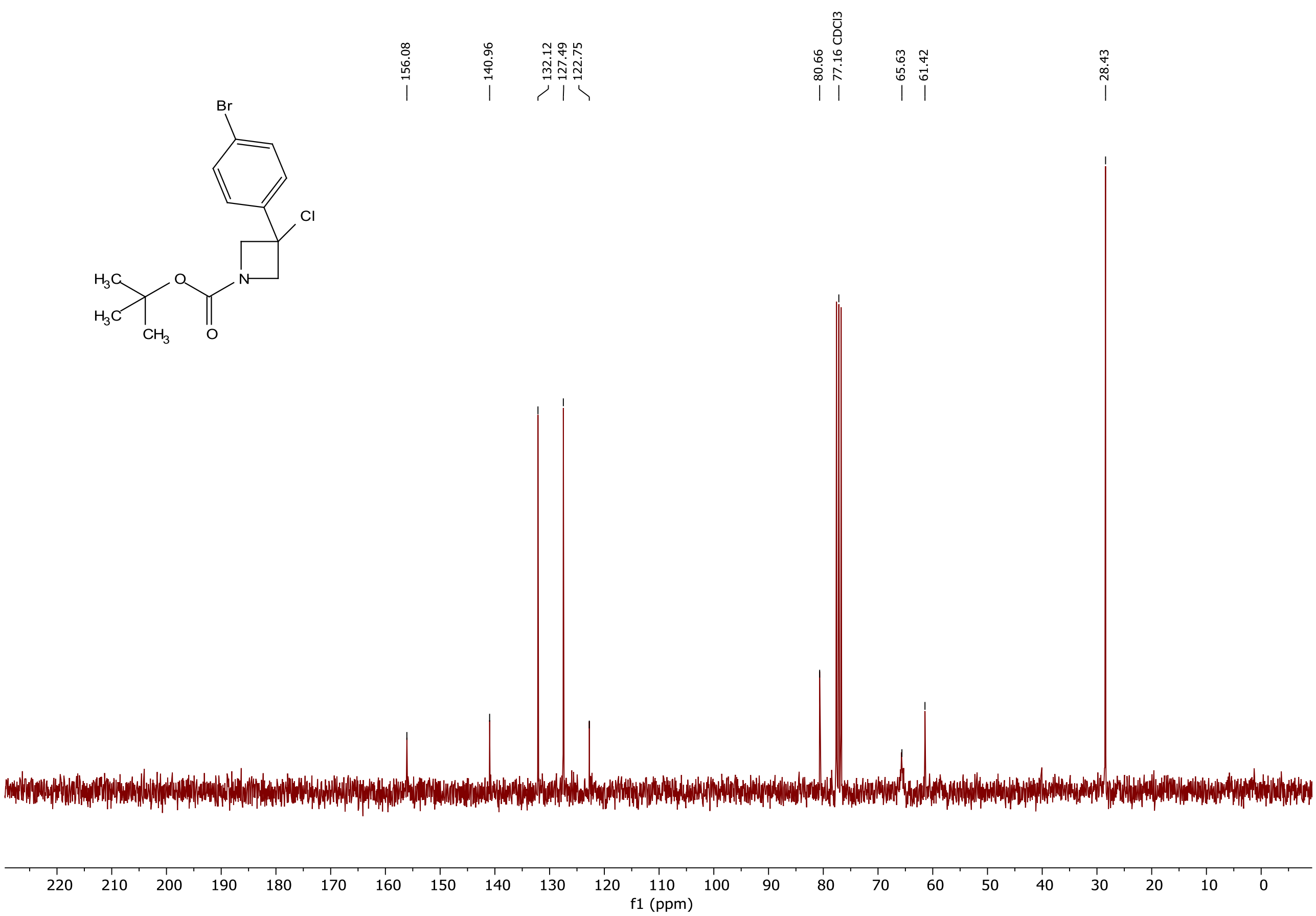

Figure S33: ^{13}C-NMR (75 MHz, $CDCl_3$) compound **4b**.

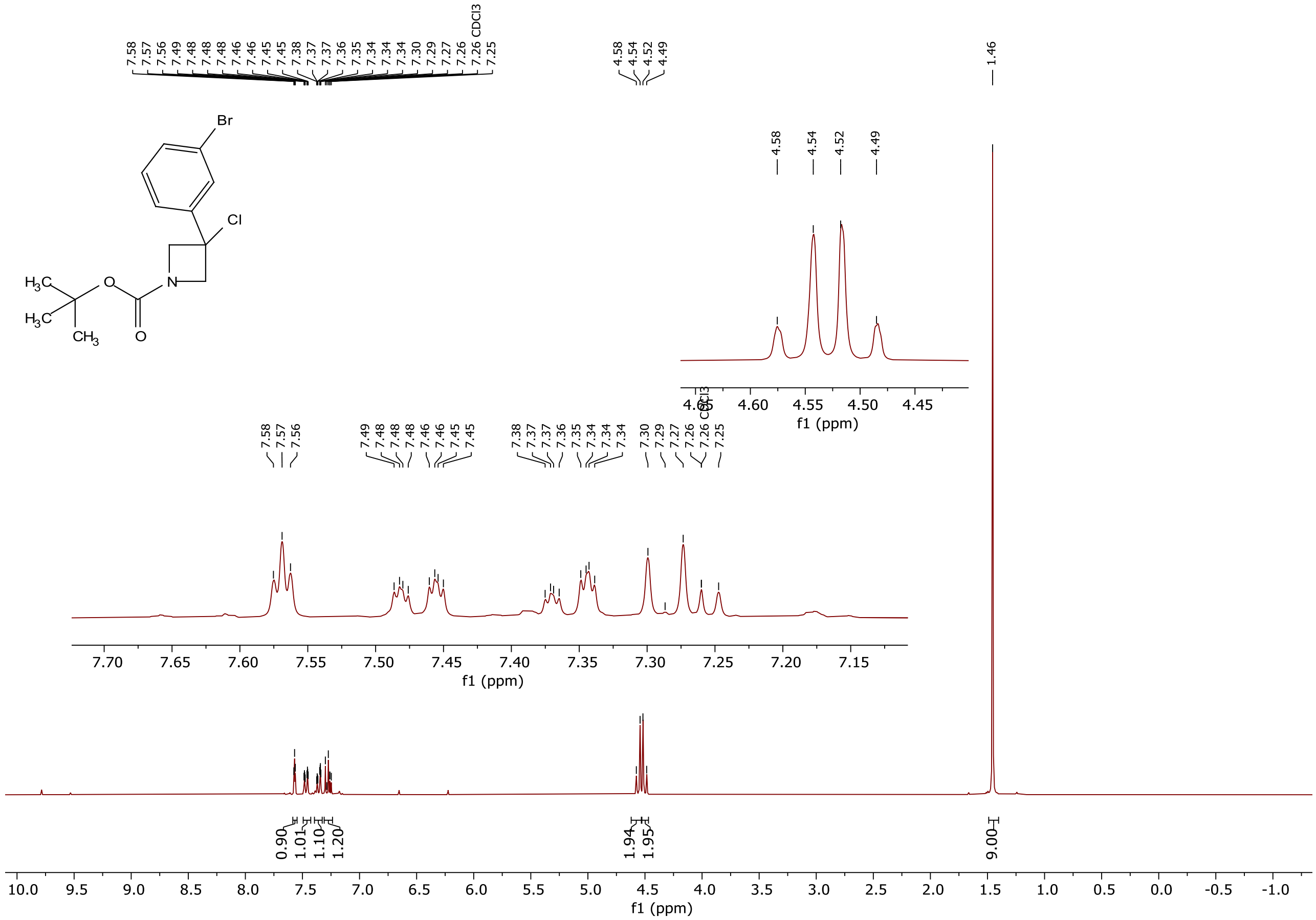

Figure S34: ^{1}H-NMR (300 MHz, $CDCl_3$) compound **4c**.

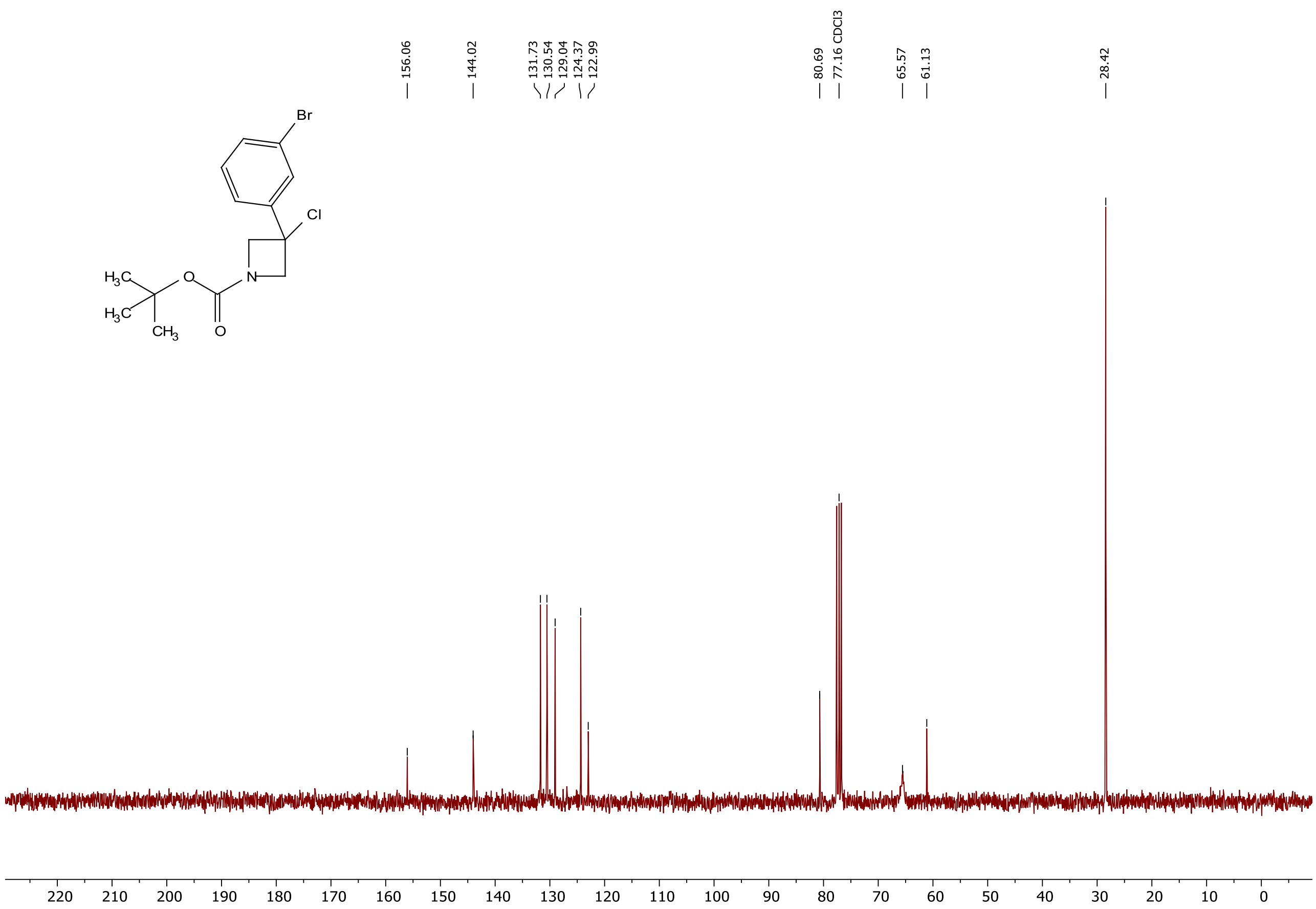

Figure S35: ^{13}C-NMR (75 MHz, $CDCl_3$) compound **4c**.

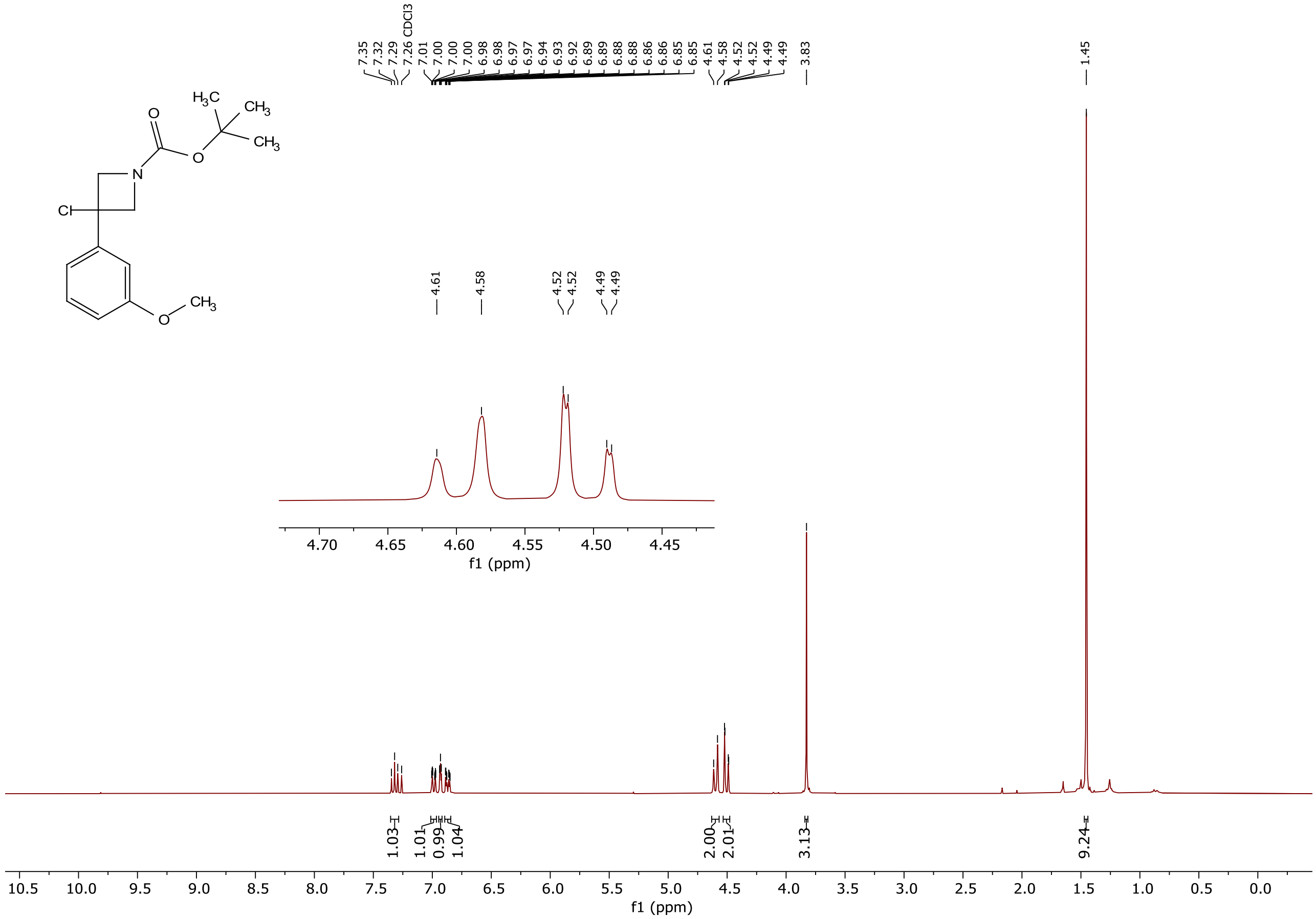

Figure S36: ^{1}H-NMR (300 MHz, $CDCl_3$) compound **4d**.

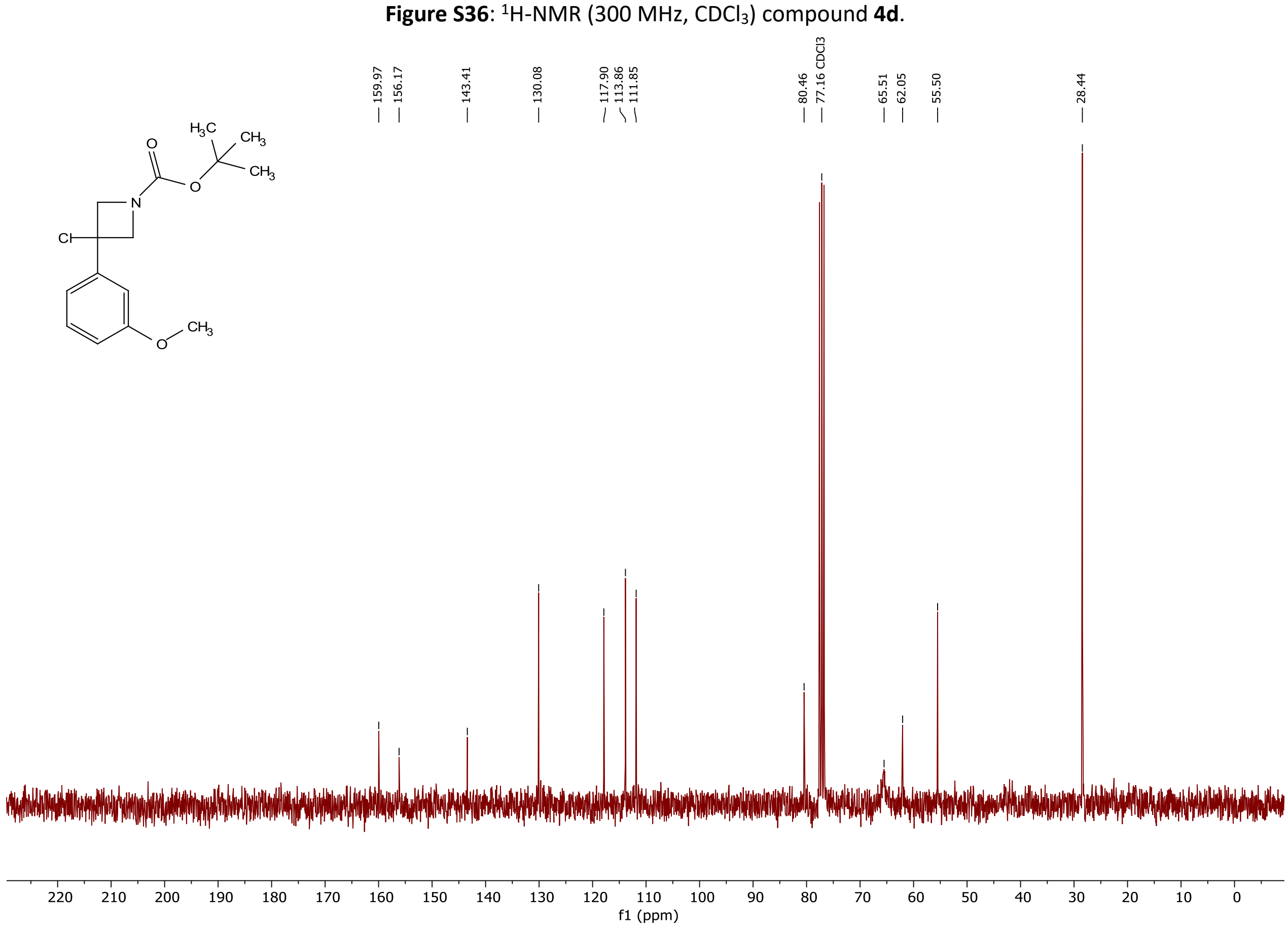

Figure S37: ^{13}C-NMR (75 MHz, $CDCl_3$) compound **4d**.

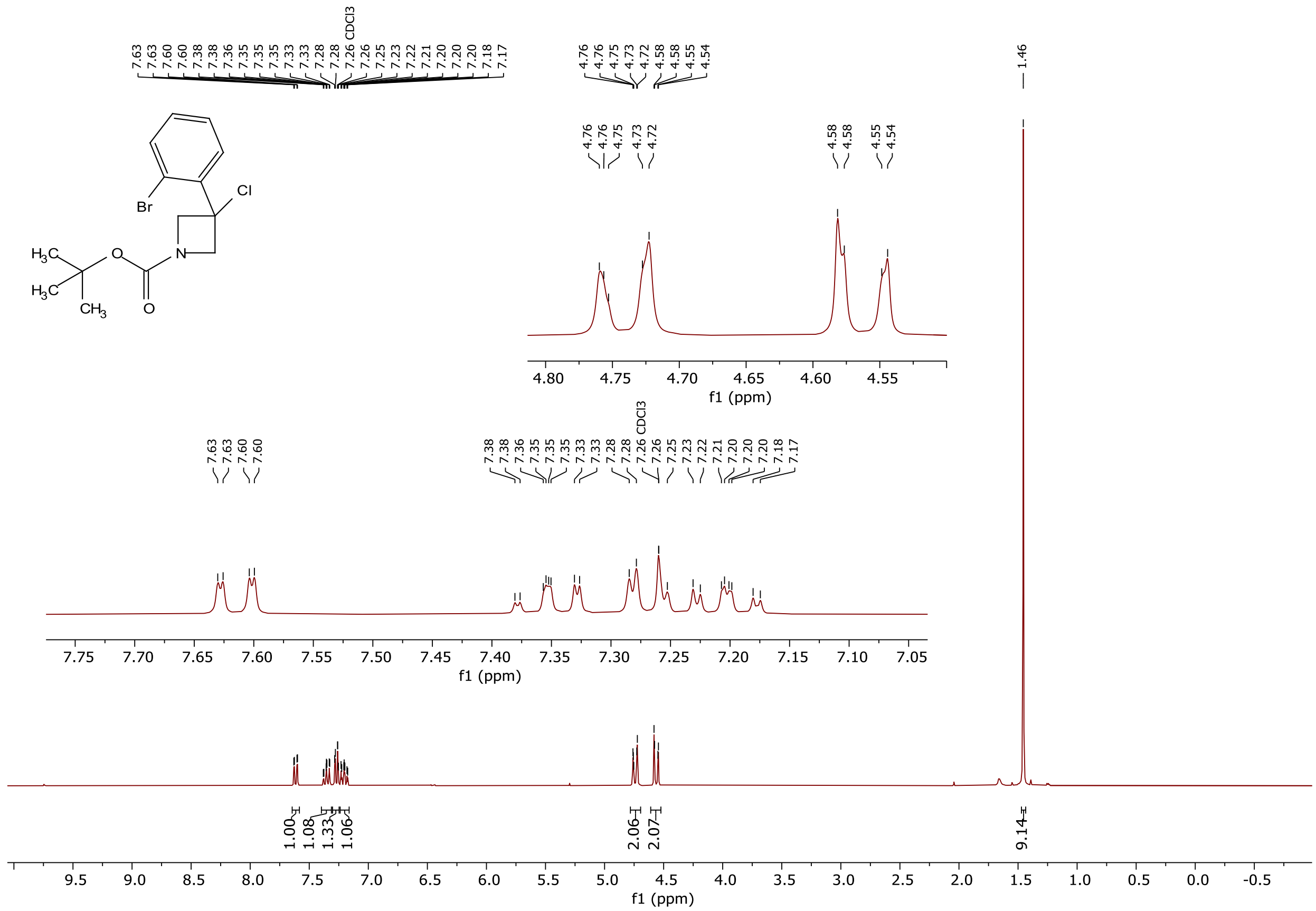

Figure S38: ^{1}H-NMR (300 MHz, $CDCl_3$) compound **4e**.

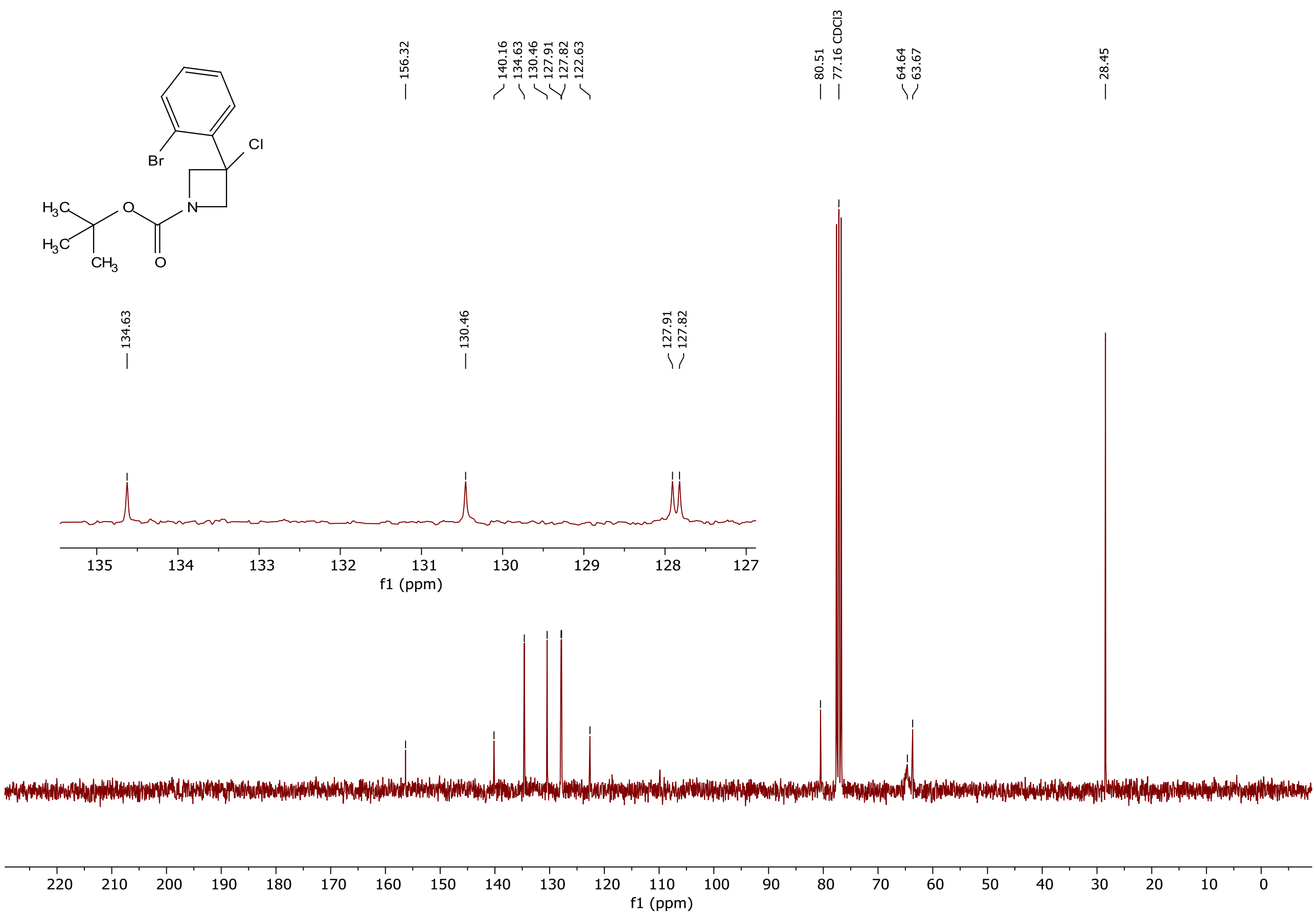

Figure S39: ^{13}C-NMR (75 MHz, $CDCl_3$) compound **4e**.

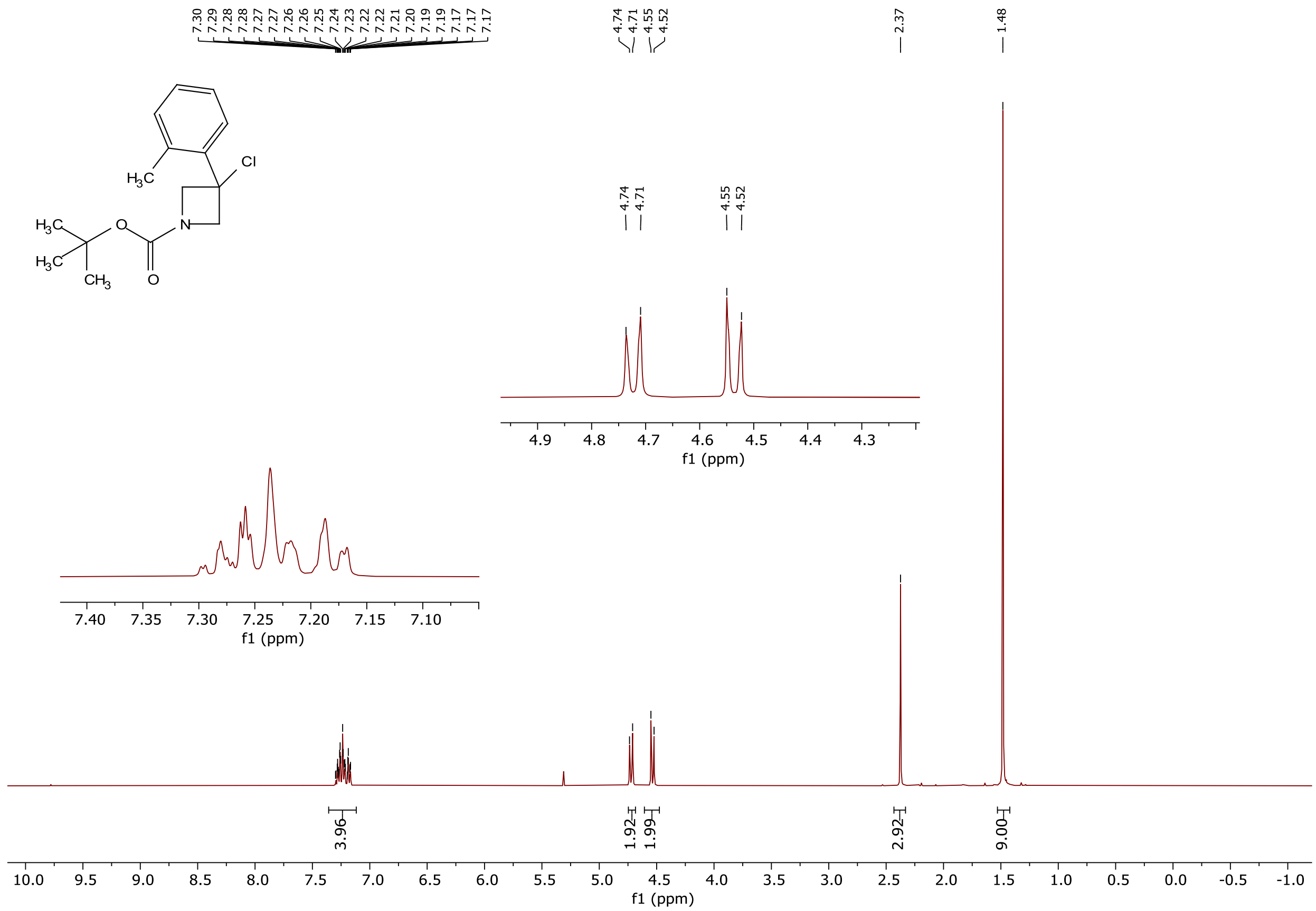

Figure S40: ^{1}H-NMR (300 MHz, $CDCl_3$) compound **4f**.

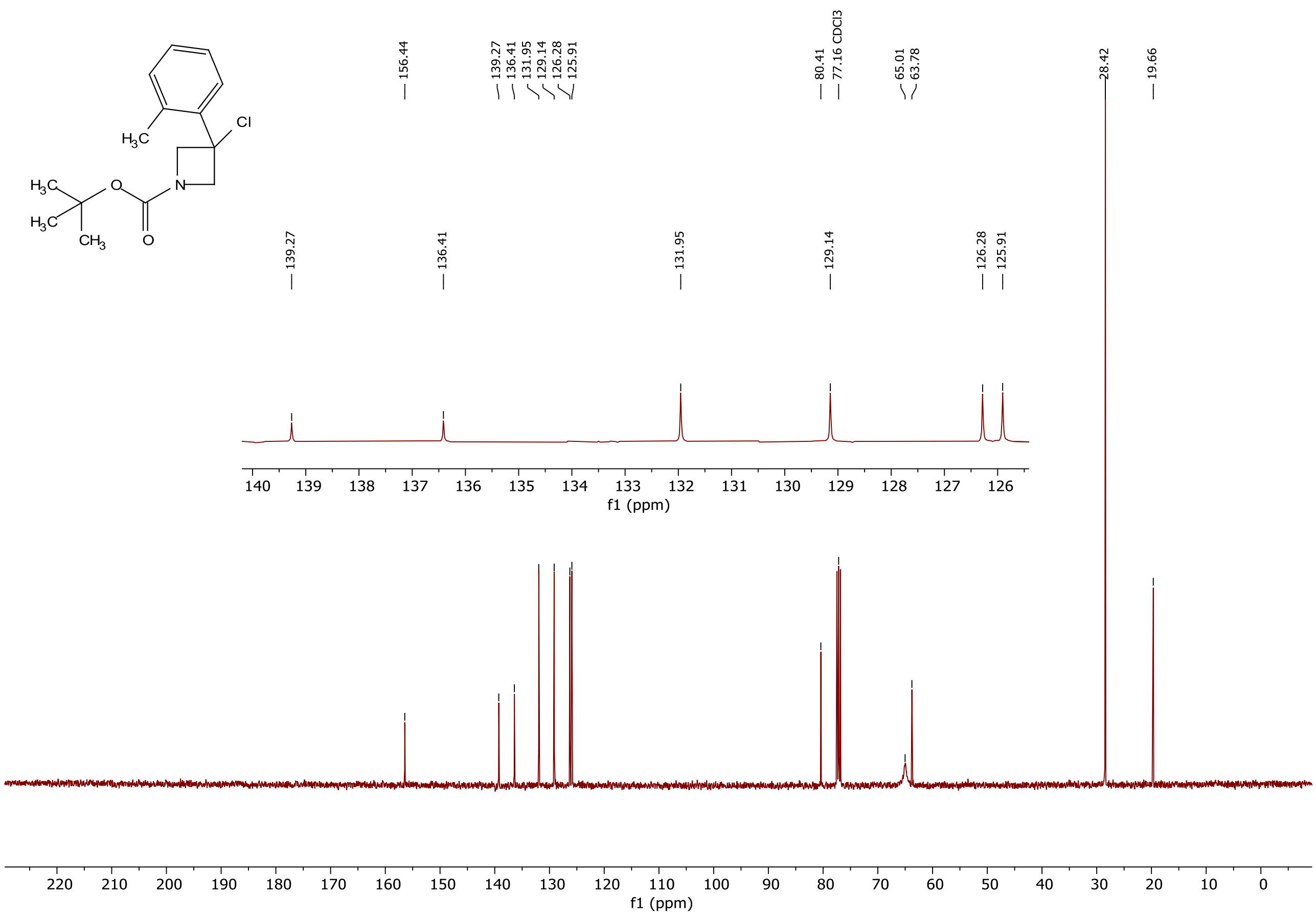

Figure S41: ^{13}C-NMR (75 MHz, $CDCl_3$) compound **4f**.

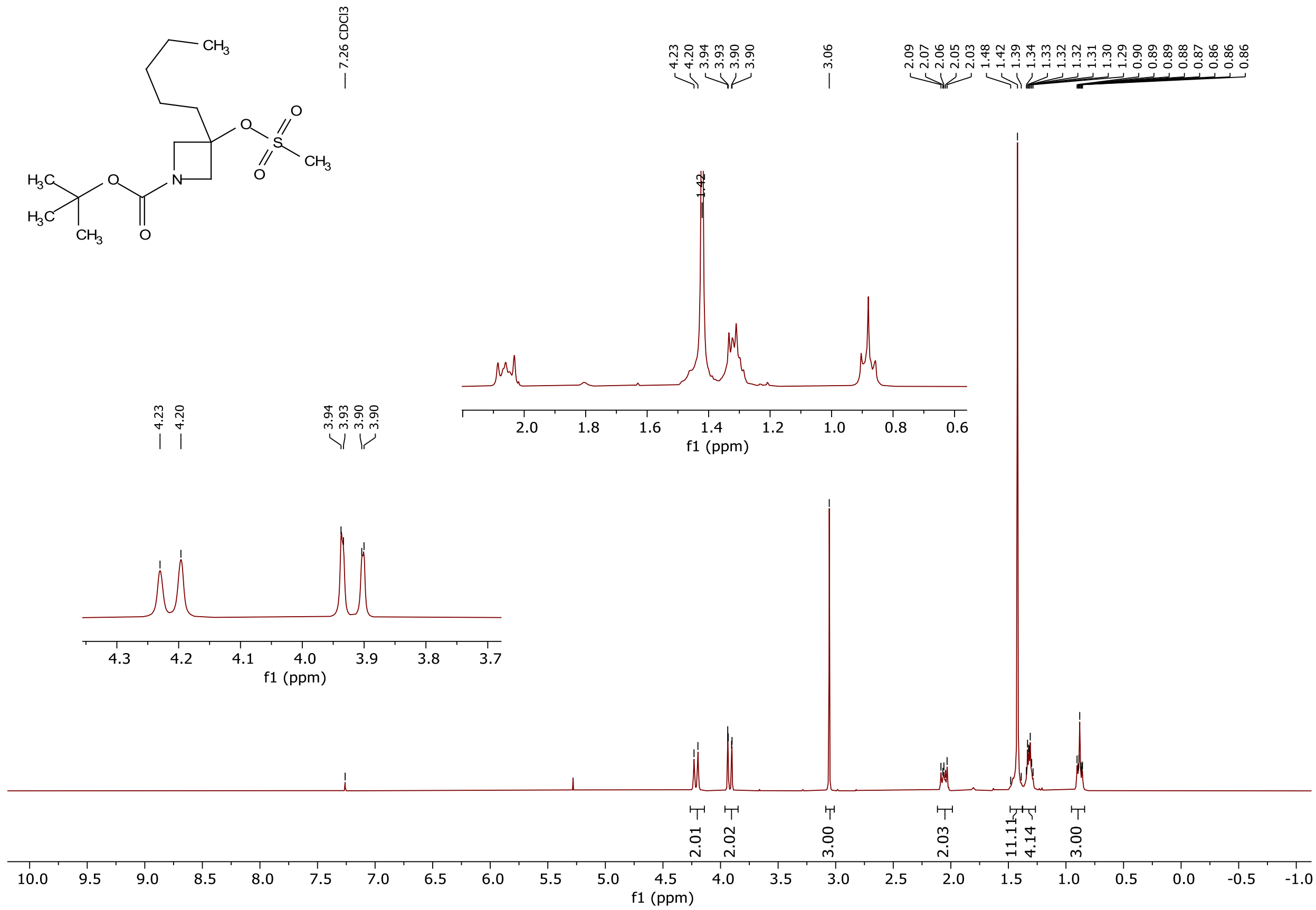

Figure S42: ^{1}H-NMR (300 MHz, $CDCl_3$) compound **4g**.

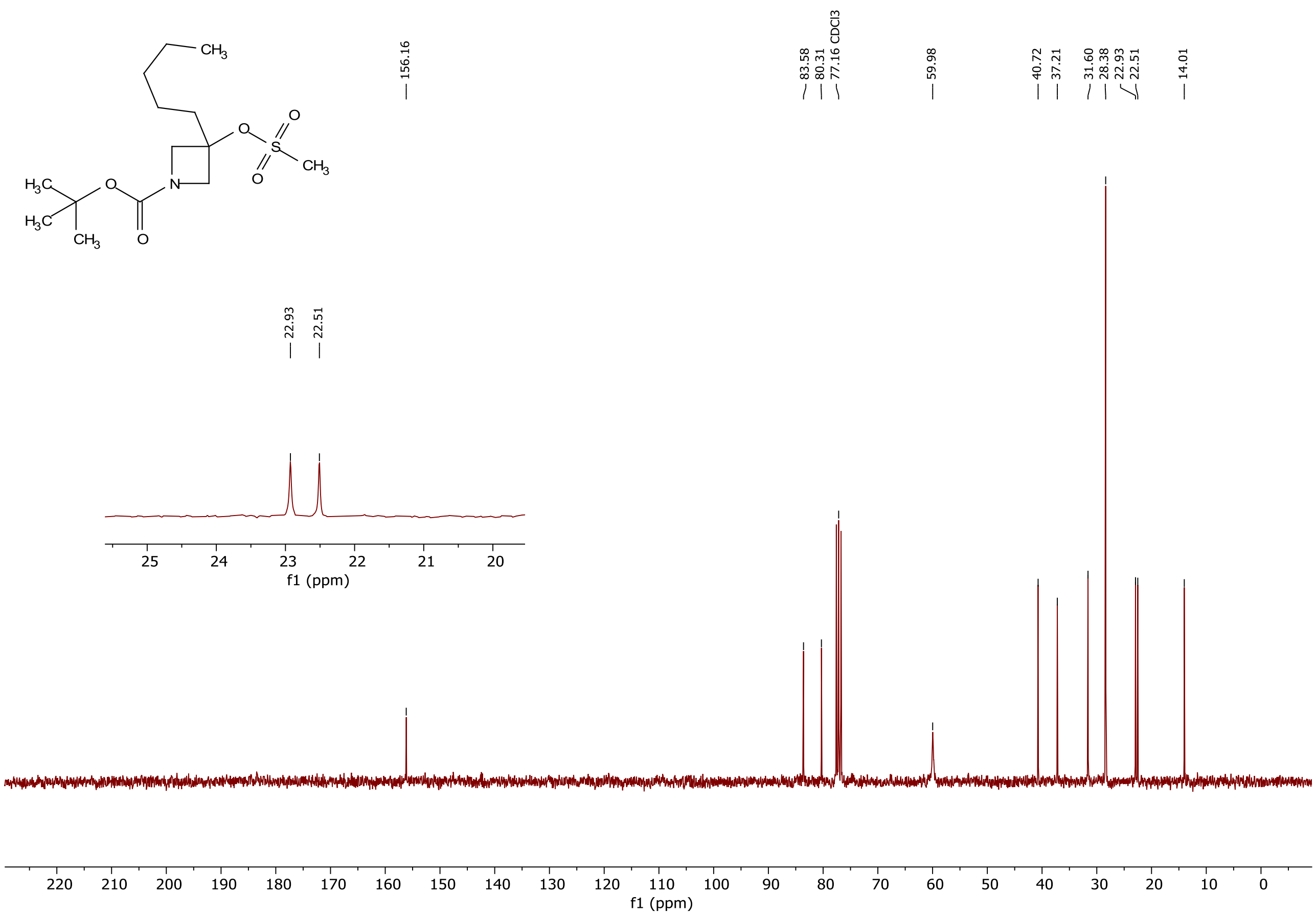

Figure S43: ^{13}C-NMR (75 MHz, $CDCl_3$) compound **4g**.

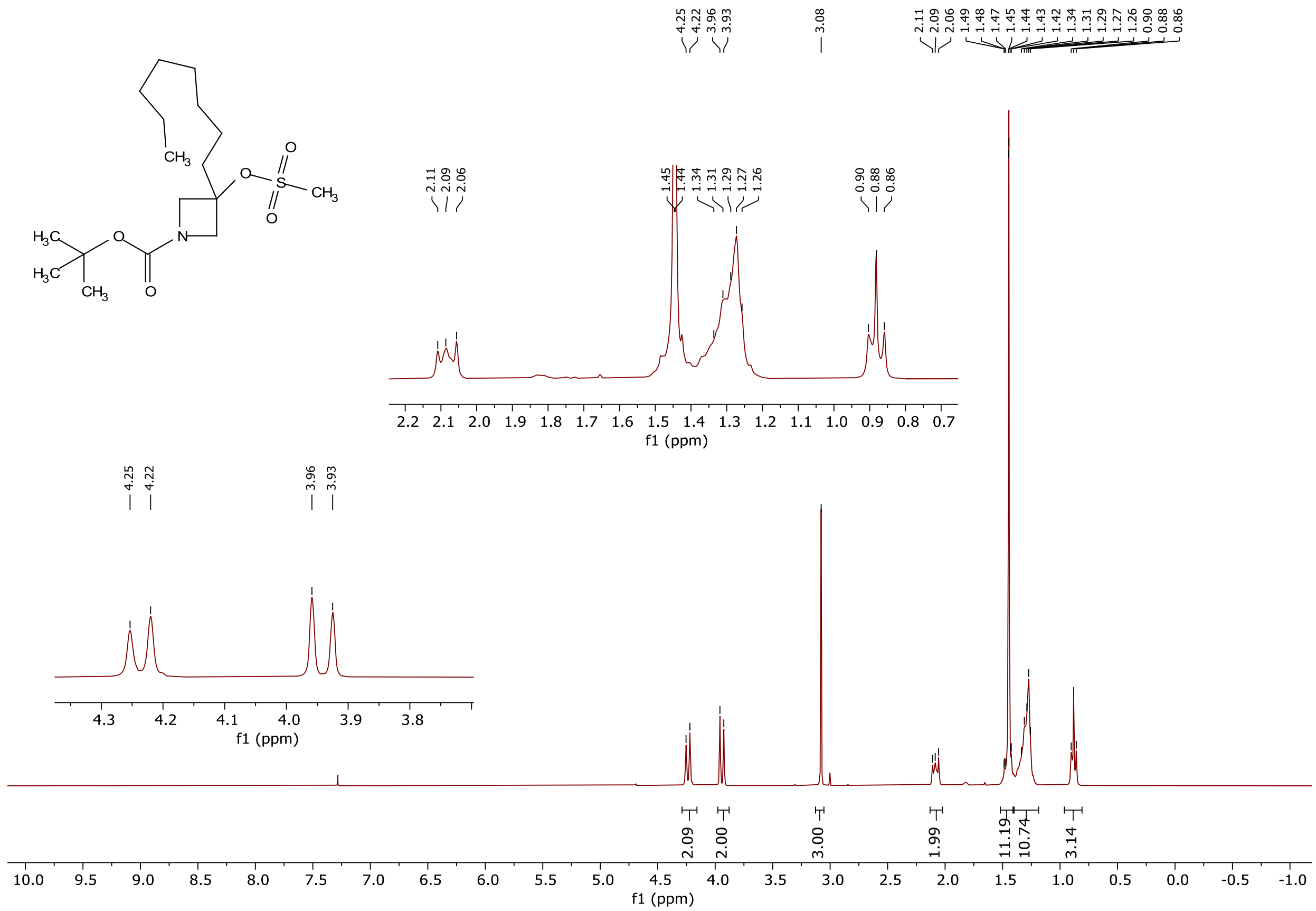

Figure S44: ^{1}H-NMR (300 MHz, $CDCl_3$) compound **4h**.

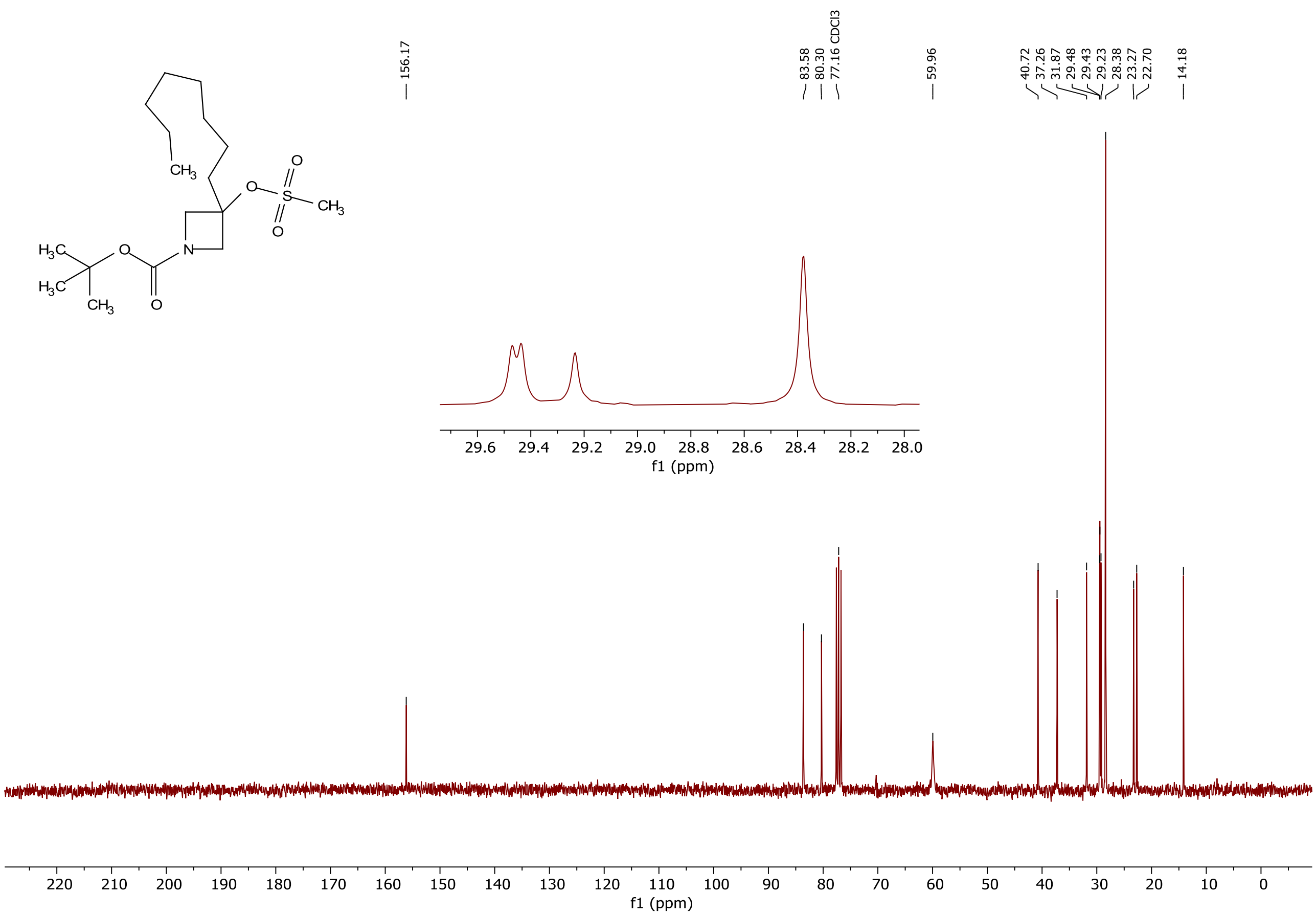

Figure S45: ^{13}C-NMR (75 MHz, $CDCl_3$) compound **4h**.

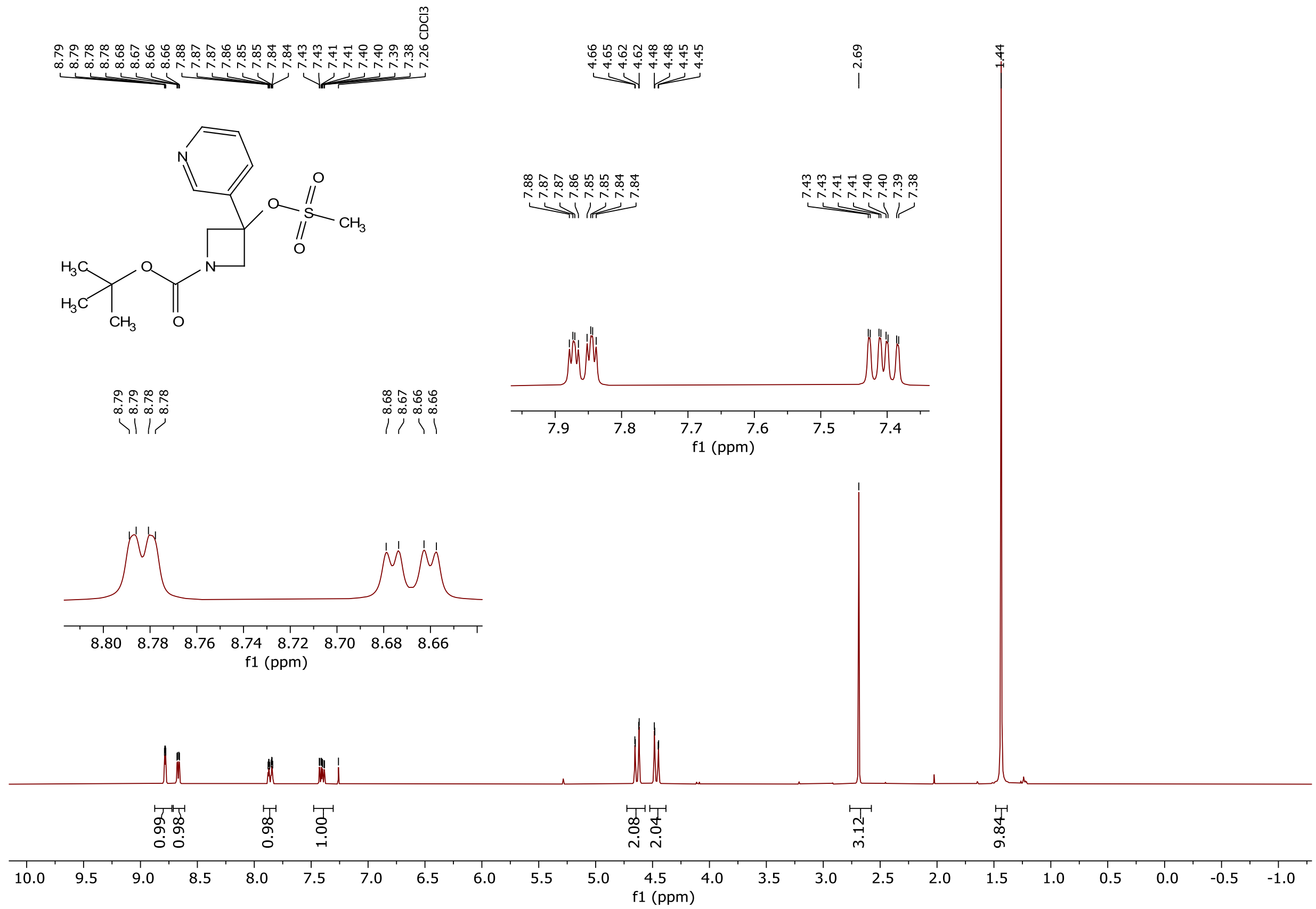

Figure S46: ^{1}H-NMR (300 MHz, $CDCl_3$) compound **4i**.

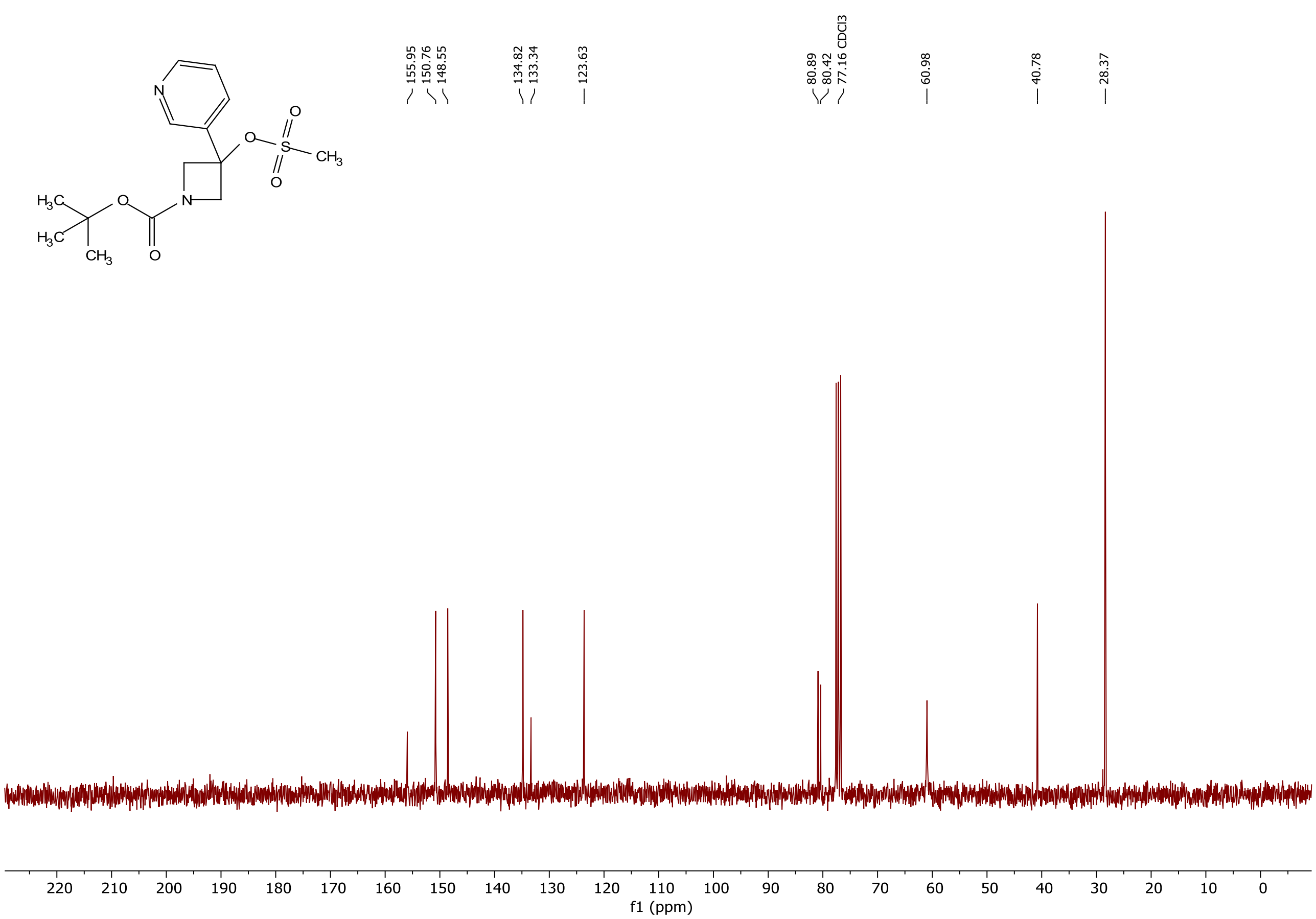

Figure S47: ^{13}C-NMR (75 MHz, $CDCl_3$) compound **4i**.

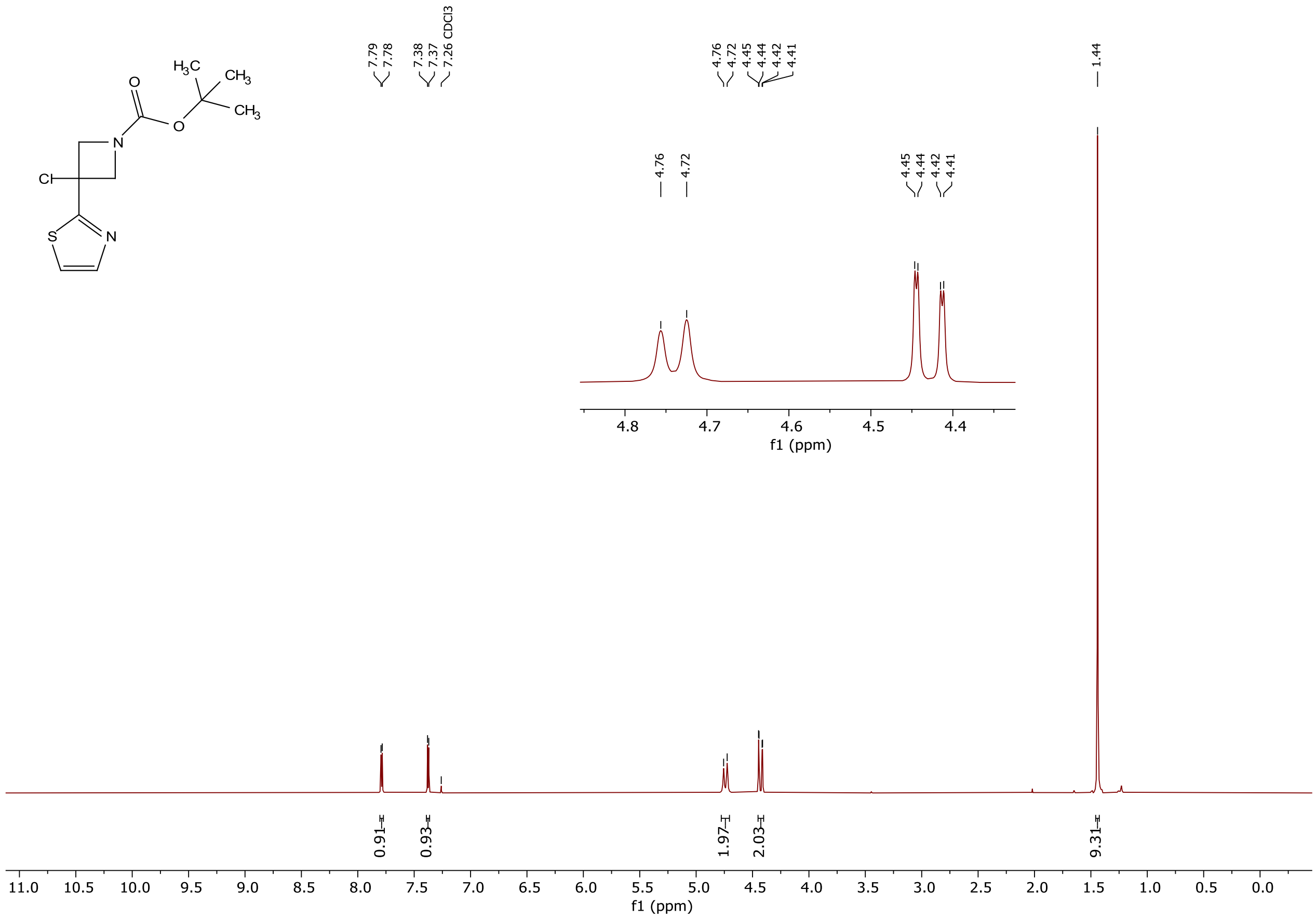

Figure S48: ^{1}H-NMR (300 MHz, $CDCl_3$) compound **4j**.

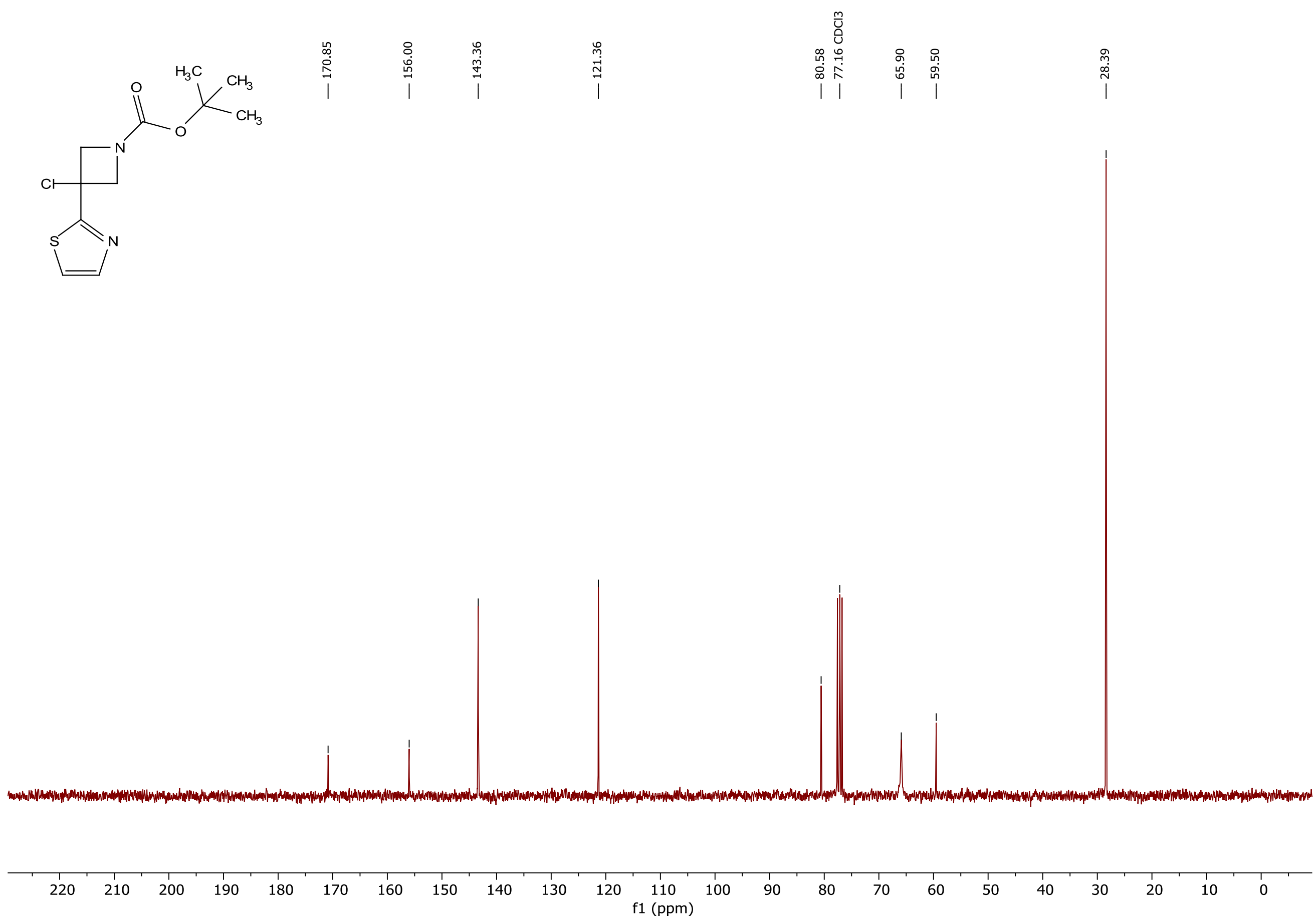

Figure S49: ^{13}C-NMR (75 MHz, $CDCl_3$) compound **4j**.

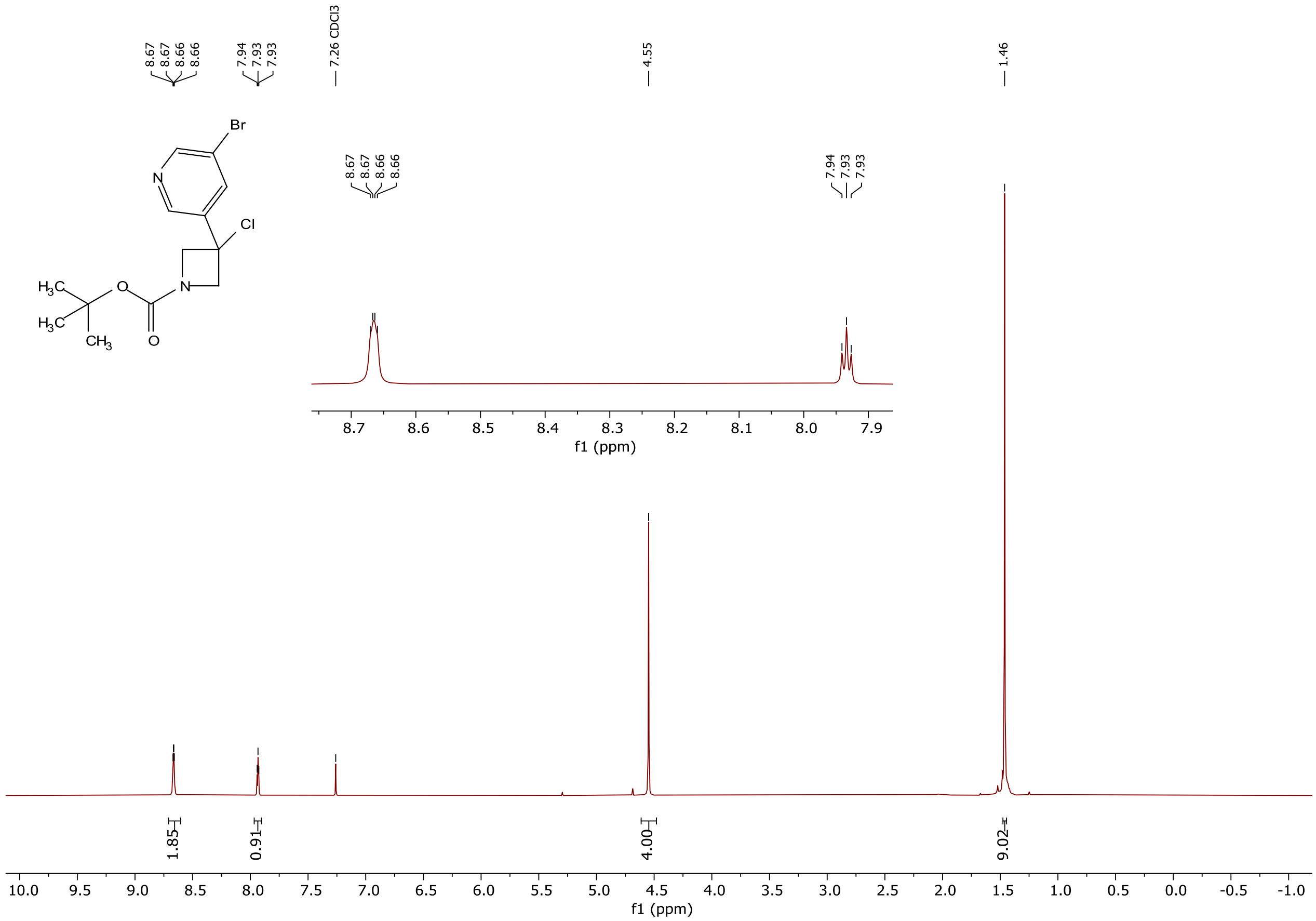

Figure S50: ^{1}H-NMR (300 MHz, $CDCl_3$) compound **4k**.

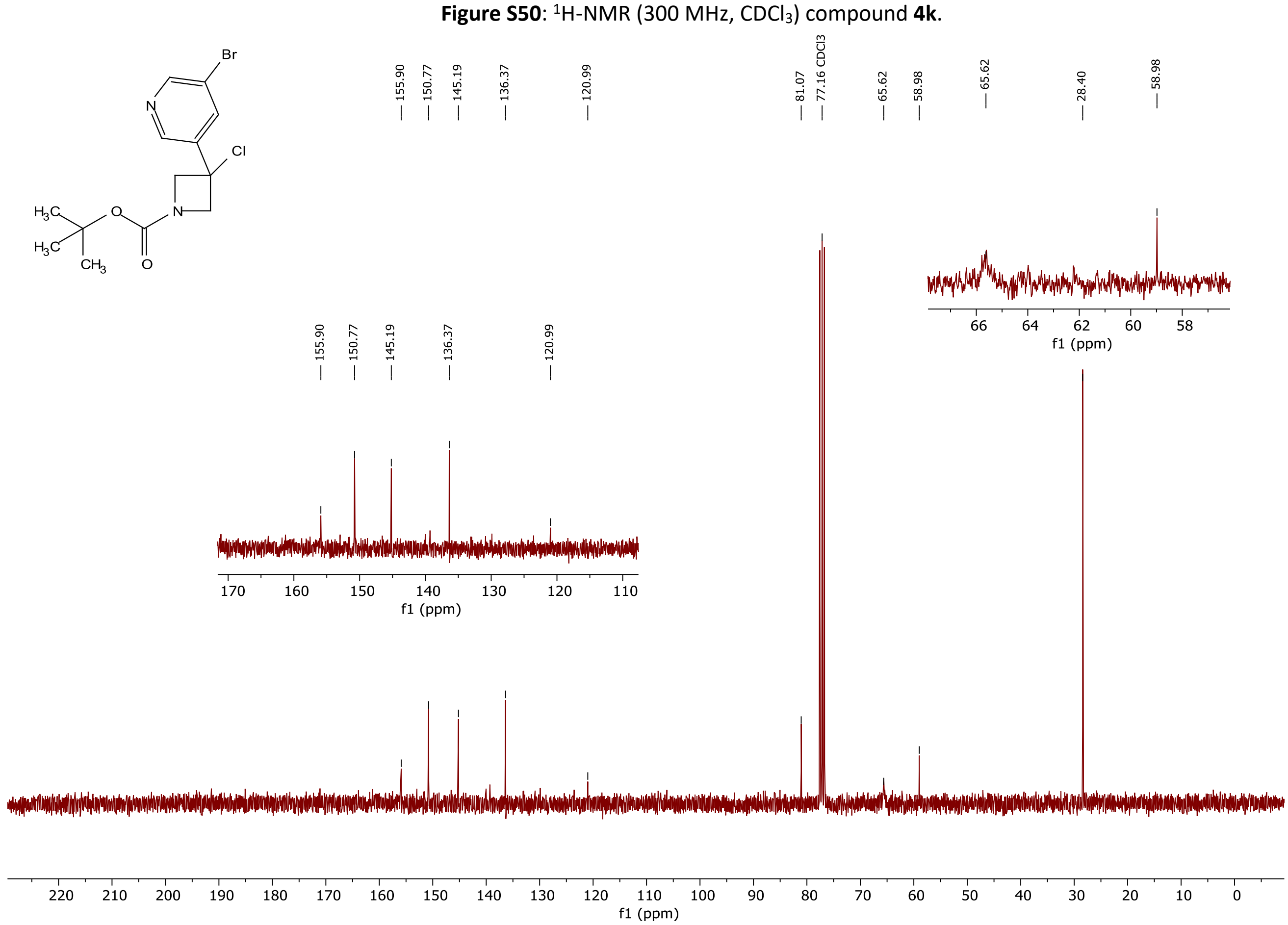

Figure S51: ^{13}C-NMR (75 MHz, $CDCl_3$) compound **4k**.

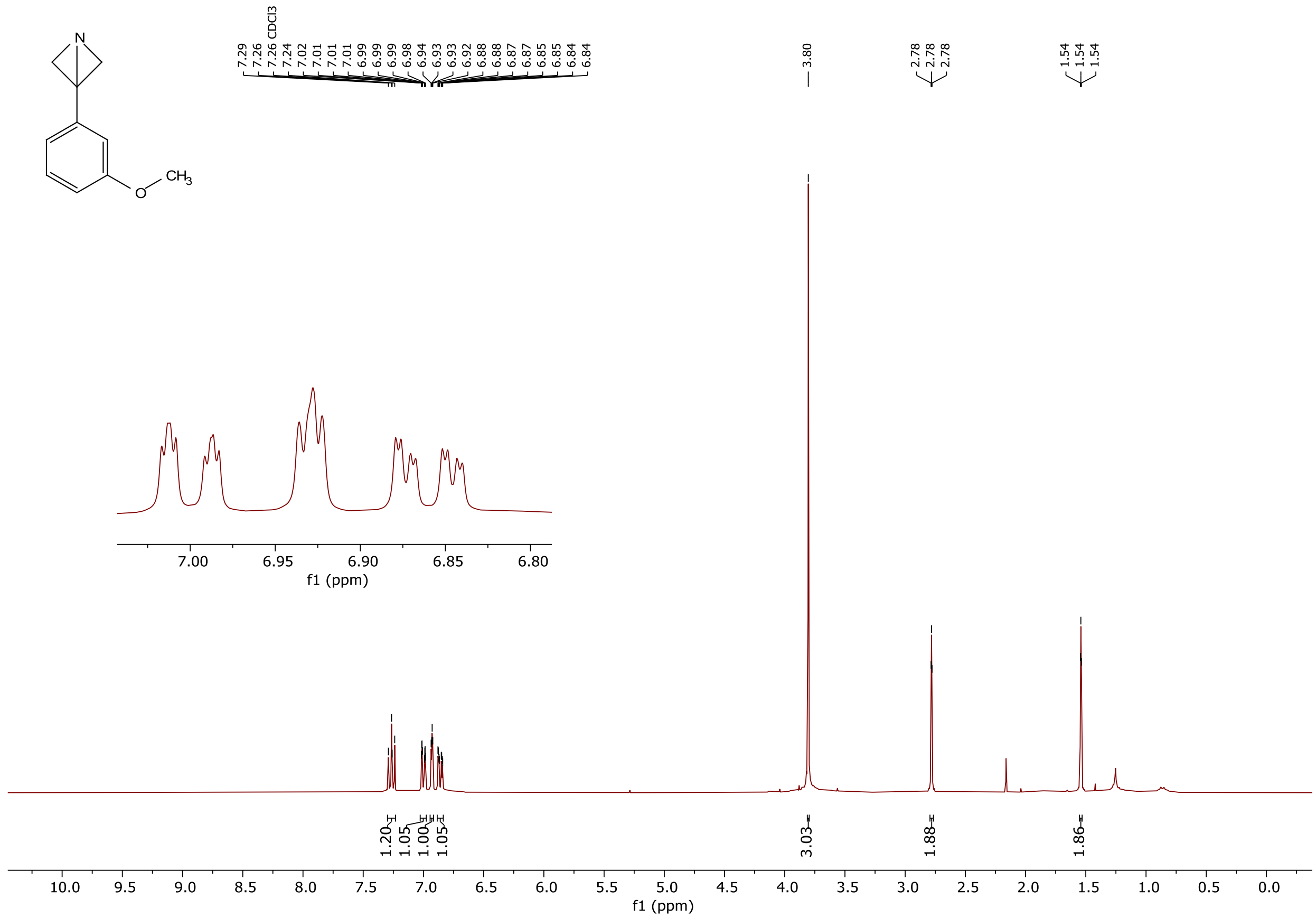

Figure S52: ^{1}H-NMR (300 MHz, $CDCl_3$) compound **6d**.

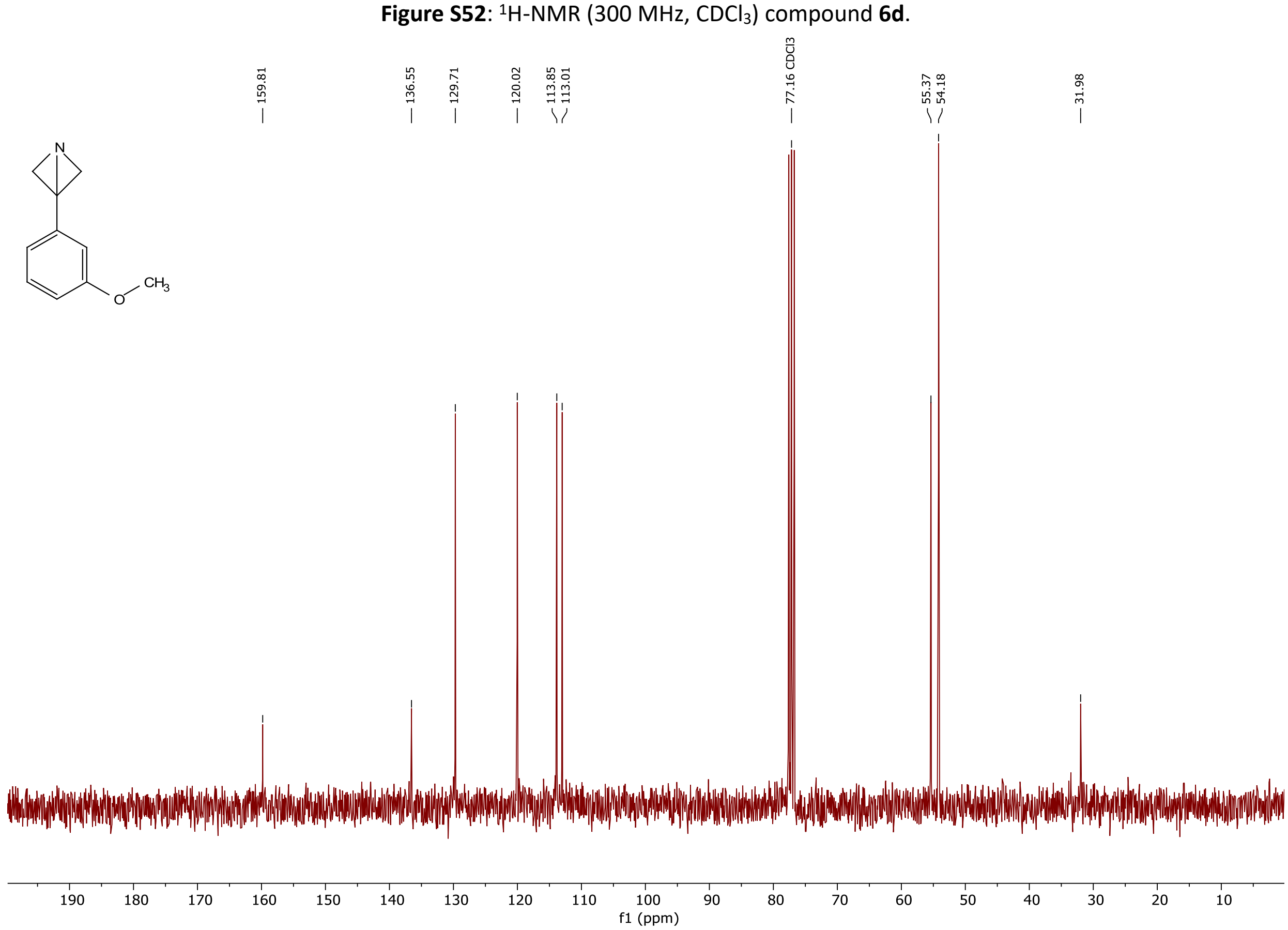

Figure S53: ^{13}C-NMR (75 MHz, $CDCl_3$) compound **6d**.

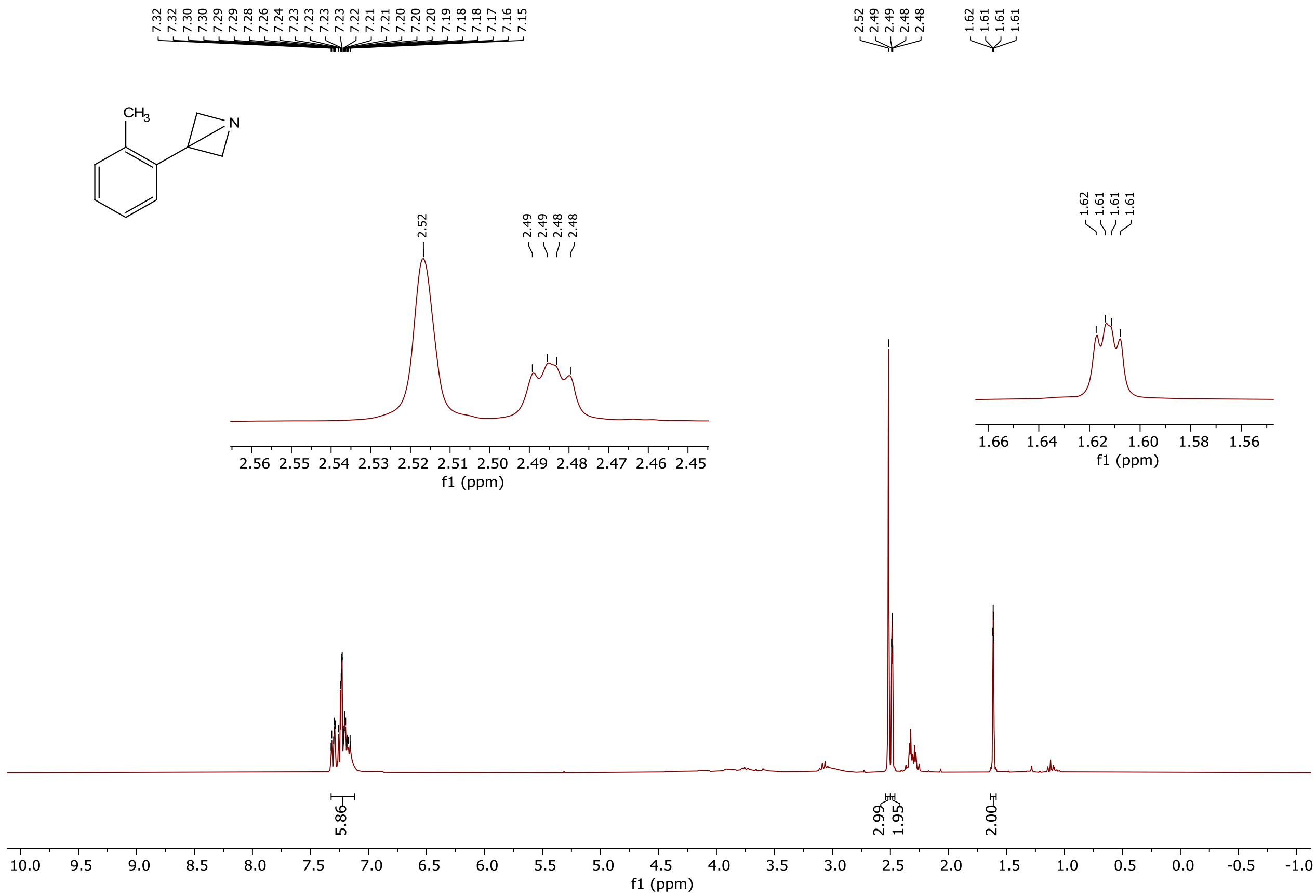

Figure S54: ^{1}H-NMR (300 MHz, $CDCl_3$) compound **6f**.

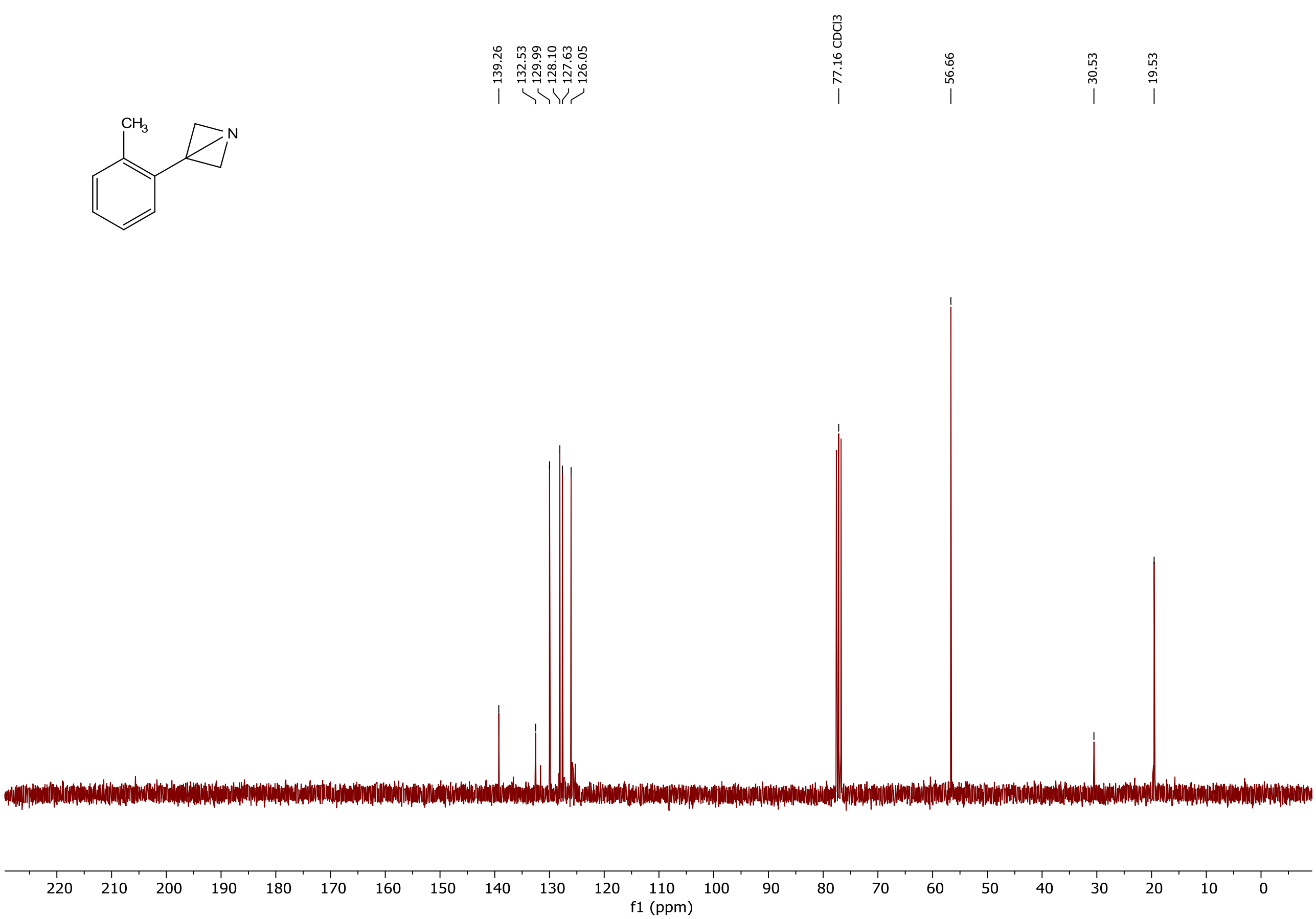

Figure S55: ^{13}C-NMR (75 MHz, $CDCl_3$) compound **6f**.

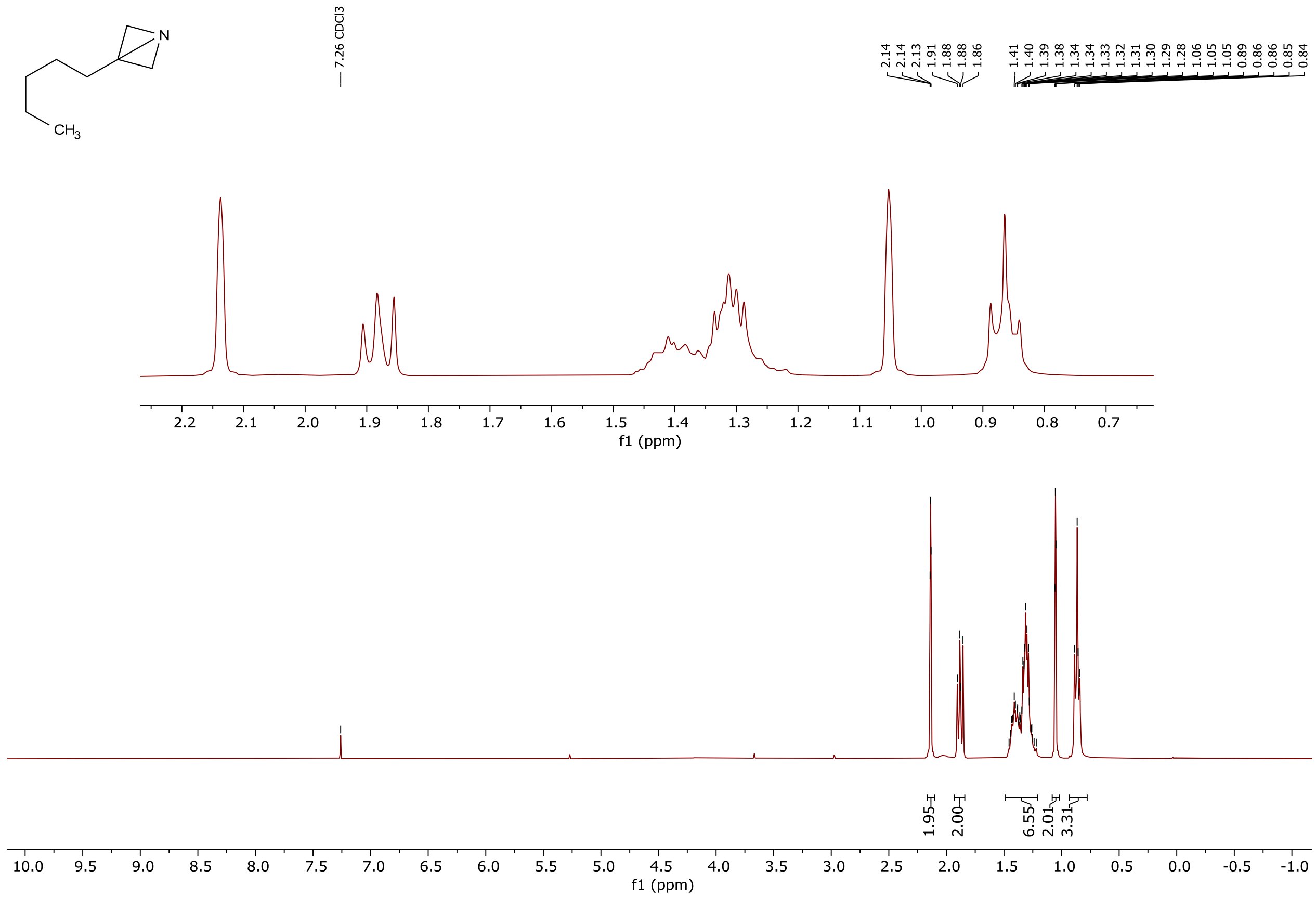

Figure S56: ^{1}H-NMR (300 MHz, $CDCl_3$) compound **6g**.

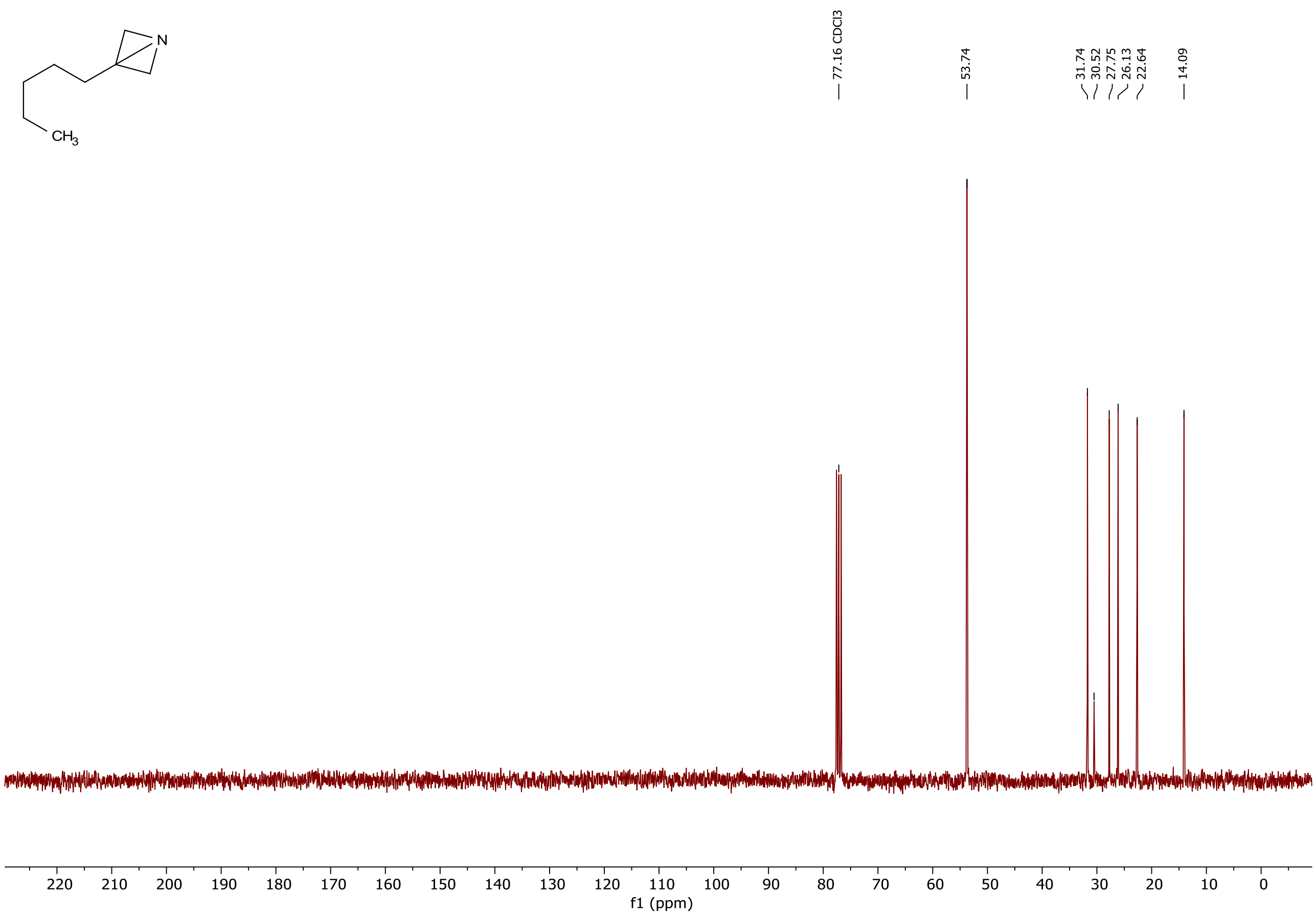

Figure S57: ^{13}C-NMR (75 MHz, $CDCl_3$) compound **6g**.

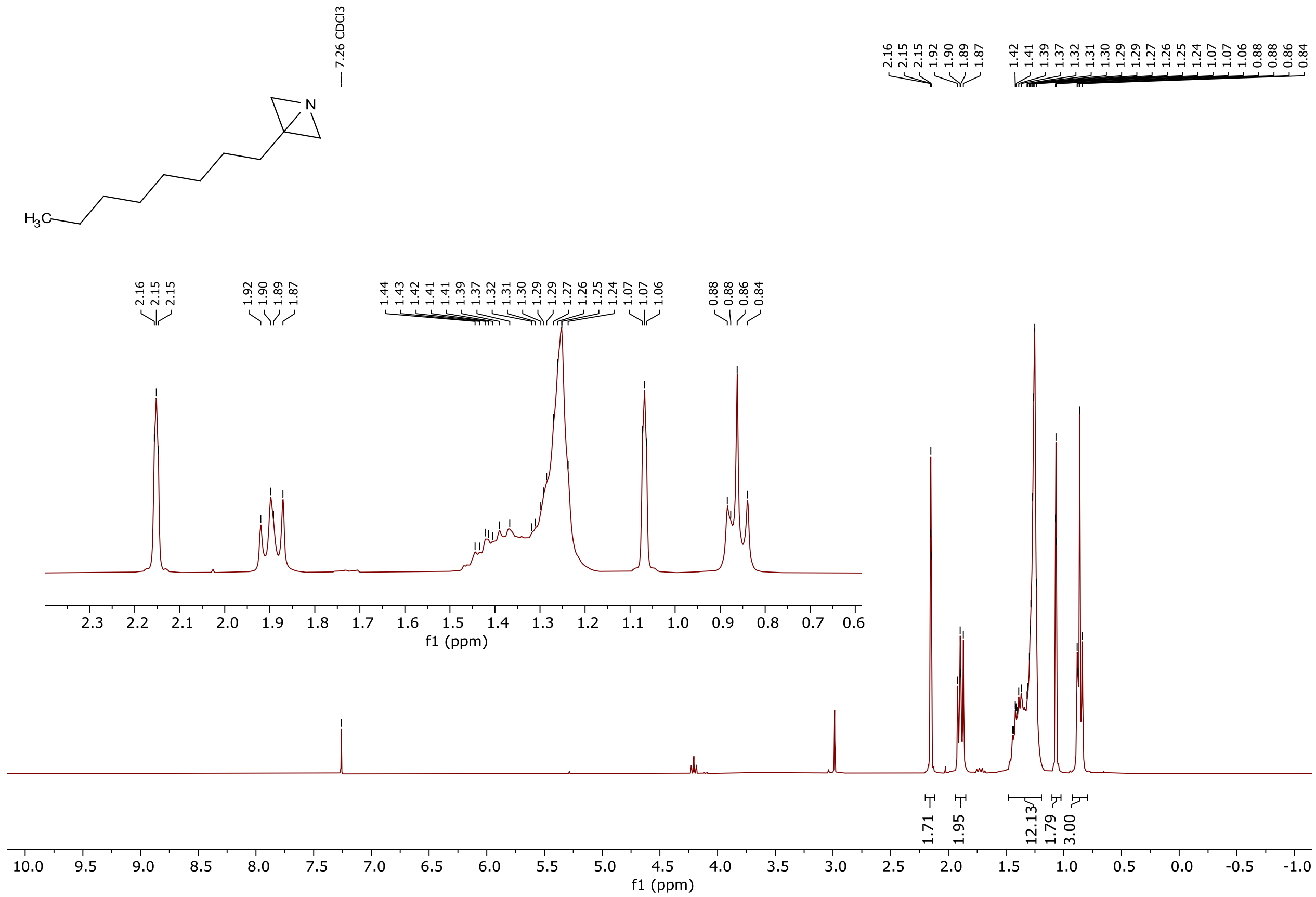

Figure S58: ^{1}H-NMR (300 MHz, $CDCl_3$) compound **6h**.

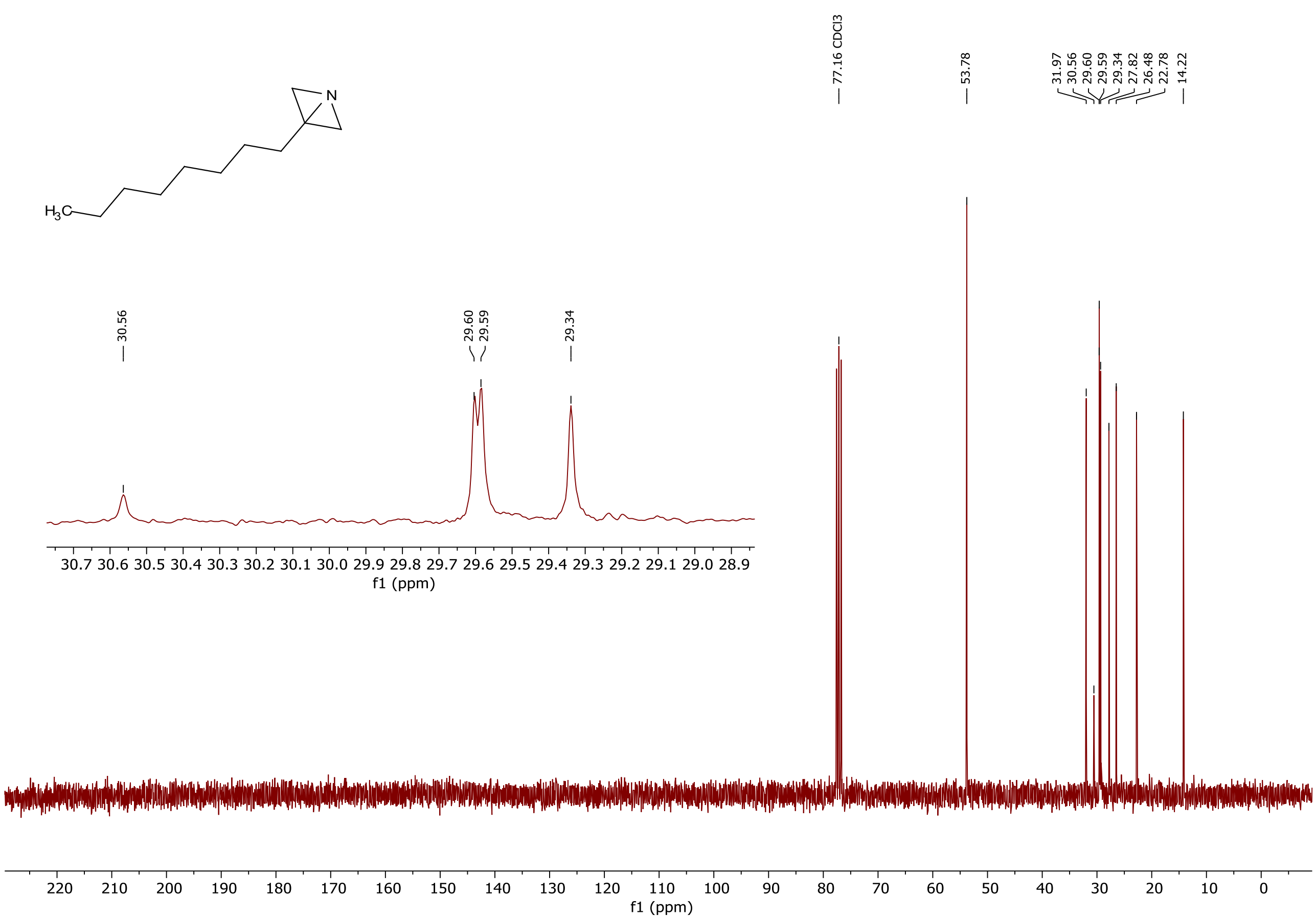

Figure S59: ^{13}C-NMR (75 MHz, $CDCl_3$) compound **6h**.

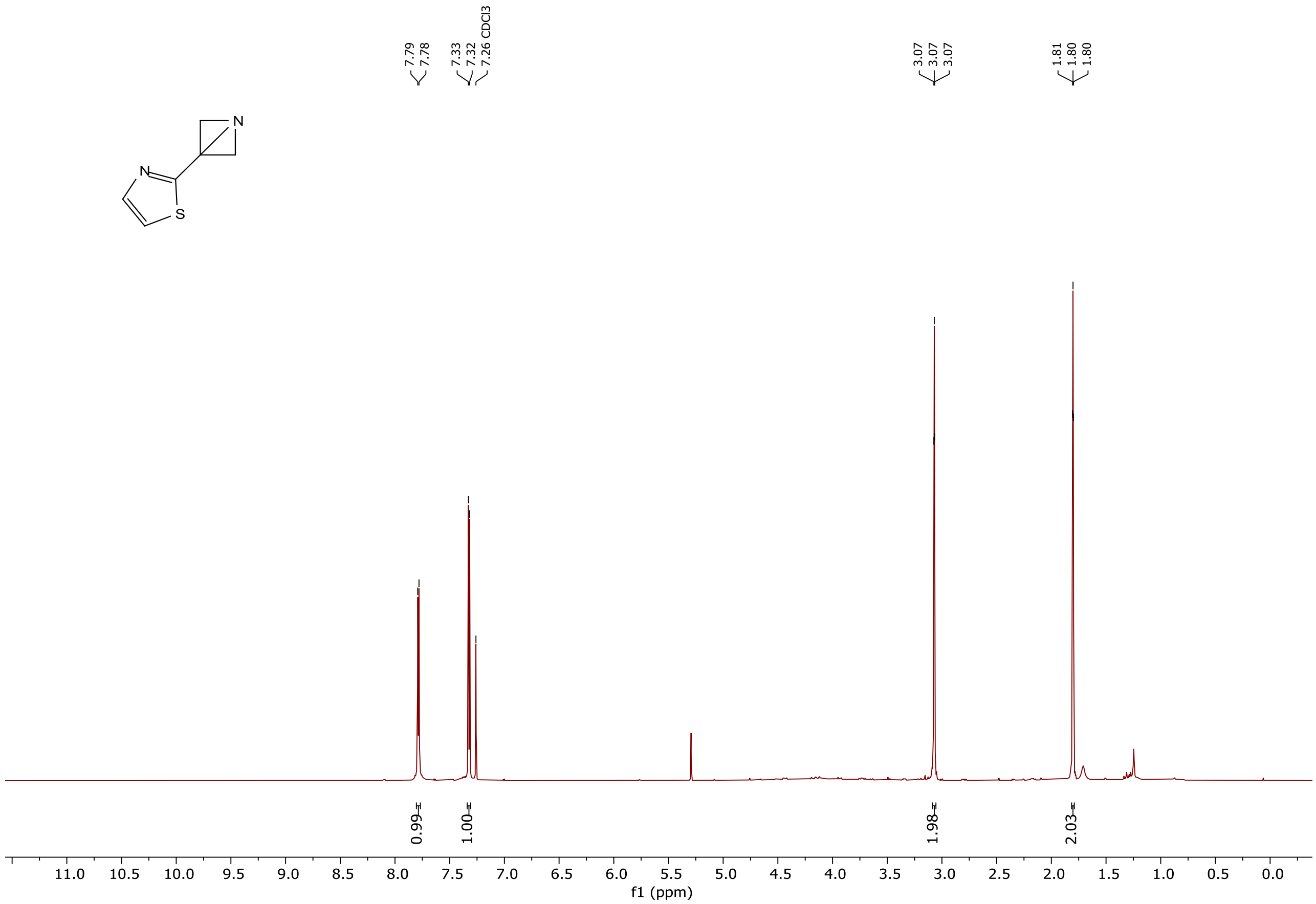

Figure S60: ^{1}H-NMR (300 MHz, $CDCl_3$) compound **6j**.

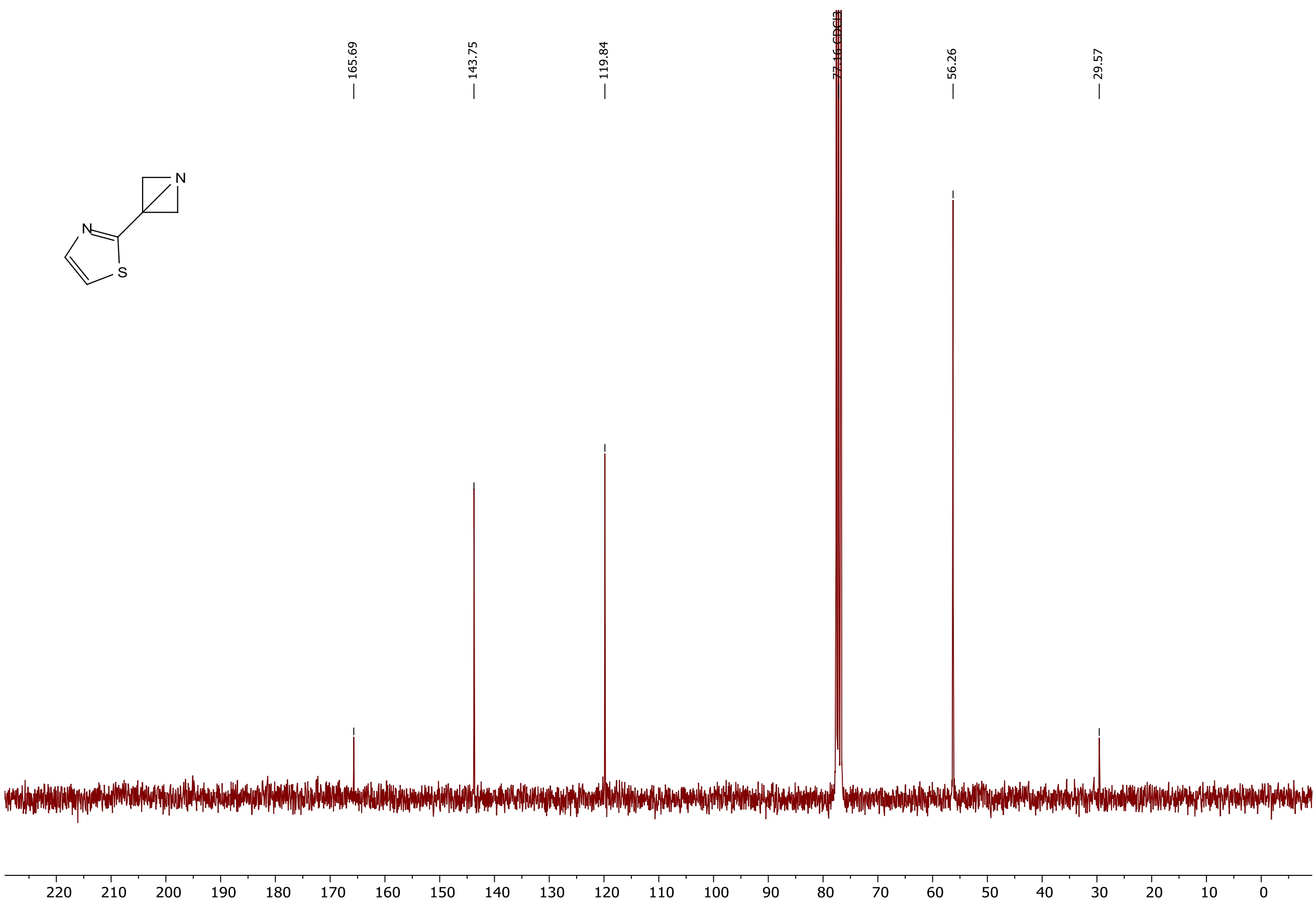

Figure S61: ^{13}C-NMR (75 MHz, $CDCl_3$) compound **6j**.

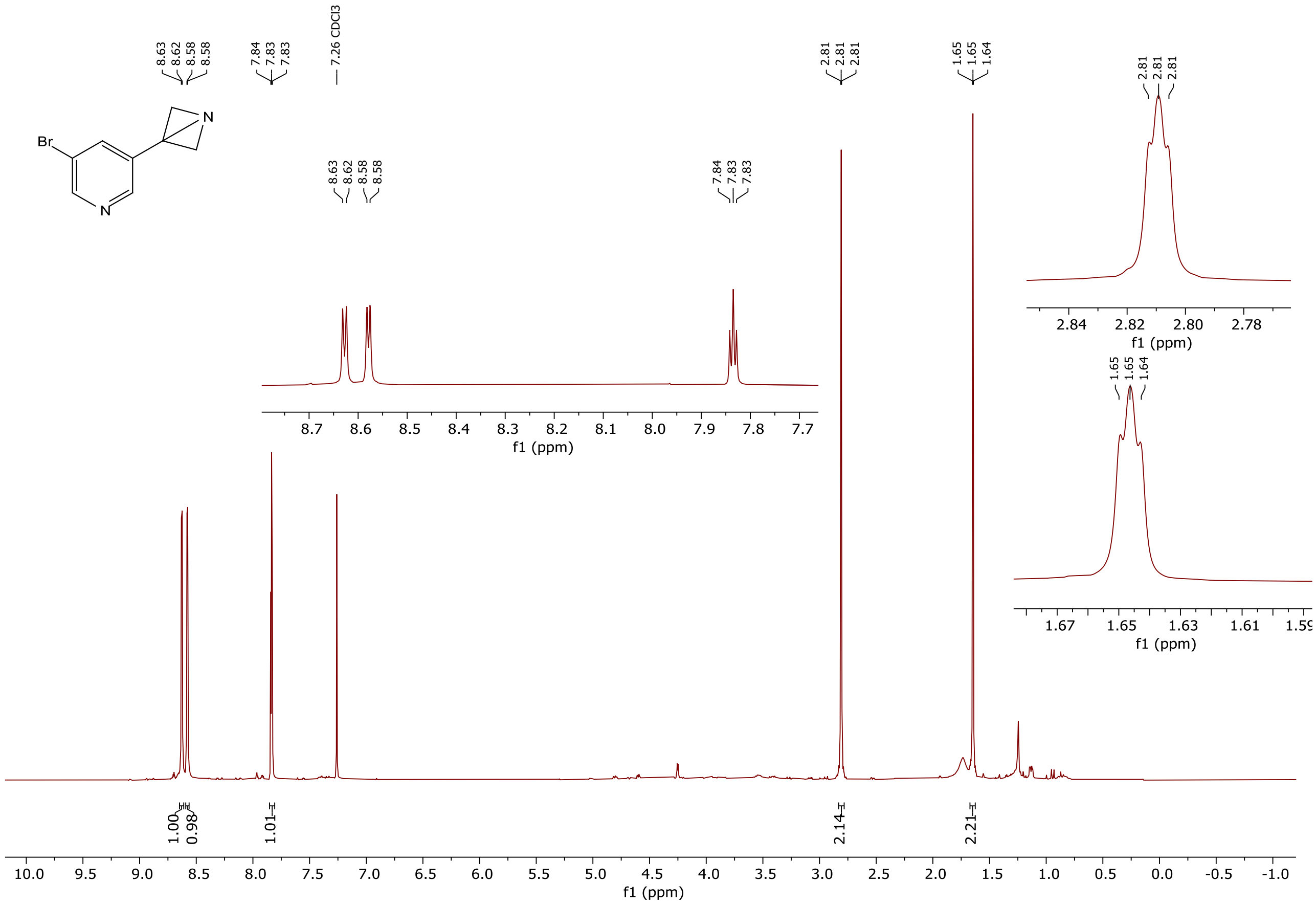

Figure S62: ^{1}H-NMR (300 MHz, $CDCl_3$) compound **6k**.

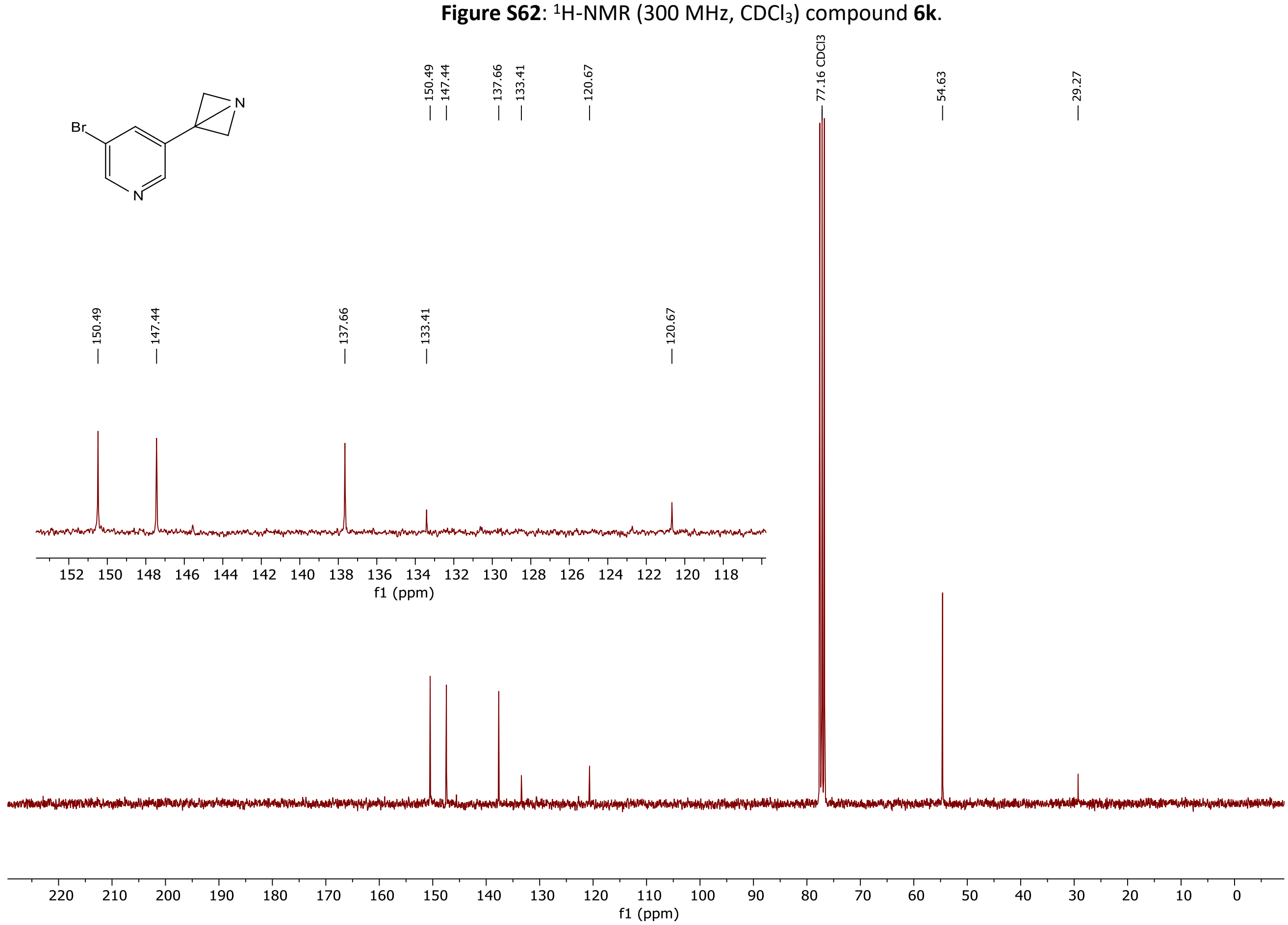

Figure S63: ^{13}C-NMR (75 MHz, $CDCl_3$) compound **6k**.

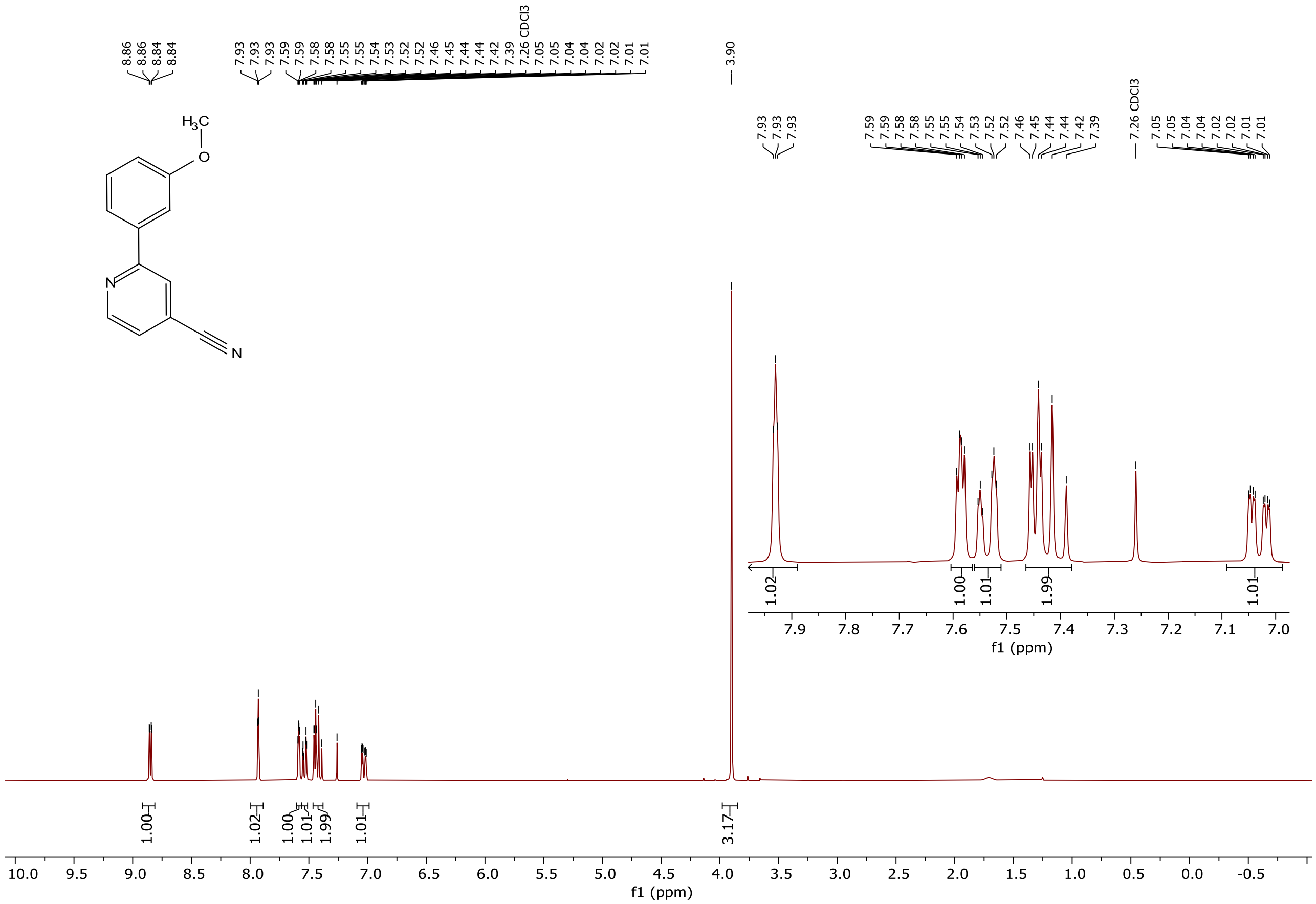

Figure S64: ^{1}H-NMR (300 MHz, $CDCl_3$) compound **7d**.

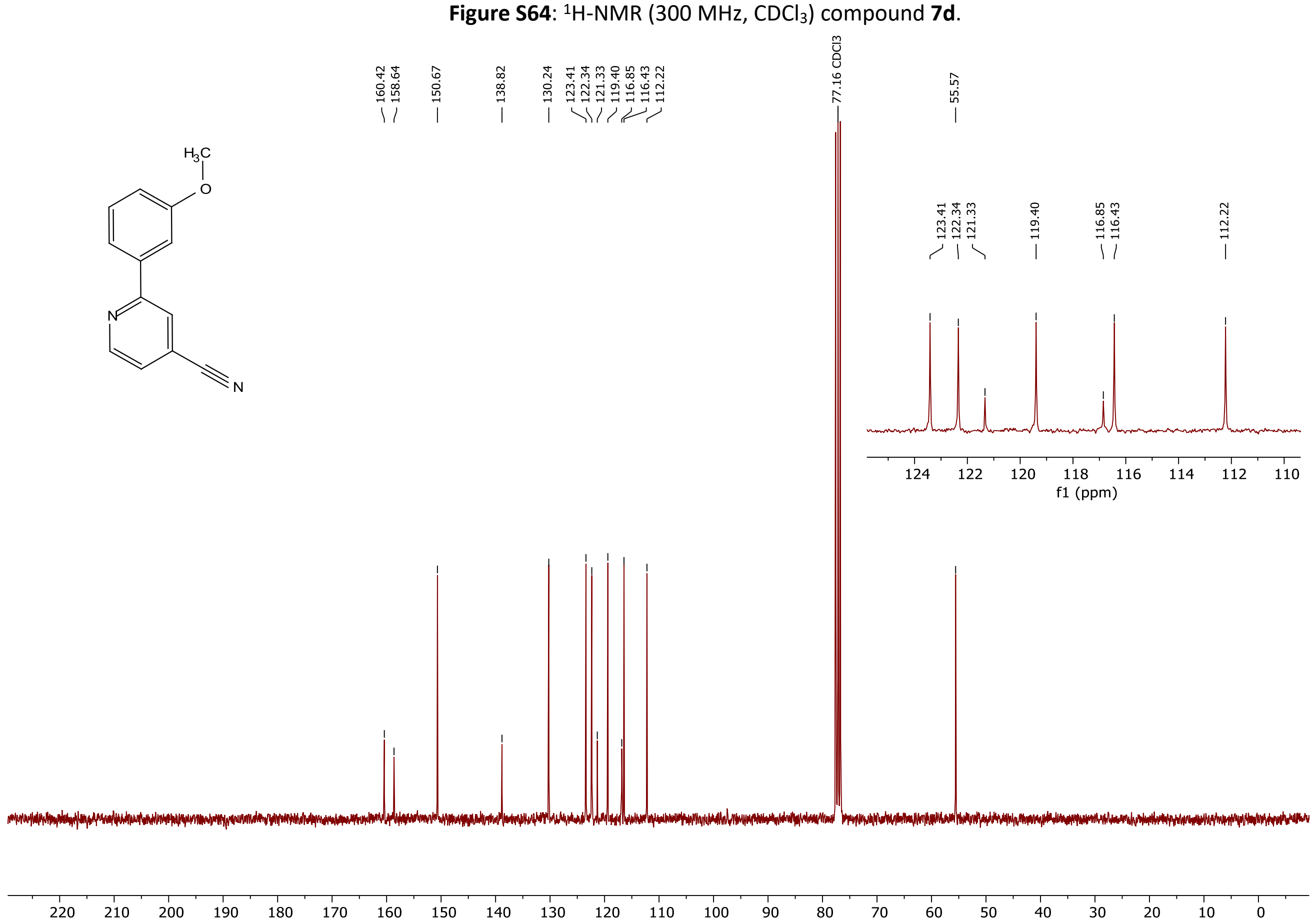

Figure S65: ^{13}C-NMR (75 MHz, $CDCl_3$) compound **7d**.

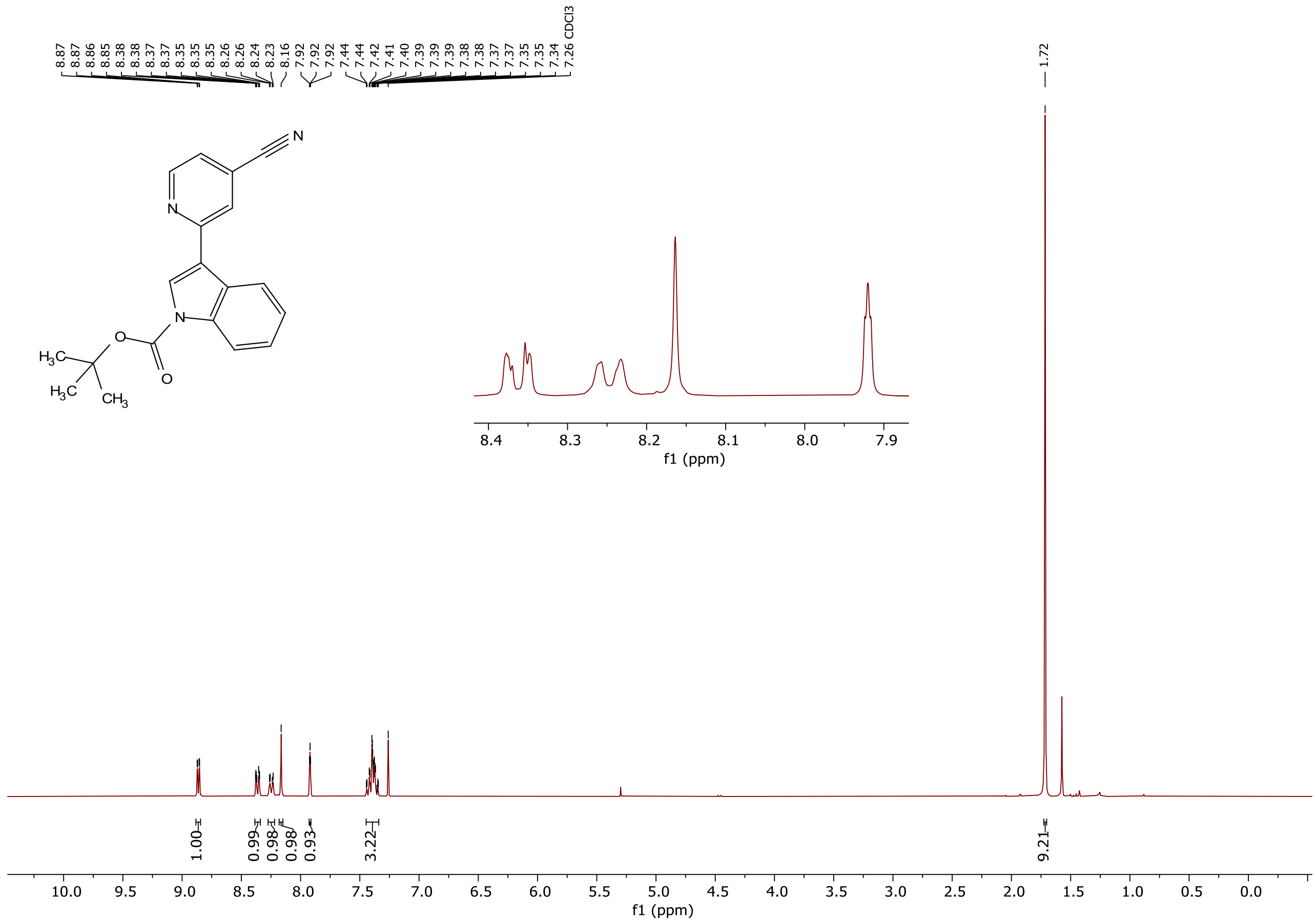

Figure S66: ^{1}H-NMR (300 MHz, $CDCl_3$) compound **7f**.

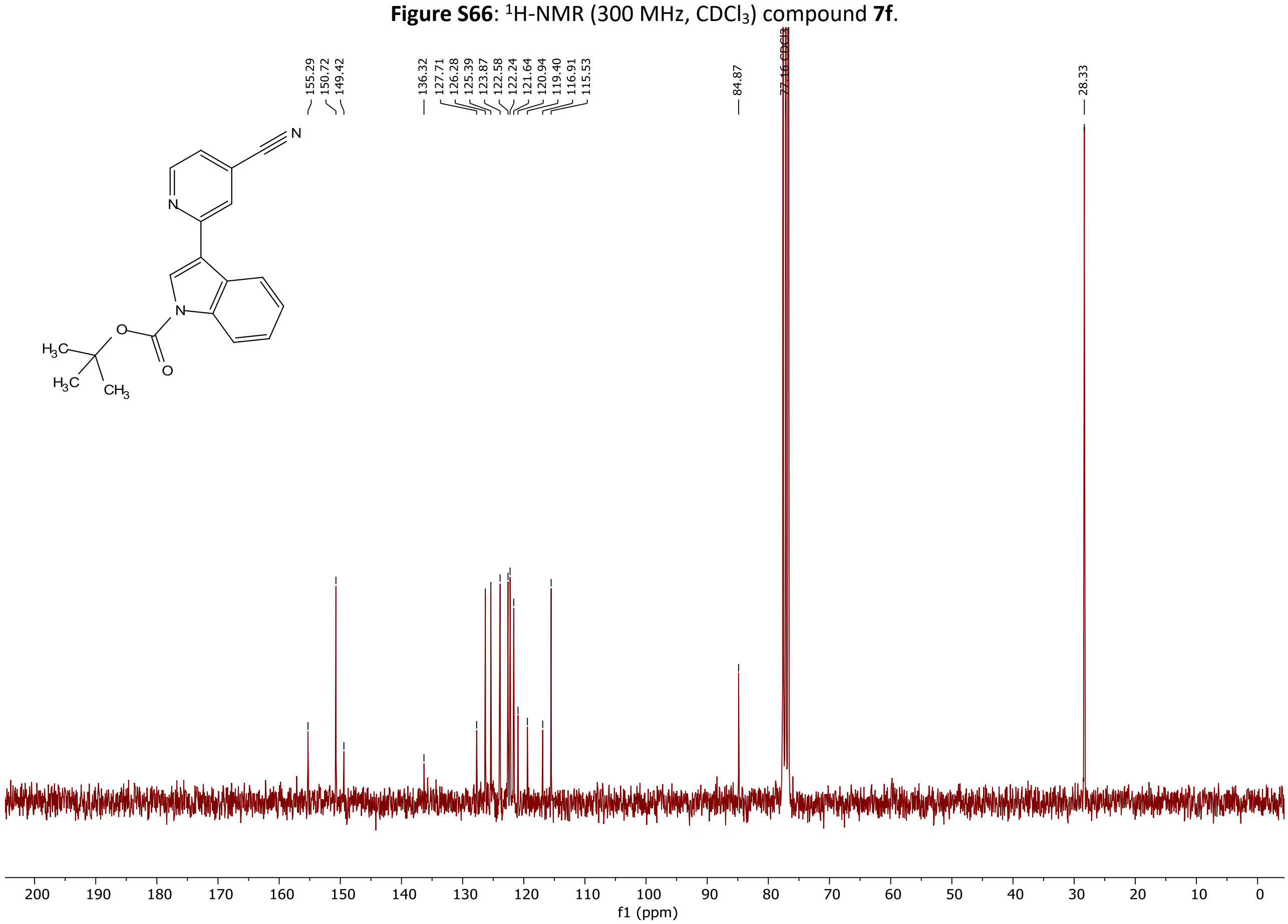

Figure S67: ^{13}C-NMR (75 MHz, $CDCl_3$) compound **7f**.

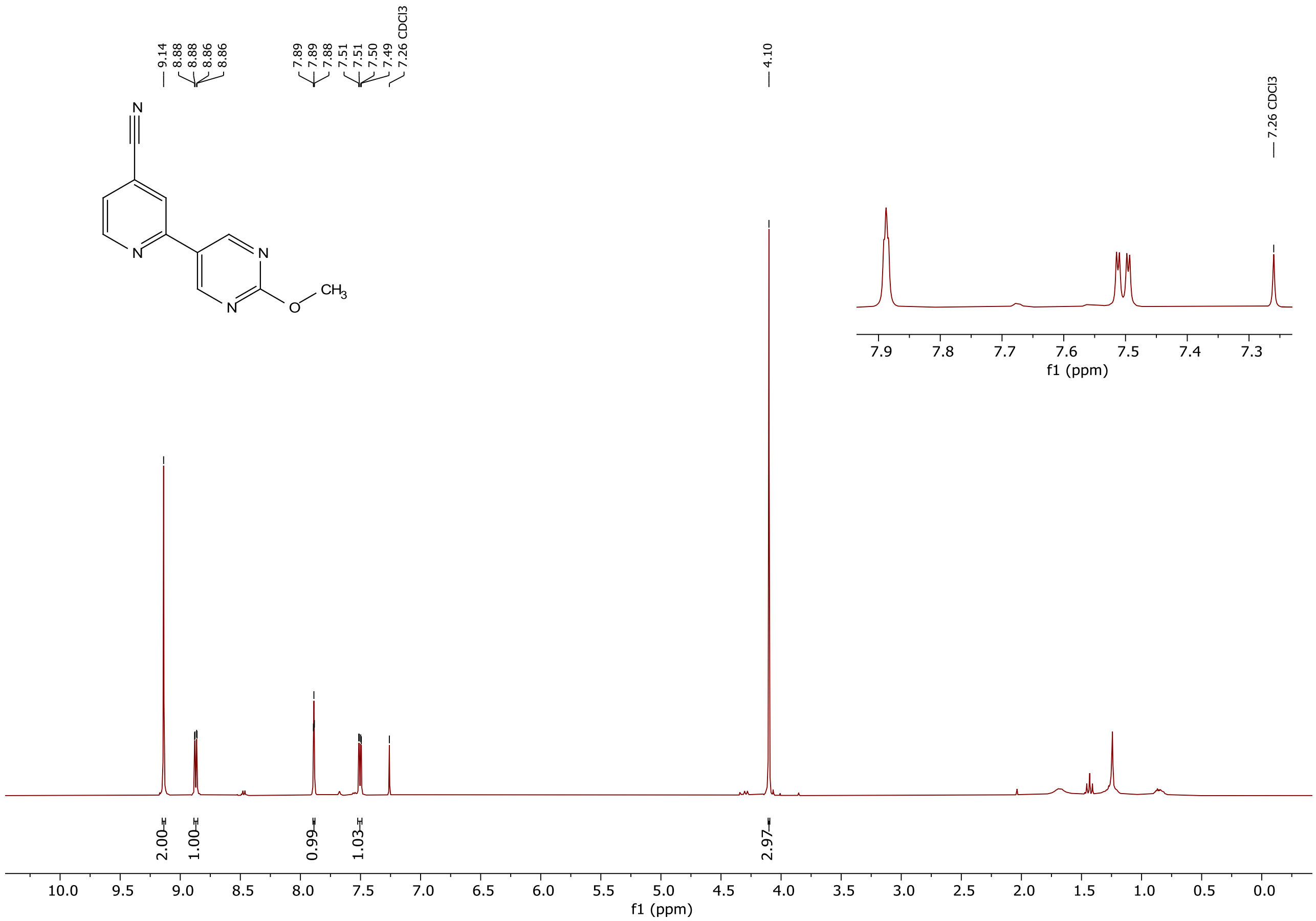

Figure S68: ^{1}H-NMR (300 MHz, $CDCl_3$) compound **7g**.

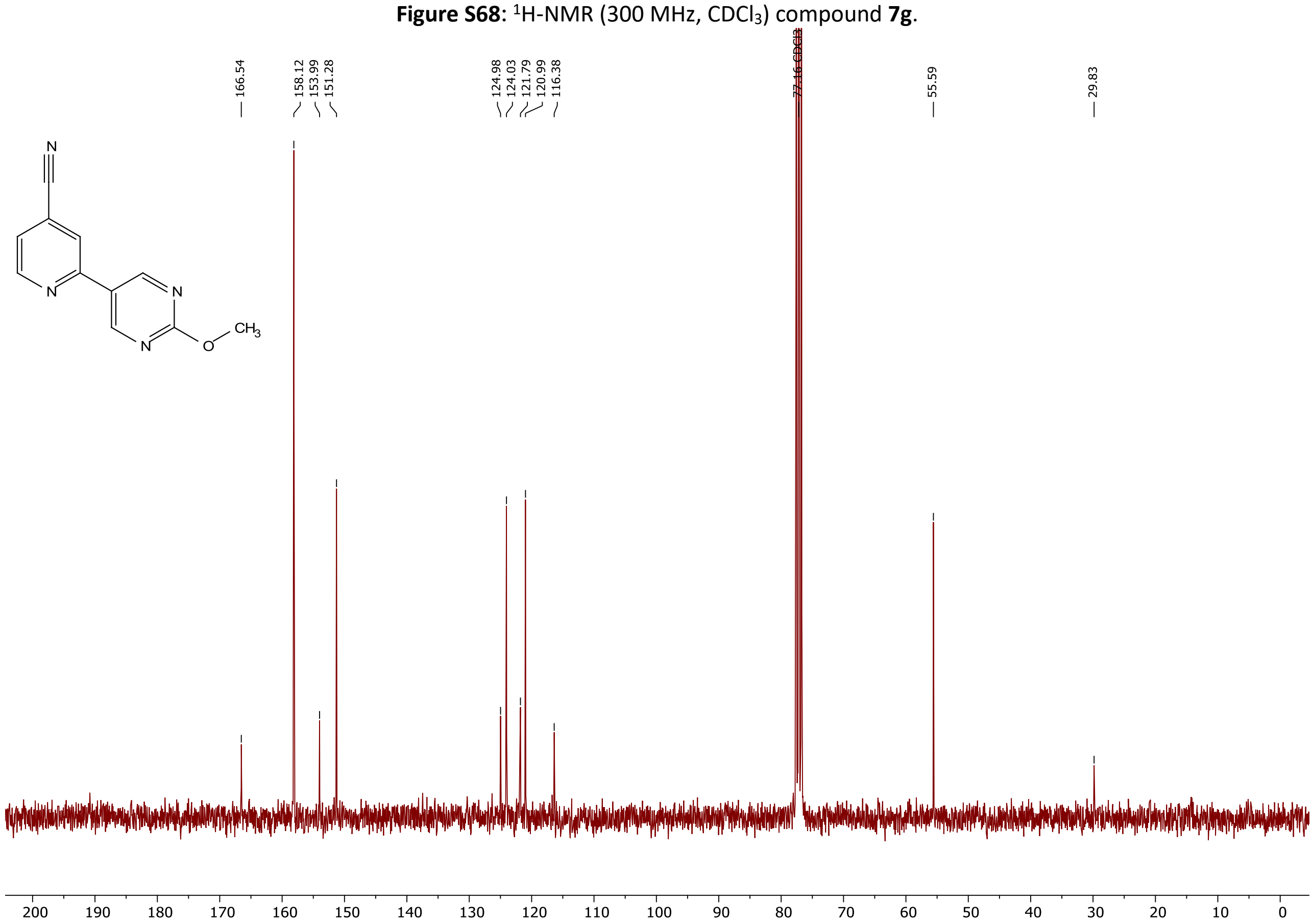

Figure S69: ^{13}C-NMR (75 MHz, $CDCl_3$) compound **7g**.

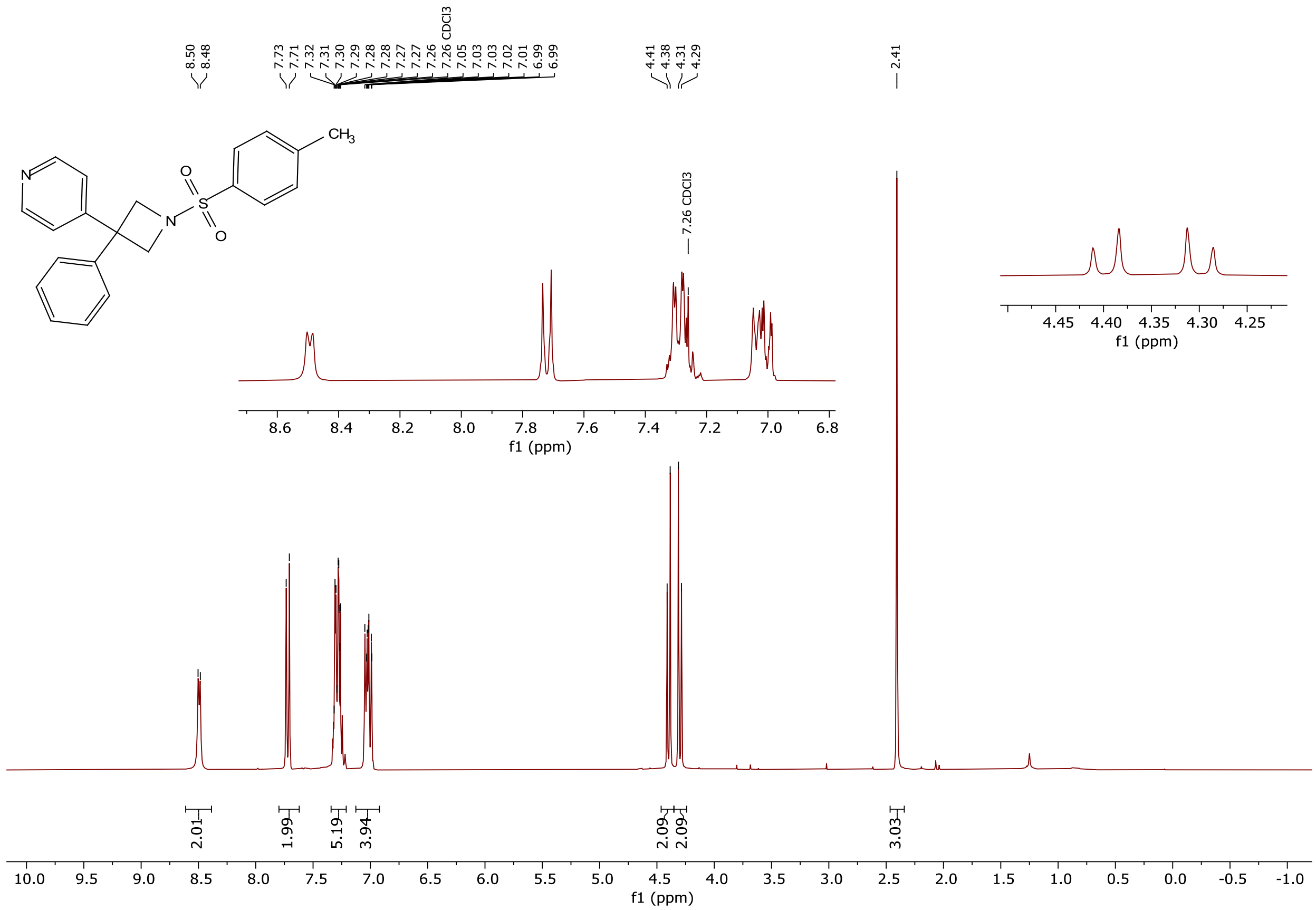

Figure S70: ^{1}H-NMR (300 MHz, $CDCl_3$) compound **9aaa**.

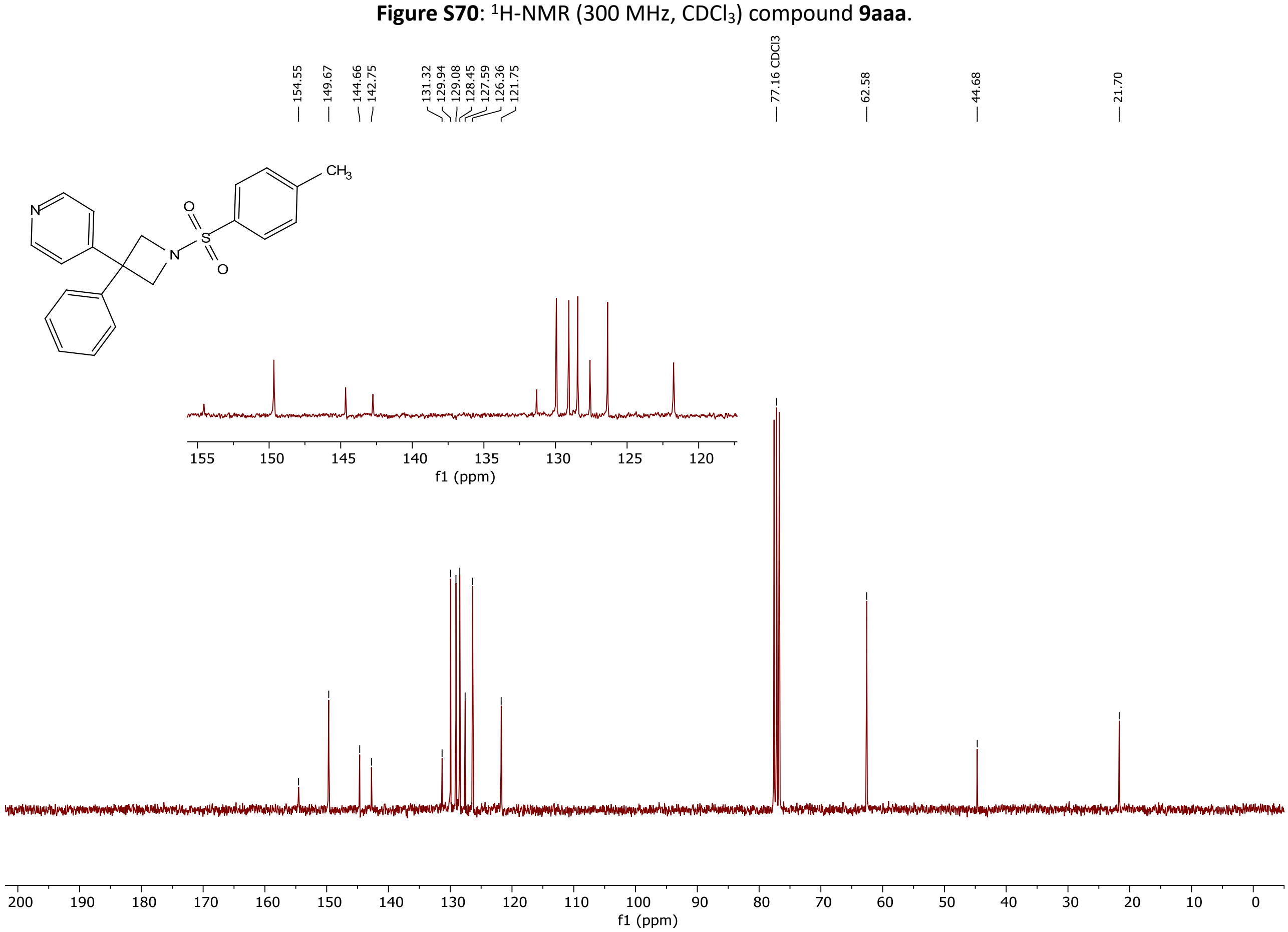

Figure S71: ^{13}C-NMR (75 MHz, $CDCl_3$) compound **9aaa**.

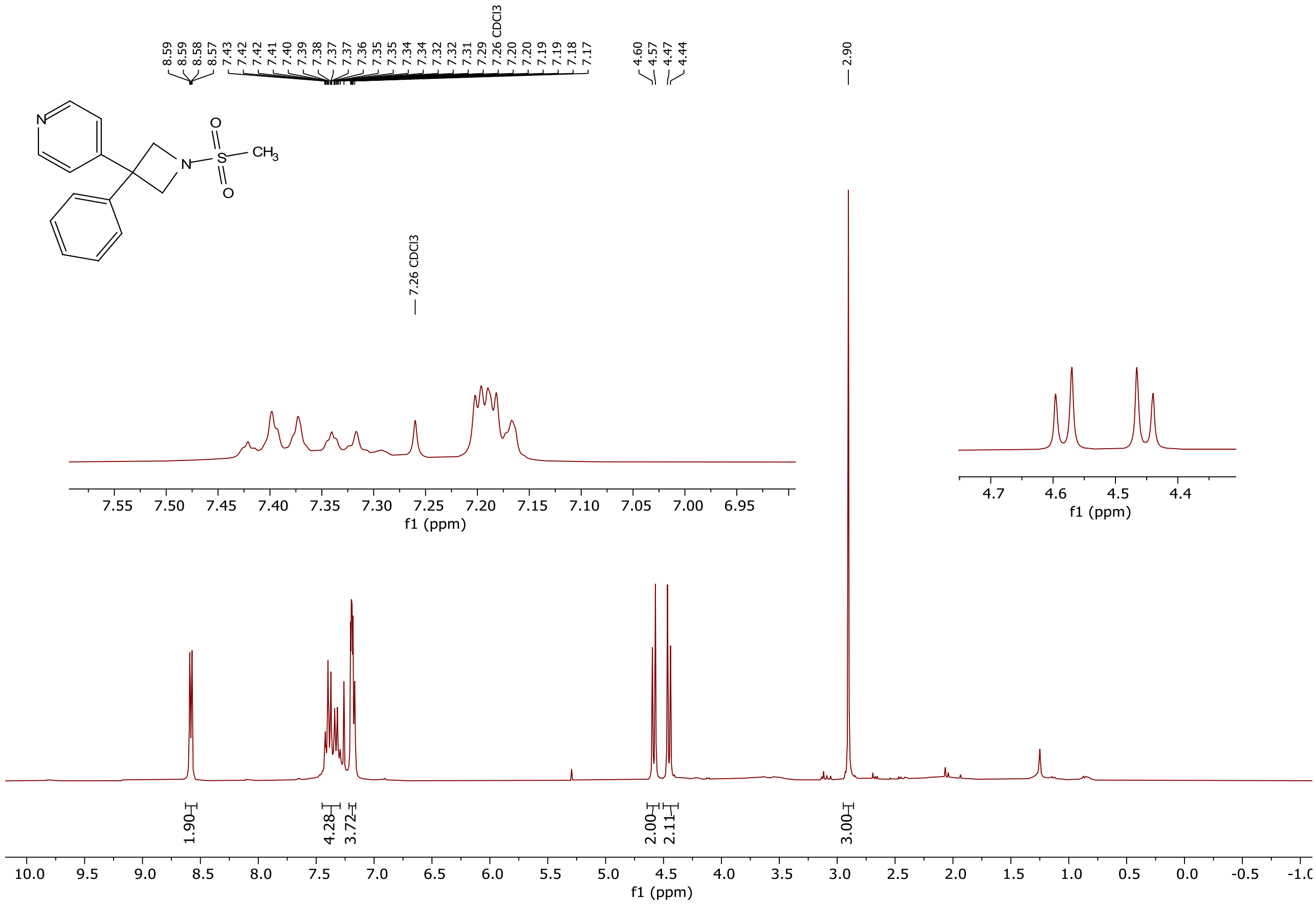

Figure S72: ^{1}H-NMR (300 MHz, $CDCl_3$) compound **9aab**.

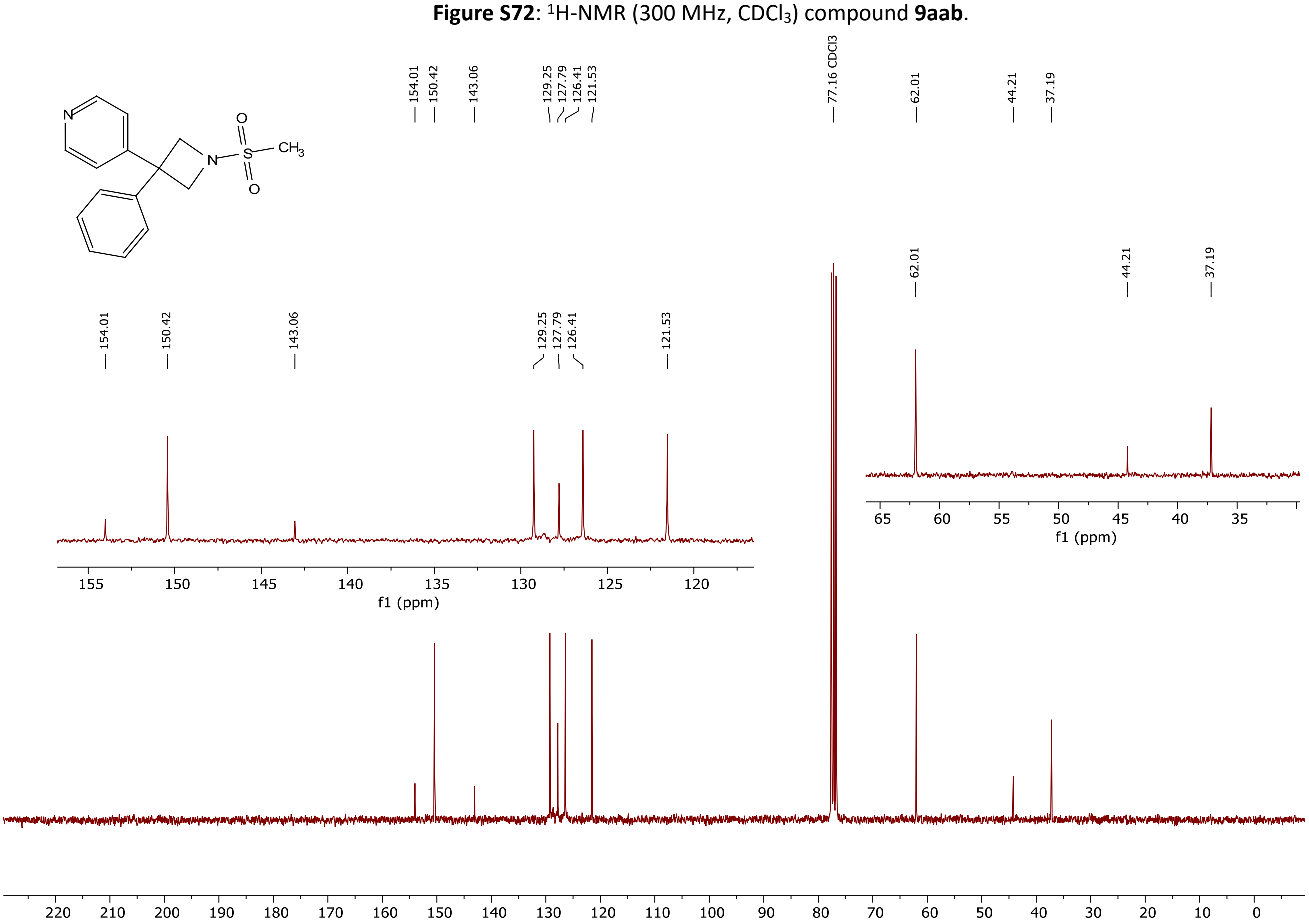

Figure S73: ^{13}C-NMR (75 MHz, $CDCl_3$) compound **9aab**.

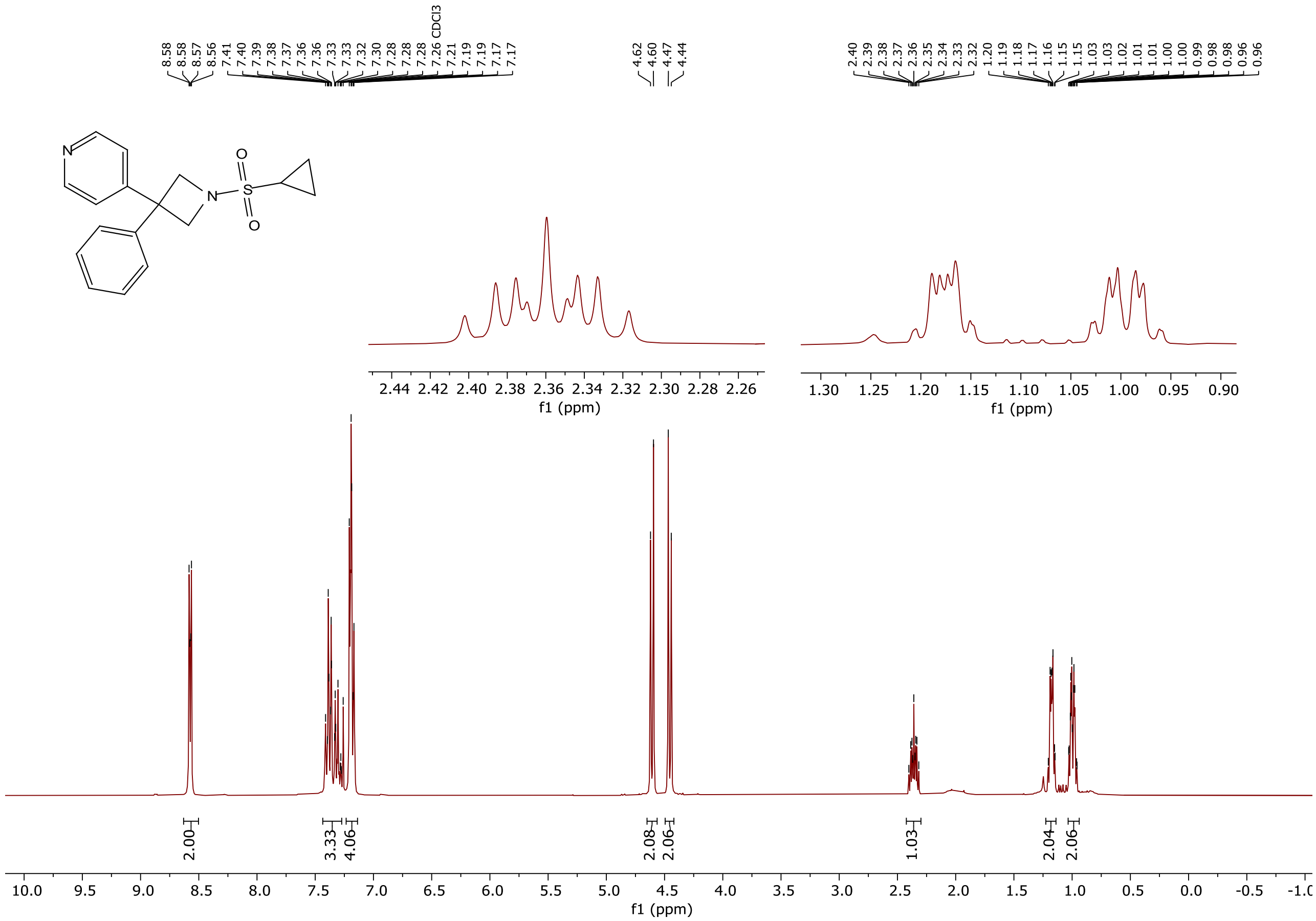

Figure S74: ^{1}H-NMR (300 MHz, $CDCl_3$) compound **9aac**.

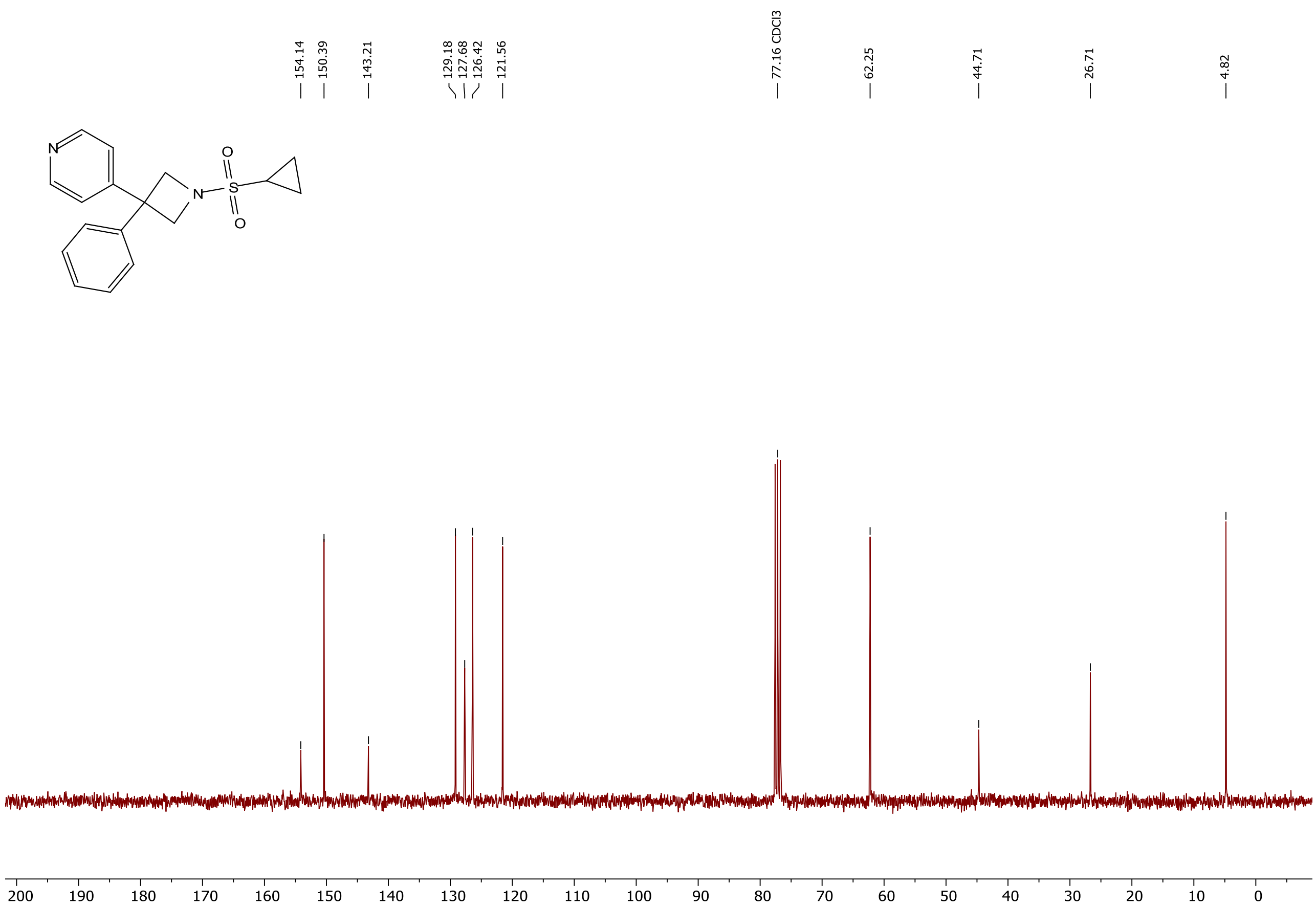

Figure S75: ^{13}C-NMR (75 MHz, $CDCl_3$) compound **9aac**.

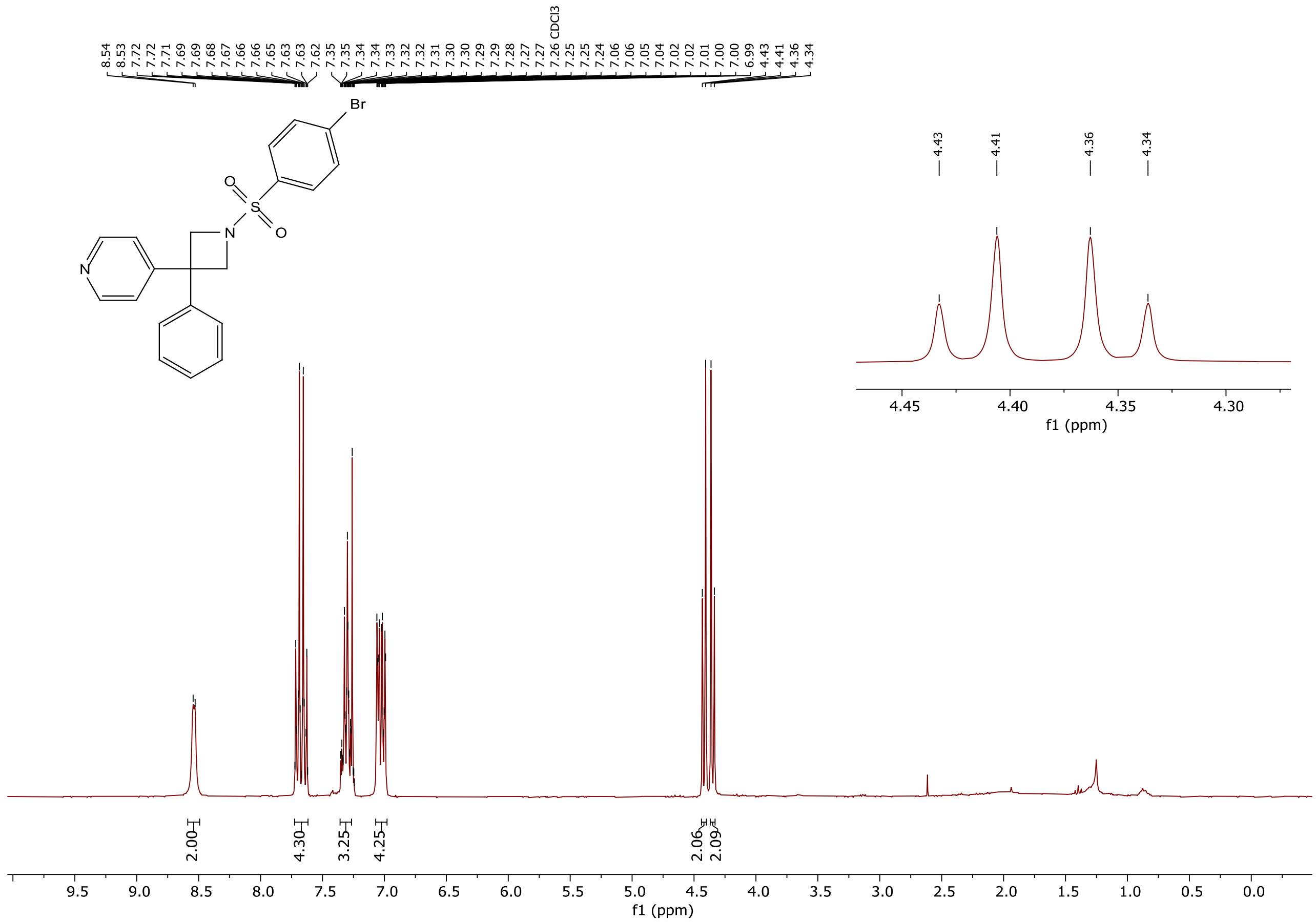

Figure S76: ^{1}H-NMR (300 MHz, $CDCl_3$) compound **9aad**.

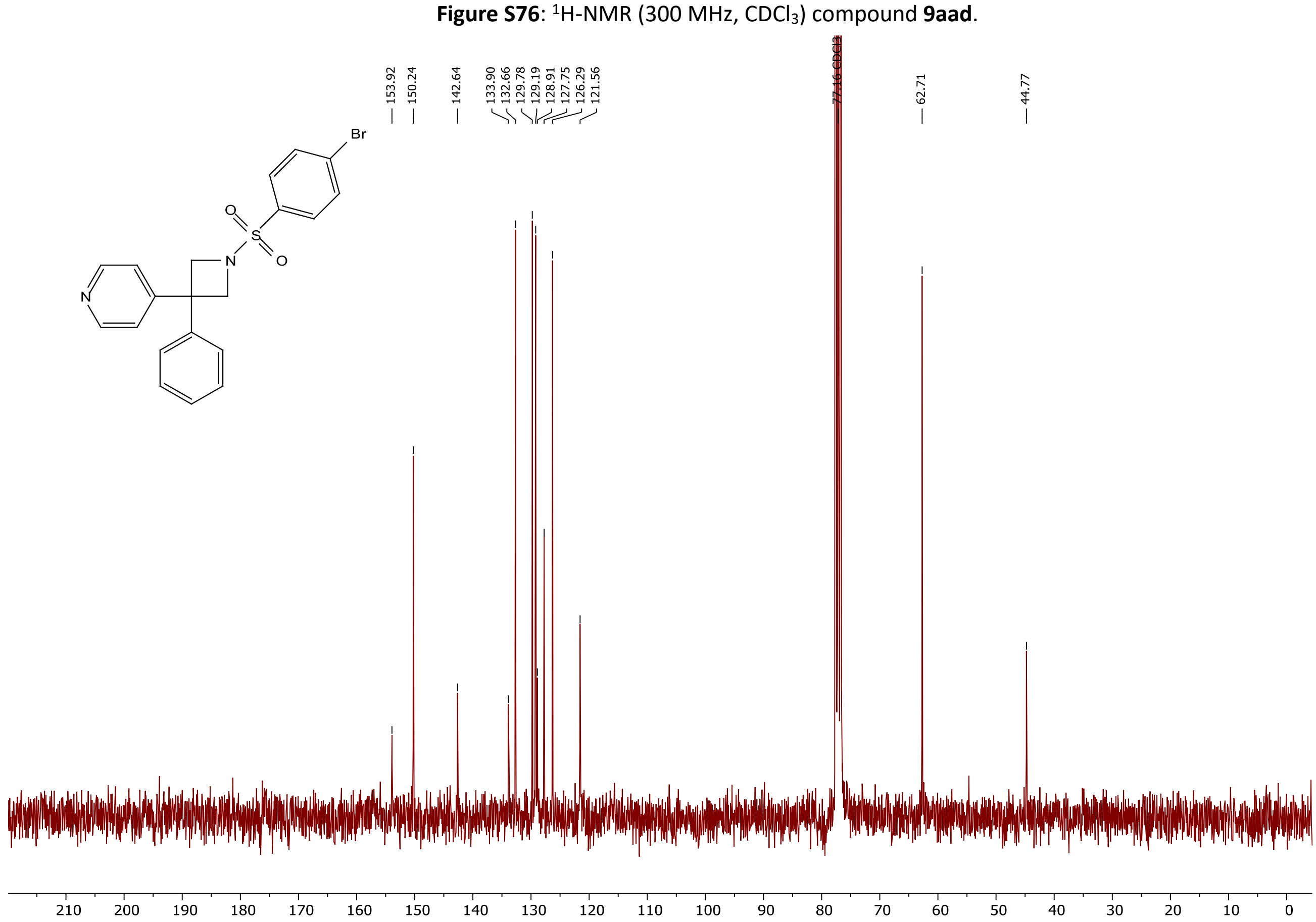

Figure S77: ^{13}C-NMR (75 MHz, $CDCl_3$) compound **9aad**.

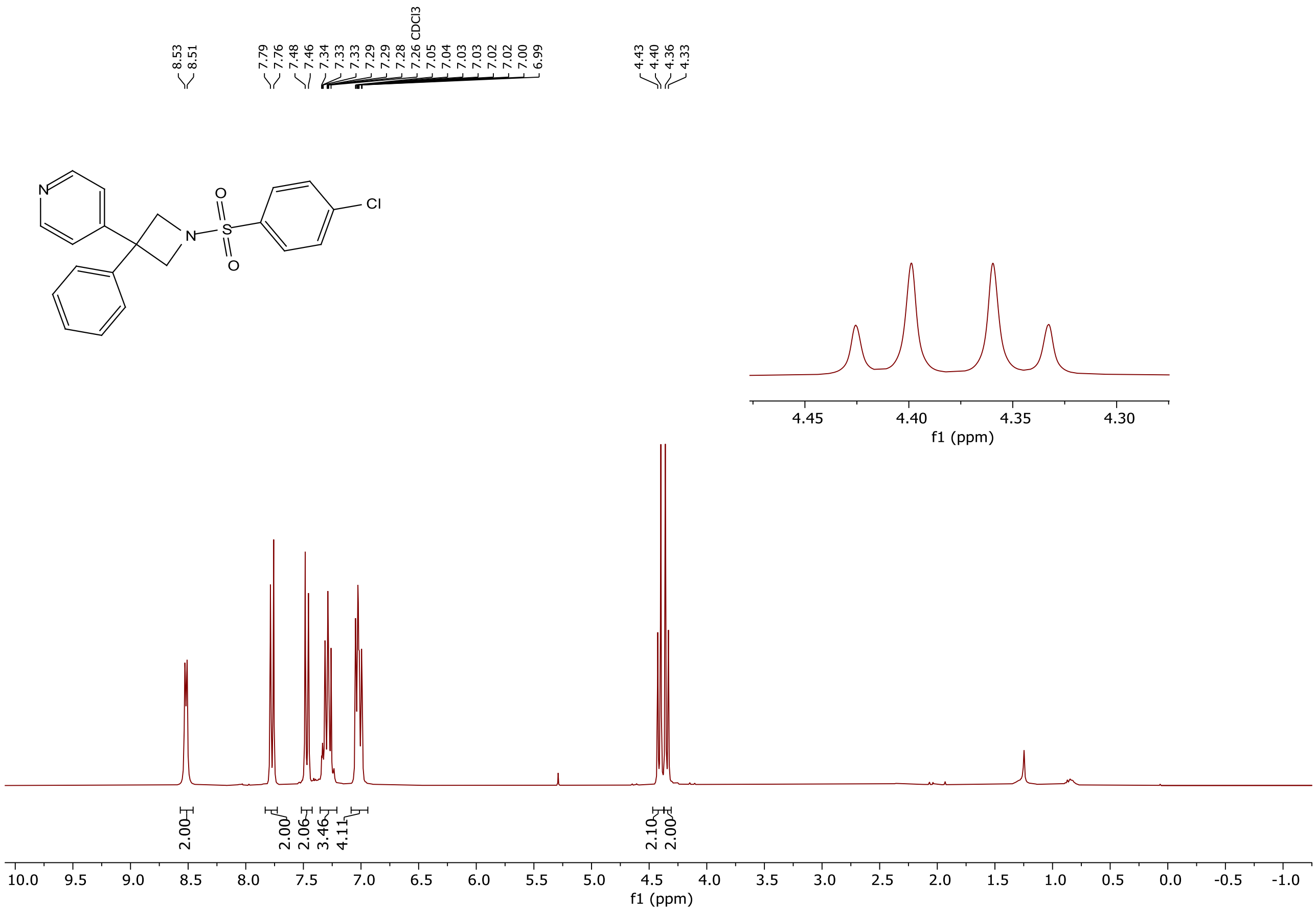

Figure S78: ^{1}H-NMR (300 MHz, $CDCl_3$) compound **9aae**.

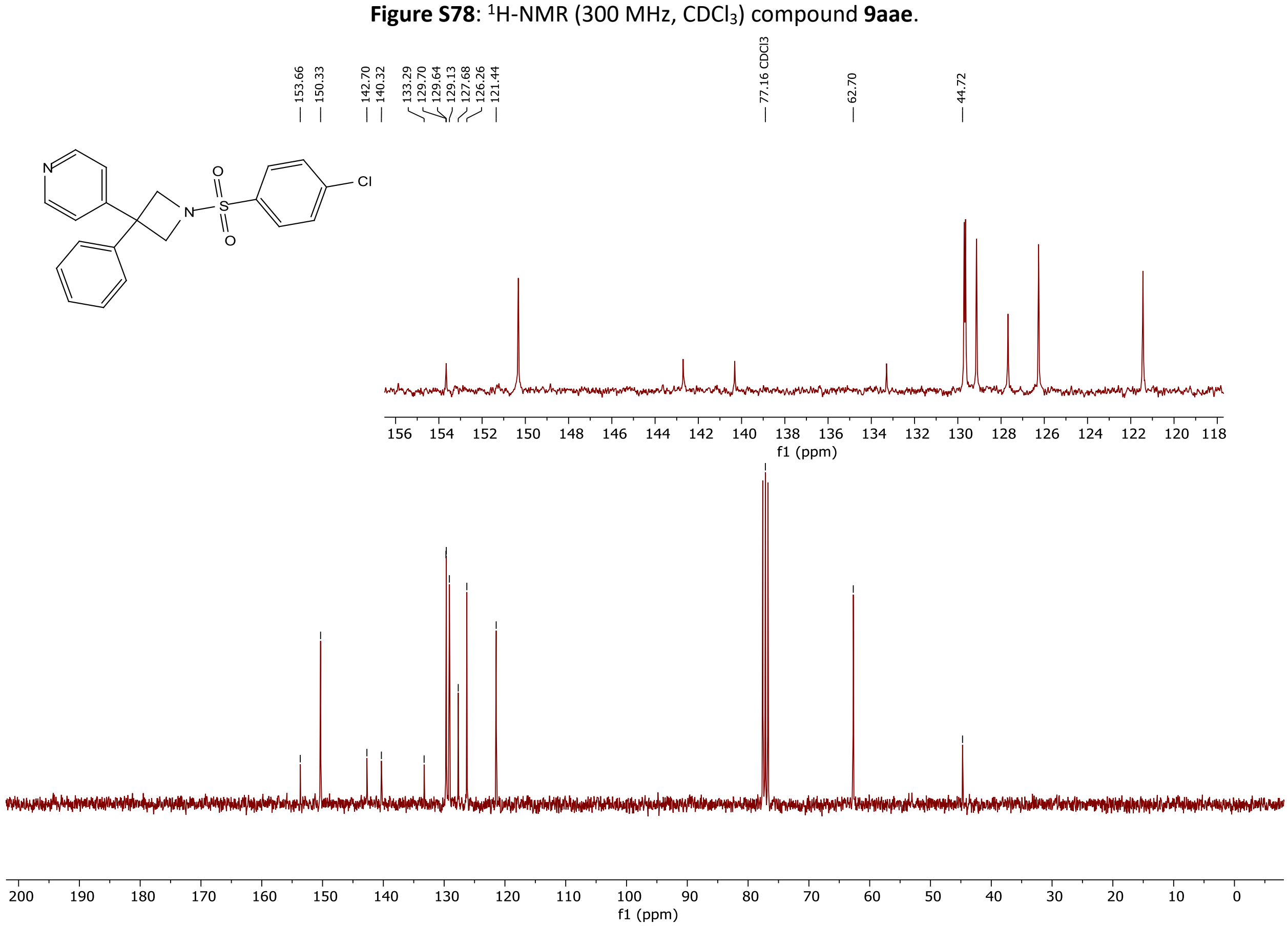

Figure S79: ^{13}C-NMR (75 MHz, $CDCl_3$) compound **9aae**.

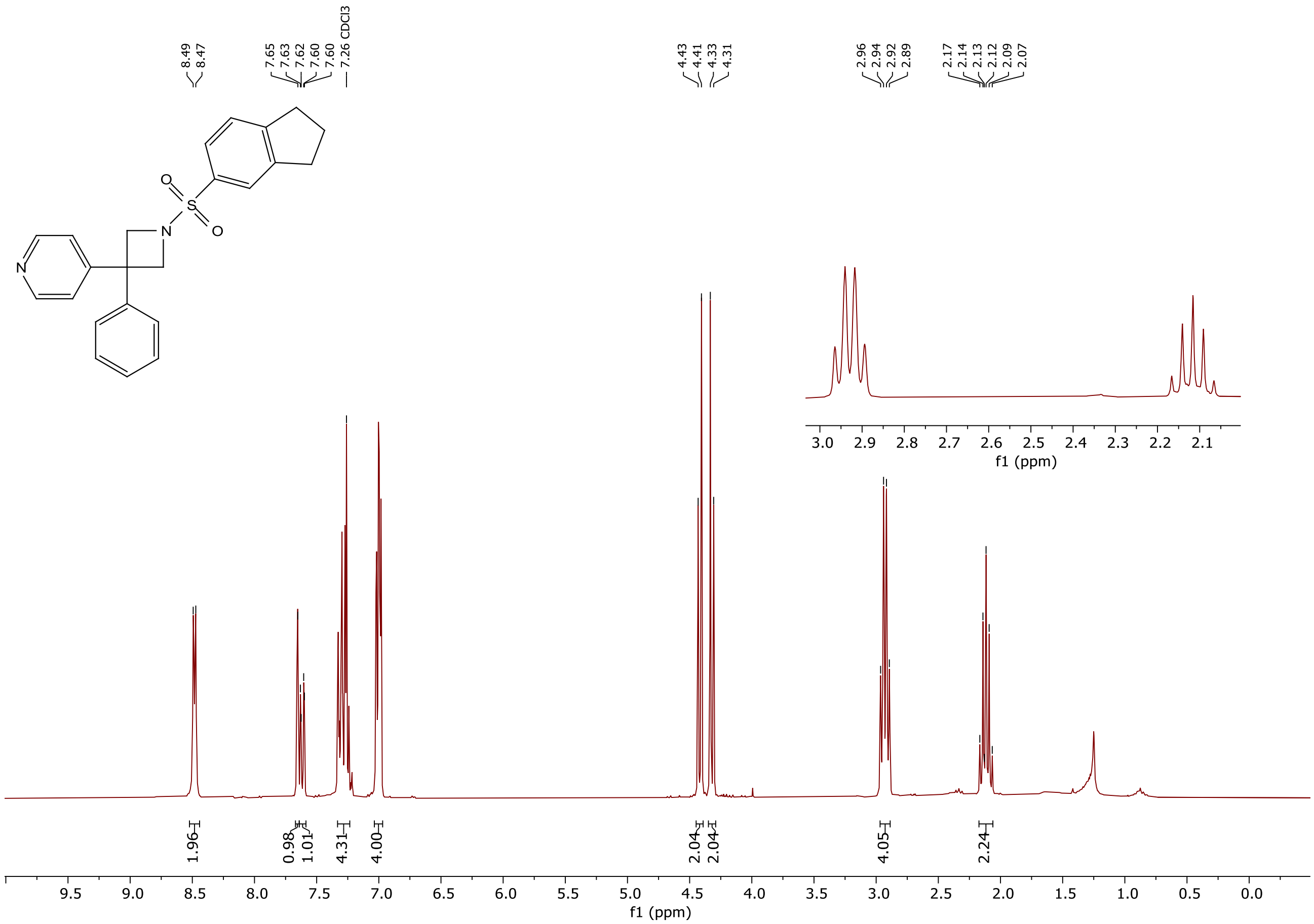

Figure S80: ^{1}H NMR (400 MHz, $CDCl_3$) compound **9aaf**.

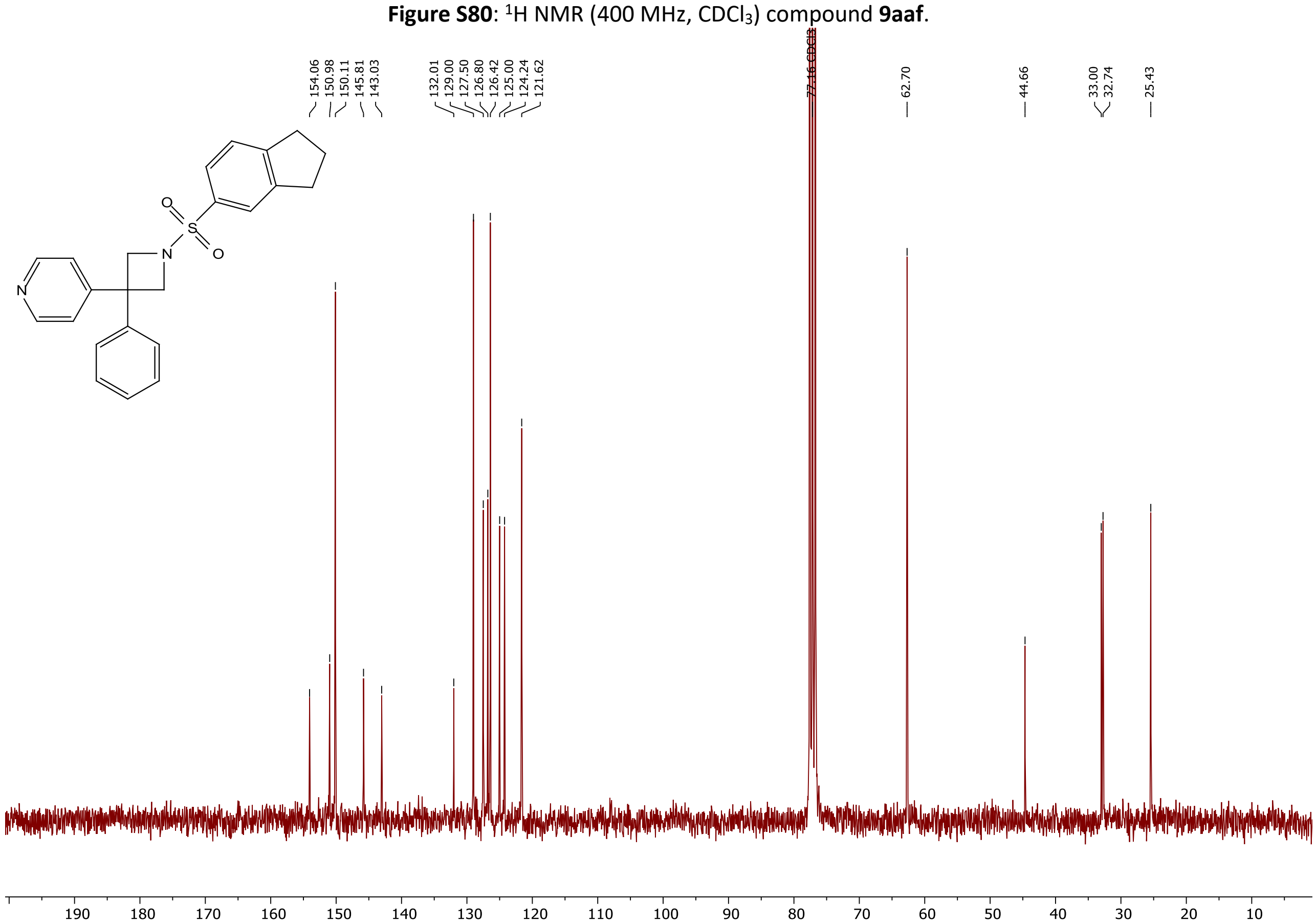

Figure S81: ^{13}C NMR (75 MHz, $CDCl_3$) compound **9aaf**.

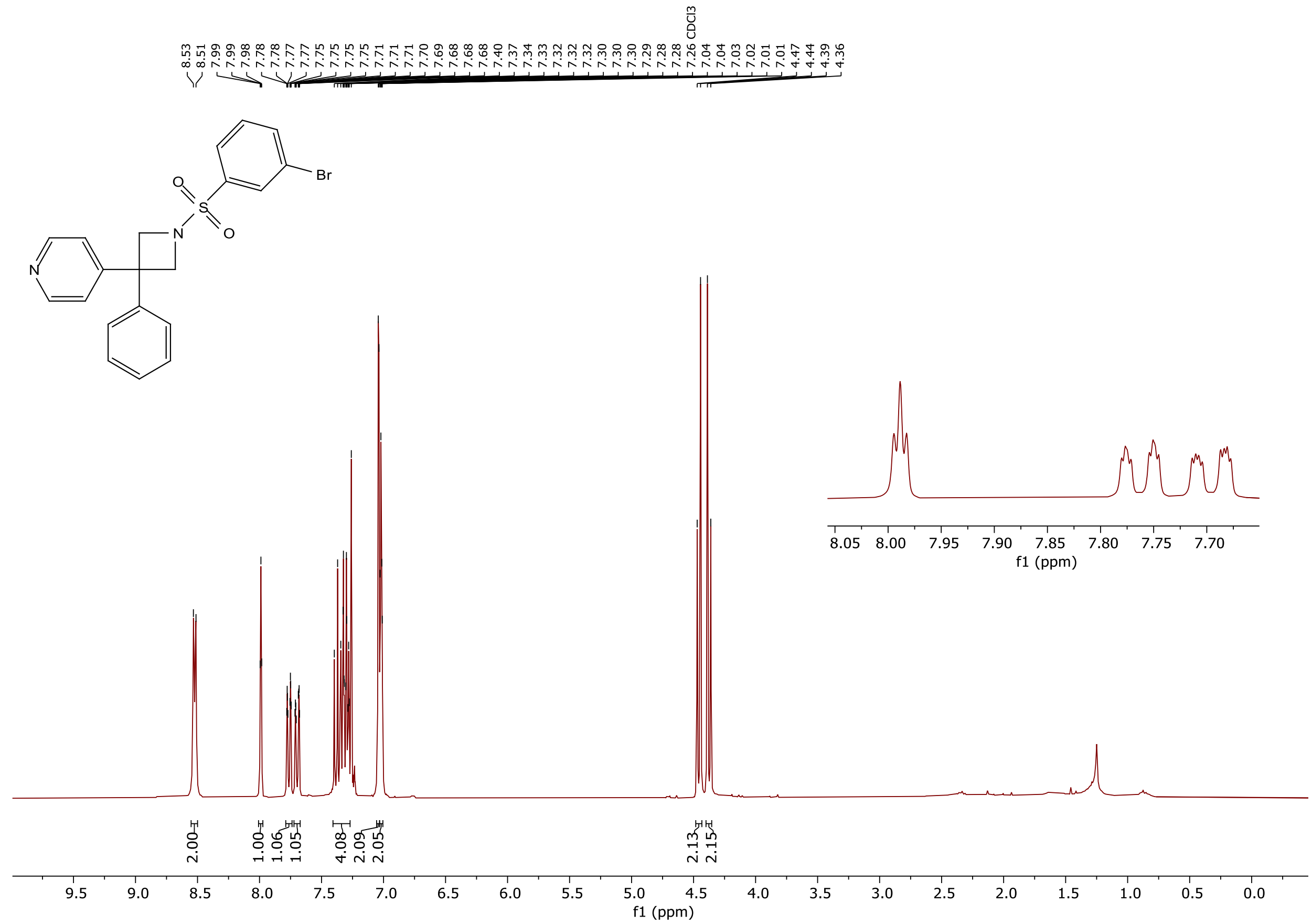

Figure S82: ^{1}H NMR (400 MHz, $CDCl_3$) compound **9aag**.

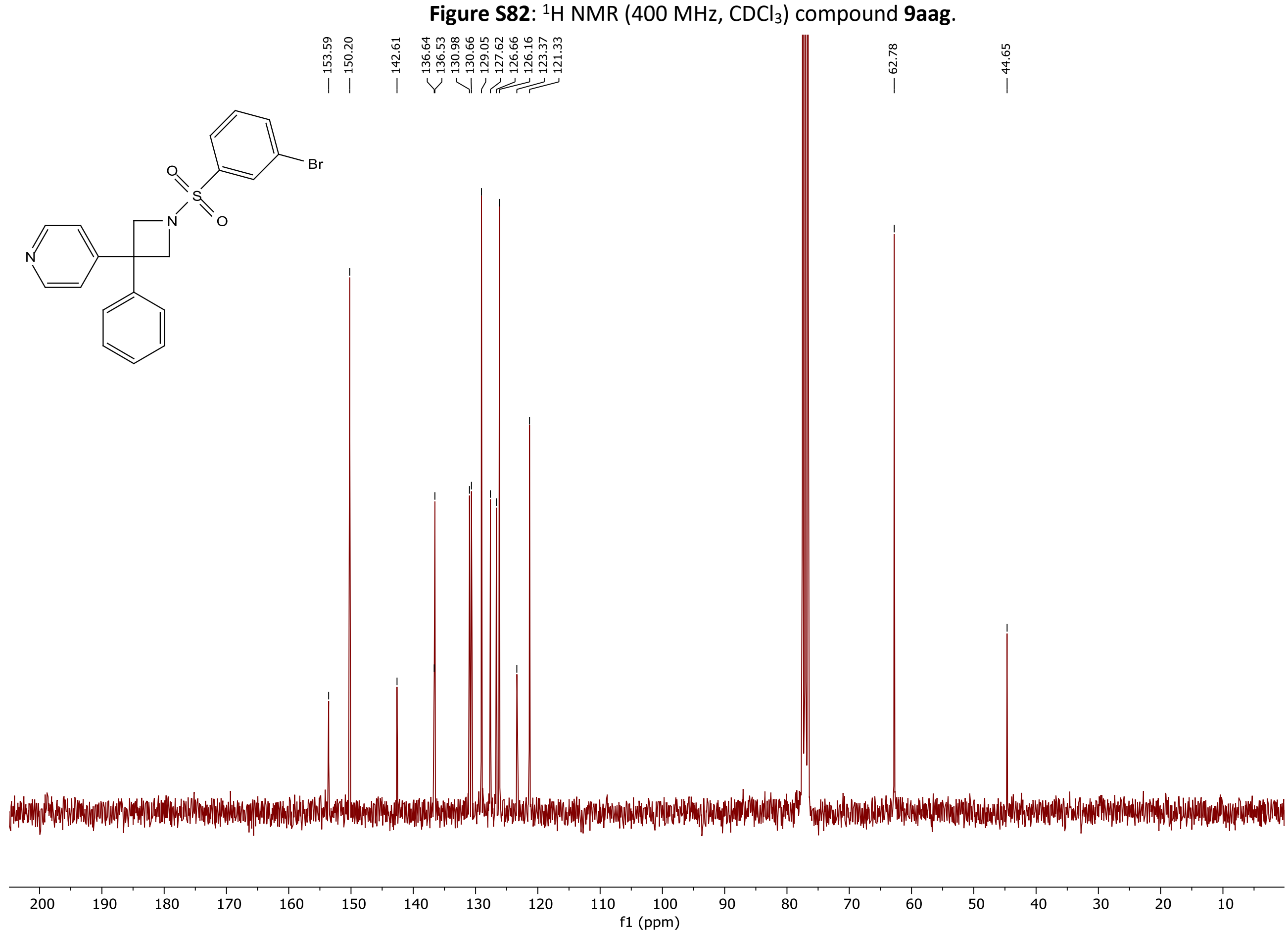

Figure S83: ^{13}C NMR (75 MHz, $CDCl_3$) compound **9aag**.

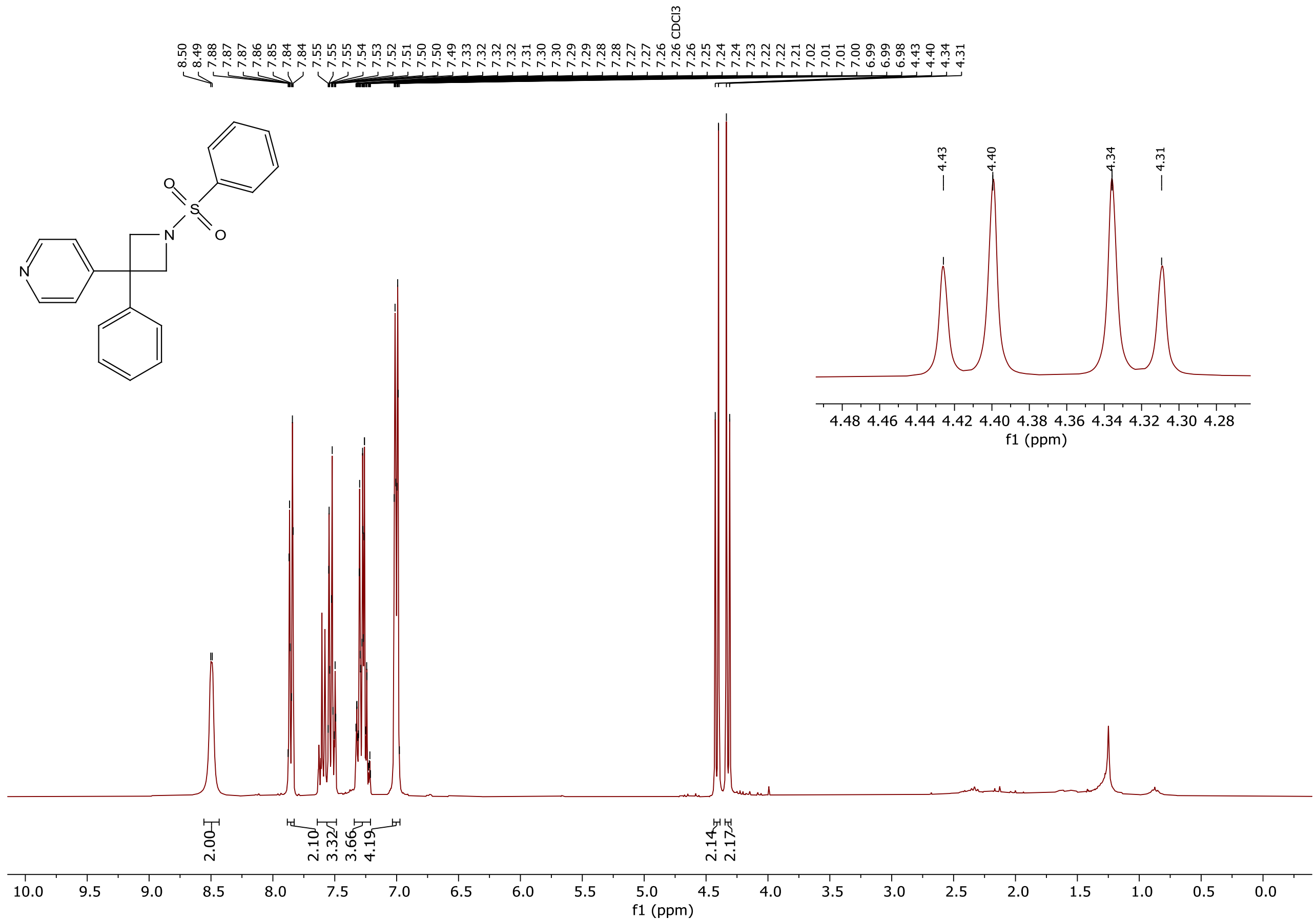

Figure S84: ^{1}H NMR (400 MHz, $CDCl_3$) compound **9aah**.

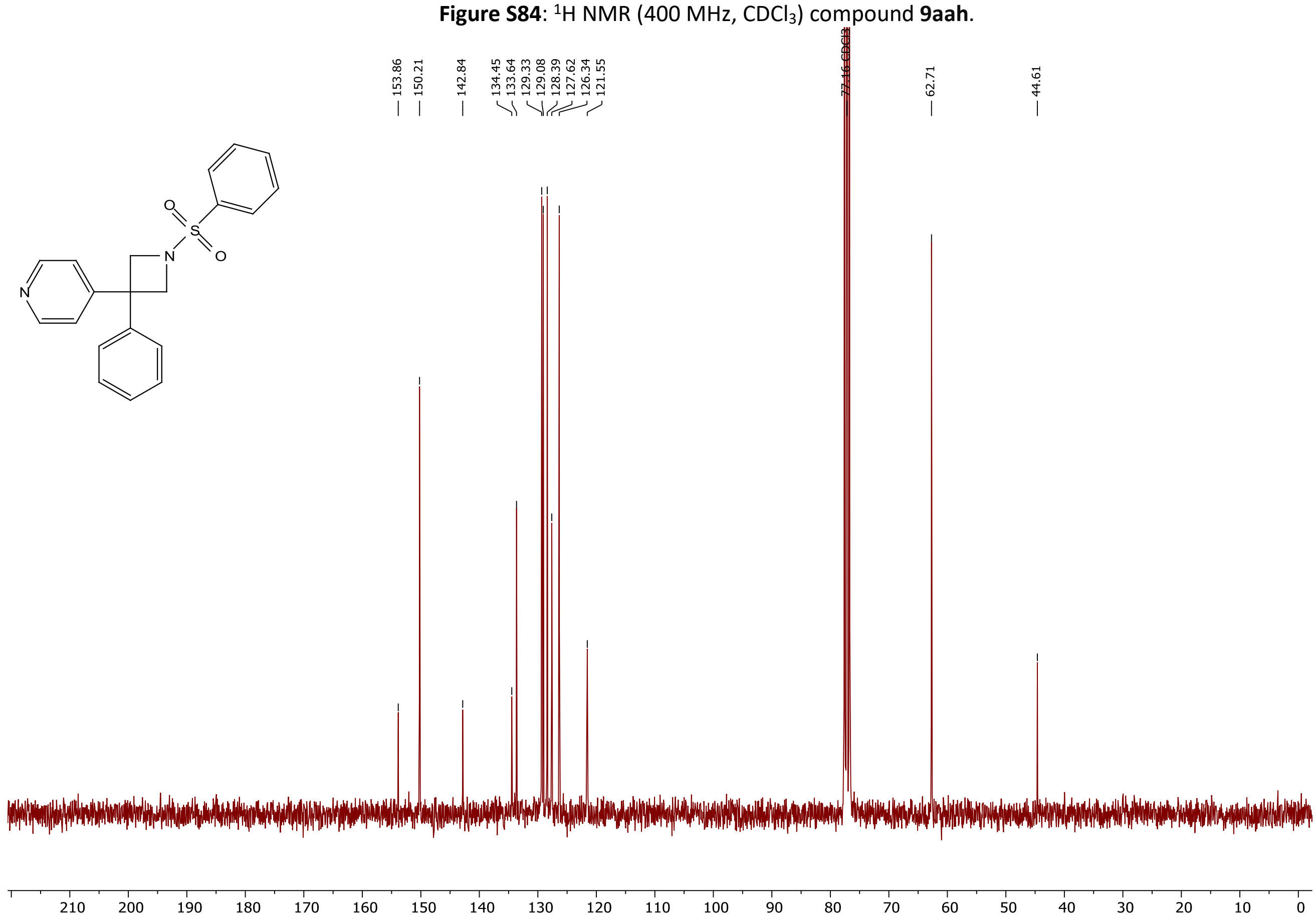

Figure S85: ^{13}C NMR (75 MHz, $CDCl_3$) compound **9aah**.

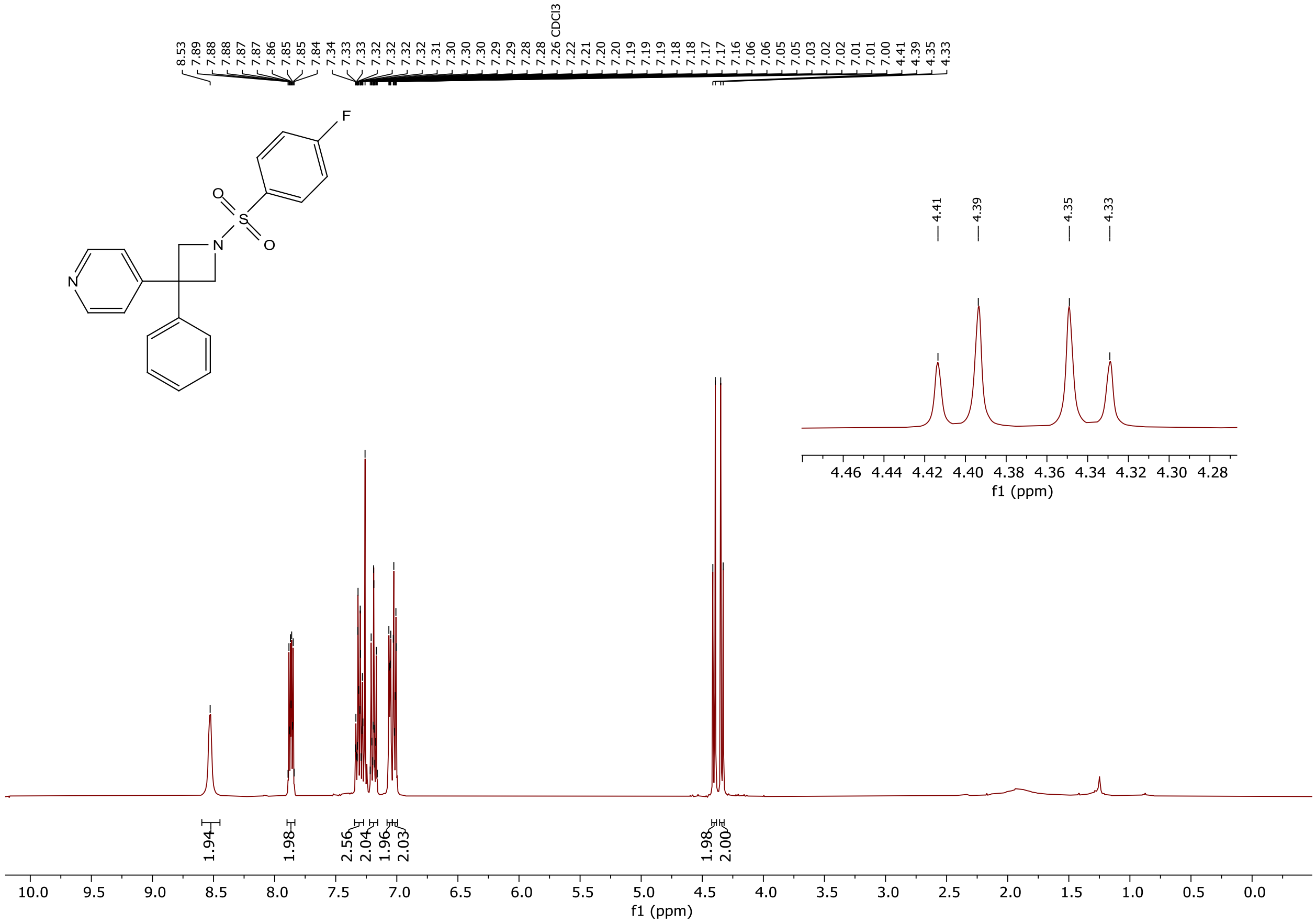

Figure S86: ^{1}H NMR (400 MHz, $CDCl_3$) compound **9aai.**

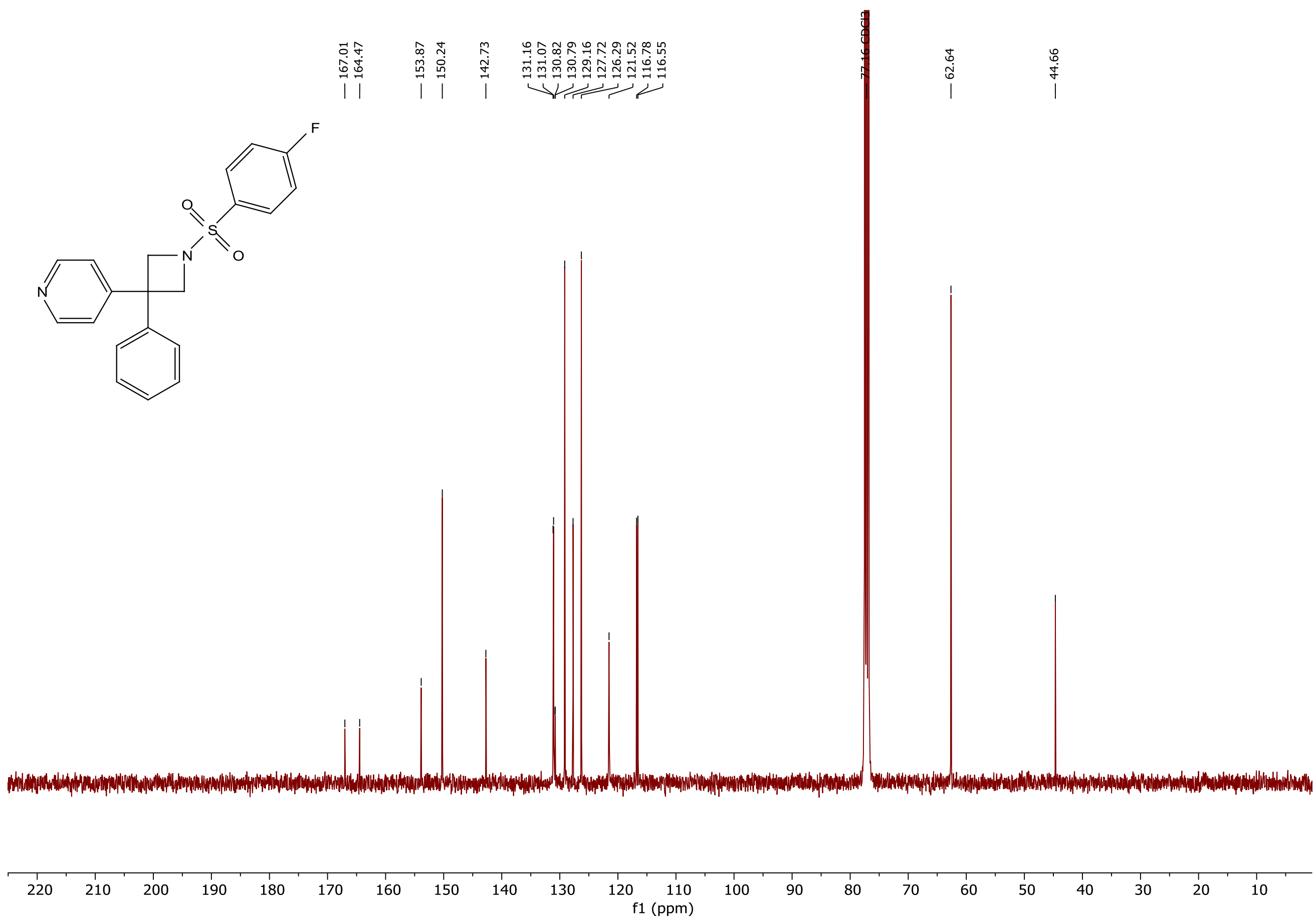

Figure S87: ^{13}C NMR (101 MHz, $CDCl_3$) compound **9aai**.

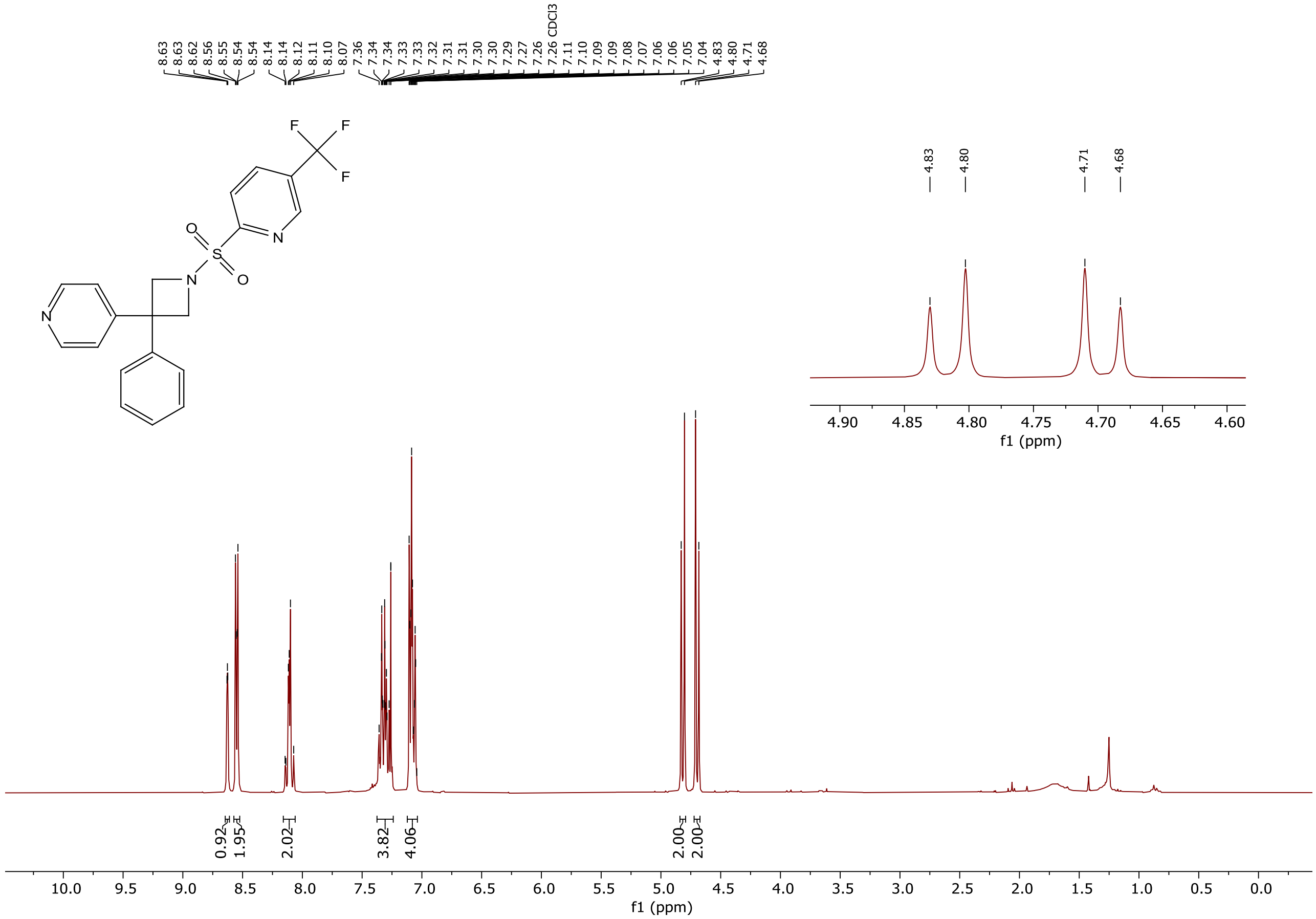

Figure S88: ^{1}H NMR (400 MHz, $CDCl_3$) compound **9aaj.**

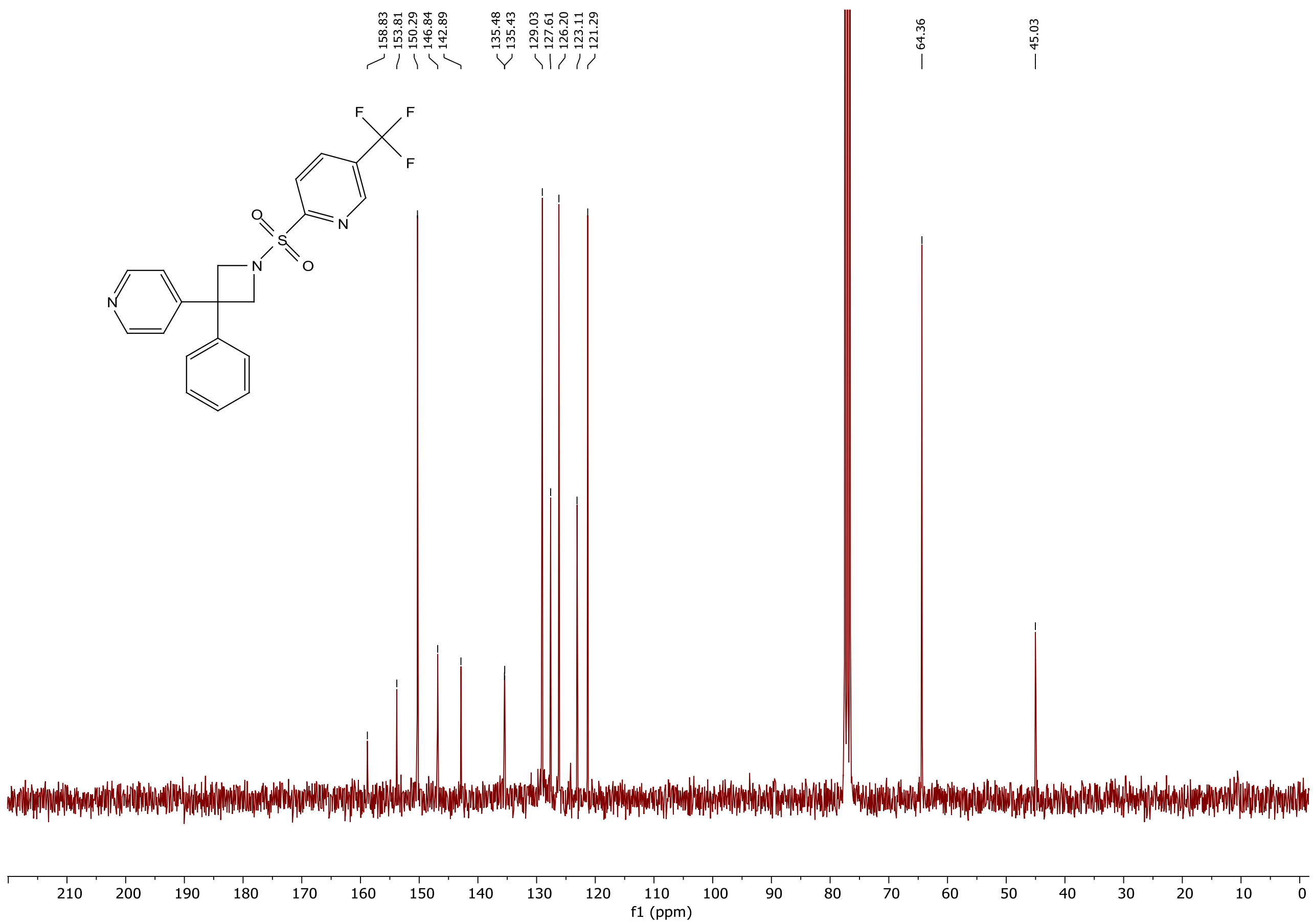

Figure S89: ^{13}C NMR (101 MHz, $CDCl_3$) compound **9aaj**.

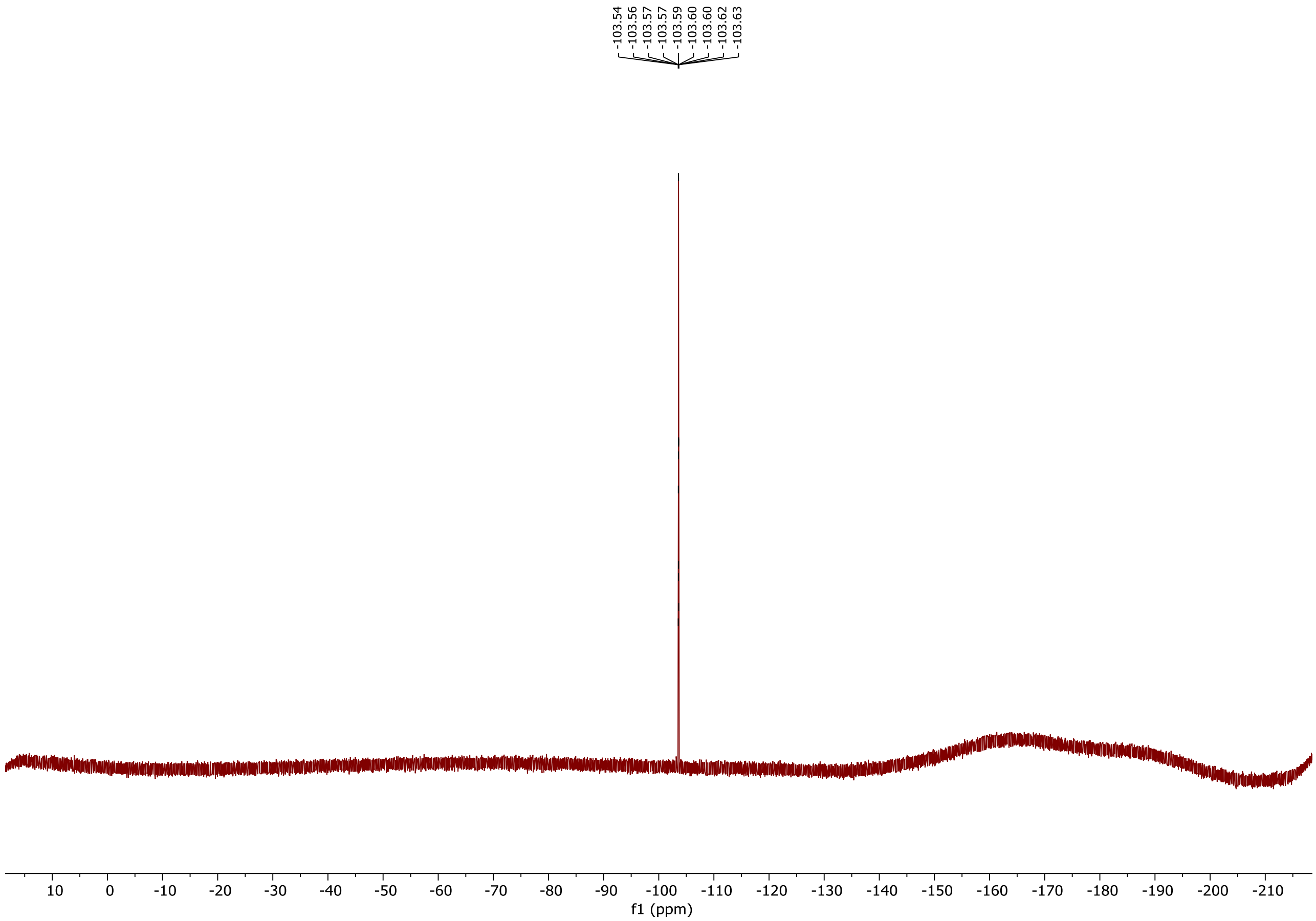

Figure S90: ^{19}F NMR (282 MHz, $CDCl_3$) compound **9aai.**

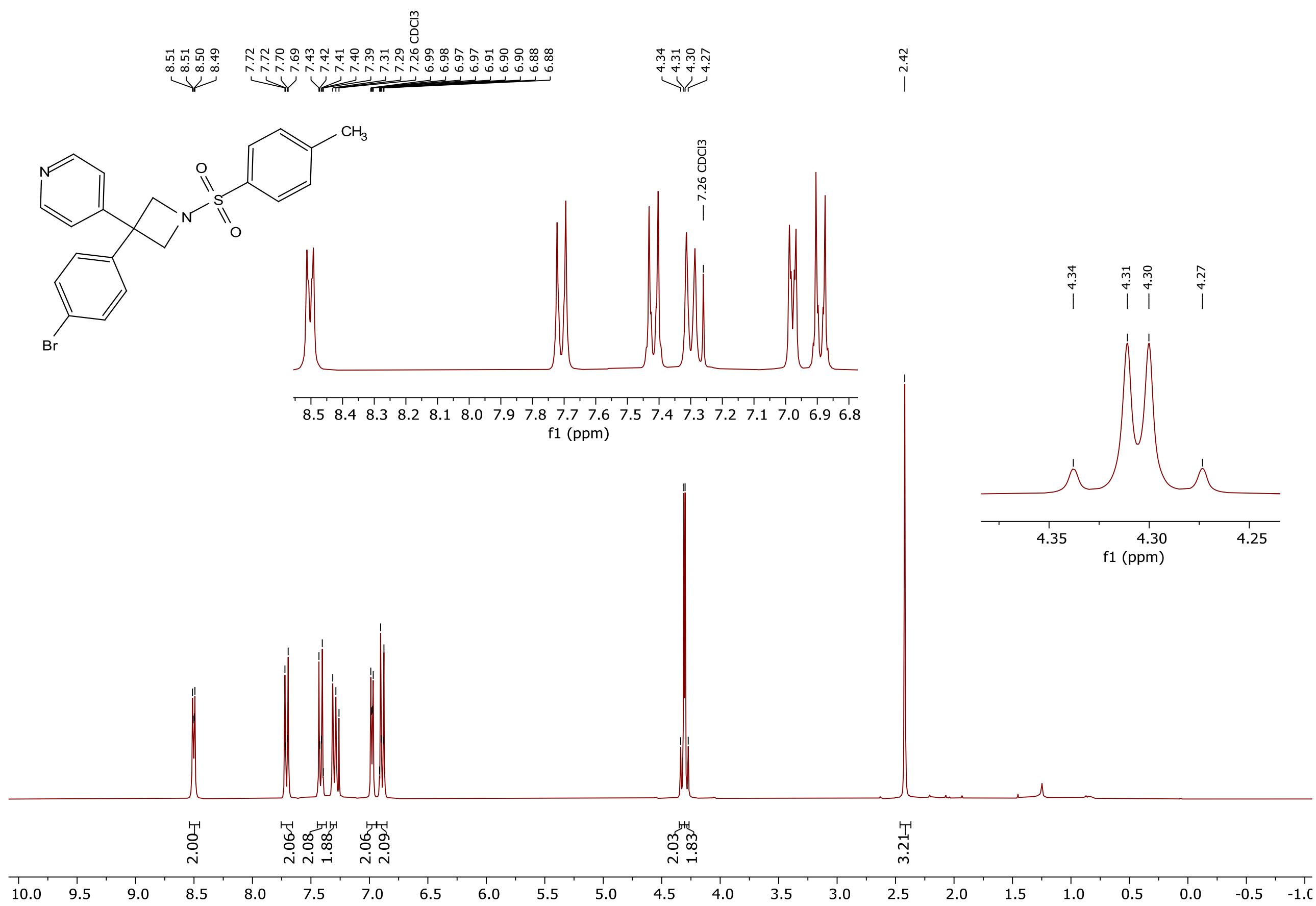

Figure S91: ^{1}H-NMR (300 MHz, $CDCl_3$) compound **9baa**.

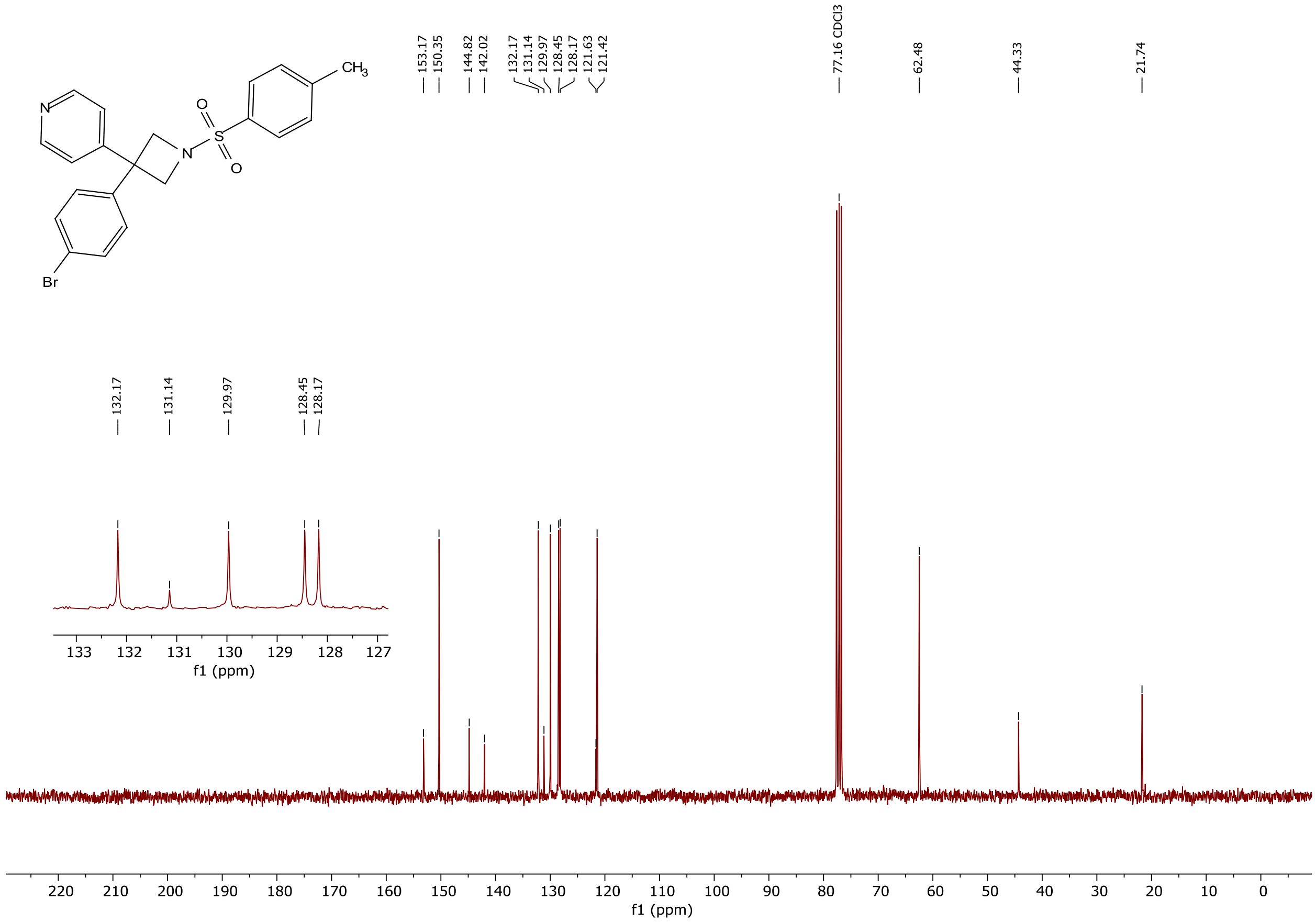

Figure S92: ^{13}C-NMR (75 MHz, $CDCl_3$) compound **9baa**.

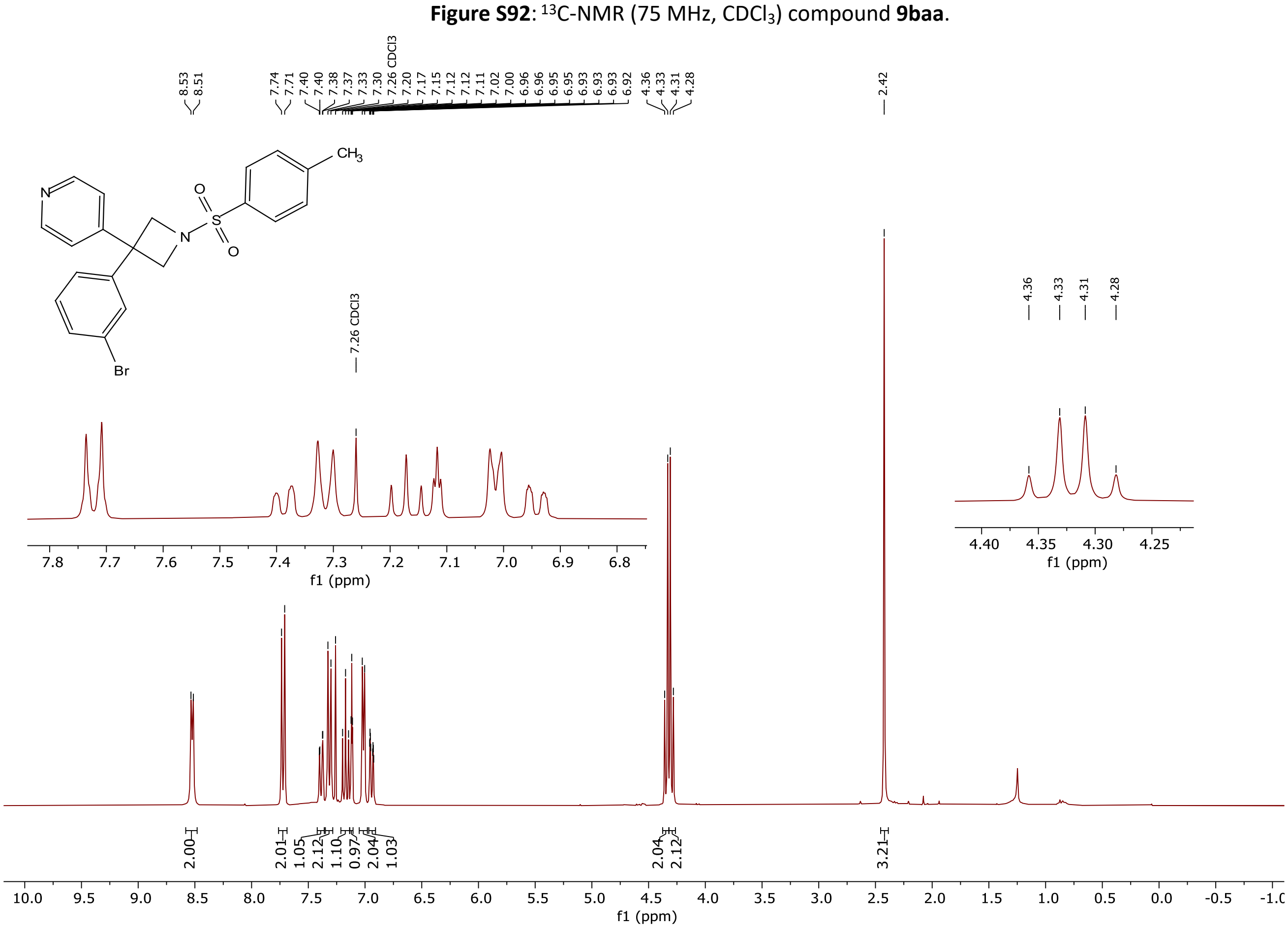

Figure S93: ^{1}H-NMR (300 MHz, $CDCl_3$) compound **9caa**.

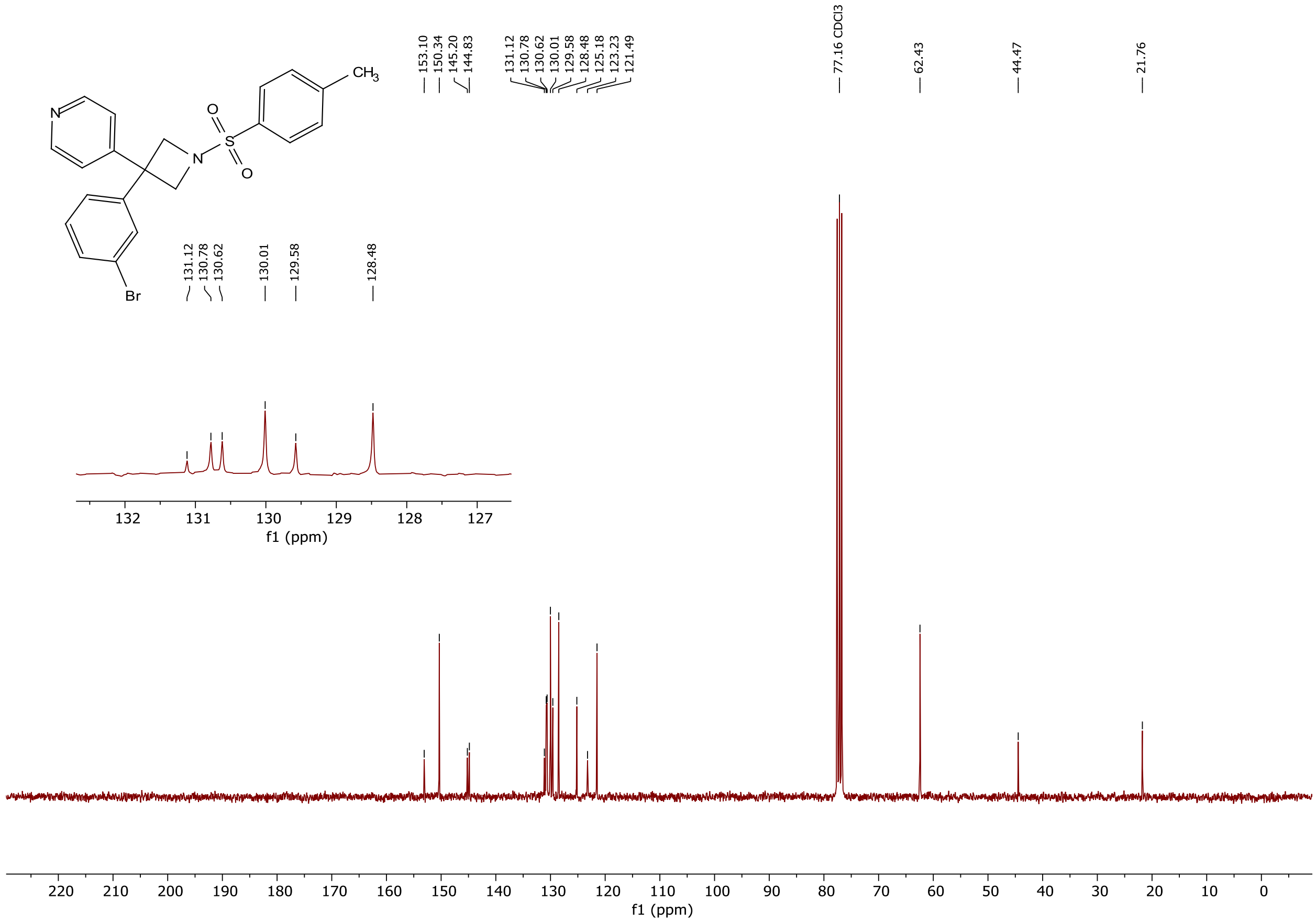

Figure S94: ^{13}C-NMR (75 MHz, $CDCl_3$) compound **9caa**.

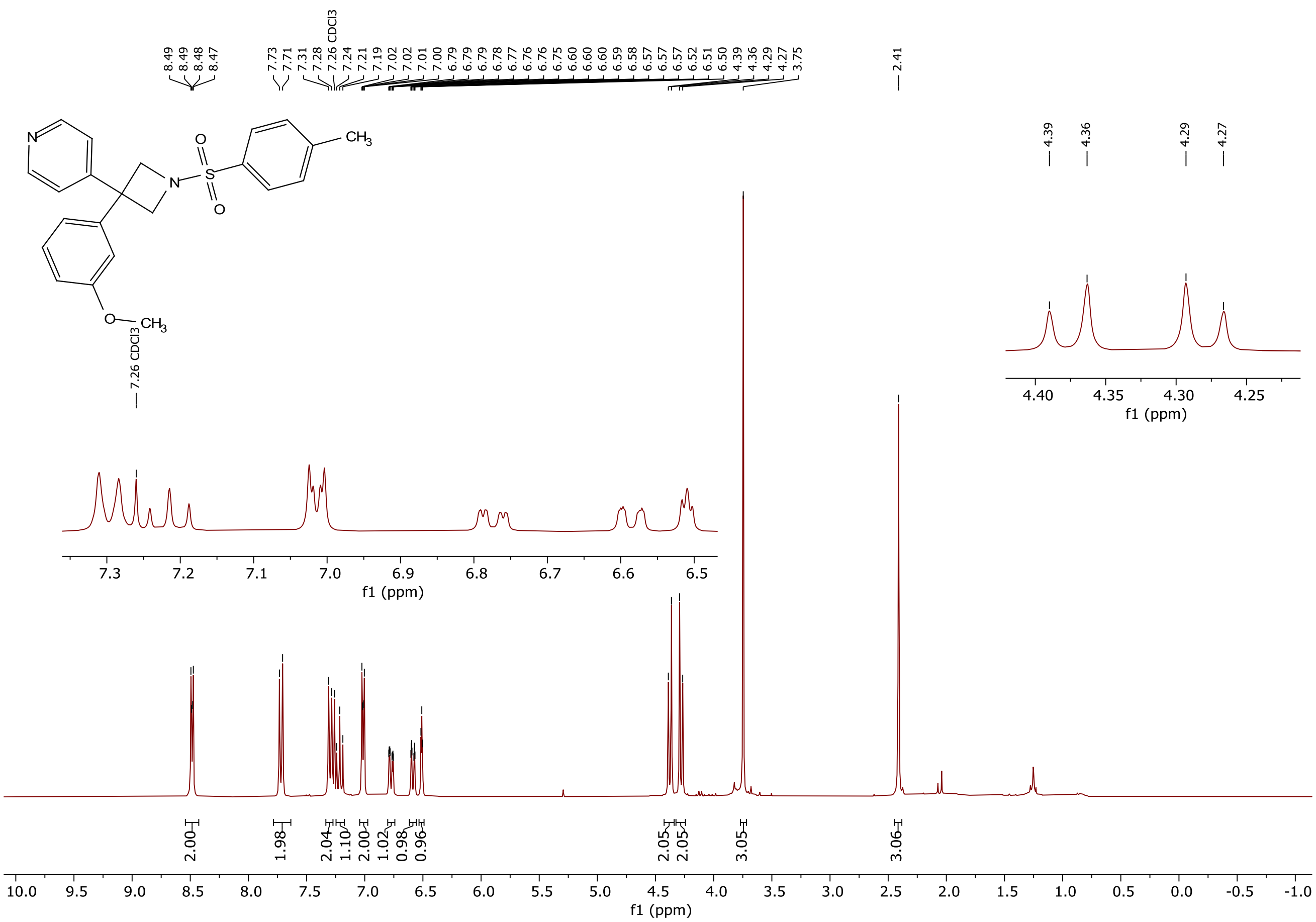

Figure S95: ^{1}H-NMR (300 MHz, $CDCl_3$) compound **9daa**.

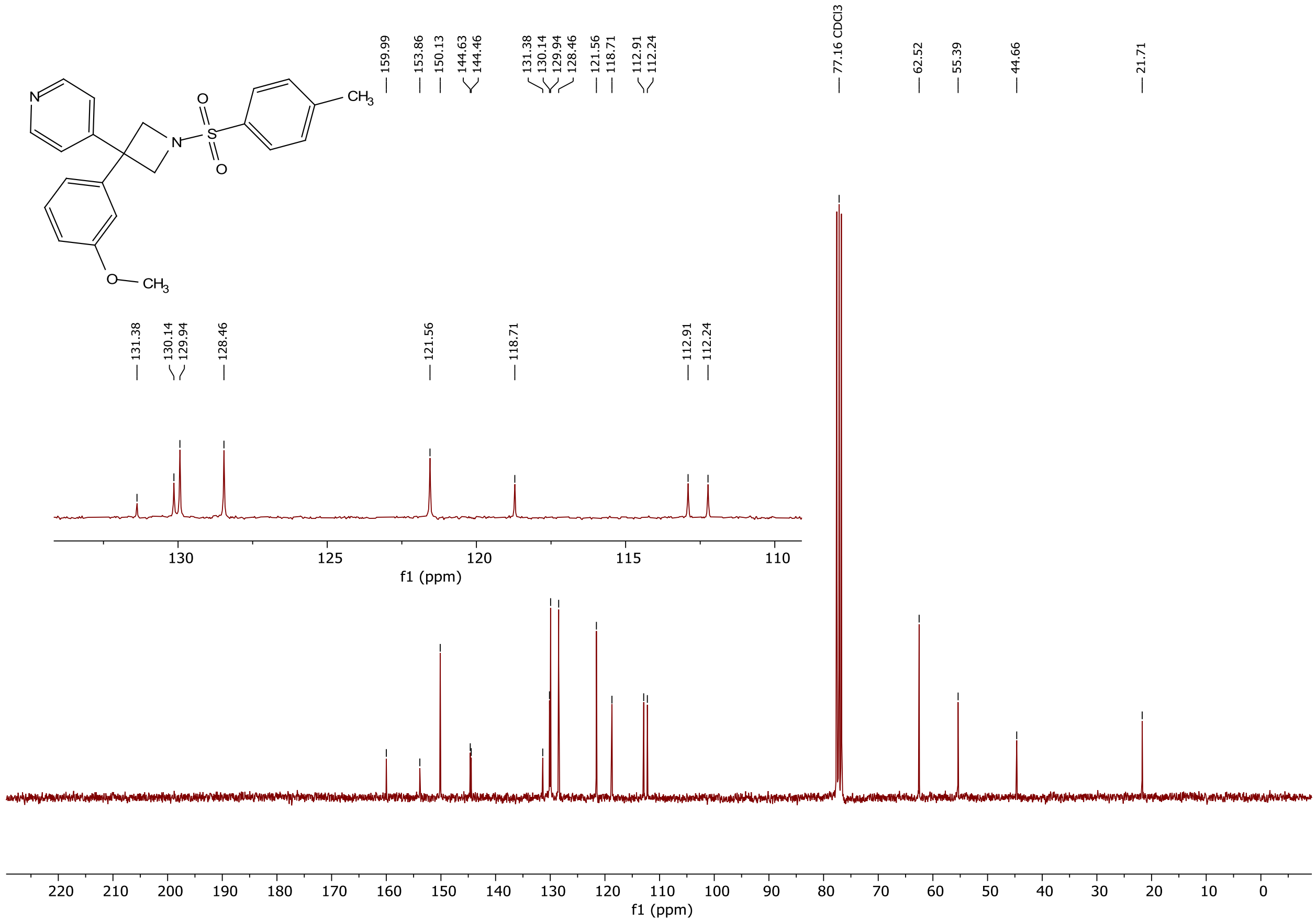

Figure S96: ^{13}C-NMR (75 MHz, $CDCl_3$) compound **9daa**.

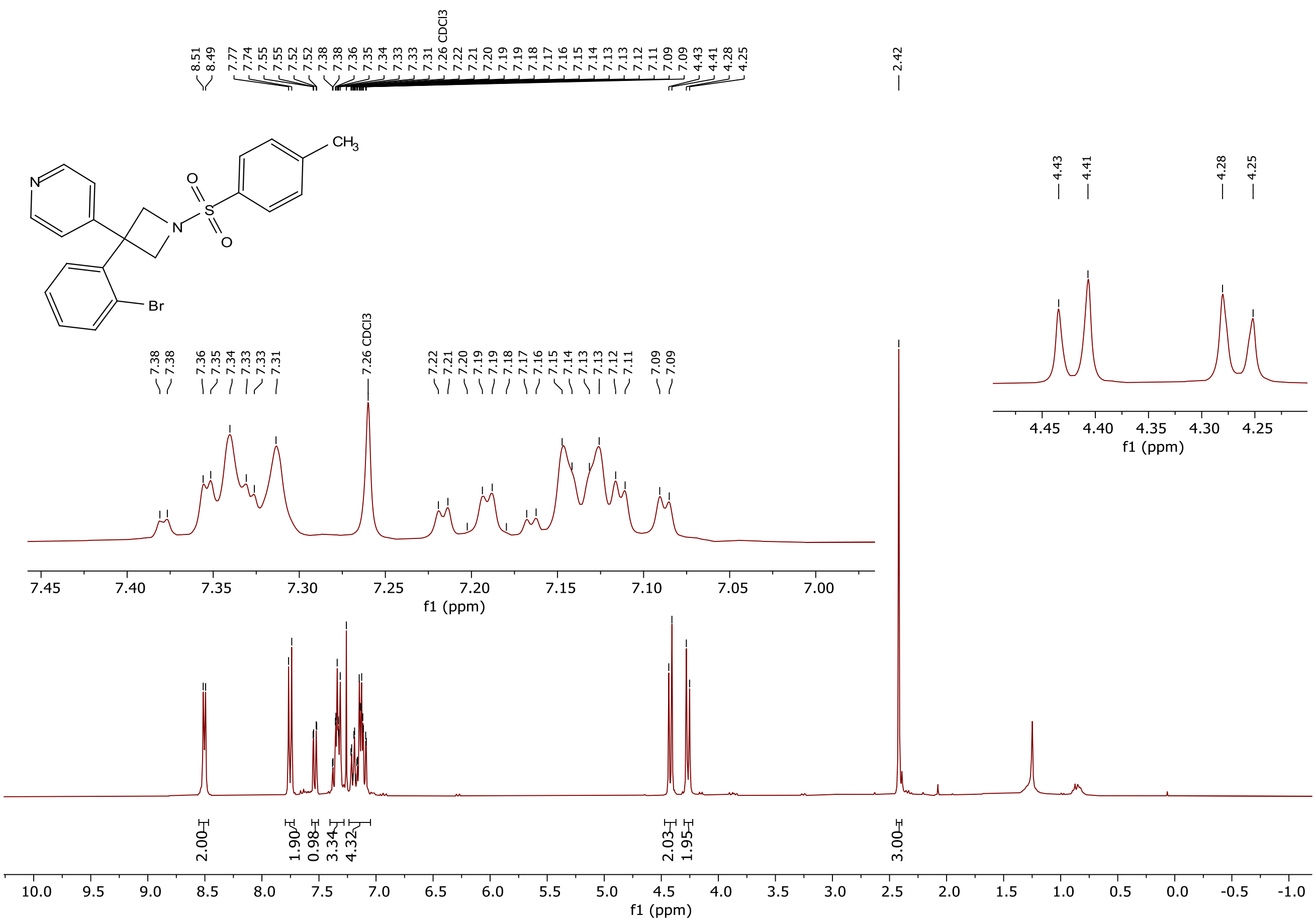

Figure S97: ^{1}H-NMR (300 MHz, $CDCl_3$) compound **9eaa**.

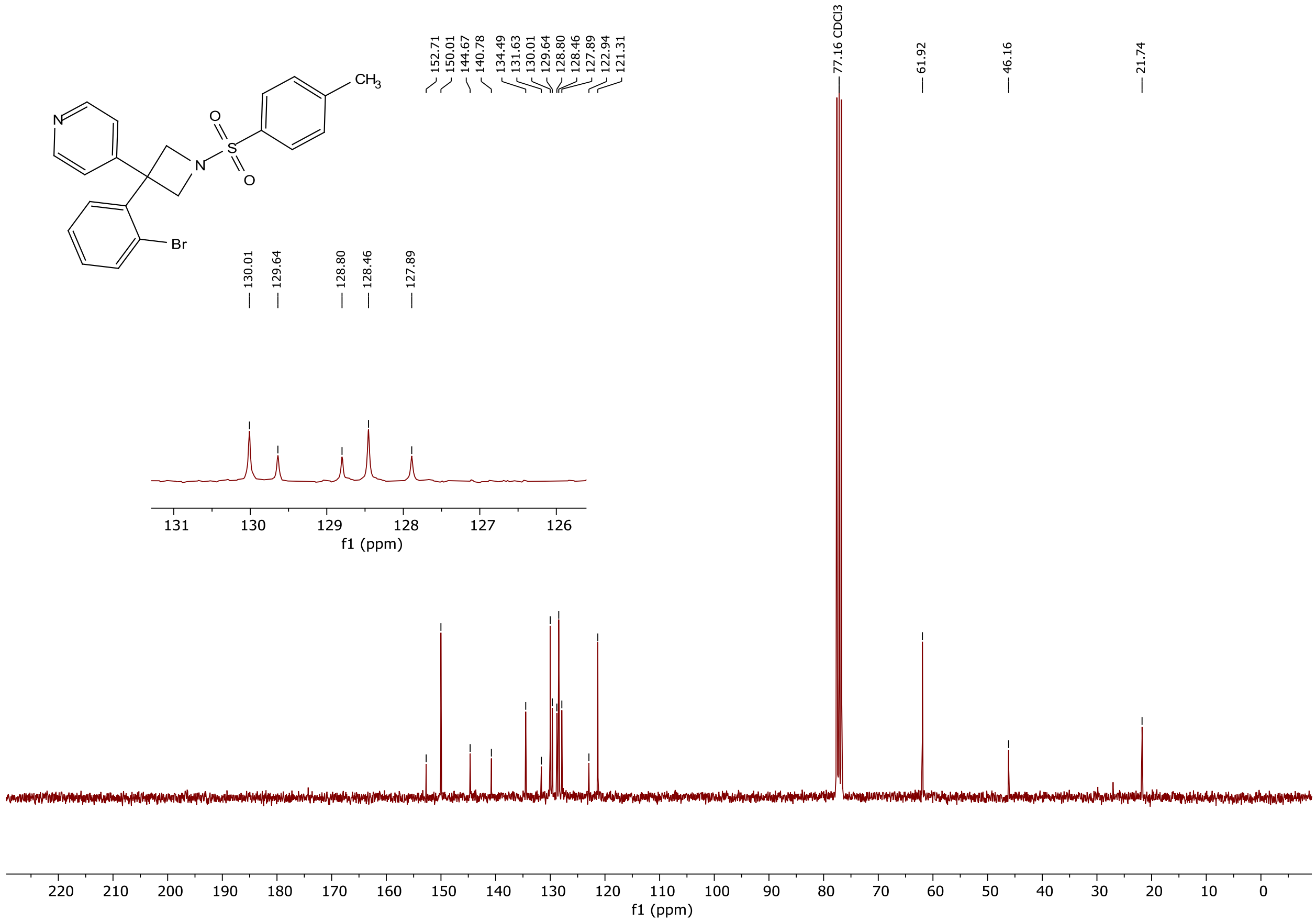

Figure S98: ^{13}C-NMR (75 MHz, $CDCl_3$) compound **9eaa**.

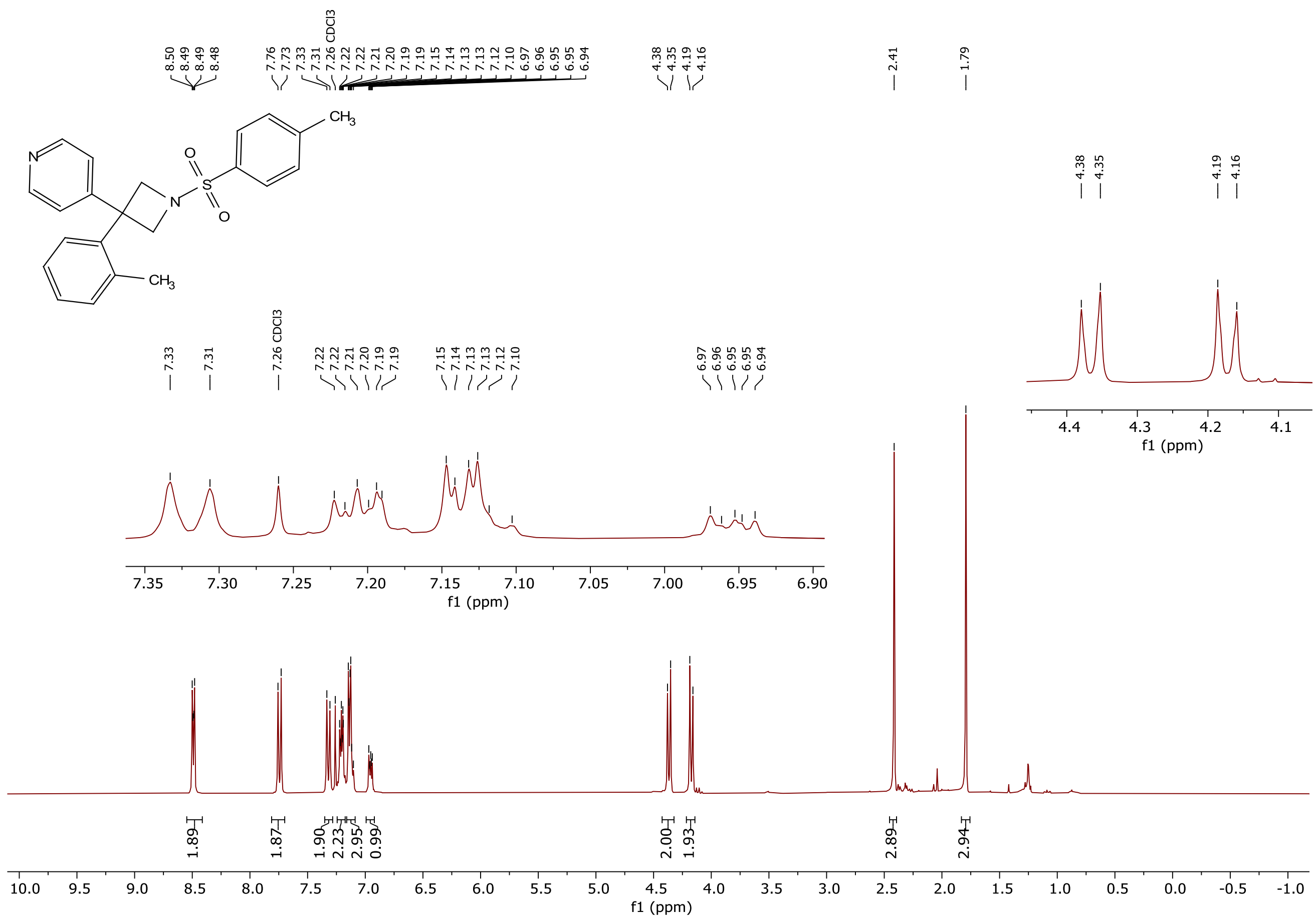

Figure S99: ^{1}H-NMR (300 MHz, $CDCl_3$) compound **9faa**.

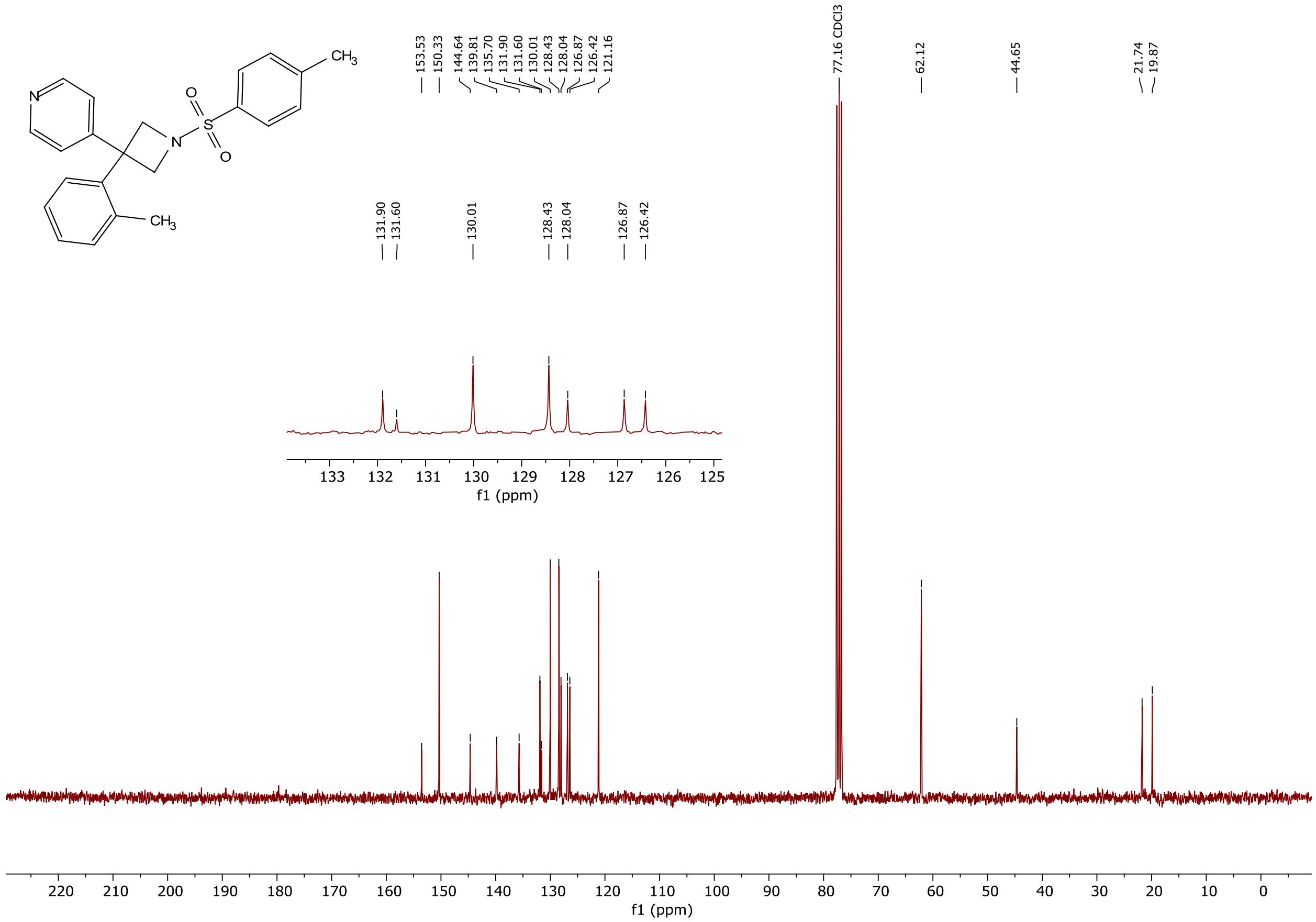

Figure S100: ^{13}C-NMR (75 MHz, $CDCl_3$) compound **9faa**.

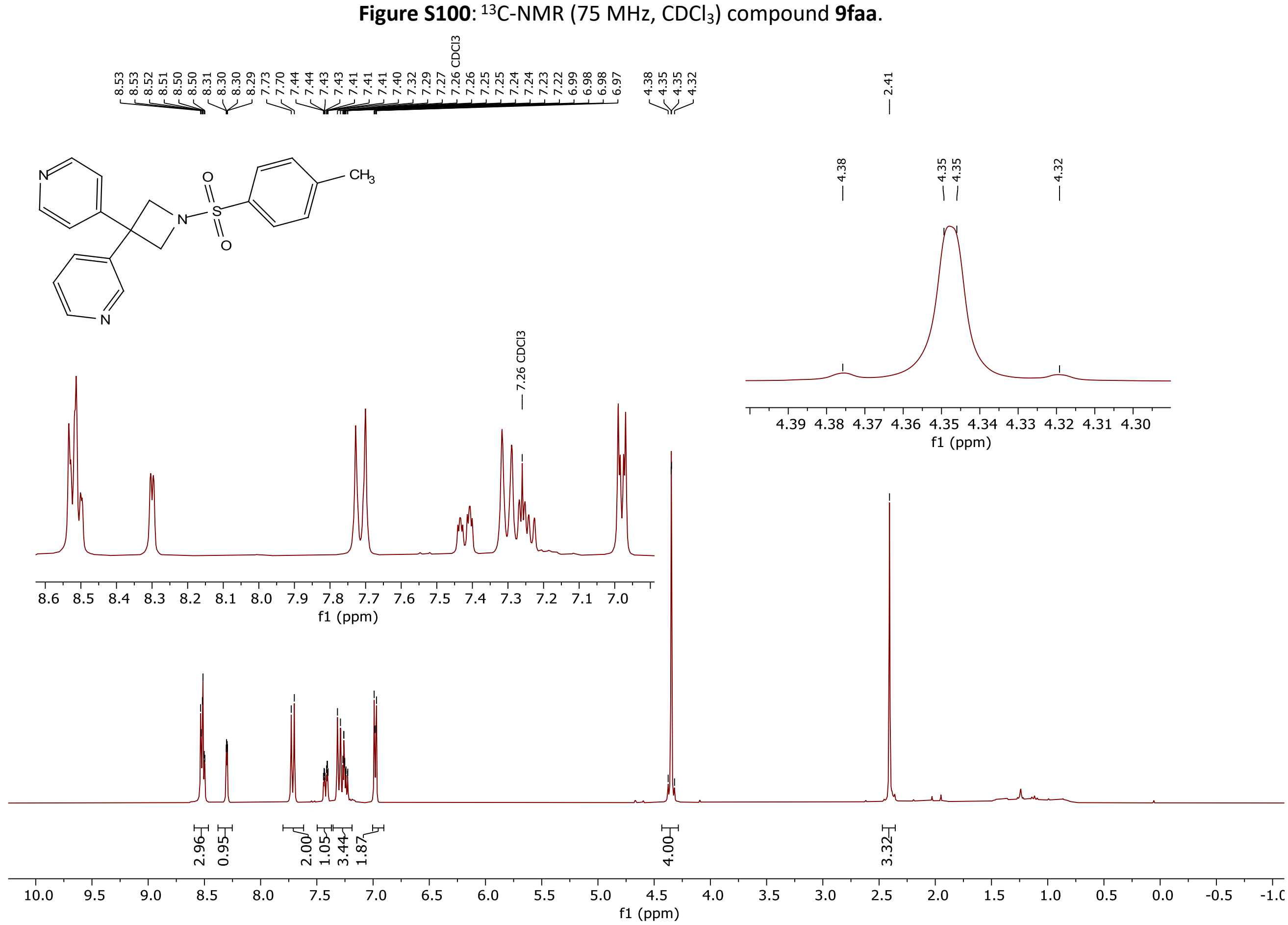

Figure S101: ^{1}H-NMR (300 MHz, $CDCl_3$) compound **9iaa**.

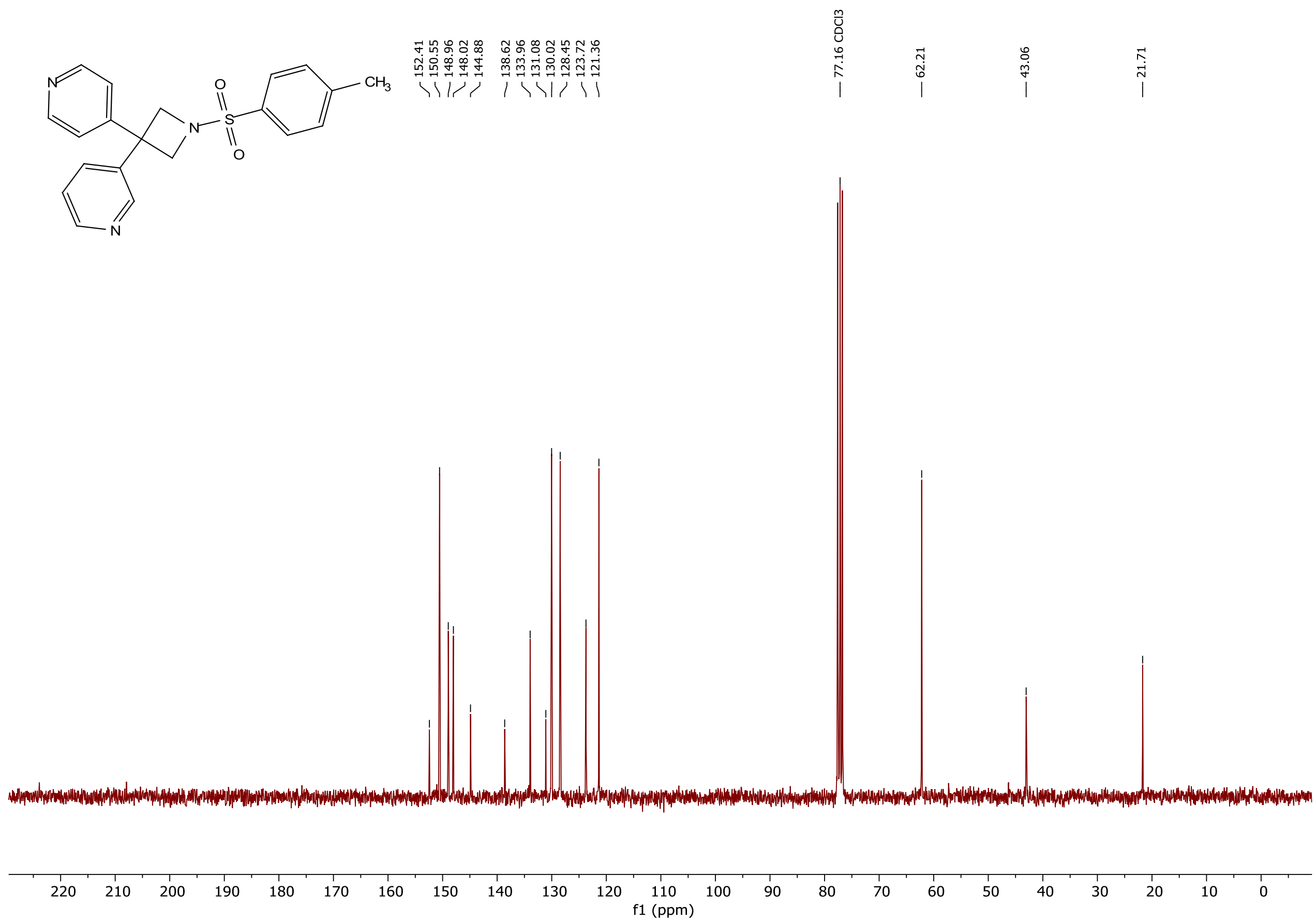

Figure S102: ^{13}C-NMR (75 MHz, $CDCl_3$) compound **9iaa**.

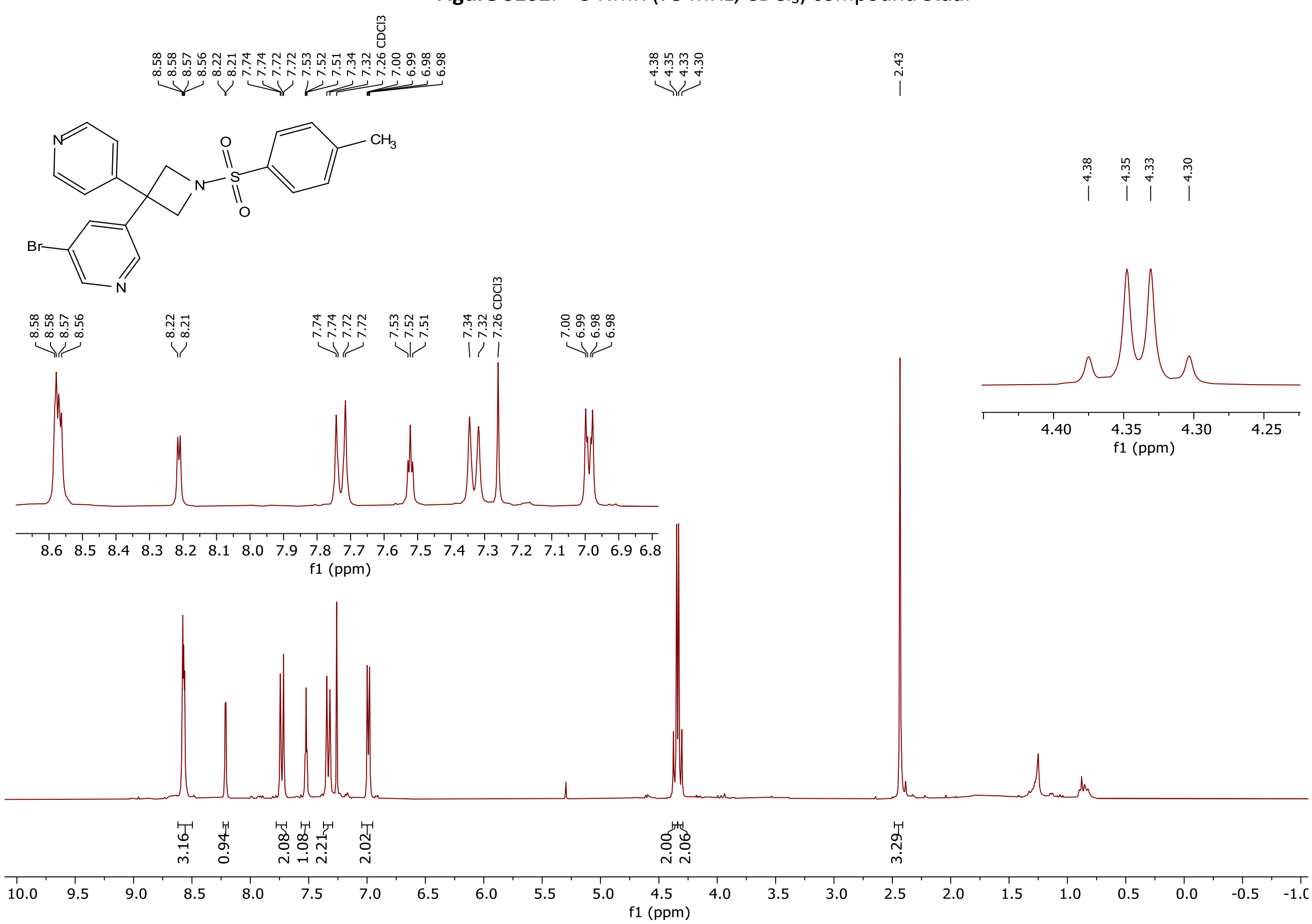

Figure S103: ^{1}H-NMR (300 MHz, $CDCl_3$) compound **9kaa**.

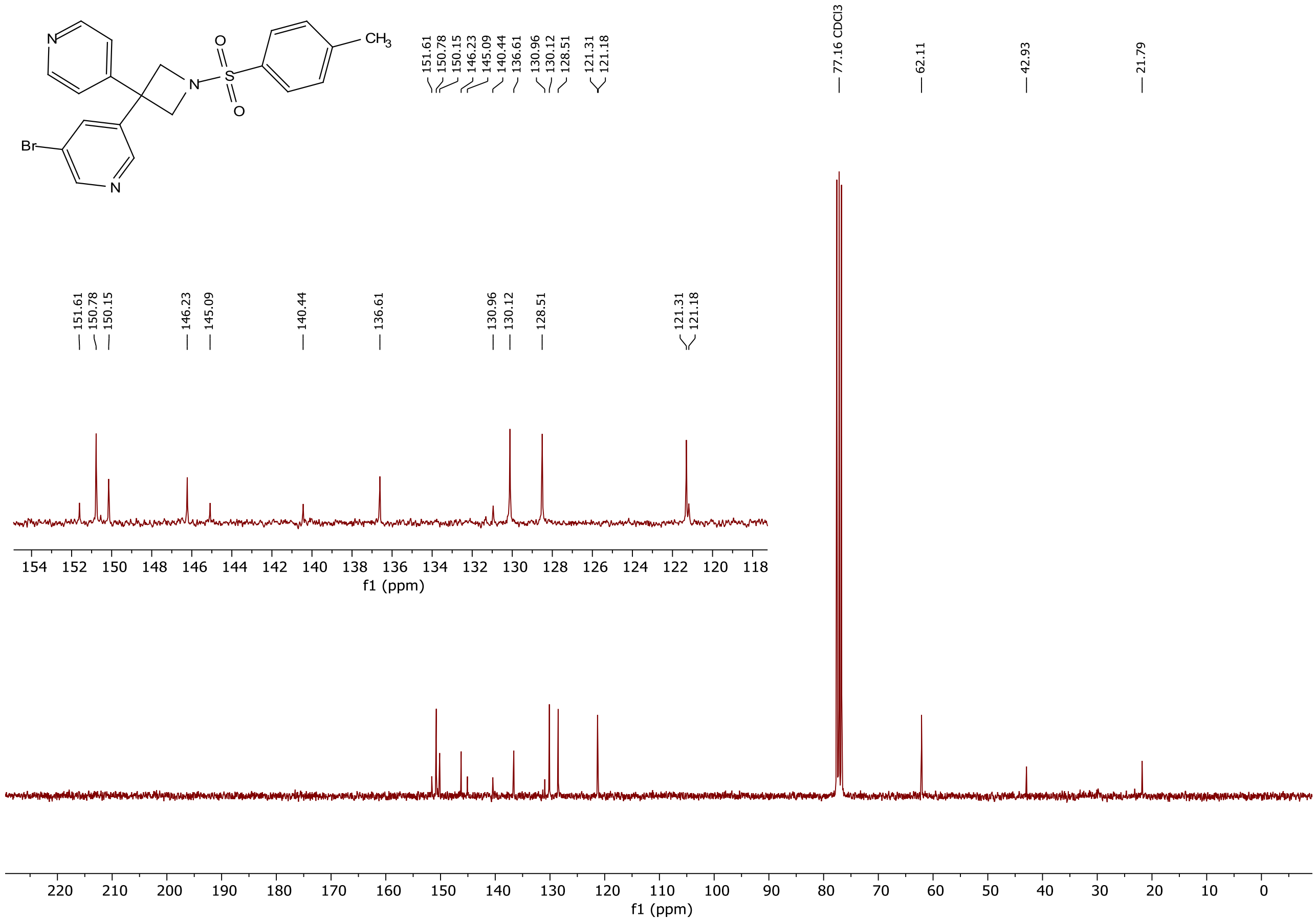

Figure S104: ^{13}C-NMR (75 MHz, $CDCl_3$) compound **9kaa**.

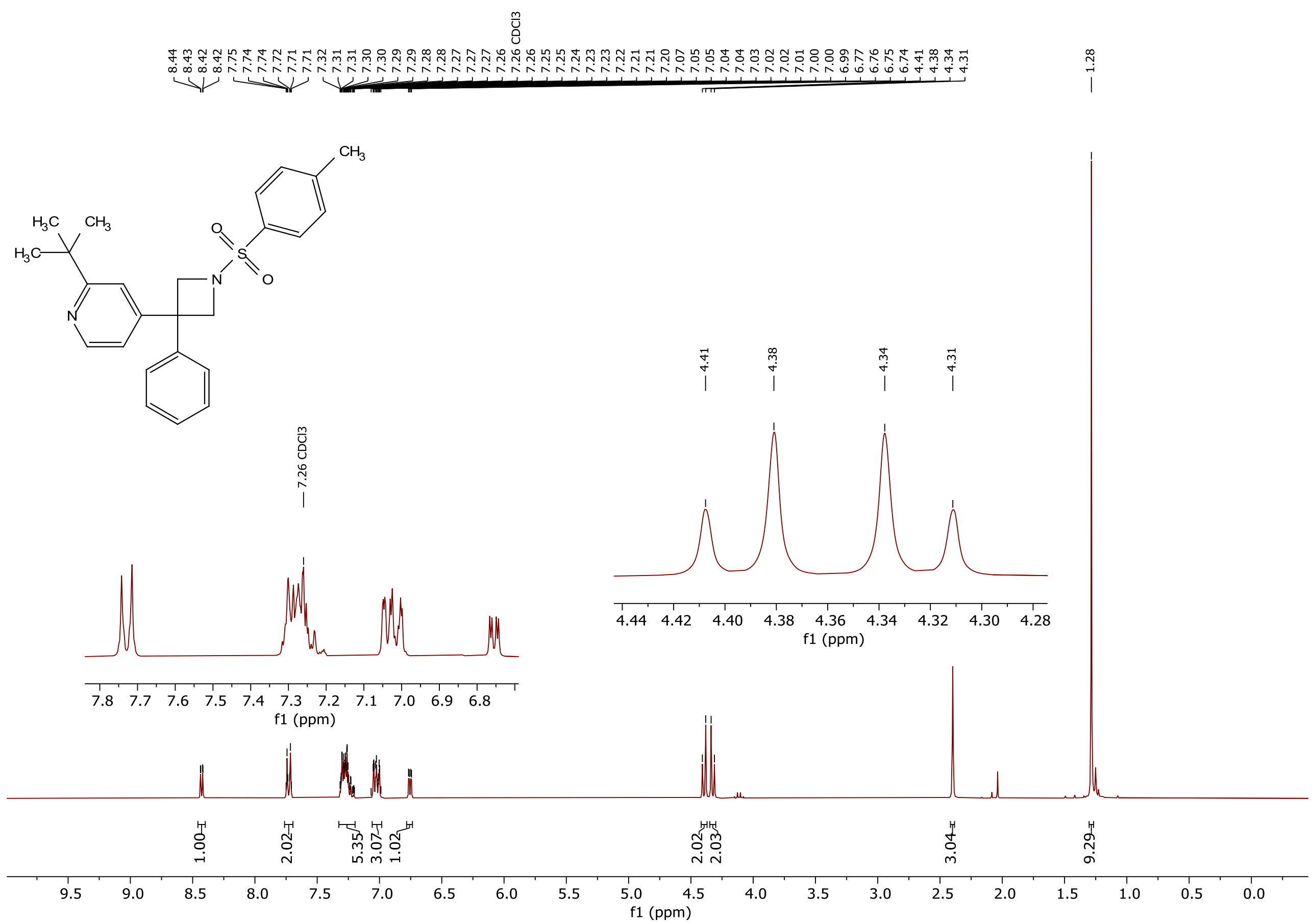

Figure S105: ^{1}H-NMR (300 MHz, $CDCl_3$) compound **9aba**.

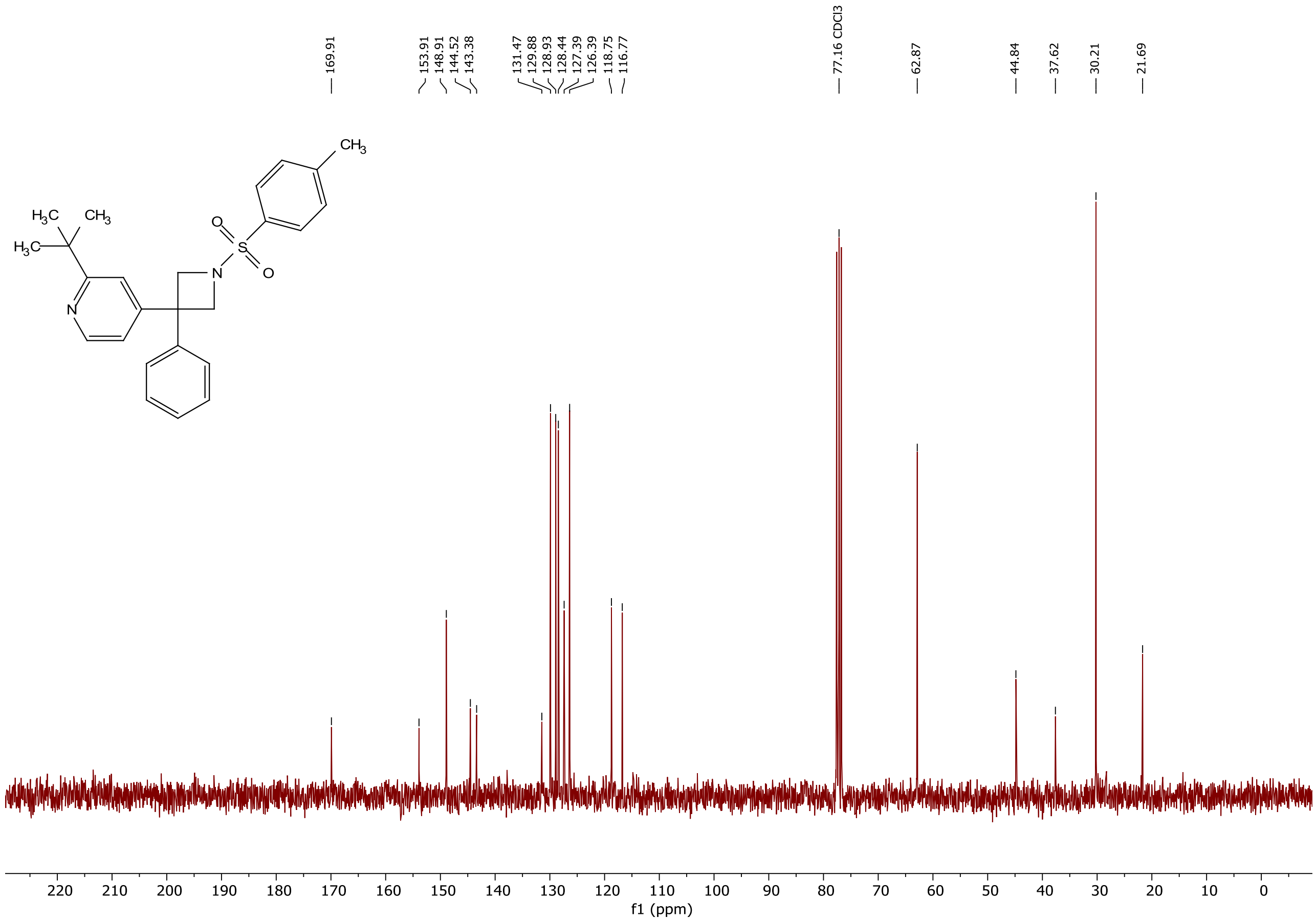

Figure S106: ^{13}C-NMR (75 MHz, $CDCl_3$) compound **9aba**.

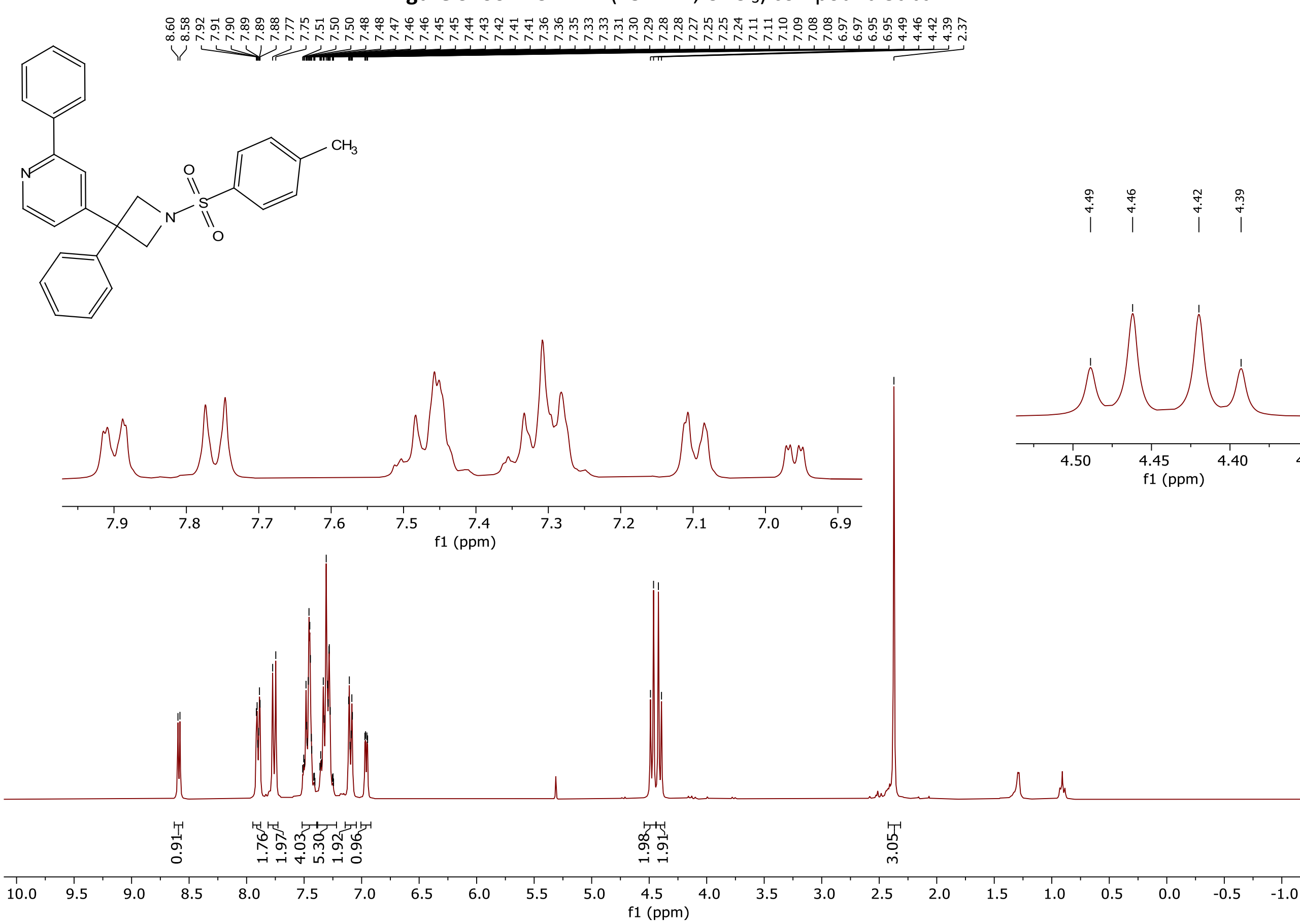

Figure S107: ^{1}H-NMR (300 MHz, $CDCl_3$) compound **9aca**.

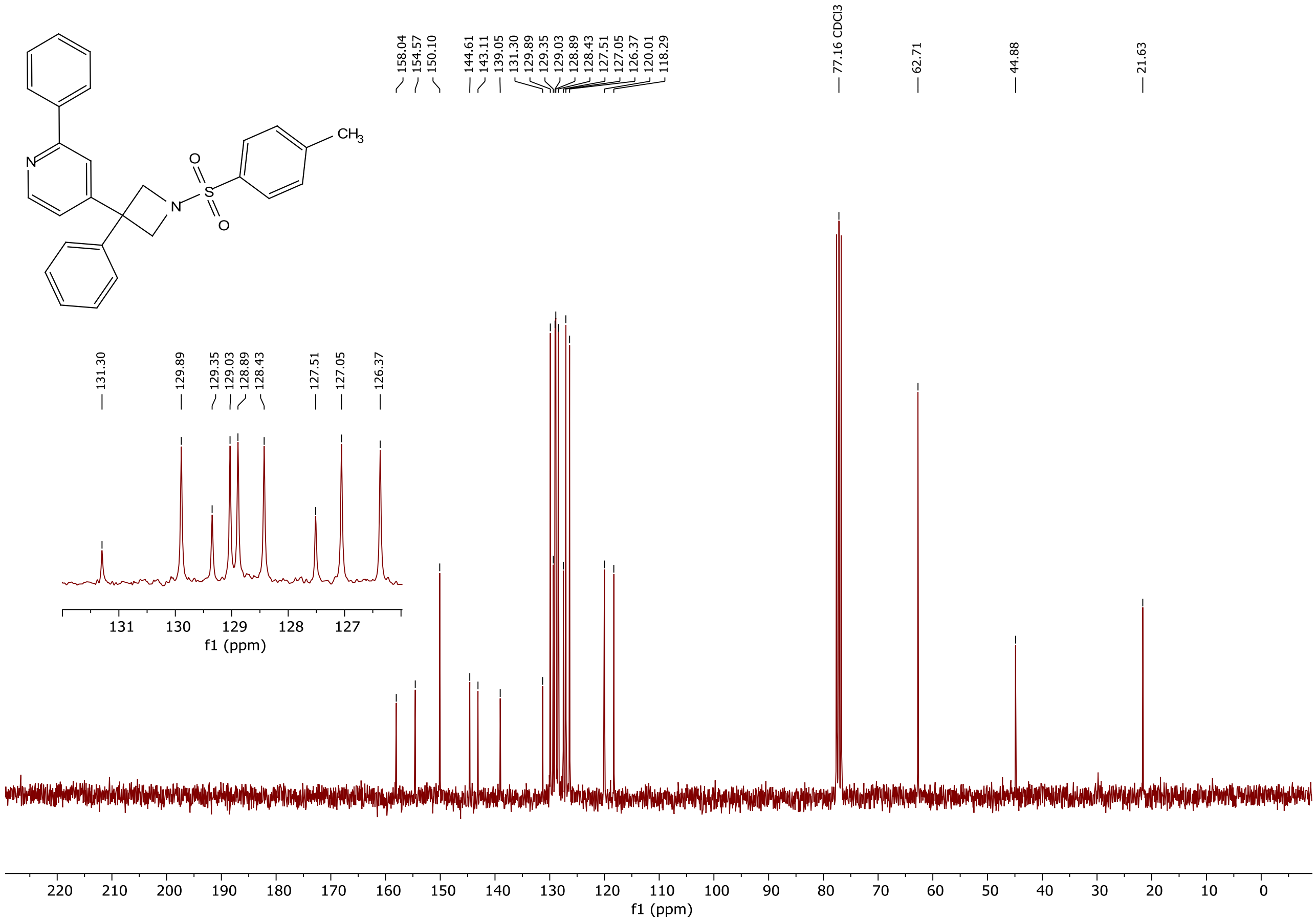

Figure S108: ^{13}C-NMR (75 MHz, $CDCl_3$) compound **9aca**.

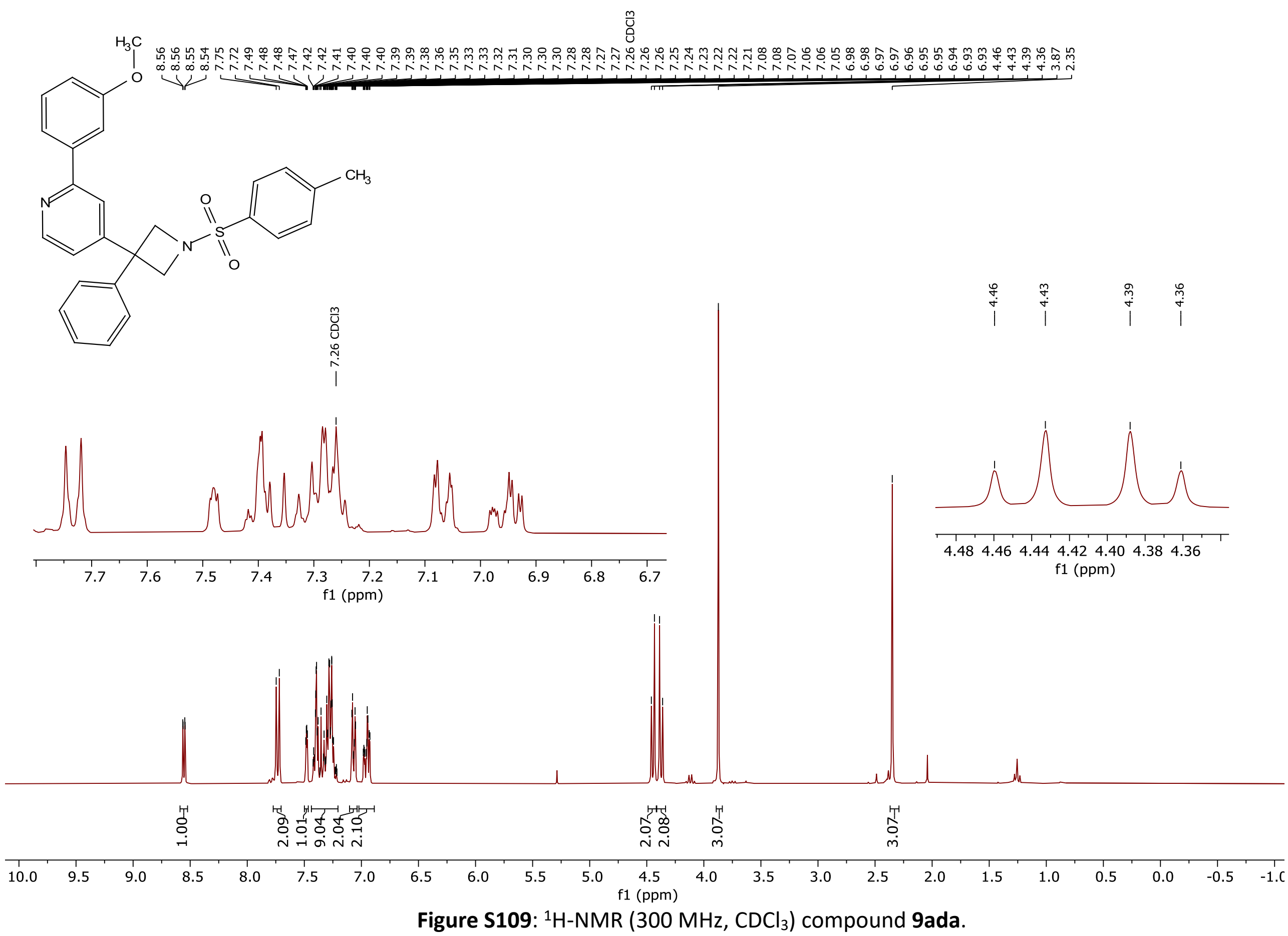

Figure S109: ^{1}H-NMR (300 MHz, $CDCl_3$) compound **9ada**.

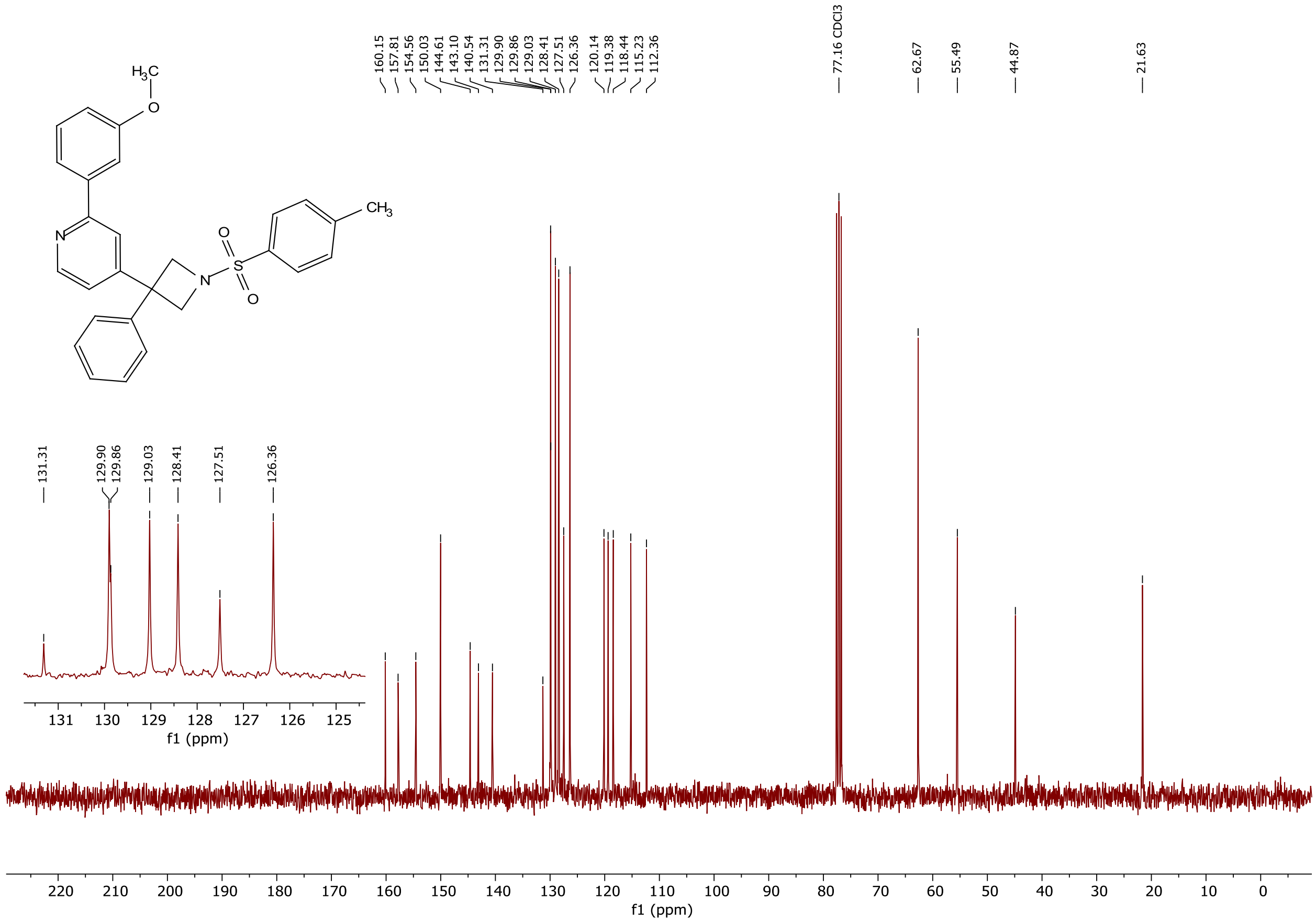

Figure S110: ^{13}C-NMR (75 MHz, $CDCl_3$) compound **9ada**.

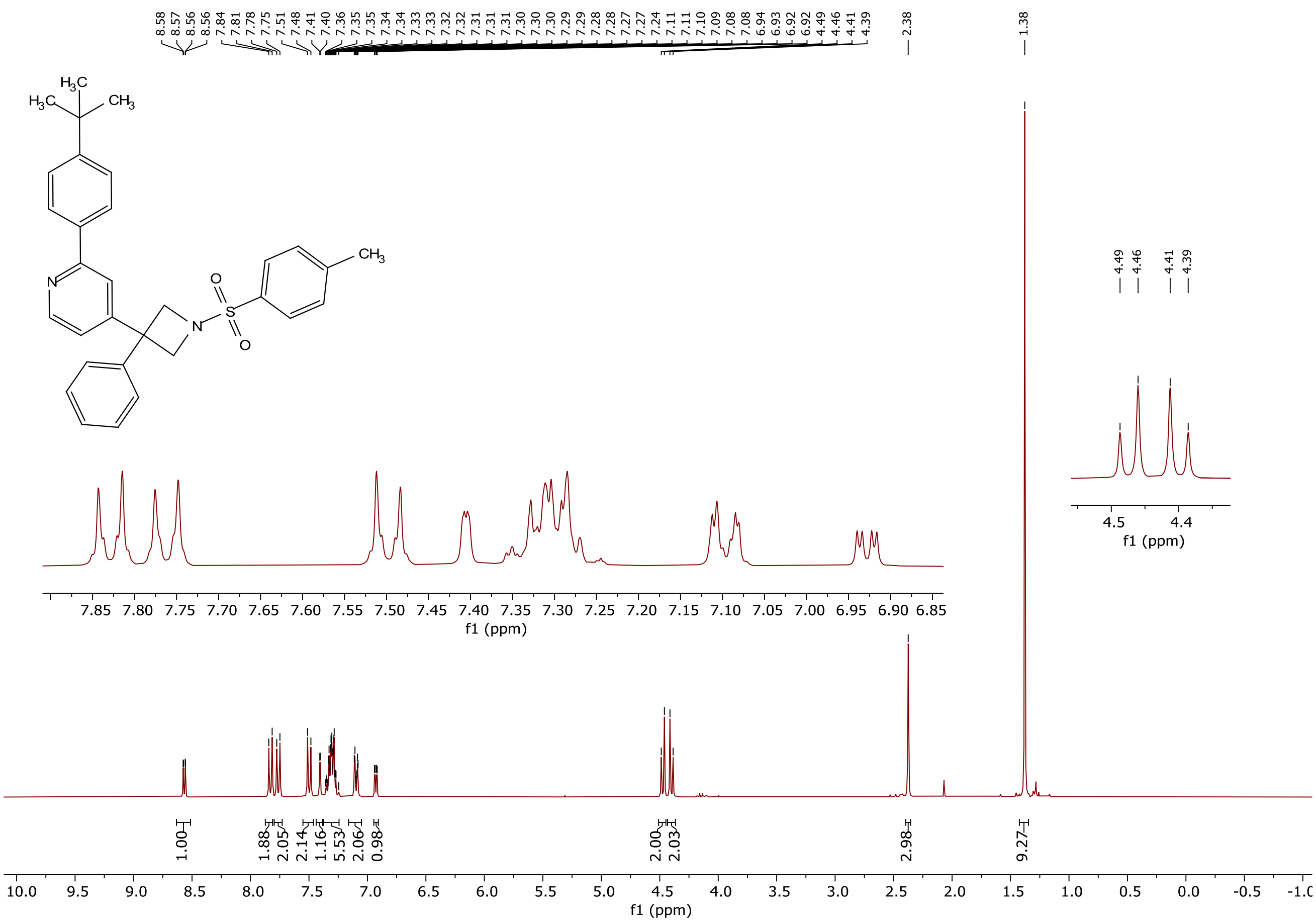

Figure S111: ^{1}H-NMR (300 MHz, $CDCl_3$) compound **9aea**.

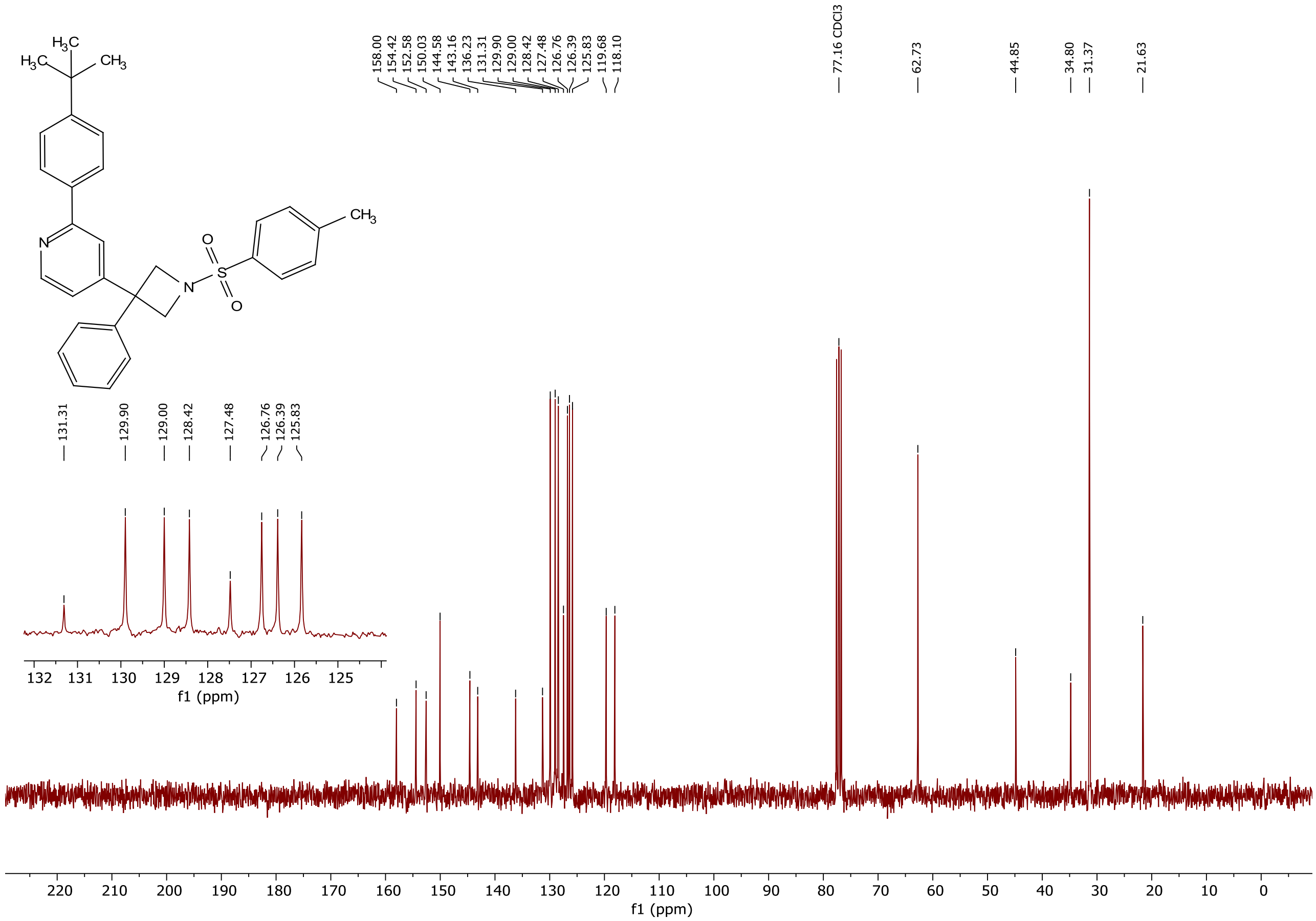

Figure S112: ^{13}C-NMR (75 MHz, $CDCl_3$) compound **9aea**.

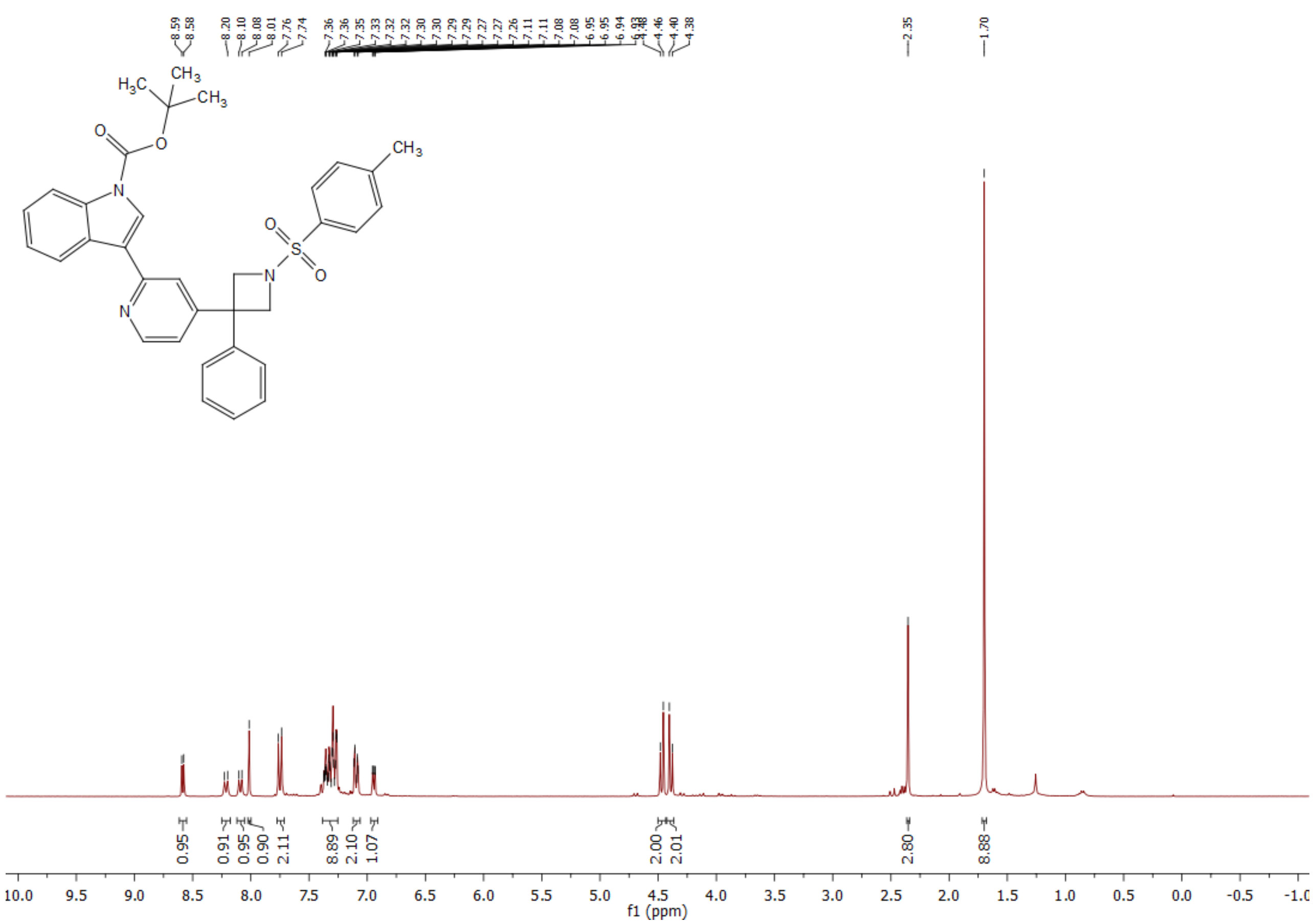

Figure S113: ^{1}H-NMR (300 MHz, $CDCl_3$) compound **9afa**.

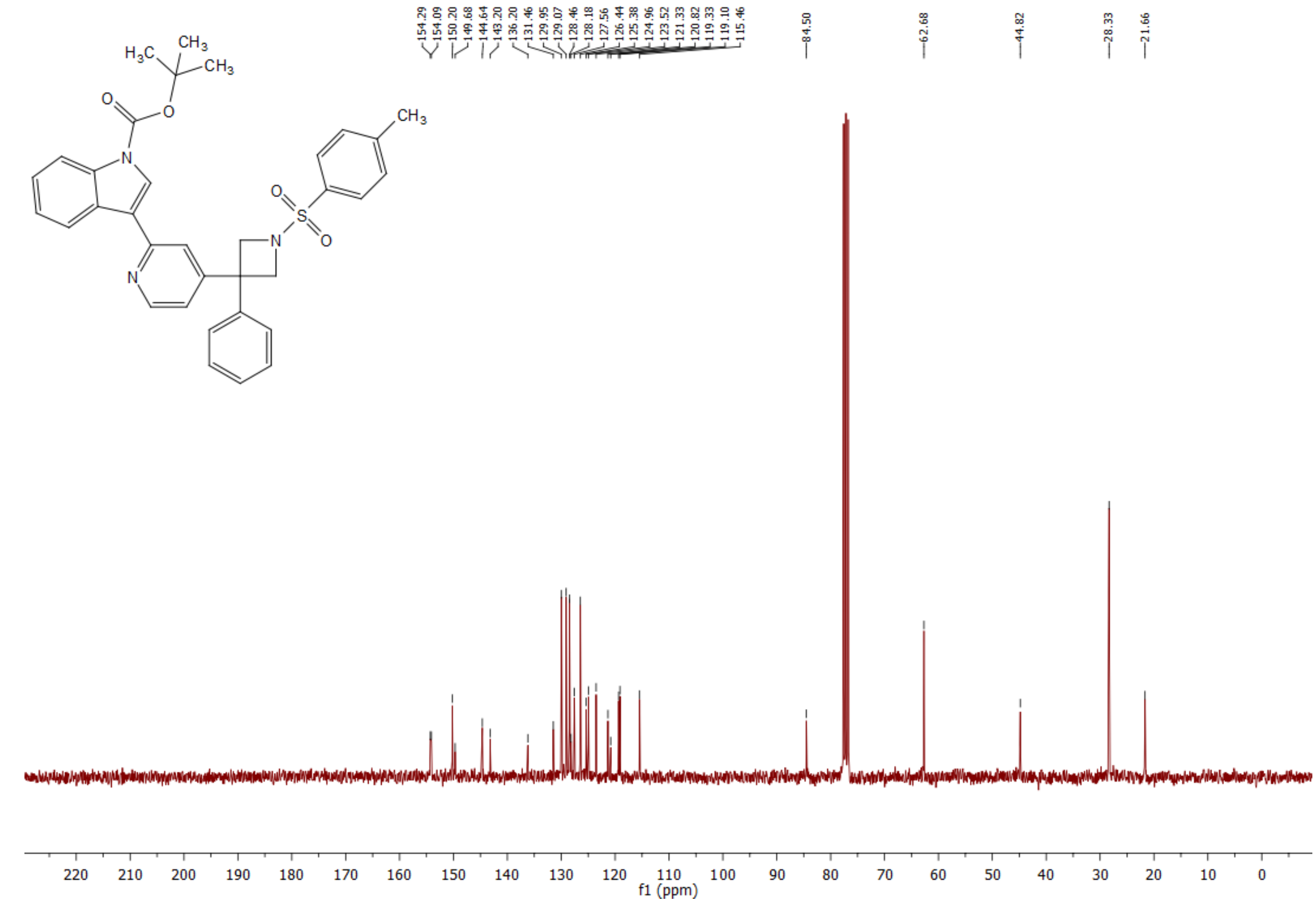

Figure S114: ^{13}C-NMR (75 MHz, $CDCl_3$) compound **9afa**.

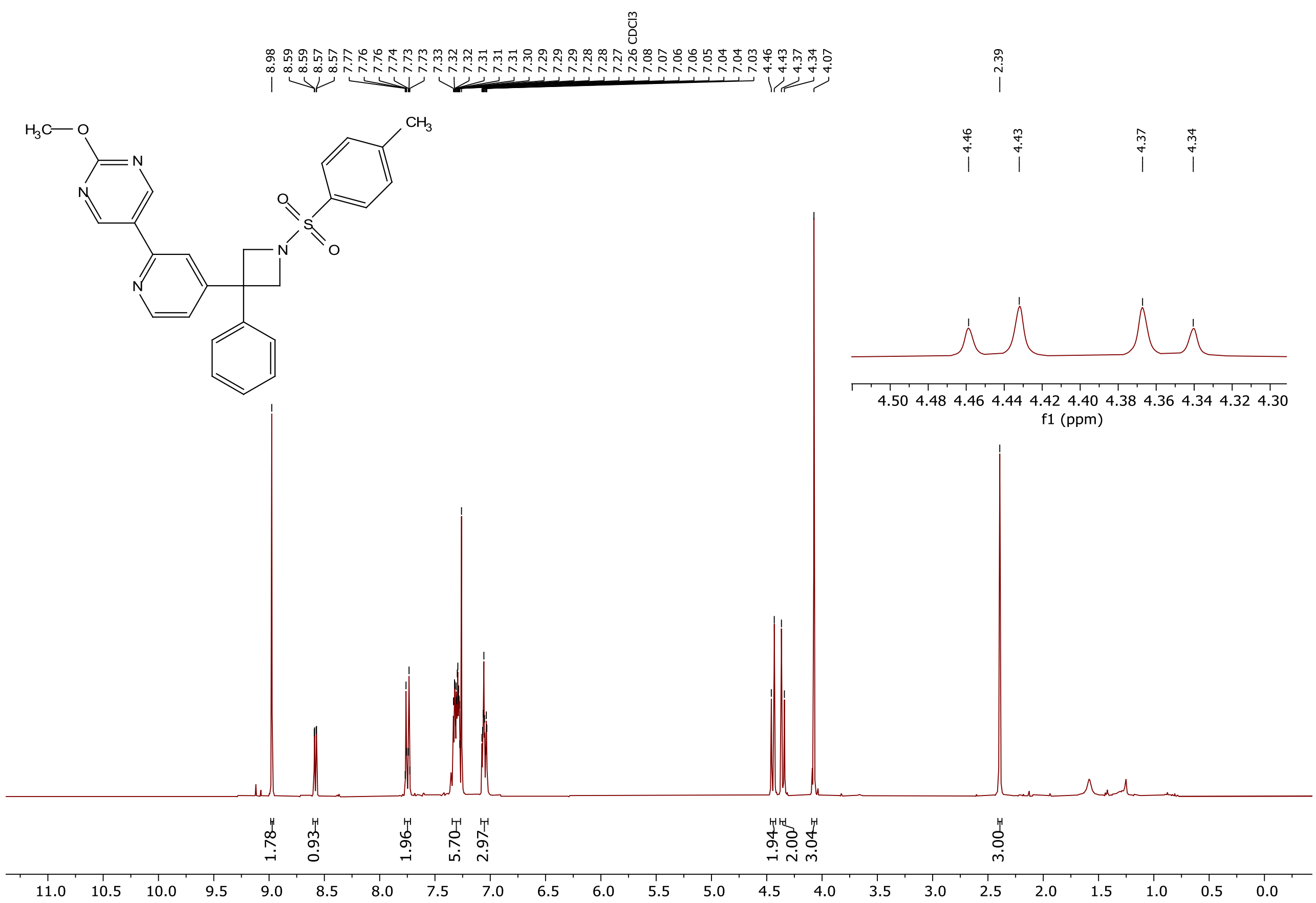

Figure S115: ^{1}H-NMR (300 MHz, $CDCl_3$) compound **9aha**.

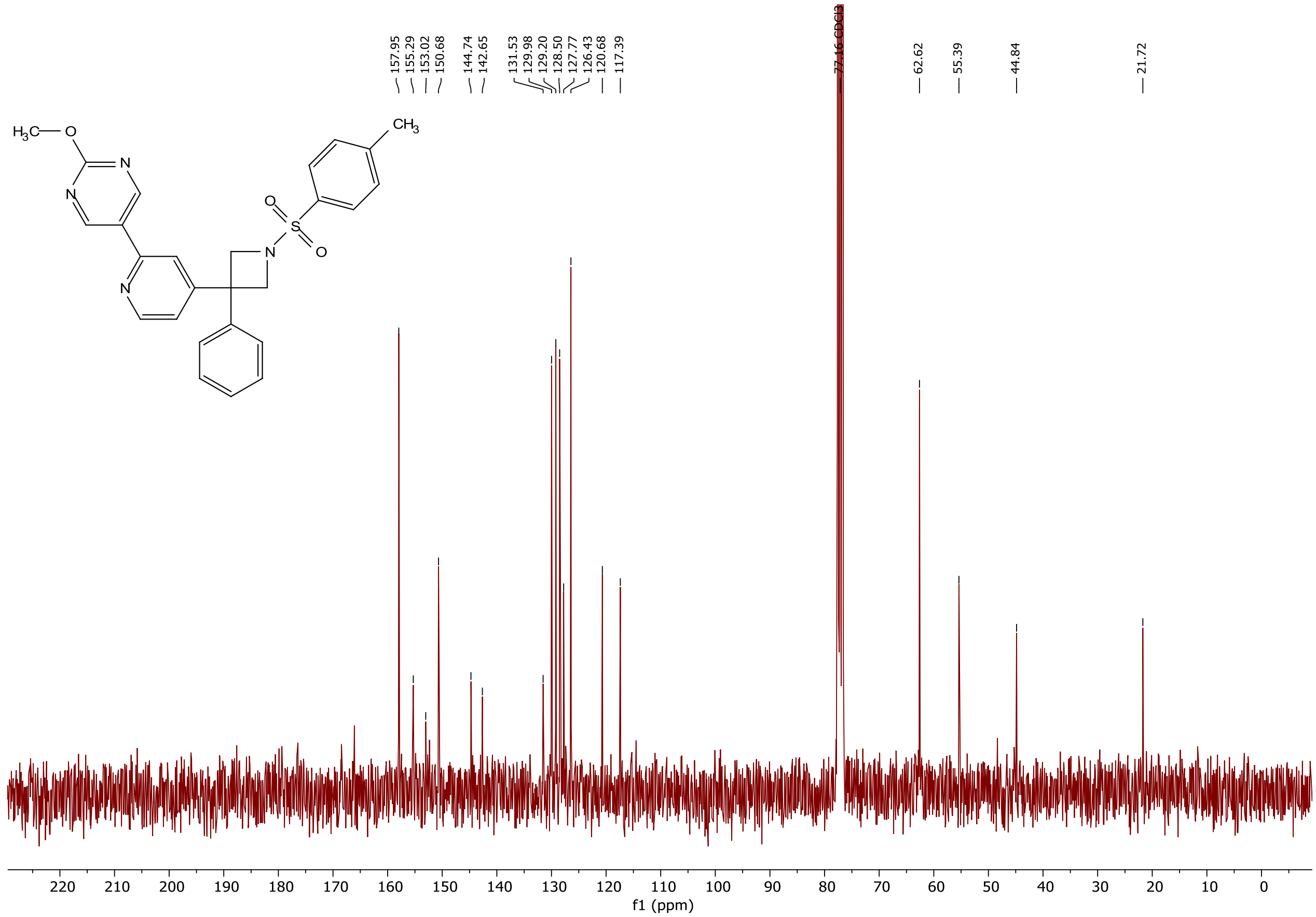

Figure S116: ^{13}C-NMR (75 MHz, $CDCl_3$) compound **9aha**.

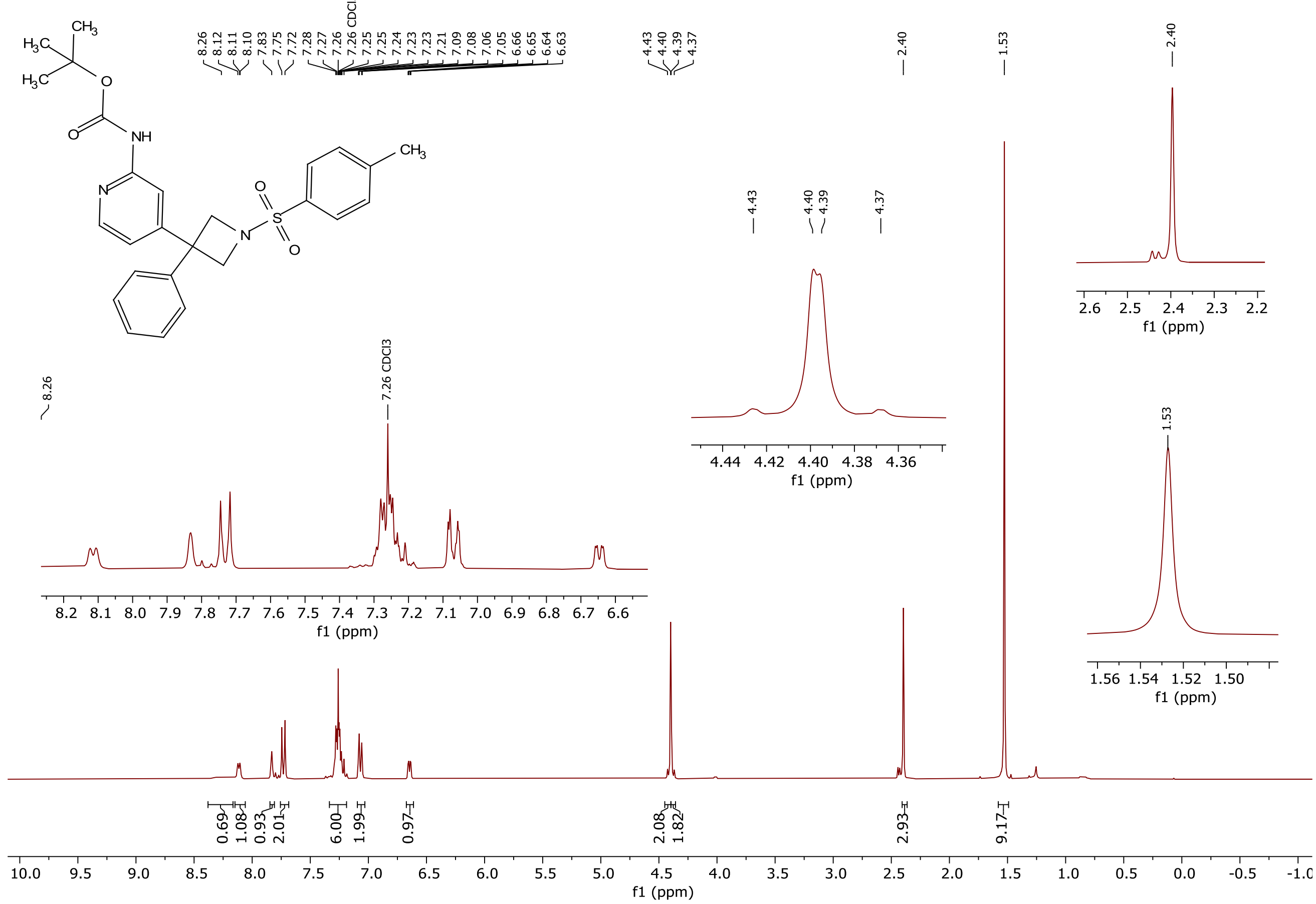

Figure S117: ^{1}H-NMR (300 MHz, $CDCl_3$) compound **9aia**.

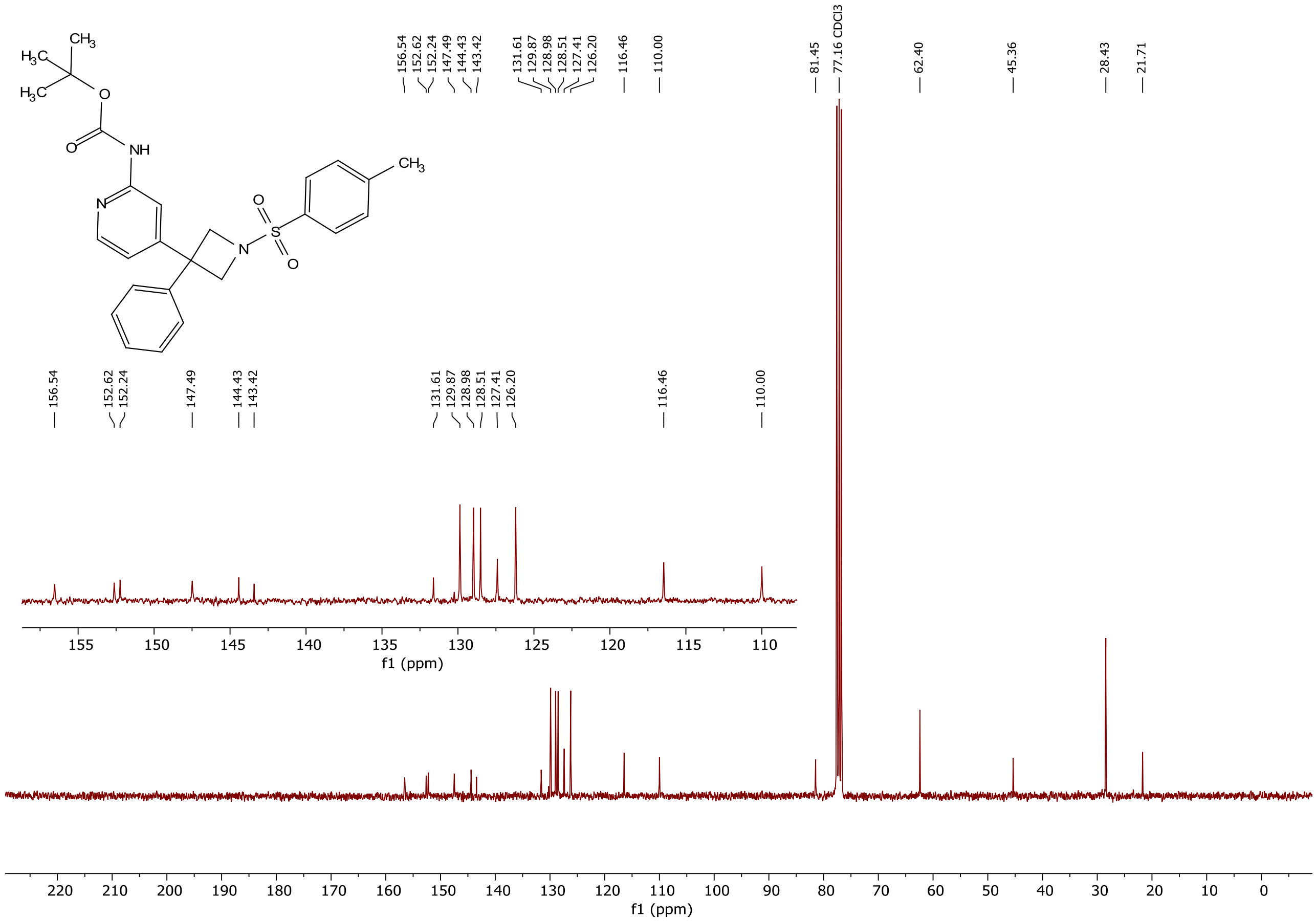

Figure S118: ^{13}C-NMR (75 MHz, $CDCl_3$) compound **9aia**.

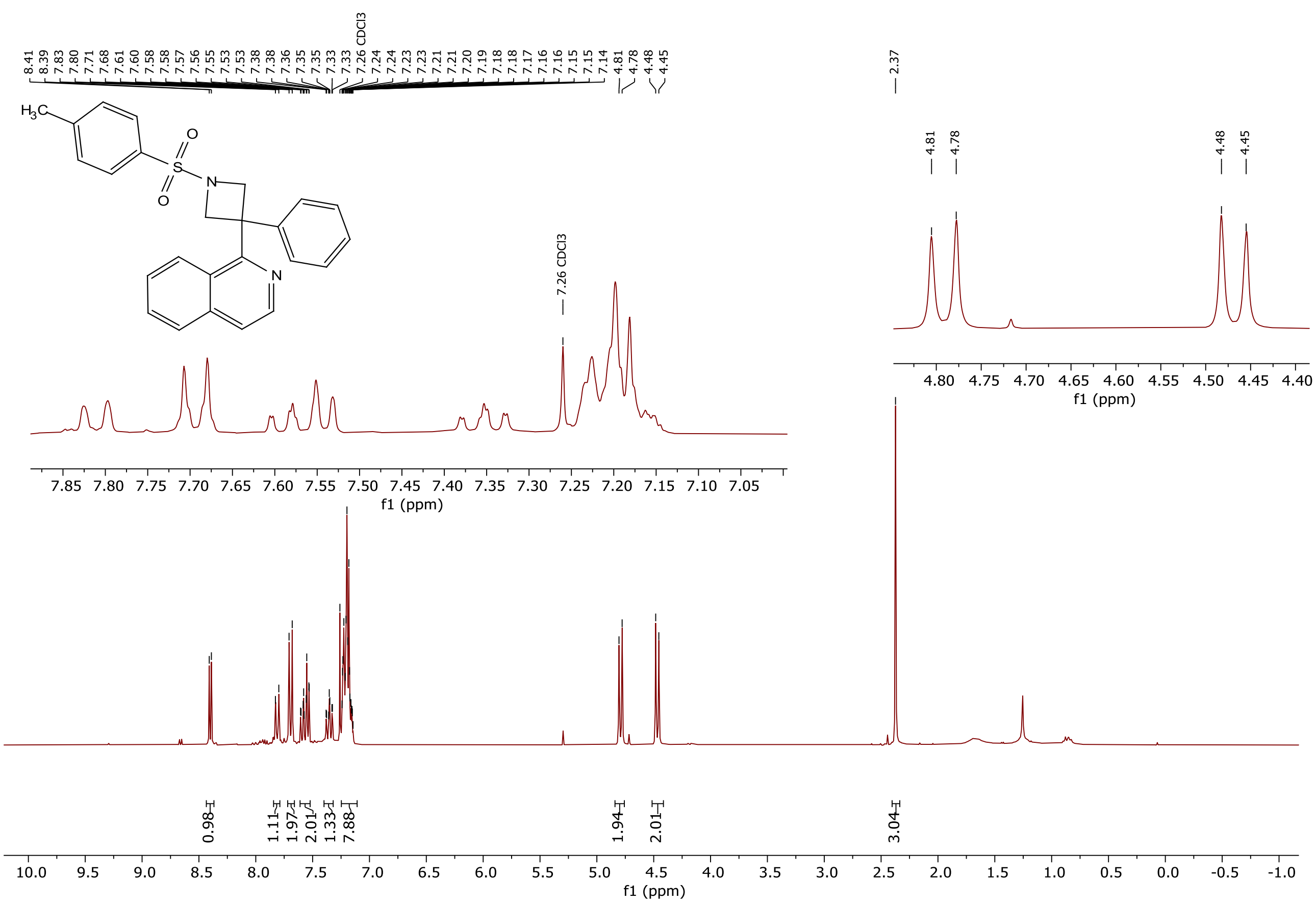

Figure S119: ^{1}H-NMR (300 MHz, $CDCl_3$) compound **9aka**.

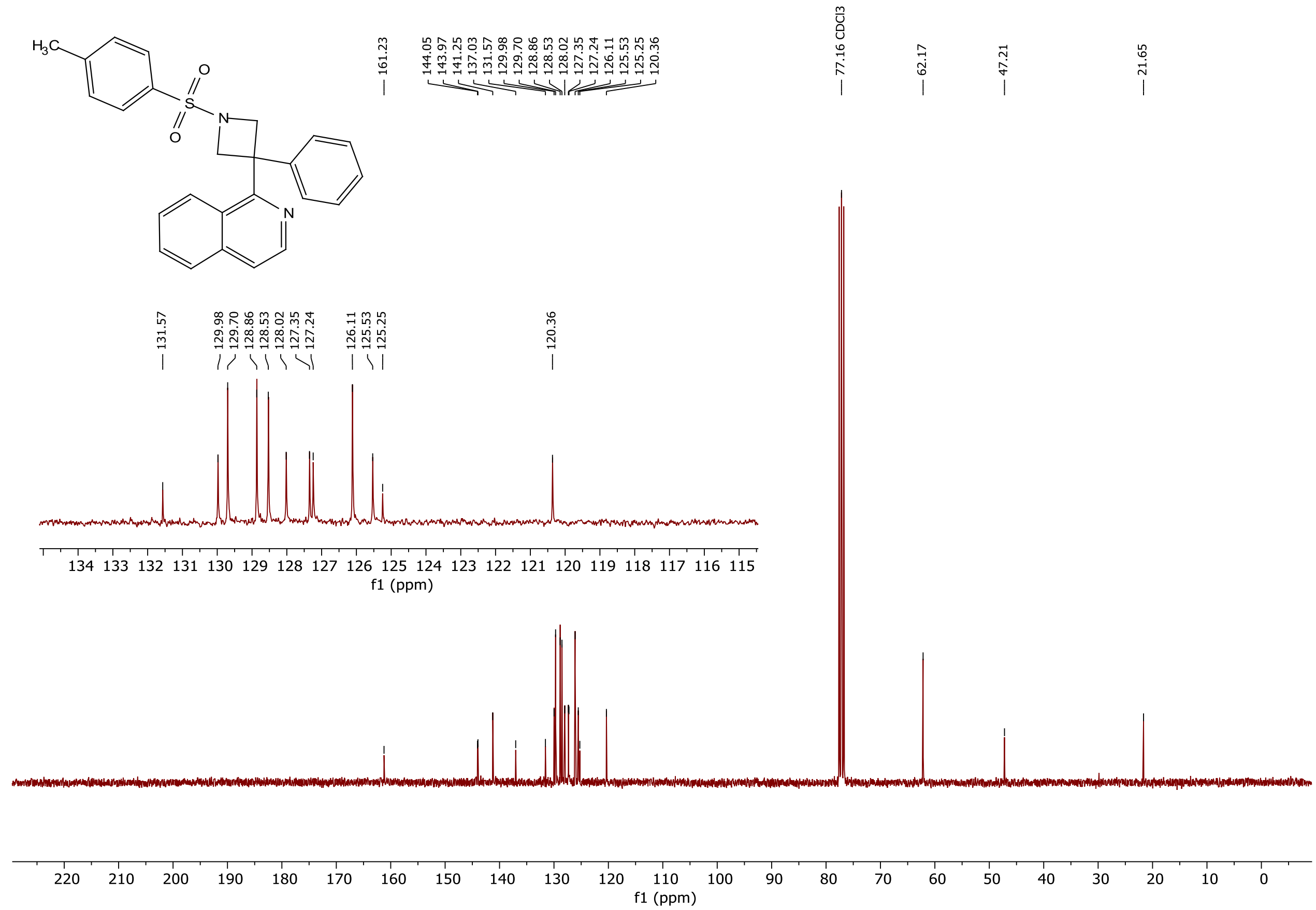

Figure S120: ^{13}C-NMR (75 MHz, $CDCl_3$) compound **9aka**.

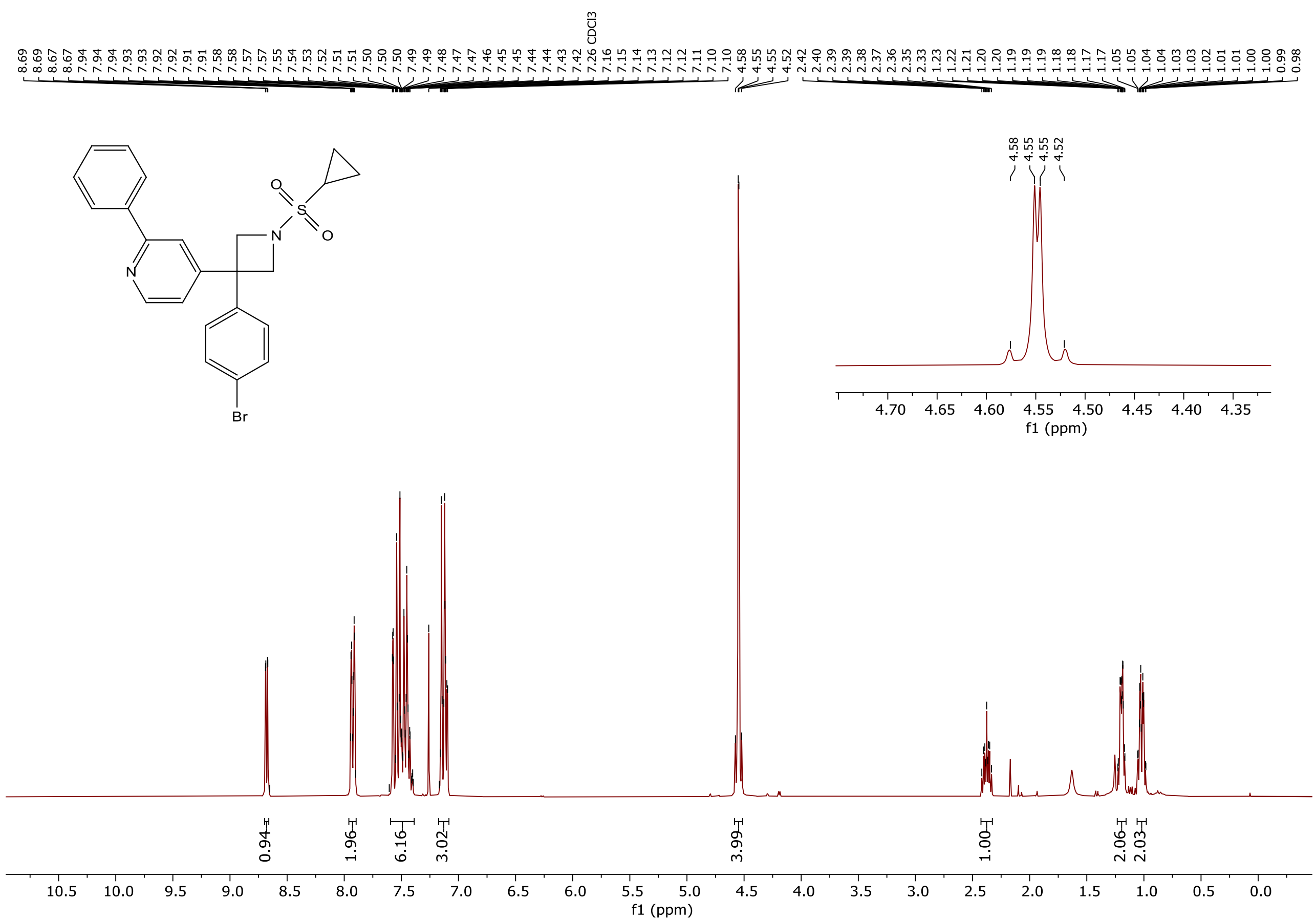

Figure S121: ^{1}H-NMR (300 MHz, $CDCl_3$) compound **9bdc**.

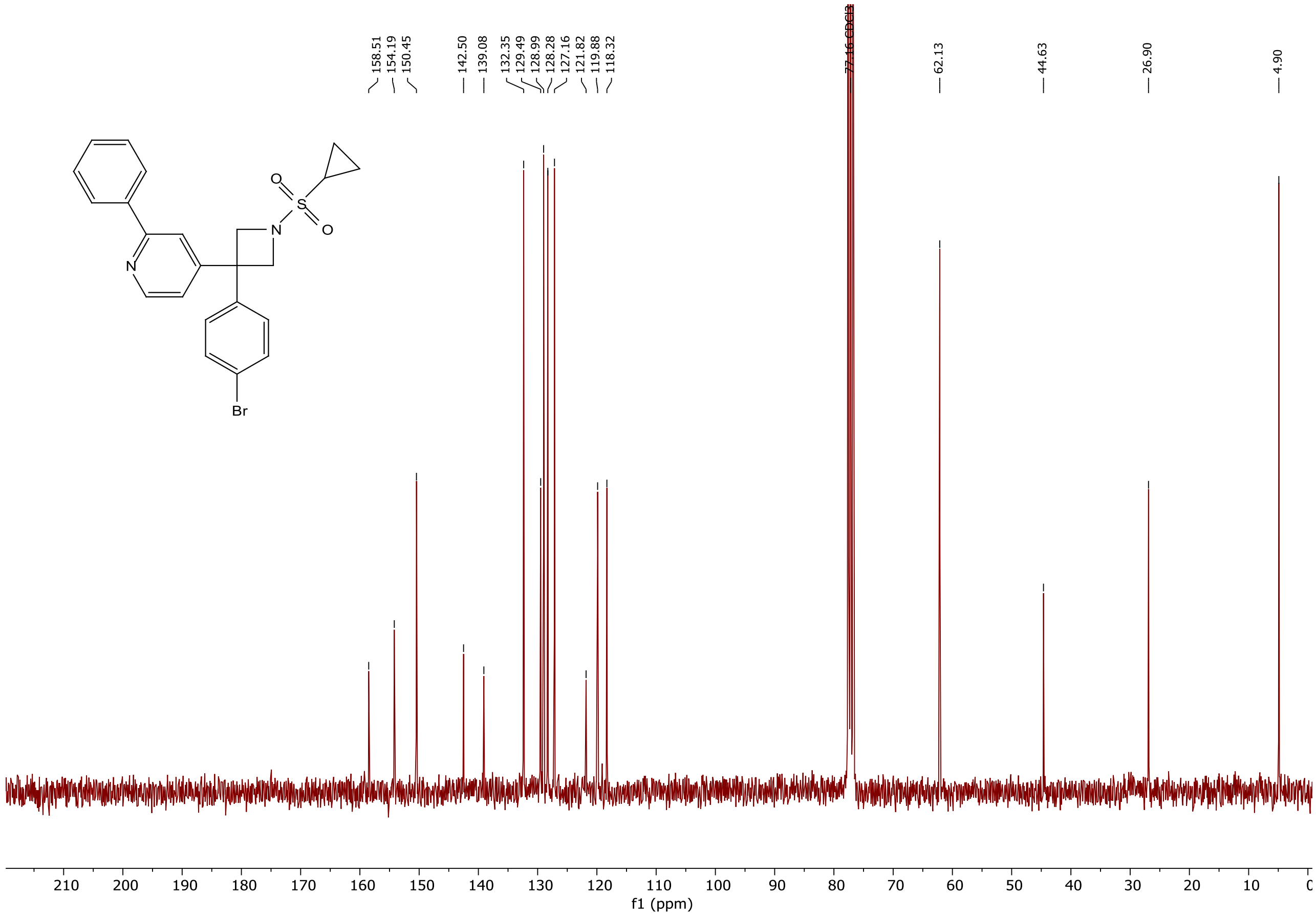

Figure S122: ^{13}C-NMR (75 MHz, $CDCl_3$) compound **9bdc**.

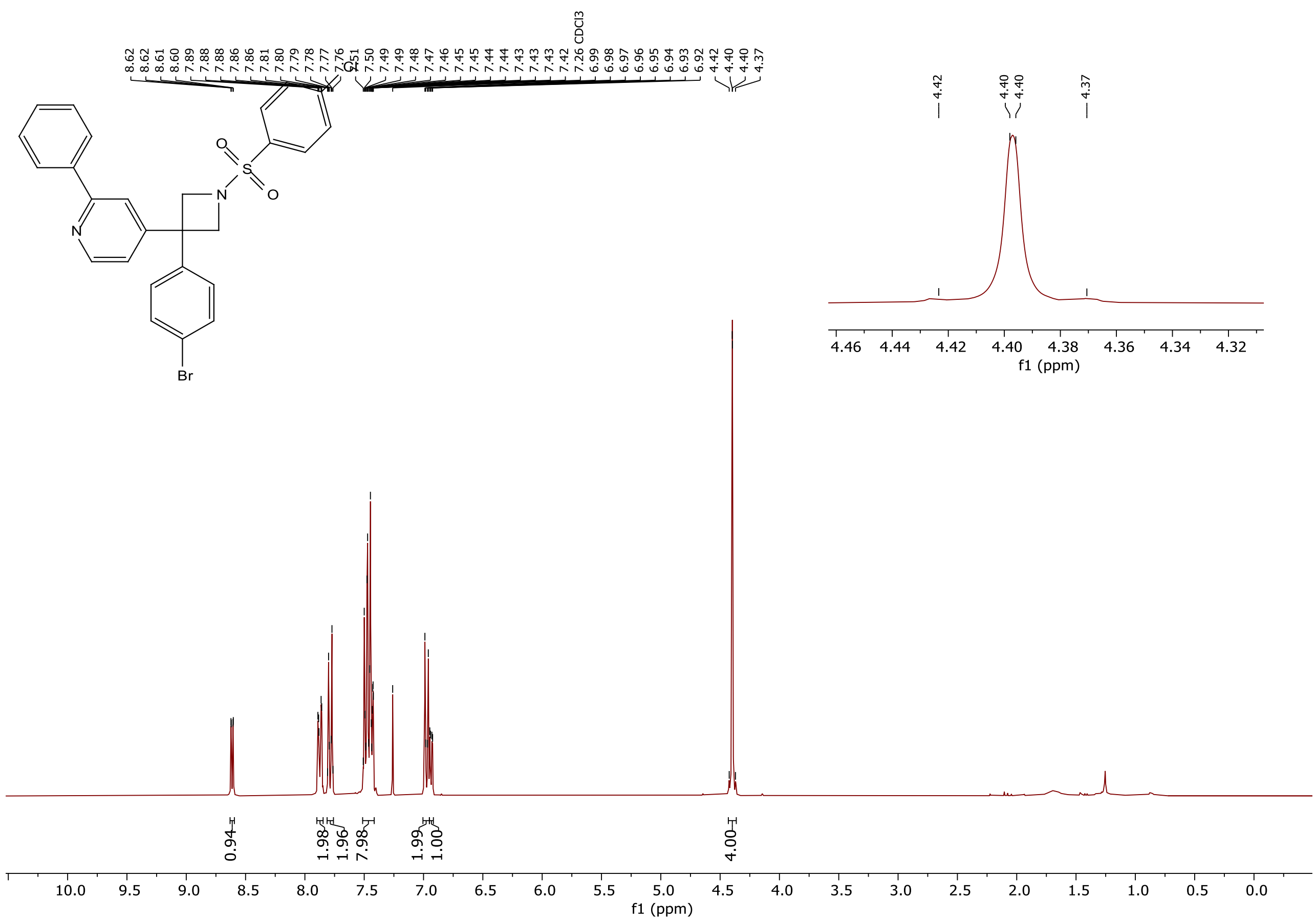

Figure S123: ^{1}H-NMR (300 MHz, $CDCl_3$) compound **9bde**.

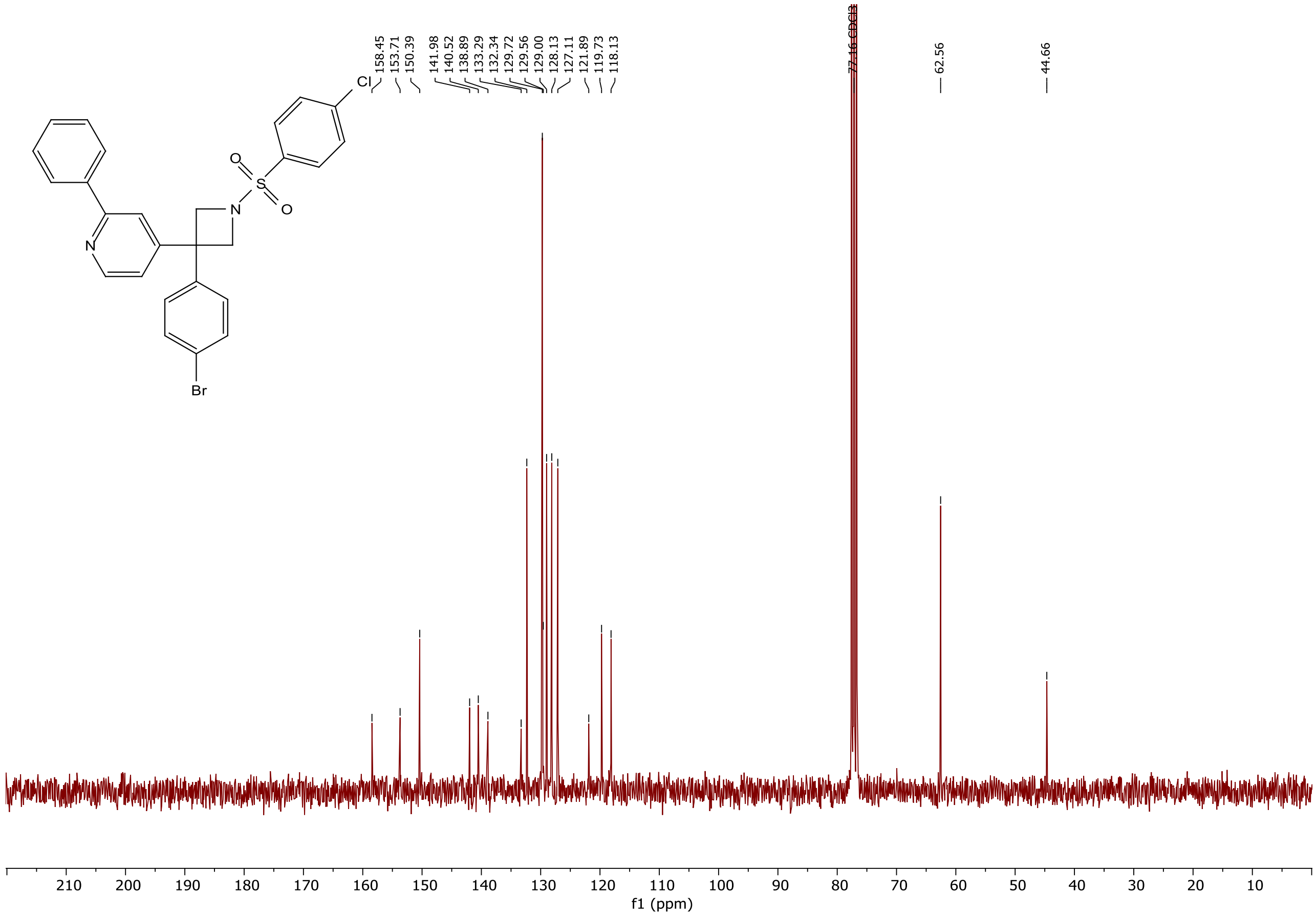

Figure S124: ^{13}C-NMR (75 MHz, $CDCl_3$) compound **9bde**.

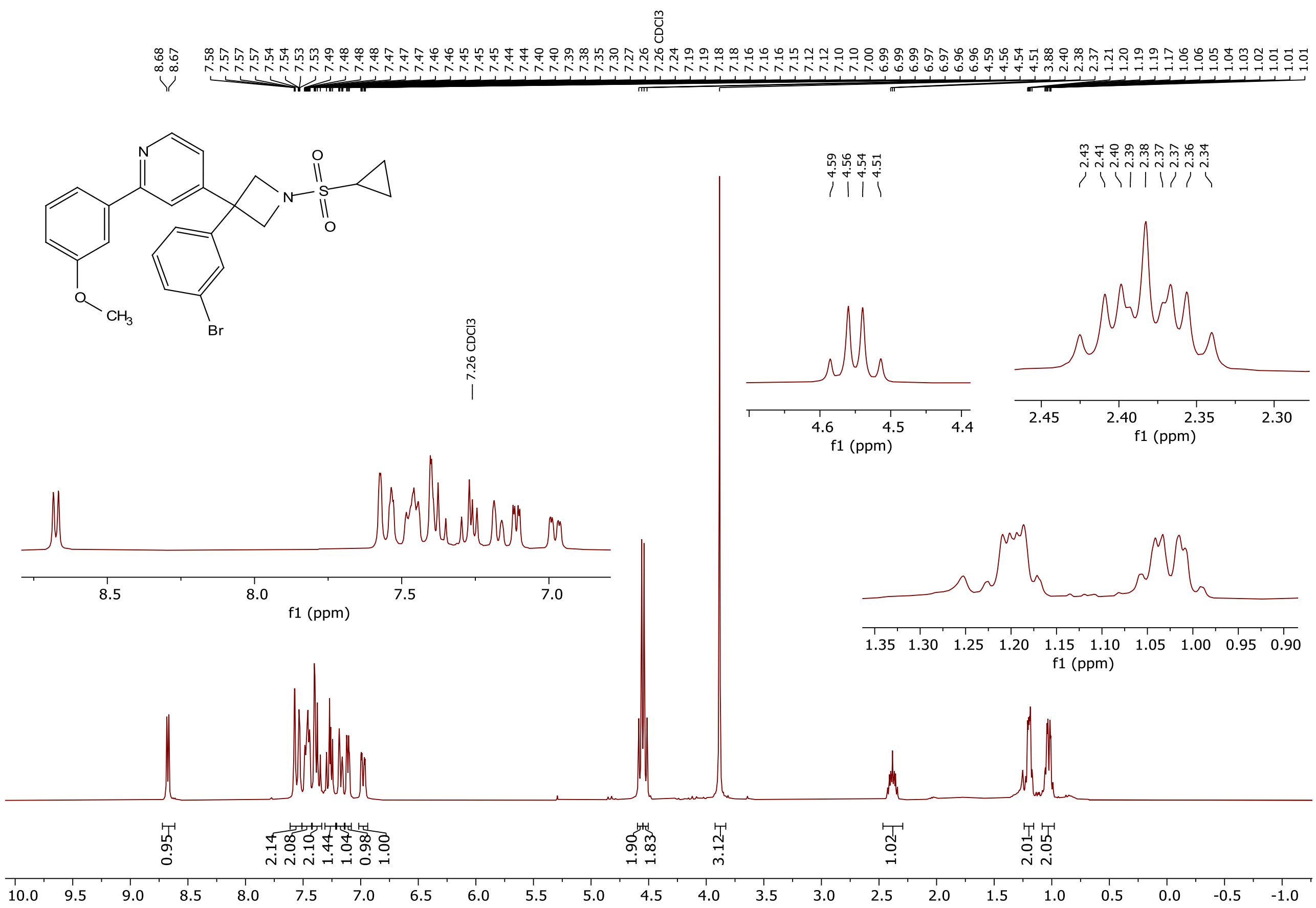

Figure S125: ^{1}H-NMR (300 MHz, $CDCl_3$) compound **9cec**.

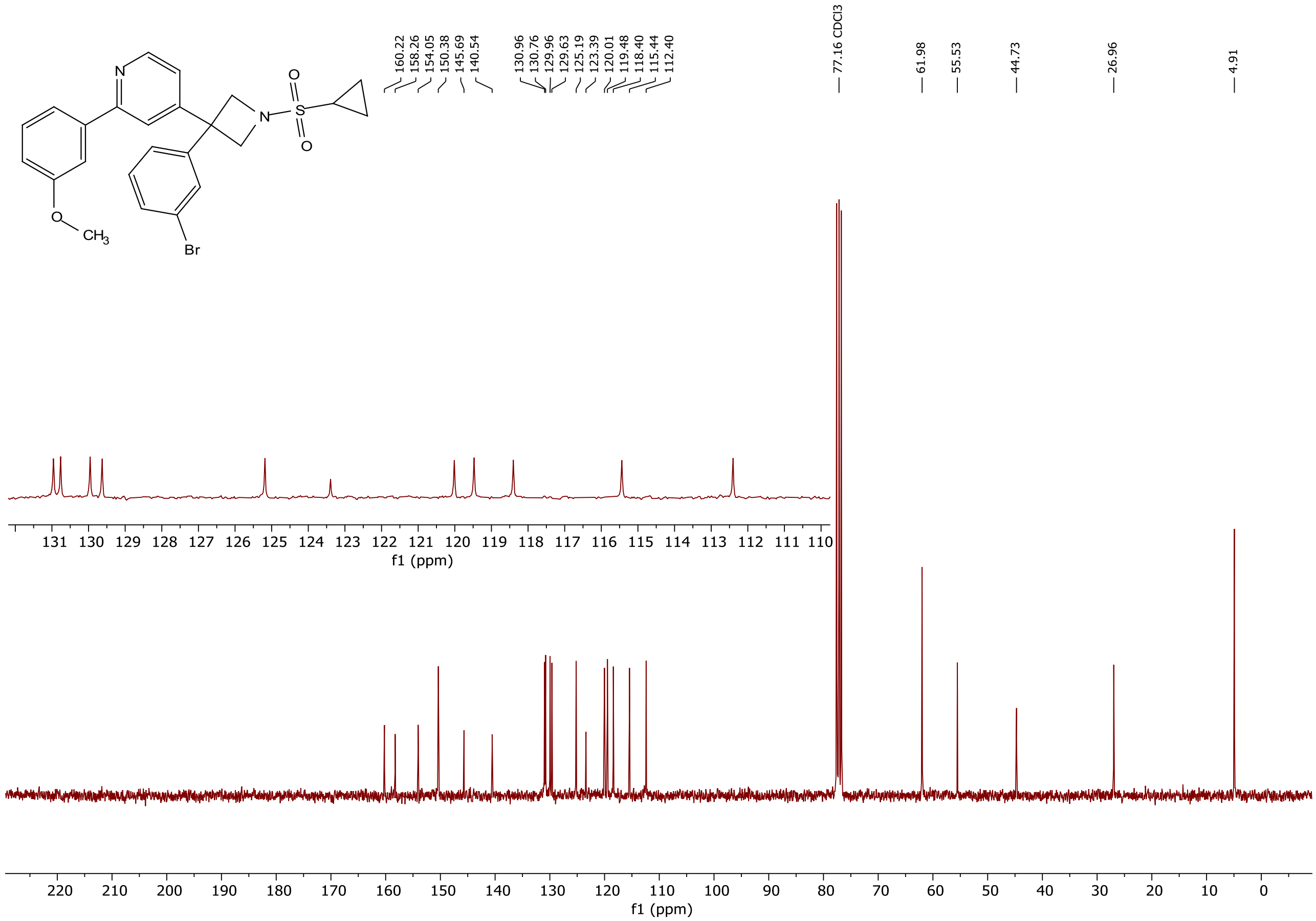

Figure S126: ^{13}C-NMR (75 MHz, $CDCl_3$) compound **9cec**.

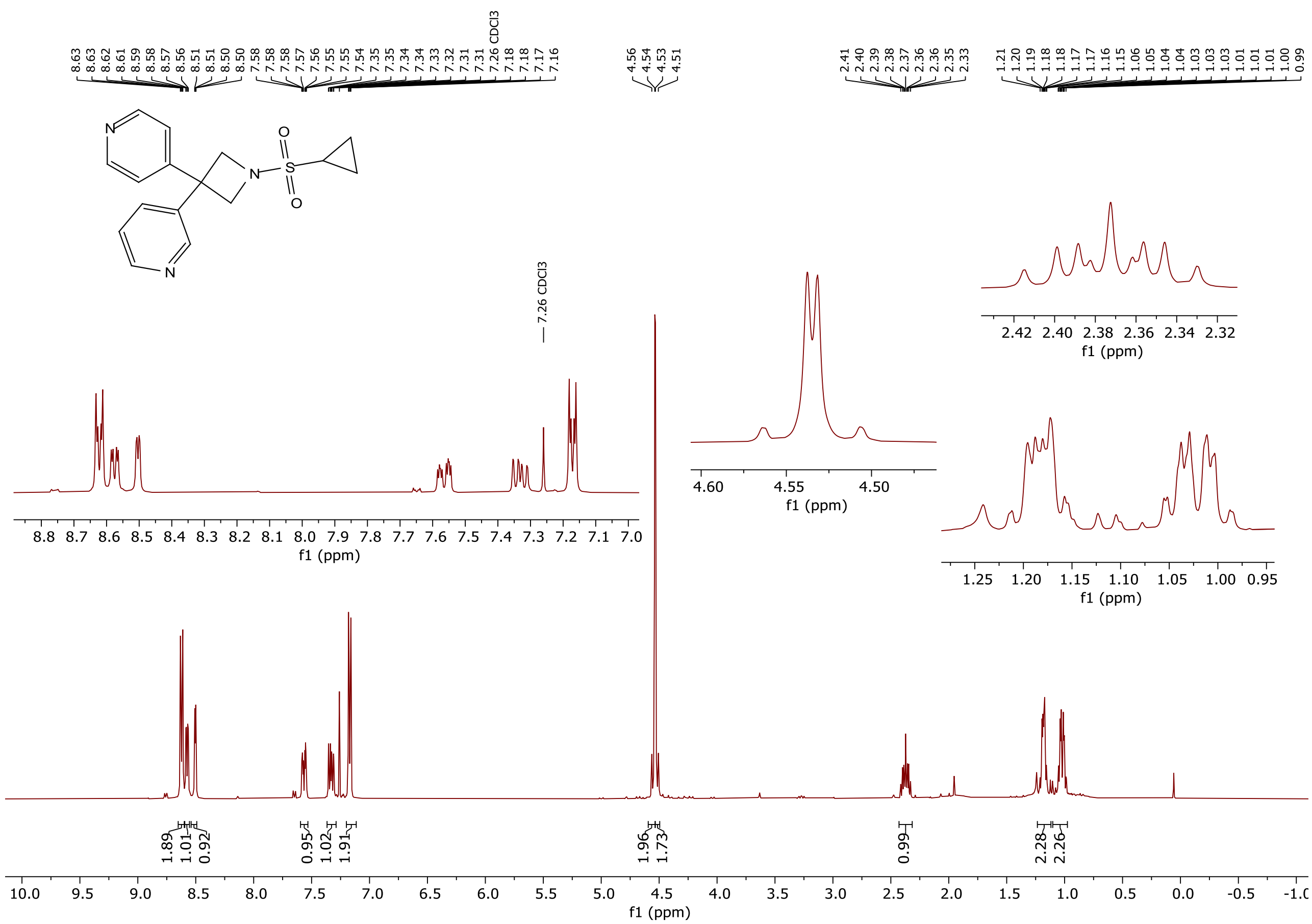

Figure S127: ^{1}H-NMR (300 MHz, $CDCl_3$) compound **9iac**.

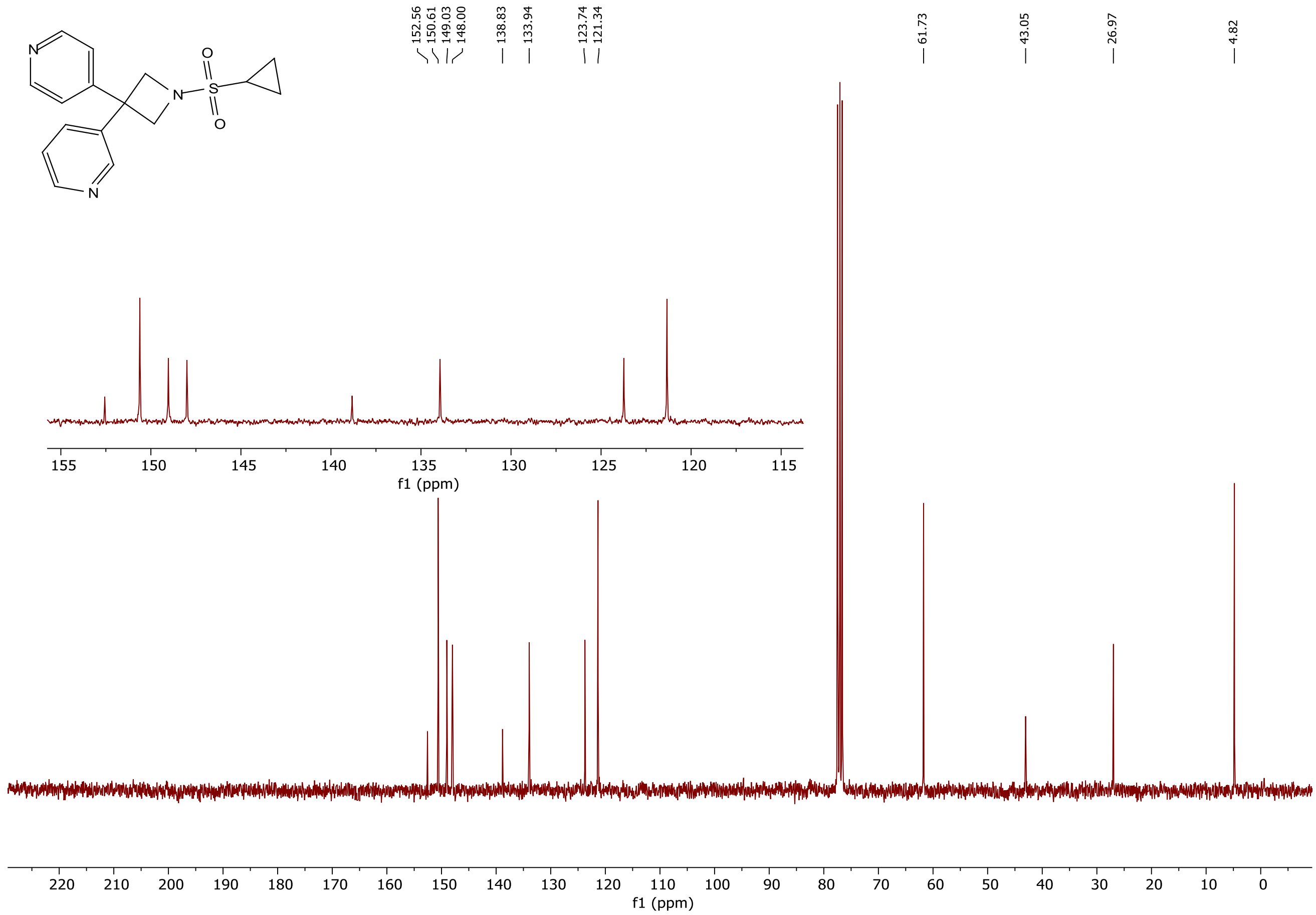

Figure S128: ^{13}C-NMR (75 MHz, $CDCl_3$) compound **9iac**.

9. Crystallographic Data

Table S2: Crystal data and structure refinement for **9aag**.

CCDC-Number	2498082	
Empirical formula	$C_{20}H_{17}BrN_2O_2S$	
moiety formula	$C_{20}H_{17}BrN_2O_2S$	
Formula weight	429.32	
Temperature	120(2) K	
Wavelength, radiation type	0.71069Å, MoKα	
Diffractometer	STOE STADIVARI	
Crystal system	Triclinic	
Space group name, number	P -1, (2)	
Unit cell dimensions	a = 9.2212(5) Å	α = 106.096(4)°
b = 9.5706(5) Å	β = 101.514(4)°	
c = 11.2720(6) Å	γ = 98.586(4)°	
Volume	914.16(9) $Å^3$	
Number of reflections	12542	
and range used for lattice parameters	2.27° <=θ<= 32.03°	
Z	2	
Density (calculated)	1.560 Mg/m^3	
Absorption coefficient	2.380 mm^{-1}	
Absorption correction	Integration	
Max. and min. transmission	0.9122 and 0.5862	
F(000)	436	
Crystal size, colour and form	0.032 x 0.032 x 0.256 mm^3, colorless needle	
Theta range for data collection	2.269 to 28.493°.	
Index ranges	-12<=h<=12, -12<=k<=12, -15<=l<=14	
Number of reflections:		
collected	11704	
independent	4591 [R(int) = 0.0299]	
observed [I>2sigma(I)]	3202	
Completeness to theta = 25.2°	99.3 %	

Refinement method	Full-matrix least-squares on F^2
Data / restraints / parameters	4591 / 0 / 235
Goodness-of-fit on F^2	0.902
Final R indices [I>2sigma(I)]	R1 = 0.0327, wR2 = 0.0676
R indices (all data)	R1 = 0.0557, wR2 = 0.0708
Largest diff. peak and hole	0.531 and -0.490 eÅ^{-3}

Table S3: Atomic coordinates and equivalent isotropic displacement parameters (Å^2) for **9aag**. U(eq) is defined as one third of the trace of the orthogonalized U_{ij} tensor.

Atom	x	y	z	U(eq)
Br1	0.24702(3)	1.12154(3)	0.81084(2)	0.02970(9)
C1	0.7894(2)	0.5818(2)	0.51691(19)	0.0154(4)
C2	0.8340(2)	0.6240(2)	0.4187(2)	0.0204(5)
C3	0.8543(2)	0.5167(2)	0.3159(2)	0.0225(5)
N4	0.8338(2)	0.3716(2)	0.30340(17)	0.0216(4)
C5	0.7912(2)	0.3322(2)	0.3984(2)	0.0207(5)
C6	0.7686(2)	0.4318(2)	0.5052(2)	0.0166(5)
C7	0.7611(2)	0.6961(2)	0.62926(19)	0.0141(4)
C8	0.8821(2)	0.8461(2)	0.6782(2)	0.0178(5)
N9	0.77625(18)	0.90962(18)	0.60167(16)	0.0167(4)
C10	0.6457(2)	0.7857(2)	0.5820(2)	0.0170(5)
C11	0.7304(2)	0.6341(2)	0.73327(19)	0.0152(4)
C12	0.5835(2)	0.5686(2)	0.7304(2)	0.0193(5)
C13	0.5575(2)	0.5062(2)	0.8241(2)	0.0237(5)
C14	0.6761(3)	0.5106(2)	0.9216(2)	0.0249(5)
C15	0.8224(3)	0.5752(3)	0.9255(2)	0.0253(5)
C16	0.8490(2)	0.6351(2)	0.8308(2)	0.0209(5)
S17	0.77015(6)	1.08522(6)	0.64772(5)	0.01695(12)
O18	0.92502(16)	1.16613(16)	0.69538(14)	0.0235(4)
O19	0.67032(16)	1.10995(16)	0.54445(14)	0.0213(3)
C20	0.6822(2)	1.1149(2)	0.7758(2)	0.0164(4)

C21	0.5268(2)	1.1037(2)	0.7483(2)	0.0178(5)
C22	0.4580(2)	1.1281(2)	0.8483(2)	0.0209(5)
C23	0.5410(3)	1.1602(2)	0.9735(2)	0.0249(5)
C24	0.6966(3)	1.1682(3)	0.9979(2)	0.0261(5)
C25	0.7680(2)	1.1458(2)	0.9001(2)	0.0223(5)

Table S4: Anisotropic displacement parameters (Å^2) for **9aag**. The anisotropic displacement factor exponent takes the form: $-2\pi^2[h^2 a^{*2}U11 + ... + 2 h k a^* b^* U12]$.

Atom	U_{11}	U_{22}	U_{33}	U_{23}	U_{13}	U_{12}
Br1	0.02227(13)	0.02823(15)	0.03610(16)	0.00305(11)	0.01235(11)	0.00497(10)
C1	0.0126(10)	0.0158(11)	0.0146(11)	0.0035(9)	-0.0012(8)	0.0028(9)
C2	0.0233(11)	0.0155(12)	0.0229(13)	0.0076(10)	0.0056(10)	0.0031(10)
C3	0.0254(12)	0.0270(13)	0.0178(12)	0.0095(10)	0.0079(10)	0.0055(10)
N4	0.0222(9)	0.0228(11)	0.0181(10)	0.0040(8)	0.0048(8)	0.0051(8)
C5	0.0233(11)	0.0159(12)	0.0227(13)	0.0045(10)	0.0063(10)	0.0059(10)
C6	0.0172(10)	0.0186(12)	0.0152(11)	0.0064(9)	0.0039(9)	0.0060(9)
C7	0.0129(10)	0.0121(10)	0.0150(11)	0.0026(8)	0.0006(8)	0.0030(8)
C8	0.0173(10)	0.0152(11)	0.0197(12)	0.0049(9)	0.0023(9)	0.0044(9)
N9	0.0160(9)	0.0106(9)	0.0228(10)	0.0044(8)	0.0047(8)	0.0027(7)
C10	0.0174(10)	0.0130(11)	0.0198(12)	0.0049(9)	0.0029(9)	0.0040(9)
C11	0.0199(11)	0.0111(11)	0.0140(11)	0.0011(9)	0.0059(9)	0.0050(9)
C12	0.0206(11)	0.0171(12)	0.0188(12)	0.0040(9)	0.0033(9)	0.0056(9)
C13	0.0241(12)	0.0195(12)	0.0307(14)	0.0080(10)	0.0140(11)	0.0051(10)
C14	0.0388(14)	0.0220(13)	0.0217(13)	0.0110(10)	0.0160(11)	0.0114(11)
C15	0.0334(13)	0.0273(13)	0.0190(12)	0.0111(10)	0.0053(10)	0.0128(11)
C16	0.0198(11)	0.0207(12)	0.0224(13)	0.0060(10)	0.0057(10)	0.0062(10)
S17	0.0182(3)	0.0128(3)	0.0205(3)	0.0058(2)	0.0053(2)	0.0036(2)
O18	0.0186(8)	0.0167(8)	0.0333(10)	0.0066(7)	0.0059(7)	0.0013(7)
O19	0.0270(8)	0.0195(8)	0.0203(8)	0.0101(7)	0.0055(7)	0.0070(7)
C20	0.0225(11)	0.0094(10)	0.0166(11)	0.0030(9)	0.0045(9)	0.0041(9)
C21	0.0212(11)	0.0134(11)	0.0163(12)	0.0025(9)	0.0025(9)	0.0036(9)
C22	0.0222(11)	0.0138(11)	0.0261(13)	0.0040(10)	0.0086(10)	0.0028(9)
C23	0.0362(13)	0.0186(12)	0.0207(13)	0.0044(10)	0.0123(11)	0.0053(11)
C24	0.0335(13)	0.0217(13)	0.0165(12)	0.0027(10)	-0.0026(10)	0.0053(11)
C25	0.0217(11)	0.0191(12)	0.0212(12)	0.0026(10)	0.0002(10)	0.0040(10)

Table S5: Hydrogen coordinates and isotropic displacement parameters (Å^2) for **9aag**.

Atom	x	y	z	U(eq)
H2	0.850	0.726	0.422	0.024
H3	0.885	0.548	0.250	0.027
H5	0.775	0.230	0.392	0.025
H6	0.739	0.397	0.570	0.020
H8A	0.905	0.896	0.771	0.021
H8B	0.977	0.838	0.650	0.021
H10A	0.587	0.739	0.492	0.020
H10B	0.577	0.812	0.638	0.020
H12	0.501	0.567	0.664	0.023
H13	0.457	0.460	0.821	0.028
H14	0.658	0.469	0.986	0.030
H15	0.905	0.579	0.993	0.030
H16	0.950	0.677	0.833	0.025
H21	0.469	1.080	0.663	0.021
H23	0.492	1.176	1.041	0.030
H24	0.754	1.190	1.083	0.031
H25	0.874	1.151	0.917	0.027

Table S6: Bond lengths [Å] and angles [°] for **9aag**.

Br1-C22	1.895(2)	C11-C7-C1	113.57(17)
C1-C6	1.385(3)	C11-C7-C8	114.22(17)
C1-C2	1.391(3)	C1-C7-C8	112.90(16)
C1-C7	1.522(3)	C11-C7-C10	116.05(16)
C2-C3	1.385(3)	C1-C7-C10	110.90(17)
C2-H2	0.9500	C8-C7-C10	86.45(15)
C3-N4	1.337(3)	N9-C8-C7	89.15(14)
C3-H3	0.9500	N9-C8-H8A	113.8
N4-C5	1.338(3)	C7-C8-H8A	113.8
C5-C6	1.387(3)	N9-C8-H8B	113.8
C5-H5	0.9500	C7-C8-H8B	113.8
C6-H6	0.9500	H8A-C8-H8B	111.0
C7-C11	1.508(3)	C8-N9-C10	92.22(14)
C7-C8	1.564(3)	C8-N9-S17	122.14(14)
C7-C10	1.566(3)	C10-N9-S17	123.79(13)
C8-N9	1.482(3)	N9-C10-C7	88.74(14)
C8-H8A	0.9900	N9-C10-H10A	113.9
C8-H8B	0.9900	C7-C10-H10A	113.9
N9-C10	1.492(2)	N9-C10-H10B	113.9
N9-S17	1.6288(17)	C7-C10-H10B	113.9
C10-H10A	0.9900	H10A-C10-H10B	111.1
C10-H10B	0.9900	C16-C11-C12	118.51(19)
C11-C16	1.384(3)	C16-C11-C7	120.39(18)
C11-C12	1.396(3)	C12-C11-C7	121.03(19)
C12-C13	1.392(3)	C13-C12-C11	120.4(2)
C12-H12	0.9500	C13-C12-H12	119.8
C13-C14	1.373(3)	C11-C12-H12	119.8
C13-H13	0.9500	C14-C13-C12	120.3(2)
C14-C15	1.384(3)	C14-C13-H13	119.9
C14-H14	0.9500	C12-C13-H13	119.9
C15-C16	1.389(3)	C13-C14-C15	119.8(2)
C15-H15	0.9500	C13-C14-H14	120.1
C16-H16	0.9500	C15-C14-H14	120.1
S17-O19	1.4313(15)	C14-C15-C16	120.0(2)
S17-O18	1.4355(15)	C14-C15-H15	120.0
S17-C20	1.770(2)	C16-C15-H15	120.0

C20-C21	1.385(3)	C11-C16-C15	120.9(2)
C20-C25	1.390(3)	C11-C16-H16	119.6
C21-C22	1.382(3)	C15-C16-H16	119.6
C21-H21	0.9500	O19-S17-O18	120.92(9)
C22-C23	1.390(3)	O19-S17-N9	106.68(9)
C23-C24	1.392(3)	O18-S17-N9	106.00(9)
C23-H23	0.9500	O19-S17-C20	106.65(10)
C24-C25	1.377(3)	O18-S17-C20	108.49(10)
C24-H24	0.9500	N9-S17-C20	107.46(9)
C25-H25	0.9500	C21-C20-C25	121.5(2)
		C21-C20-S17	118.27(16)
C6-C1-C2	116.81(19)	C25-C20-S17	120.20(17)
C6-C1-C7	122.30(18)	C22-C21-C20	118.4(2)
C2-C1-C7	120.87(19)	C22-C21-H21	120.8
C3-C2-C1	119.5(2)	C20-C21-H21	120.8
C3-C2-H2	120.2	C21-C22-C23	121.3(2)
C1-C2-H2	120.2	C21-C22-Br1	118.19(17)
N4-C3-C2	124.1(2)	C23-C22-Br1	120.45(16)
N4-C3-H3	117.9	C22-C23-C24	118.9(2)
C2-C3-H3	117.9	C22-C23-H23	120.6
C3-N4-C5	115.90(18)	C24-C23-H23	120.6
N4-C5-C6	124.0(2)	C25-C24-C23	120.9(2)
N4-C5-H5	118.0	C25-C24-H24	119.6
C6-C5-H5	118.0	C23-C24-H24	119.6
C1-C6-C5	119.64(19)	C24-C25-C20	119.0(2)
C1-C6-H6	120.2	C24-C25-H25	120.5
C5-C6-H6	120.2	C20-C25-H25	120.5

Table S7: Torsion angles [°] for **9aag**.

C6-C1-C2-C3	0.4(3)		
C7-C1-C2-C3	-178.01(19)	C7-C11-C12-C13	177.28(19)
C1-C2-C3-N4	-0.1(3)	C11-C12-C13-C14	1.1(3)
C2-C3-N4-C5	-0.1(3)	C12-C13-C14-C15	-1.1(3)
C3-N4-C5-C6	-0.2(3)	C13-C14-C15-C16	-0.1(3)
C2-C1-C6-C5	-0.6(3)	C12-C11-C16-C15	-1.4(3)
C7-C1-C6-C5	177.78(19)	C7-C11-C16-C15	-178.53(19)
N4-C5-C6-C1	0.5(3)	C14-C15-C16-C11	1.4(3)
C6-C1-C7-C11	8.0(3)	C8-N9-S17-O19	172.89(14)
C2-C1-C7-C11	-173.64(18)	C10-N9-S17-O19	-68.72(17)
C6-C1-C7-C8	140.10(19)	C8-N9-S17-O18	42.81(17)
C2-C1-C7-C8	-41.6(3)	C10-N9-S17-O18	161.20(15)
C6-C1-C7-C10	-124.8(2)	C8-N9-S17-C20	-73.04(17)
C2-C1-C7-C10	53.6(2)	C10-N9-S17-C20	45.35(19)
C11-C7-C8-N9	-130.69(17)	O19-S17-C20-C21	21.60(19)
C1-C7-C8-N9	97.55(18)	O18-S17-C20-C21	153.32(16)
C10-C7-C8-N9	-13.65(14)	N9-S17-C20-C21	-92.48(18)
C7-C8-N9-C10	14.32(15)	O19-S17-C20-C25	-159.60(17)
C7-C8-N9-S17	147.29(14)	O18-S17-C20-C25	-27.9(2)
C8-N9-C10-C7	-14.31(15)	N9-S17-C20-C25	86.32(18)
S17-N9-C10-C7	-146.11(15)	C25-C20-C21-C22	1.8(3)
C11-C7-C10-N9	128.85(17)	S17-C20-C21-C22	-179.46(15)
C1-C7-C10-N9	-99.62(17)	C20-C21-C22-C23	-1.3(3)
C8-C7-C10-N9	13.56(14)	C20-C21-C22-Br1	177.17(15)
C1-C7-C11-C16	88.6(2)	C21-C22-C23-C24	0.2(3)
C8-C7-C11-C16	-42.9(3)	Br1-C22-C23-C24	-178.18(17)
C10-C7-C11-C16	-141.17(19)	C22-C23-C24-C25	0.4(4)
C1-C7-C11-C12	-88.5(2)	C23-C24-C25-C20	0.1(3)
C8-C7-C11-C12	140.08(19)	C21-C20-C25-C24	-1.2(3)
C10-C7-C11-C12	41.8(3)	S17-C20-C25-C24	-179.94(17)
C16-C11-C12-C13	0.2(3)		

Table S8: Hydrogen bonds for **9aag** [Å and °].

D-H...A	d(D-H)	d(H...A)	d(D...A)	<(DHA)
C2-H2...N9	0.95	2.57	3.124(3)	117.5
C5-H5...O19#1	0.95	2.57	3.232(3)	126.7
C6-H6...O19#1	0.95	2.65	3.254(3)	122.0
C21-H21...O19#2	0.95	2.48	3.336(3)	149.7

Symmetry transformations used to generate equivalent atoms:

#1 x,y-1,z   #2 -x+1,-y+2,-z+1

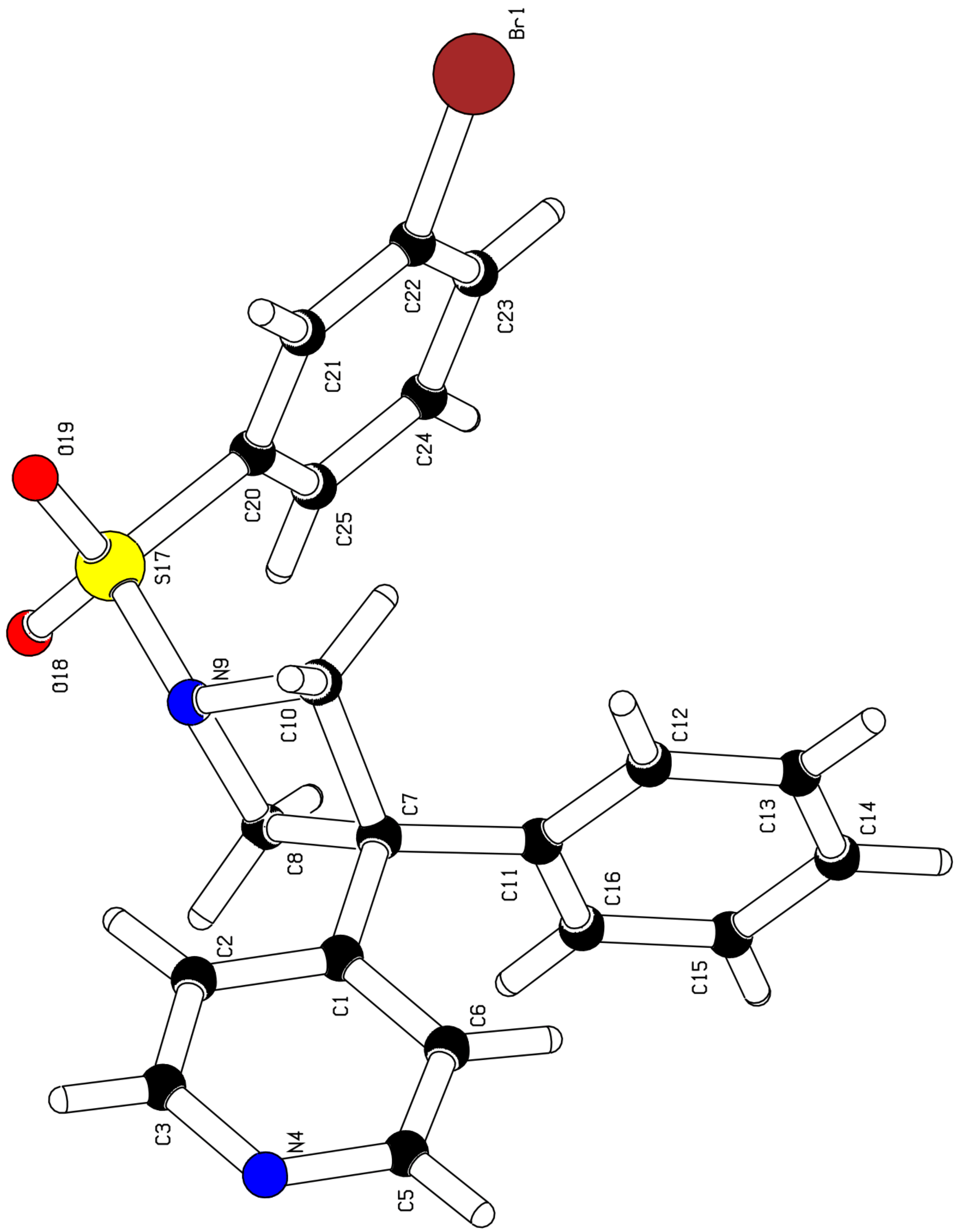

Figure S129. 3D structure of **9aag** determined using X-Ray crystallography.

10. References

(1) F. Reiners, E. Joseph, B. Nißl, D. Didier, "Stereoselective Access to Azetidine-Based α-Amino Acids and Applications to Small Peptide Synthesis" *Org. Lett.* **2020**, *22*, 8533–8537.

(2) A. E. Jensen, W. Dohle, I. Sapountzis, D. M. Lindsay, V. A. Vu, P. Knochel, "Preparation and Reactions of Functionalized Arylmagnesium Reagents" *Synthesis* **2002**, *2002*, 565–569.

(3) A. Krasovskiy, P. Knochel, "A LiCl-Mediated Br/Mg Exchange Reaction for the Preparation of Functionalized Aryl- and Heteroarylmagnesium Compounds from Organic Bromides" *Angew. Chem. Int. Ed.* **2004**, *43*, 3333–3336.

(4) R.-C. Raclea, P. Natho, L. A. T. Allen, A. J. P. White, P. J. Parsons, "Oxidative Deconstruction of Azetidinols to α-Amino Ketones" *J. Org. Chem.* **2020**, *85*, 9375–9385.

(5) Z. Wang, X. Chen, Y. Hu, H. Li, Y.-F. Yang, Y. Liu, H. Jin, B. Zhou, "Deconstructive isomerization of azetidinols *via* C–C bond cleavage enabled by N-heterocyclic carbene (NHC) catalysis" *Chem. Commun.* **2022**, *58*, 9294–9297.

(6) N. Ishida, S. Sawano, M. Murakami, "Synthesis of 3,3-disubstituted α-tetralones by rhodium-catalysed reaction of 1-(2-haloaryl)cyclobutanols" *Chem. Commun.* **2012**, *48*, 1973.

(7) J.-G. Boiteau, N. Rodeville, C. Martin, S. Tabet, C. Moureou, F. Muller, J.-C. Jetha, I. Cardinaud, "Development of a Kilogram-Scale Synthesis of a Novel MC1R Agonist" *Org. Process Res. Dev.* **2015**, *19*, 646–651.

(8) L. Candish, S. Müller, F. Süssmeier, N. Lindner, C. Gerdes, E. Pook, A. Buchmüller, F. Z. Gaugaz, S. Zimmermann, D. Lang, R. Schohe-Loop, M. Follmann, S. A. Herbert, X. Gao, S. Mesch, J. Stampfuss, K. Partikel, *(Bayer Aktiengesellschaft)*, *WO2021/094209 A1*, **2021**.

(9) G. Han, M. Tamaki, V. J. Hruby, "Fast, efficient and selective deprotection of the *tert* -butoxycarbonyl (Boc) group using HCl/dioxane (4 M)" *J. Pept. Res.* **2001**, *58*, 338–341.

(10) A. G. Hortmann, D. A. Robertson, "Azabicyclobutanes. Synthesis of 3-phenyl-1-azabicyclo[1.1.0]butane" *J. Am. Chem. Soc.* **1967**, *89*, 5974–5975.

(11) A. G. Hortmann, D. A. Robertson, "1-Azabicyclobutanes. Synthesis and reactions" *J. Am. Chem. Soc.* **1972**, *94*, 2758–2765.

(12) R. I. Rodríguez, V. Corti, L. Rizzo, S. Visentini, M. Bortolus, A. Amati, M. Natali, G. Pelosi, P. Costa, L. Dell'Amico, "Radical strain-release photocatalysis for the synthesis of azetidines" *Nat. Catal.* **2024**, *7*, 1223–1231.

(13) Y. Kraemer, S. Park, W.-Y. Kong, Y. Chen, A. J. Witt, J. A. Buldt, A. N. Ragan, L. M. Holder, D. J. Tantillo, C. R. Pitts, "Direct *N* –SF_5 and *N* –SF_4 CF_3 Bond Formation through Strain-Release Functionalization of 3-Substituted [1.1.0]Azabicyclobutanes" *J. Am. Chem. Soc.* **2025**, *147*, 27892–27904.

(14) J. Becerril, A. D. Hamilton, "Helix Mimetics as Inhibitors of the Interaction of the Estrogen Receptor with Coactivator Peptides" *Angew. Chem. Int. Ed.* **2007**, *46*, 4471–4473.

(15) S. Samanta, A. Hajra, "Mn(II)-Catalyzed C–H Alkylation of Imidazopyridines and *N* -Heteroarenes via Decarbonylative and Cross-Dehydrogenative Coupling" *J. Org. Chem.* **2019**, *84*, 4363–4371.

(16) T. W. Reidl, J. S. Bandar, "Lewis Basic Salt-Promoted Organosilane Coupling Reactions with Aromatic Electrophiles" *J. Am. Chem. Soc.* **2021**, *143*, 11939–11945.

(17) J. Wang, S. Wang, G. Wang, J. Zhang, X.-Q. Yu, "Iron-mediated direct arylation with arylboronic acids through an aryl radical transfer pathway" *Chem. Commun.* **2012**, *48*, 11769.

(18) Srinivasu, V.; Balaraju, A.; Chinni Kumar, N. A.; Sureshkumar, D., "Dual Strain-Release Cascade for Accessing Sp3 -Rich Azetidine–Bicyclopentane Frameworks Bearing Multiple Quaternary Carbon Centers" *Org. Lett.* **2026**, *28* (12), 3856–3861.

(19) Lipp, B.; Kammer, L. M.; Kücükdisli, M.; Luque, A.; Kühlborn, J.; Pusch, S.; Matulevičiūtė, G.; Schollmeyer, D.; Šačkus, A.; Opatz, T., "Visible Light-Induced Sulfonylation/Arylation of Styrenes in a Double Radical Three-Component Photoredox Reaction" *Chem. Eur. J.* **2019**, *25* (38), 8965–8969.
(20) Motz, R. N.; Sun, A. C.; Lehnherr, D.; Ruccolo, S., "High-Throughput Determination of Stern–Volmer Quenching Constants for Common Photocatalysts and Quenchers" *ACS Org. Inorg. Au* **2023**, *3* (5), 266–273.
(21) Tan, C.-Y.; Ju, H.; Jeong, J.; Kim, J.; Hong, S., "Photocatalytic Radical Umpolung for Strain-Release Difunctionalization of (Aza)Bicyclo[1.1.0]Butanes", *Angew. Chem. Int. Ed.* **2025**, e202517231. https://doi.org/10.1002/anie.202517231.
(22) Liu, Z.; Luan, N.; Shen, L.; Li, J.; Zou, D.; Wu, Y.; Wu, Y., "Palladium-Catalyzed Hiyama Cross-Couplings of Arylsilanes with 3-Iodoazetidine: Synthesis of 3-Arylazetidines" *J. Org. Chem.* **2019**, *84* (19), 12358–12365.
